# Supplementary material for: IGF2BP3 recognizes m6A to regulate histone-to-protamine replacement during mouse sperm development
Source: EMBO J. 2025 Dec 5;45(2):504–36. doi: 10.1038/s44318-025-00659-y (PMC12811620; doi:10.1038/s44318-025-00659-y)
Supplement: Supplementary file 1 — Appendix [file 44318_2025_659_MOESM1_ESM.pdf]

Table of contents

Page 2: Appendix Figure S1. Dynamic gene expression patterns of m^{6}A binding proteins in mouse testicular germ cells and somatic cells as well as the location of IGF2BP3 in the epididymis, related to Figure 1.

Page 4: Appendix Figure S2. The proportion of spermatogonia, spermatocytes and Sertoli cells in *Igf2bp3*$^{-/-}$ testes, related to Figure 2.

Page 6: Appendix Figure S3. Quality assessment of sequencing data, and colocalization of IGF2BP3 and HDAC11 (DOT1L) in mouse testes, related to Figure 4.

Page 8: Appendix Figure S4. IGF2BP3 represses RNA translation via the interaction with YBX2, related to Figure 5.

Page 10: Appendix Figure S5. IGF2BP3-YBX2 complex reduced the translation efficiency of CopGFP regulated by the 3′UTR of *Hdac11* containing mutant m^{6}A motifs, related to Figure 6.

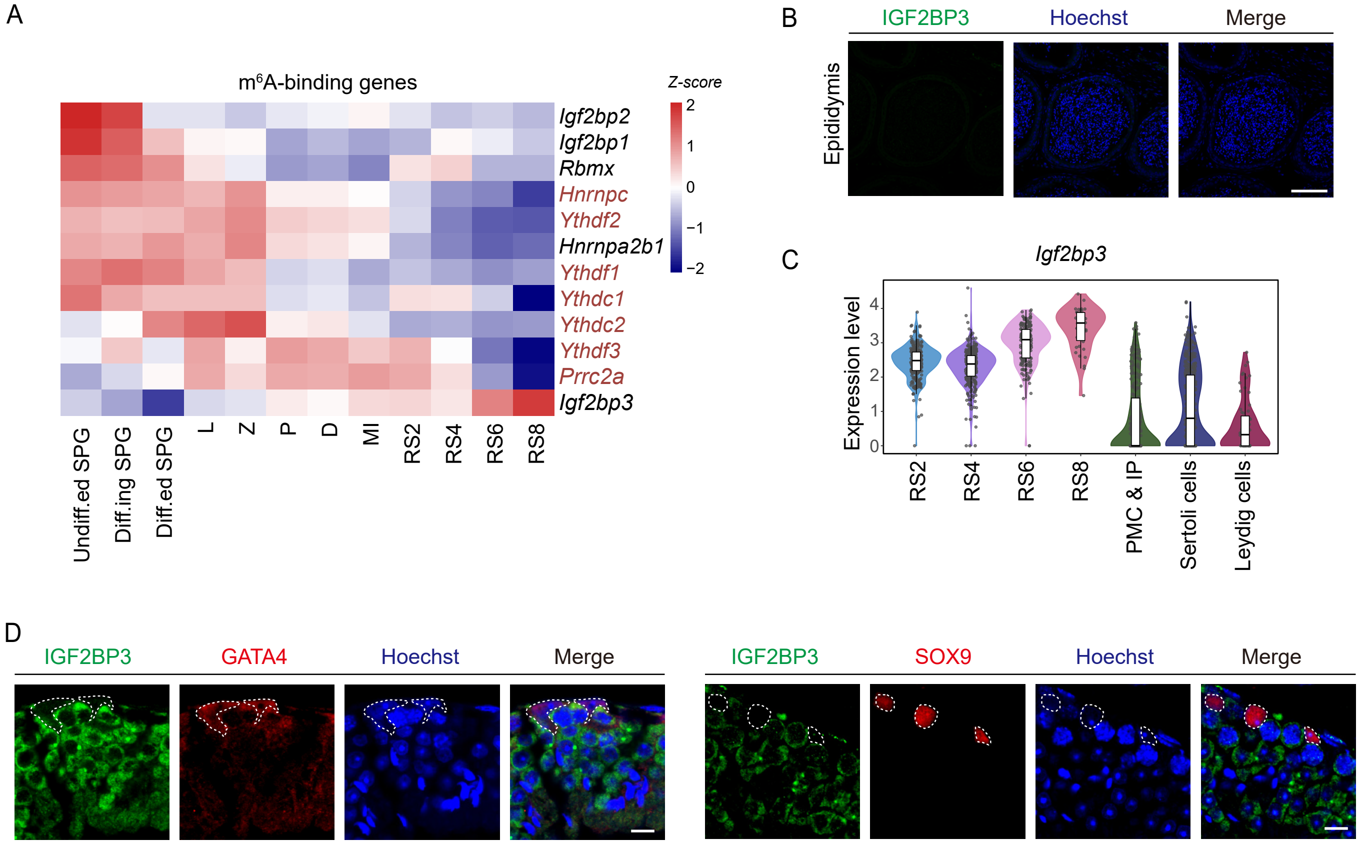
A
m6A-binding genes
Z-score
2
1
0
−1
−2
Igf2bp2
Igf2bp1
Rbmx
Hnrnpc
Ythdf2
Hnrnpa2b1
Ythdf1
Ythdc1
Ythdc2
Ythdf3
Prrc2a
Igf2bp3
Undiff.ed SPG
Diff.ing SPG
Diff.ed SPG
L
Z
P
D
MI
RS2
RS4
RS6
RS8
B
IGF2BP3
Hoechst
Merge
Epididymis
C
Igf2bp3
Expression level
0
1
2
3
4
RS2
RS4
RS6
RS8
PMC & IP
Sertoli cells
Leydig cells
D
IGF2BP3
GATA4
Hoechst
Merge
IGF2BP3
SOX9
Hoechst
Merge

Appendix Figure S1. Dynamic gene expression patterns of m^{6}A binding proteins in mouse testicular germ cells and somatic cells as well as the location of IGF2BP3 in the epididymis, related to Figure 1.

(**A**) Heatmap of reported m^{6}A-binding genes in mouse spermatogenic cells. m^{6}A, N^6-methyladenosine. Undiff.ed SPG, Undifferentiated spermatogonia; Diff.ing SPG, Differentiating spermatogonia; Diff.ed SPG, Differentiated spermatogonia; L, leptotene; Z, zygotene; P, pachytene; D, diplotene; MI, metaphase I; RS, round spermatids.

(**B**) Immunofluorescence of IGF2BP3 in adult caudal epididymis sections. Scale bar, 50 µm.

(**C**) Violin plot showing the relative expression levels [log (TPM/10+1)] of *Igf2bp3* in mouse round spermatids and neonatal testicular somatic cells. Each box within the violin plots represents the median and the 25% and 75% quartiles, and the whiskers indicate 1.5 times the interquartile range. Each dot corresponds to a single cell, data from two biologically independent samples (integration of all samples). PMC & IP, peritubular myoid cells & interstitial progenitors.

(**D**) Immunofluorescence of IGF2BP3 (green), GATA4 (red, left) and SOX9 (red, right) in adult testicular paraffin sections from 8-week-old mice. Scale bar, 10 µm. The dotted border demarcates the boundary of positive signals for GFTA4 or SOX9.

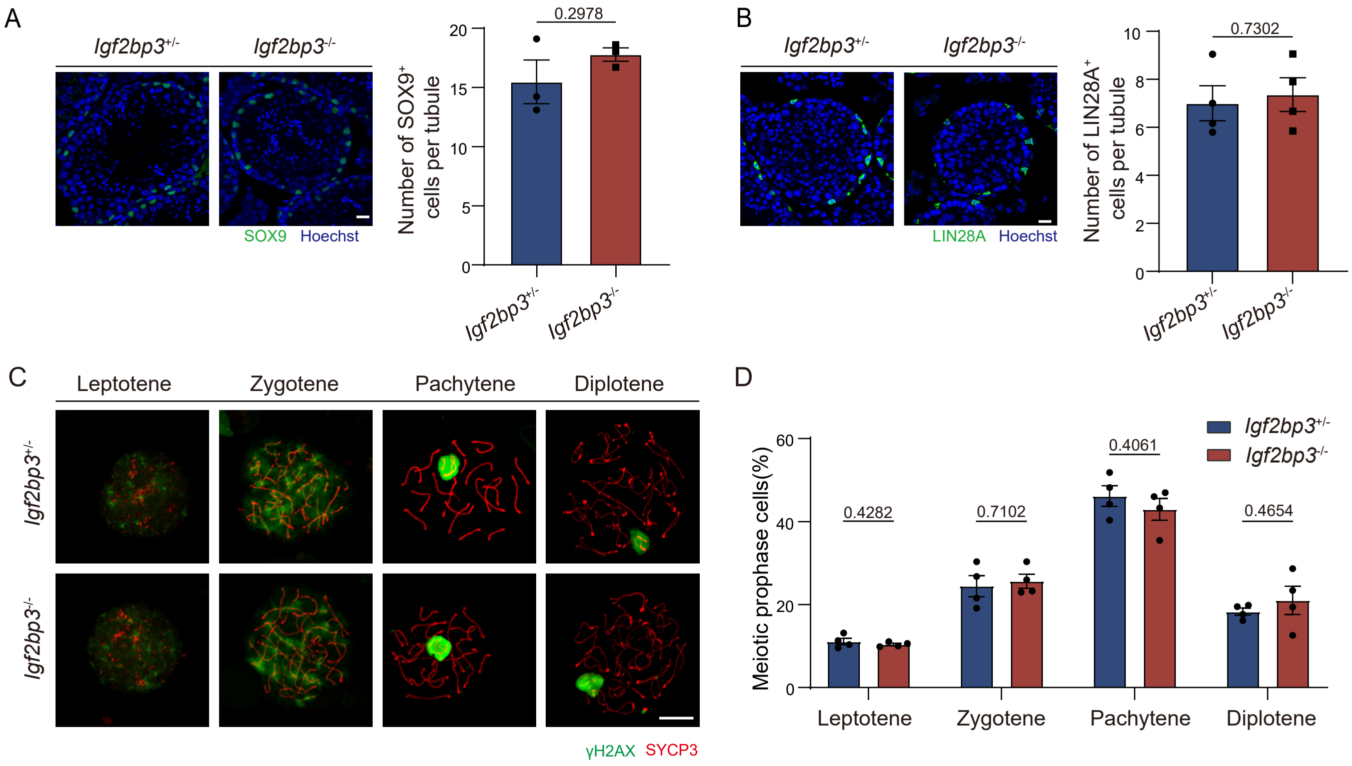
A
Igf2bp3+/-
Igf2bp3-/-
SOX9 Hoechst
Number of SOX9+ cells per tubule
0.2978
0
5
10
15
20
B
Igf2bp3+/-
Igf2bp3-/-
LIN28A Hoechst
Number of LIN28A+ cells per tubule
0.7302
0
2
4
6
8
10
C
Leptotene
Zygotene
Pachytene
Diplotene
γH2AX SYCP3
D
Meiotic prophase cells(%)
0
20
40
60
0.4282
0.7102
0.4061
0.4654
Leptotene
Zygotene
Pachytene
Diplotene

Appendix Figure S2. The proportion of spermatogonia, spermatocytes and Sertoli cells in *Igf2bp3*$^{-/-}$ testes, related to Figure 2.

(**A**) Quantitative comparison of SOX9^{+} cells per tubule between adult *Igf2bp3*$^{+/-}$ and *Igf2bp3*$^{-/-}$ testes. At least 20 tubules for each mouse were counted. Unpaired two-tailed *t*-test. Error bars, n = 3 biological replicates, mean ± SEM. Scale bar, 20 μm.

(**B**) Quantitative comparison of LIN28A^{+} cells per tubule between adult *Igf2bp3*$^{+/-}$ and *Igf2bp3*$^{-/-}$ testes. At least 20 tubules for each mouse were counted. Scale bar, 20 μm. Unpaired two-tailed *t*-test. Error bars, n = 4 biological replicates, mean ± SEM. Scale bar, 20 μm.

(**C**) Immunostaining of γH2AX (green) and SYCP3 (red) on chromosome spreads of *Igf2bp3*$^{+/-}$ and *Igf2bp3*$^{-/-}$ spermatocytes. Scale bar, 10 μm.

(**D**) Proportion of leptotene, zygotene, pachytene, and diplotene spermatocytes in adult *Igf2bp3*$^{+/-}$ and *Igf2bp3*$^{-/-}$ spermatocytes. Unpaired two-tailed *t*-test. Error bars, n = 4 biological replicates, mean ± SEM.

A

	ENCODE Standards	Data Sets
Unique fragments	> 1,000,000	IP1 (8,157,184)<br>SMInput1 (6,731,942)<br>IP2 (7,866,228)<br>SMInput2 (20,839,473)
FRiP	> 0.005	Rep1 (0.078)<br>Rep2 (0.181)
IDR	< 2	Rescue ratio (1.75)<br>Self-consistency ratio (1.13)

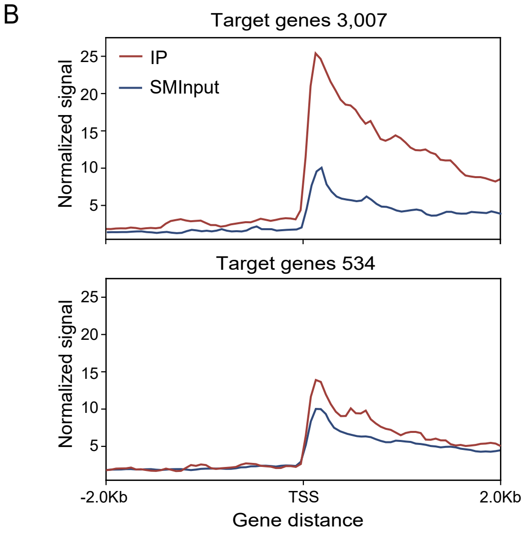

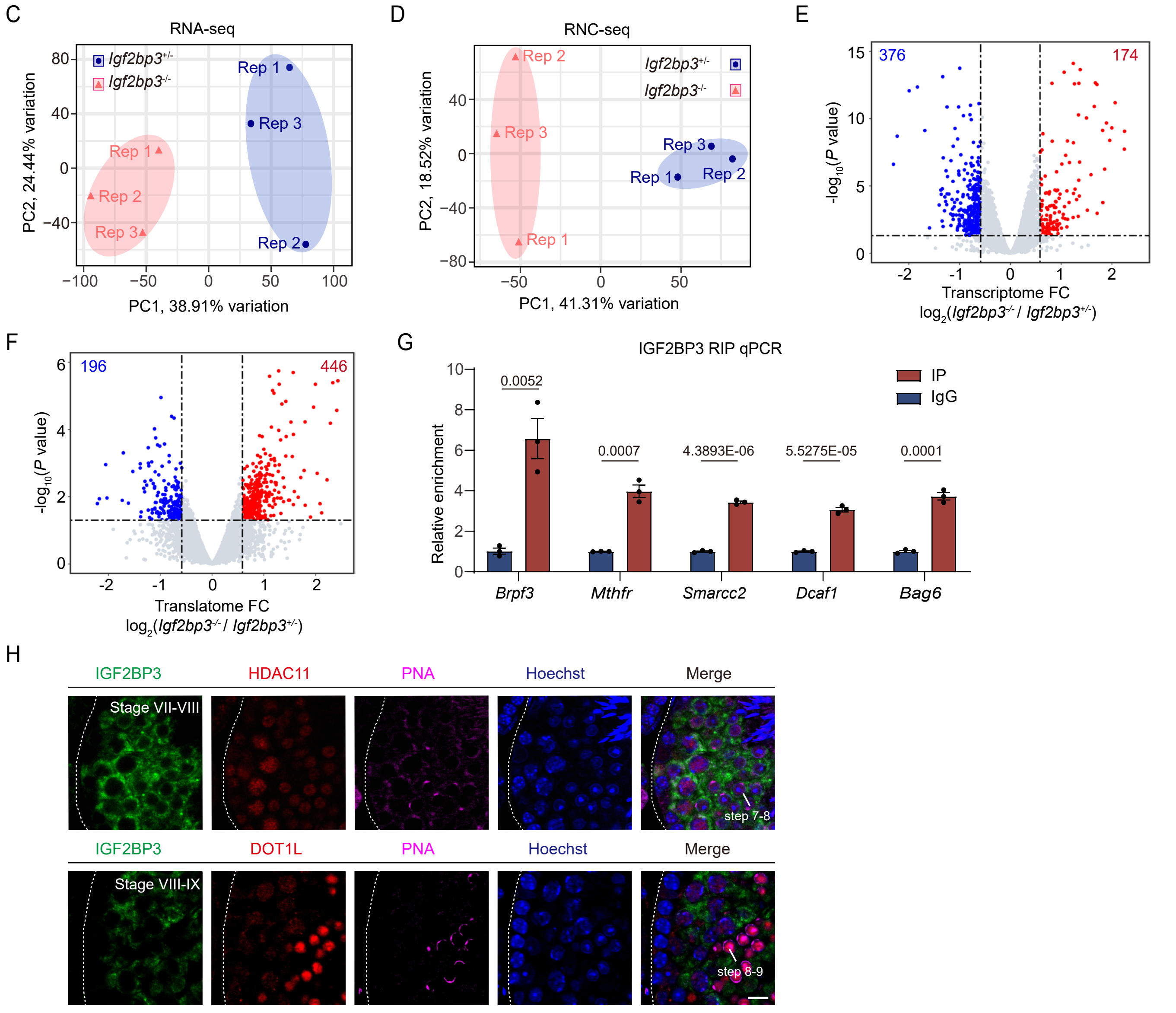

Appendix Figure S3. Quality assessment of sequencing data, and colocalization of IGF2BP3 and HDAC11 (DOT1L) in mouse testes, related to Figure 4.

(**A**) Assessment of eCLIP-seq data quality using the standardized evaluation protocol from the ENCODE project. FRiP (Fraction of reads in peaks), IDR (Irreproducible Discovery Rate).

(**B**) Aggregated IGF2BP3 eCLIP-seq signals at 534 target genes highly expressed in both germ and somatic cells upon 3,007 germ cell-specific target genes.

(**C**, **D**) Principal Component Analysis (PCA) plots of the RNA-seq and RNC-seq data from adult *Igf2bp3*$^{+/-}$ and *Igf2bp3*$^{-/-}$ round spermatids. Each dot represents one sample.

(**E**, **F**) Volcano plots showing the differential expression genes (DEGs) in transcriptome (E) and translatome (F) profiles of adult round spermatids comparing adult *Igf2bp3*$^{+/-}$ vs *Igf2bp3*$^{-/-}$ round spermatids. DEGs were identified by DESeq2 (Wald test) with thresholds of $P < 0.05$ and $|\log_2$ (fold change)$| > \log_2(1.5)$, n = 3 biological replicates. Red and blue dots indicate up-regulated and down-regulated genes, respectively.

(**G**) IGF2BP3 RIP-qPCR analysis of the relatively enrichment of indicated transcripts in adult WT mouse testes. Unpaired two-tailed *t*-test. Error bars, n = 3 biological replicates, mean ± SEM.

(**H**) Immunofluorescence of IGF2BP3 (green), HDAC11 (red, top), DOT1L (red, bottom), and PNA (magenta) in adult WT testicular paraffin sections from 8-week-old mice. Scale bar, 10 μm. Dotted borders demarcates the basement membrane of the seminiferous tubule.

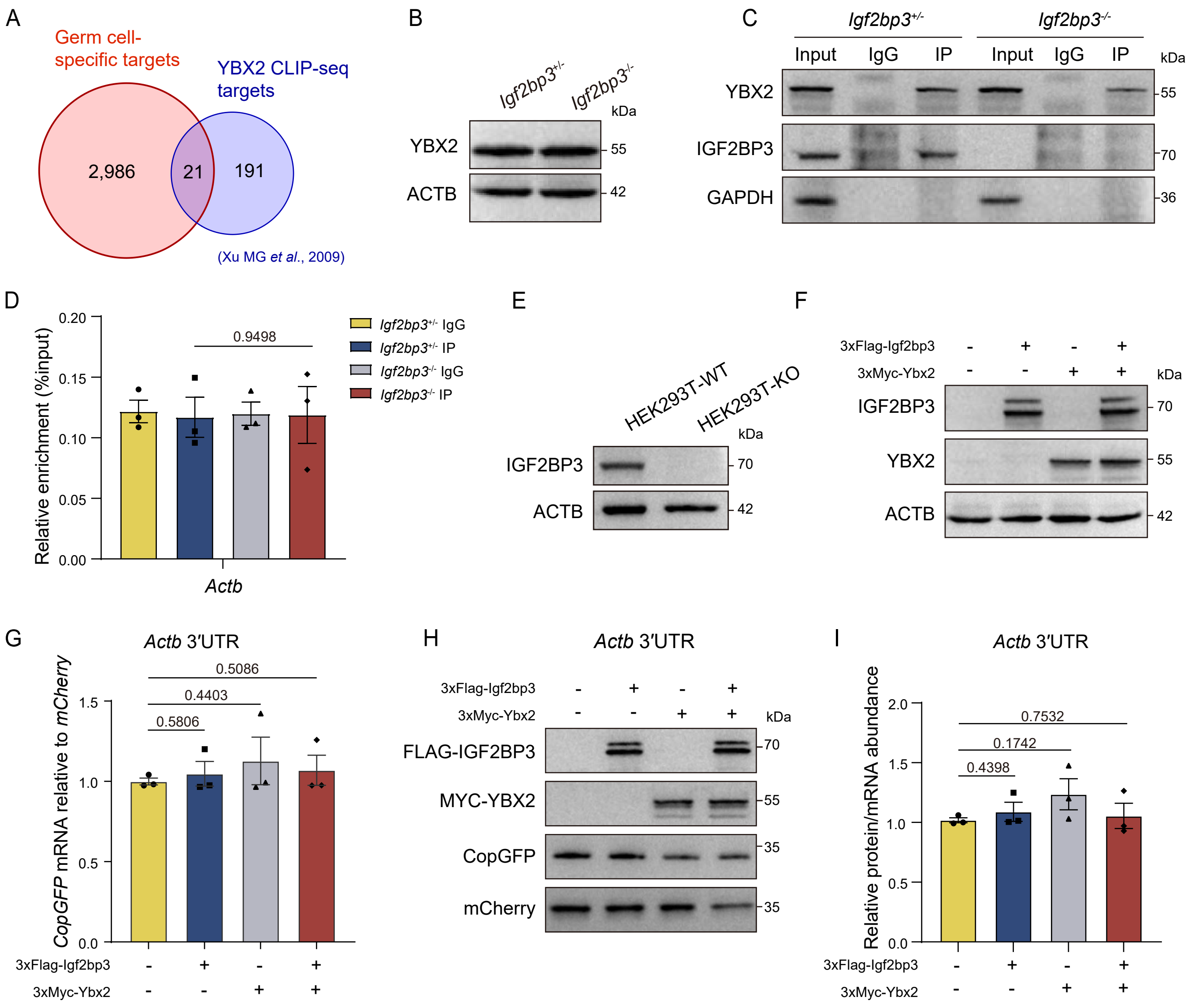
A
Germ cell-specific targets
YBX2 CLIP-seq targets
2,986
21
191
(Xu MG et al., 2009)
B
Igf2bp3+/-
Igf2bp3-/-
kDa
YBX2
ACTB
55
42
C
Igf2bp3+/-
Igf2bp3-/-
Input
IgG
IP
Input
IgG
IP
kDa
YBX2
IGF2BP3
GAPDH
55
70
36
D
Relative enrichment (%input)
0.20
0.15
0.10
0.05
0.00
0.9498
Actb
Igf2bp3+/- IgG
Igf2bp3+/- IP
Igf2bp3-/- IgG
Igf2bp3-/- IP
E
HEK293T-WT
HEK293T-KO
kDa
IGF2BP3
ACTB
70
42
F
3xFlag-Igf2bp3
3xMyc-Ybx2
kDa
IGF2BP3
YBX2
ACTB
70
55
42
G
Actb 3'UTR
CopGFP mRNA relative to mCherry
1.5
1.0
0.5
0.0
0.5086
0.4403
0.5806
3xFlag-Igf2bp3
3xMyc-Ybx2
H
Actb 3'UTR
3xFlag-Igf2bp3
3xMyc-Ybx2
kDa
FLAG-IGF2BP3
MYC-YBX2
CopGFP
mCherry
70
55
35
35
I
Actb 3'UTR
Relative protein/mRNA abundance
2.0
1.5
1.0
0.5
0.0
0.7532
0.1742
0.4398
3xFlag-Igf2bp3
3xMyc-Ybx2

Appendix Figure S4. IGF2BP3 represses RNA translation via the interaction with YBX2, related to Figure 5.

(**A**). Venn diagram showing the 3,007 germ cell-specific targets of IGF2BP3 (in the current study) and 212 CLIP-seq targets of YBX2 in mouse testes (Xu MG *et al*., 2009).

(**B**) Western blotting analysis of the protein levels of YBX2 in adult *Igf2bp3*$^{+/-}$ and *Igf2bp3*$^{-/-}$ mouse testes. ACTB serves as a loading control.

(**C**) Western blotting analysis of the YBX2 in adult *Igf2bp3*$^{+/-}$ and *Igf2bp3*$^{-/-}$ mouse testes lysates (input) and lysates immunoprecipitated by YBX2 (IP) or IgG antibodies.

(**D**) YBX2 RIP-qPCR analyses of *Actb* in adult *Igf2bp3*$^{+/-}$ and *Igf2bp3*$^{-/-}$ testes. Unpaired two-tailed *t*-test. Error bars, n = 3 biological replicates, mean ± SEM.

(**E**) Western blotting analysis of the protein levels of IGF2BP3 in wild-type (WT) and IGF2BP3-KO HEK293T cells. ACTB serves as a loading control.

(**F**) Western blotting analysis of the protein levels of IGF2BP3 and YBX2 in four HEK293T lines, corresponding to Fig. 5E. ACTB serves as a loading control.

(**G**) qPCR analyses of the relative levels of *CopGFP* mRNAs under the regulation of 3′UTR of *Actb* normalized to *mCherry* mRNAs. Cell lines were treated with 2 μg/ml of actinomycin D for 2 hours. Unpaired two-tailed *t*-test. Error bars, n = 3 biological replicates, mean ± SEM.

(**H**) Western blotting analysis of the protein levels of CopGFP under the regulation of 3′UTR of *Actb* with the overexpression of FLAG-tagged IGF2BP3 or MYC-tagged YBX2. The level of mCherry is set as the internal control.

(**I**) Histogram showing the ratios of CopGFP proteins (normalized to mCherry proteins) to the *CopGFP* mRNAs (normalized to *mCherry* mRNAs), corresponding to Appendix Fig. S4G,H. Unpaired two-tailed *t*-test. Error bars, n = 3 biological replicates, mean ± SEM.

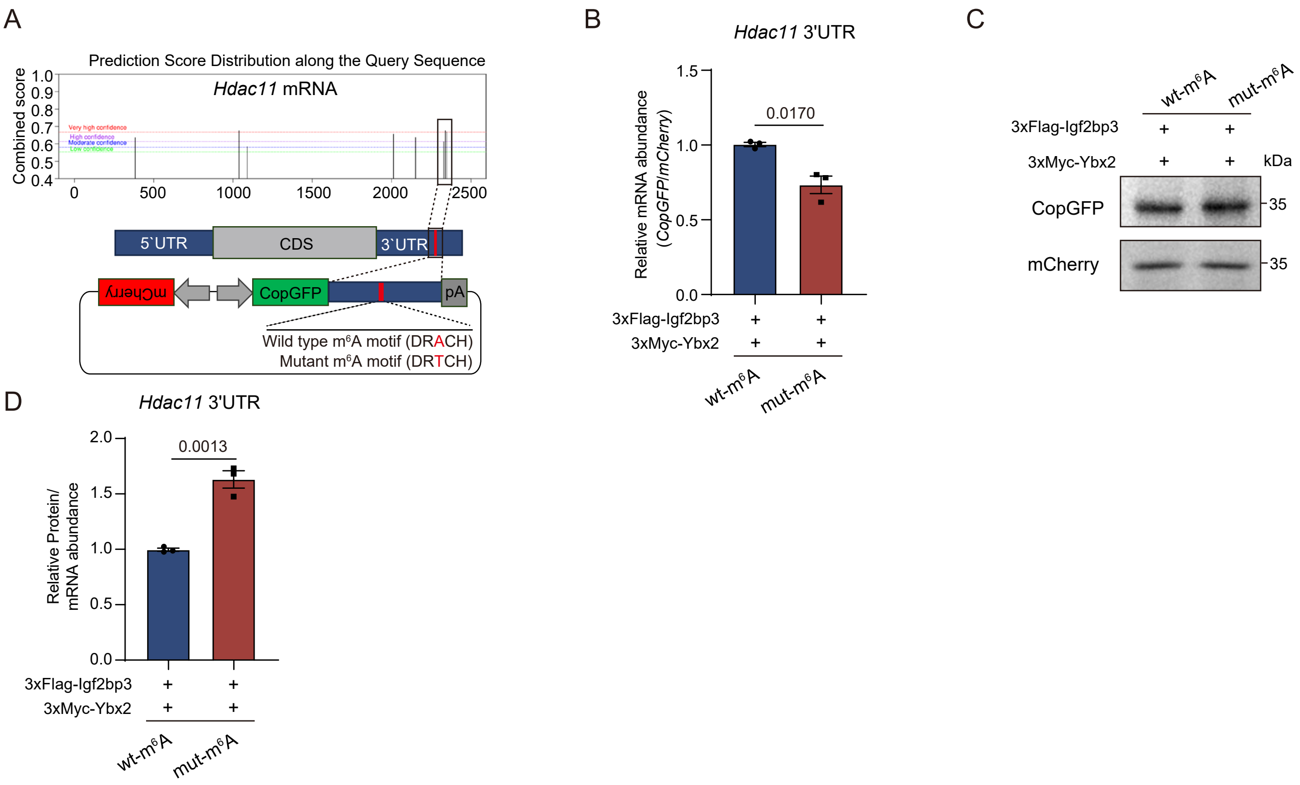
A
Prediction Score Distribution along the Query Sequence
Hdac11 mRNA
Combined score
1.0
0.9
0.8
0.7
0.6
0.5
0.4
0
500
1000
1500
2000
2500
5`UTR
CDS
3`UTR
mCherry
CopGFP
pA
Wild type m6A motif (DRACH)
Mutant m6A motif (DRTCH)
B
Hdac11 3'UTR
Relative mRNA abundance (CopGFP/mCherry)
1.5
1.0
0.5
0.0
0.0170
3xFlag-Igf2bp3 + +
3xMyc-Ybx2 + +
wt-m6A
mut-m6A
C
wt-m6A
mut-m6A
3xFlag-Igf2bp3 + +
3xMyc-Ybx2 + +
kDa
CopGFP
35
mCherry
35
D
Hdac11 3'UTR
Relative Protein/ mRNA abundance
2.0
1.5
1.0
0.5
0.0
0.0013
3xFlag-Igf2bp3 + +
3xMyc-Ybx2 + +
wt-m6A
mut-m6A

Appendix Figure S5. IGF2BP3-YBX2 complex reduced the translation efficiency of CopGFP regulated by the 3′UTR of *Hdac11* containing mutant m^{6}A motifs, related to Figure 6.

(**A**) m^{6}A sites within *Hdac11* mRNAs predicted by SRAMP online software (top). Schematic of bidirectional expression vectors (bottom). CopGFP ligates the m^{6}A-enriched region of *Hdac11* 3′UTR with the overexpression of FLAG-tagged IGF2BP3, and mCherry is used as a transfection control.

(**B**) qPCR analysis of the relative levels of CopGFP mRNA under the regulation of the m^{6}A motif in the *Hdac11* 3′UTR, normalized to *mCherry* mRNA. Cell lines were treated with 2 μg/ml actinomycin D for 2 hours. Unpaired two-tailed *t*-test. Error bars, n = 3 biological replicates, mean ± SEM.

(**C**) Western blotting analysis of CopGFP protein levels in FLAG-tagged IGF2BP3 and MYC-tagged YBX2 overexpressed HEK293T under the control of the m^{6}A motif in the *Hdac11* 3′UTR. The level of mCherry is set as the internal control.

(**D**) Histogram showing the ratios of CopGFP proteins (normalized to mCherry proteins) to the *CopGFP* mRNAs (normalized to *mCherry* mRNAs), corresponding to Appendix Fig. S5B,C. Unpaired two-tailed *t*-test. Error bars, n = 3 biological replicates, mean ± SEM.
