# Supplementary material for: IGF2BP3 recognizes m6A to regulate histone-to-protamine replacement during mouse sperm development
Source: EMBO J. 2025 Dec 5;45(2):504–36. doi: 10.1038/s44318-025-00659-y (PMC12811620; doi:10.1038/s44318-025-00659-y)
Supplement: Supplementary file 10 — Movie EV3 [file 44318_2025_659_MOESM10_ESM.zip › Movie EV3/Movie EV3.docx]

**Movie EV3.** CASA of the Motility and Concentration of *Igf2bp3*^-/-^ Epididymal Spermatozoa, Related to Figure 2. The Movie was Shot with 50 Frames Per Second.
