# Supplementary material for: IGF2BP3 recognizes m6A to regulate histone-to-protamine replacement during mouse sperm development
Source: EMBO J. 2025 Dec 5;45(2):504–36. doi: 10.1038/s44318-025-00659-y (PMC12811620; doi:10.1038/s44318-025-00659-y)
Supplement: Supplementary file 12 — Movie EV5 [file 44318_2025_659_MOESM12_ESM.zip › Movie EV5/Movie EV5.docx]

**Movie EV5.** CASA of the Motility and Concentration of Epididymal Spermatozoa from *Igf2bp3*^-/-^ Mouse Injected with siNC, Related to Figure 7. The Movie was Shot with 50 Frames Per Second.
