# Supplementary material for: IGF2BP3 recognizes m6A to regulate histone-to-protamine replacement during mouse sperm development
Source: EMBO J. 2025 Dec 5;45(2):504–36. doi: 10.1038/s44318-025-00659-y (PMC12811620; doi:10.1038/s44318-025-00659-y)
Supplement: Supplementary file 14 — Source data Fig. [file 44318_2025_659_MOESM14_ESM.zip › EMBOJ-2025-121587_Source Data/Source Data Figure 7/SD FIgure 7B/SD Figure 7B.pdf]

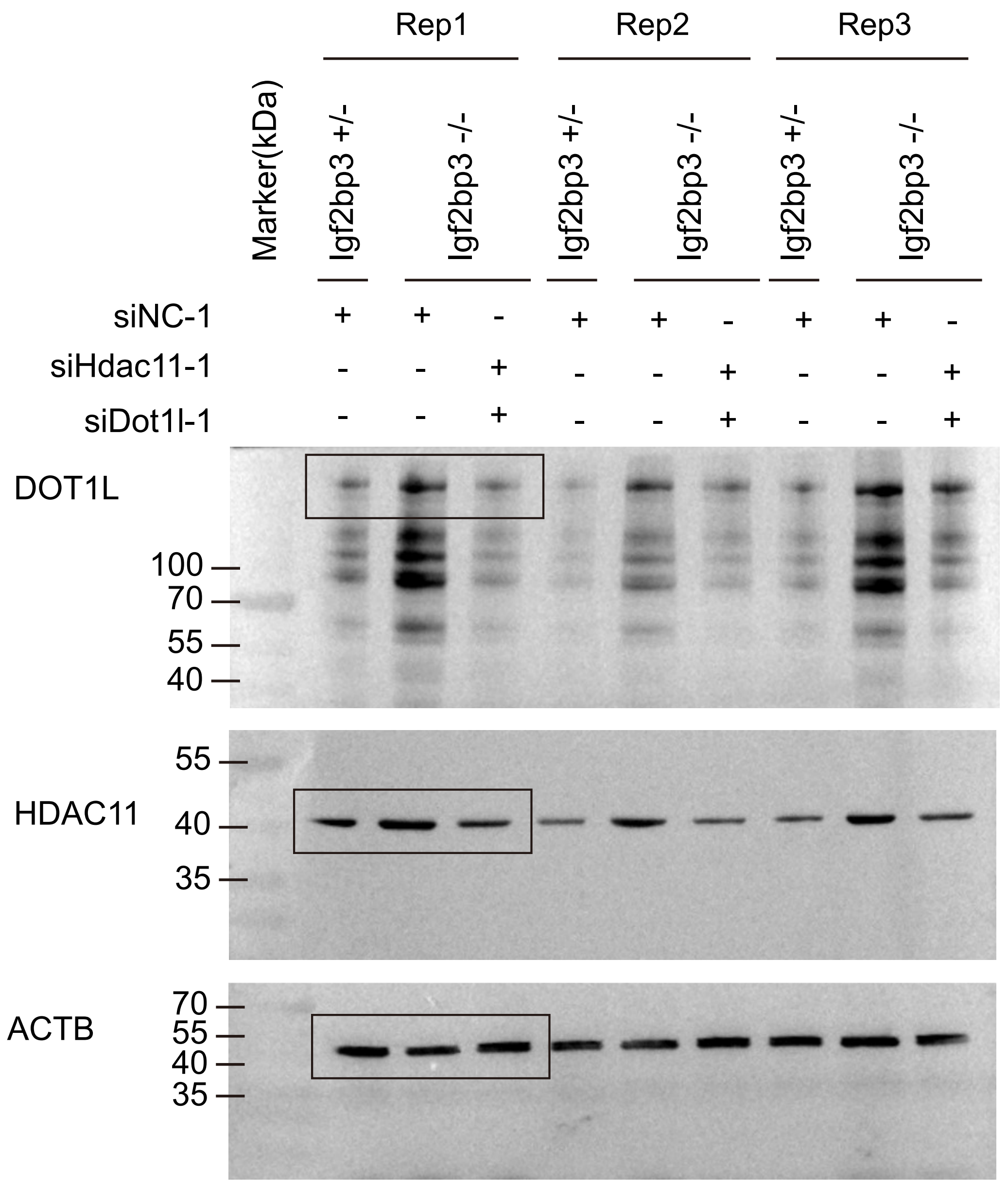
Rep1
Rep2
Rep3
Marker(kDa)
Igf2bp3 +/-
Igf2bp3 -/-
siNC-1
siHdac11-1
siDot1l-1
DOT1L
100
70
55
40
HDAC11
55
40
35
ACTB
70
55
40
35
