# Supplementary figures and images for: IGF2BP3 recognizes m6A to regulate histone-to-protamine replacement during mouse sperm development (part 2 of 3)
Source: EMBO J. 2025 Dec 5;45(2):504–36. doi: 10.1038/s44318-025-00659-y (PMC12811620; doi:10.1038/s44318-025-00659-y)

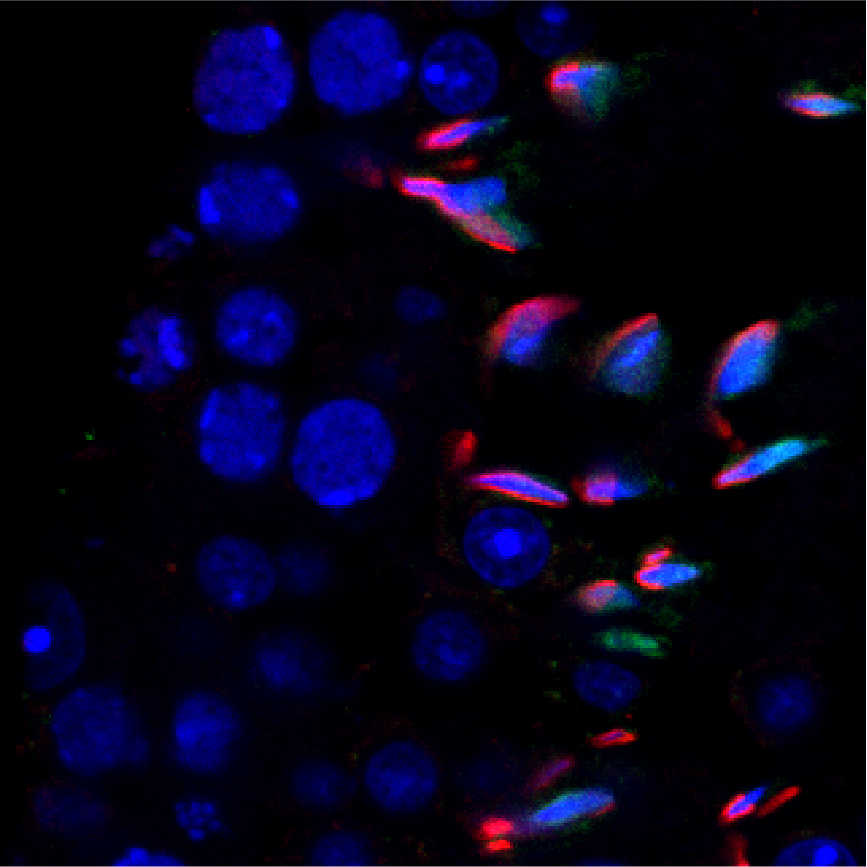

Supplement: Supplementary file 14 — Source data Fig. [file 44318_2025_659_MOESM14_ESM.zip › EMBOJ-2025-121587_Source Data/Source Data Figure 3/SD Figure 3F/Igf2bp3+/- Merge.jpg]

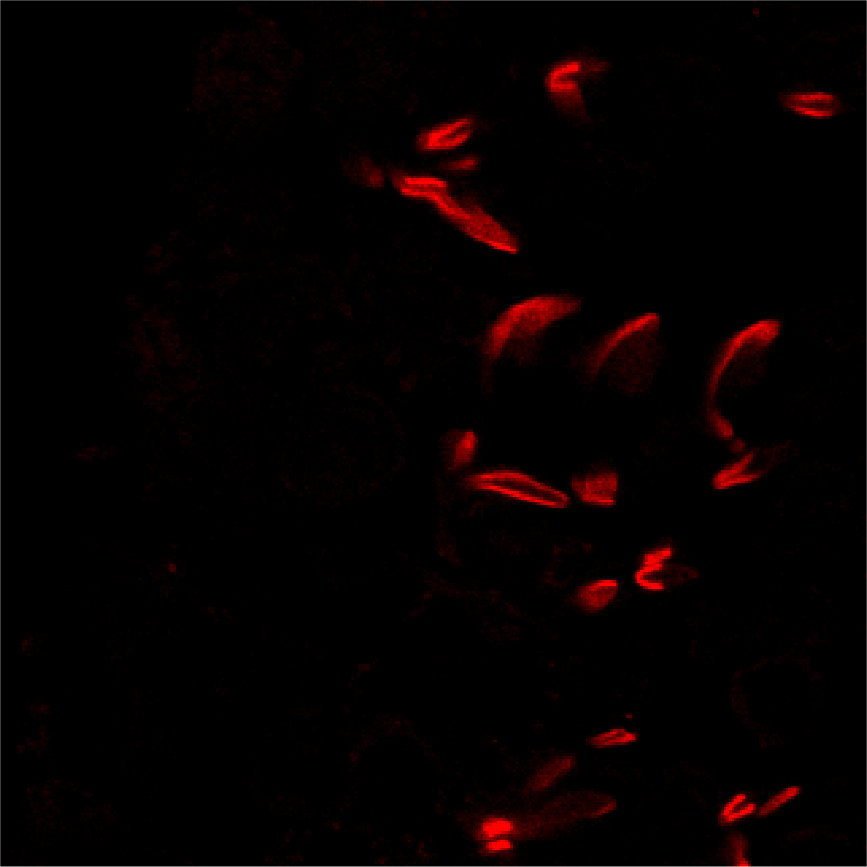

Supplement: Supplementary file 14 — Source data Fig. [file 44318_2025_659_MOESM14_ESM.zip › EMBOJ-2025-121587_Source Data/Source Data Figure 3/SD Figure 3F/Igf2bp3+/- PNA.jpg]

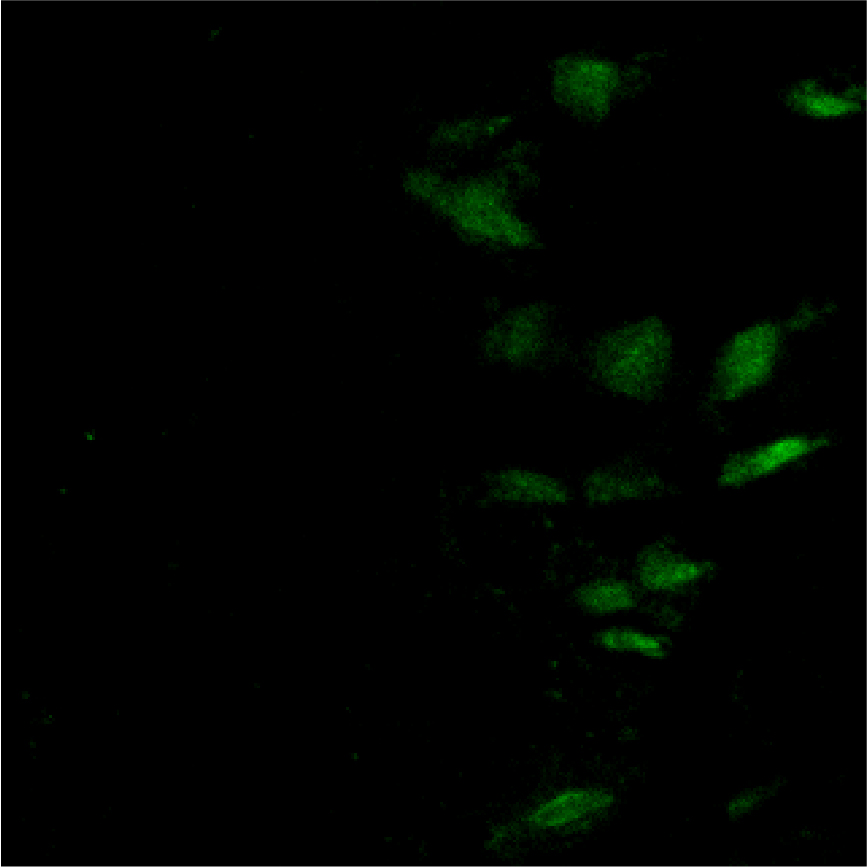

Supplement: Supplementary file 14 — Source data Fig. [file 44318_2025_659_MOESM14_ESM.zip › EMBOJ-2025-121587_Source Data/Source Data Figure 3/SD Figure 3F/Igf2bp3+/- TNP2.jpg]

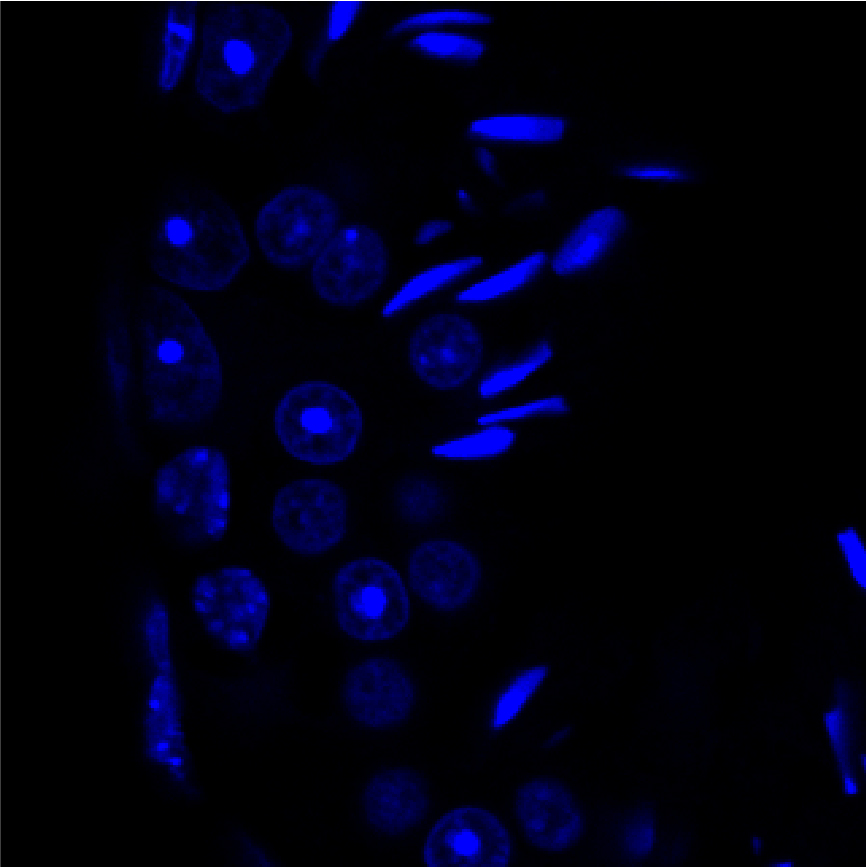

Supplement: Supplementary file 14 — Source data Fig. [file 44318_2025_659_MOESM14_ESM.zip › EMBOJ-2025-121587_Source Data/Source Data Figure 3/SD Figure 3F/Igf2bp3-/- Hoechst.jpg]

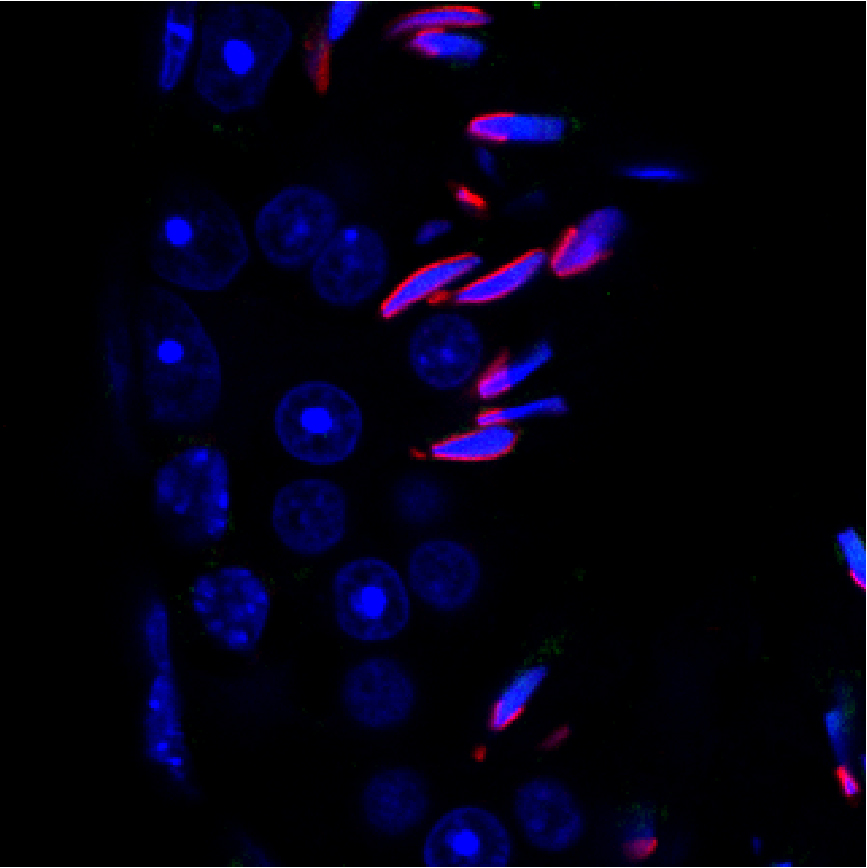

Supplement: Supplementary file 14 — Source data Fig. [file 44318_2025_659_MOESM14_ESM.zip › EMBOJ-2025-121587_Source Data/Source Data Figure 3/SD Figure 3F/Igf2bp3-/- Merge.jpg]

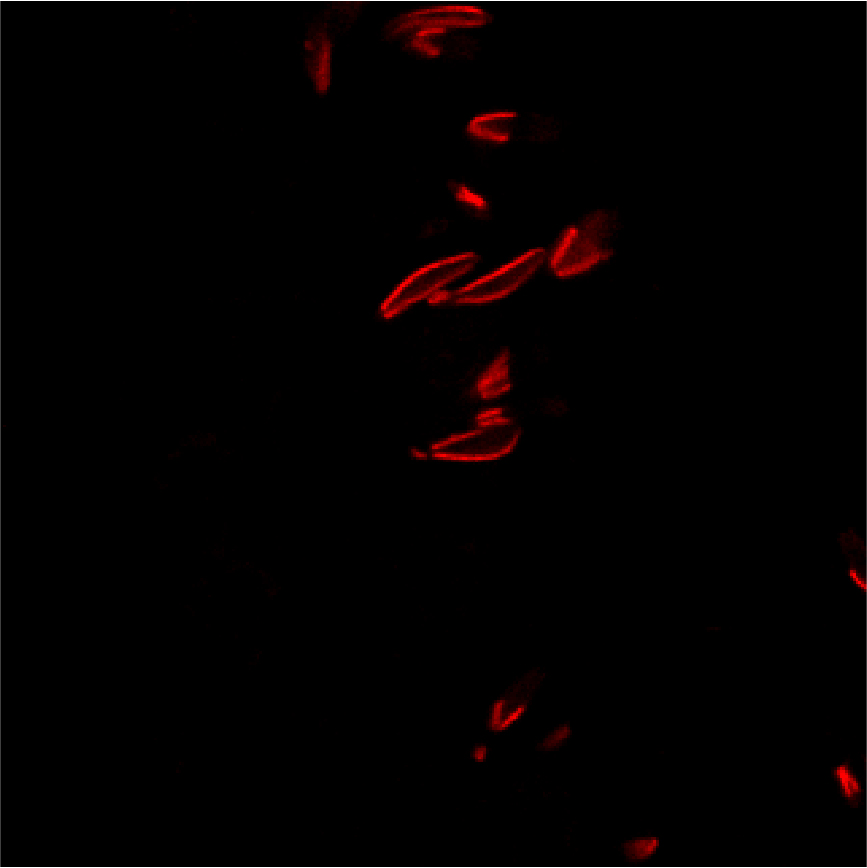

Supplement: Supplementary file 14 — Source data Fig. [file 44318_2025_659_MOESM14_ESM.zip › EMBOJ-2025-121587_Source Data/Source Data Figure 3/SD Figure 3F/Igf2bp3-/- PNA.jpg]

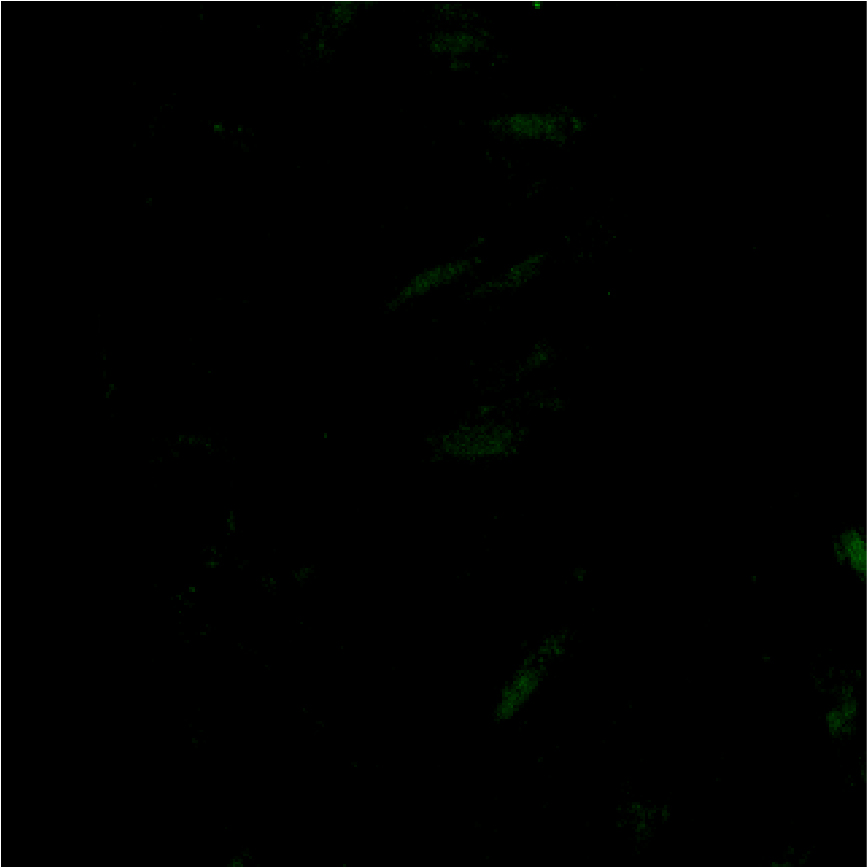

Supplement: Supplementary file 14 — Source data Fig. [file 44318_2025_659_MOESM14_ESM.zip › EMBOJ-2025-121587_Source Data/Source Data Figure 3/SD Figure 3F/Igf2bp3-/- TNP2.jpg]

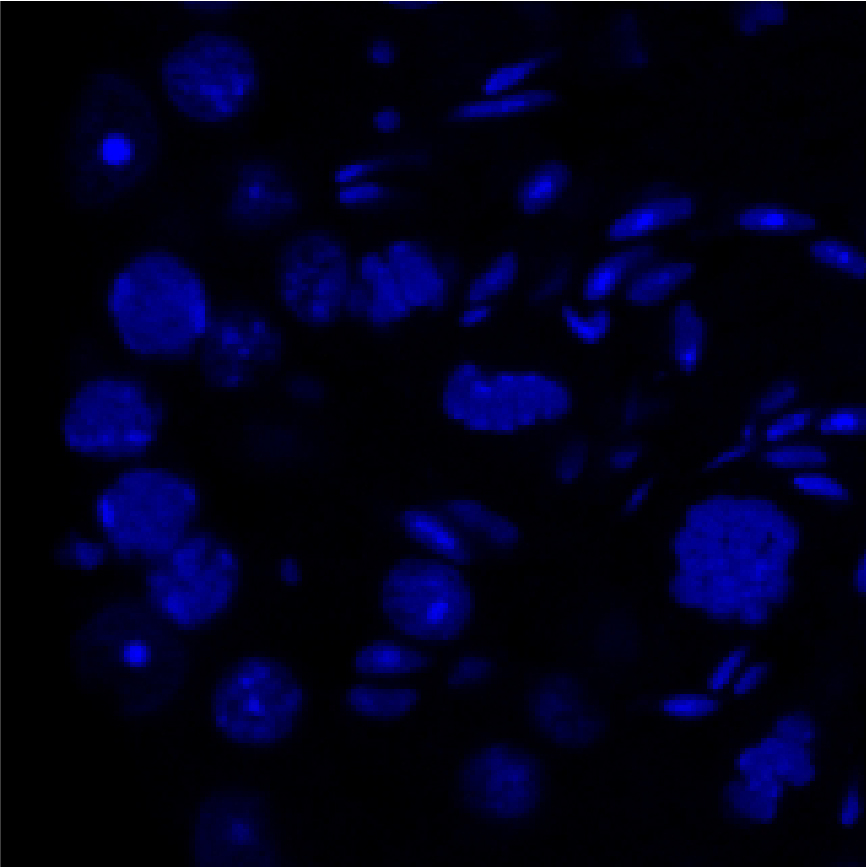

Supplement: Supplementary file 14 — Source data Fig. [file 44318_2025_659_MOESM14_ESM.zip › EMBOJ-2025-121587_Source Data/Source Data Figure 3/SD Figure 3G/Igf2bp3+/- Hoechst.jpg]

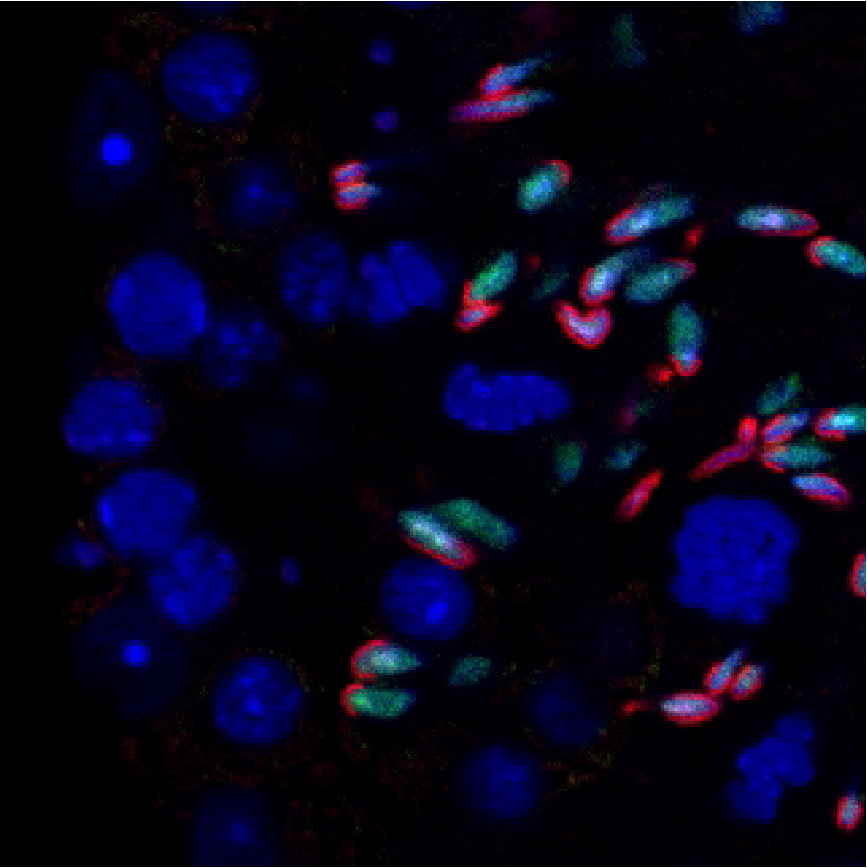

Supplement: Supplementary file 14 — Source data Fig. [file 44318_2025_659_MOESM14_ESM.zip › EMBOJ-2025-121587_Source Data/Source Data Figure 3/SD Figure 3G/Igf2bp3+/- Merge.jpg]

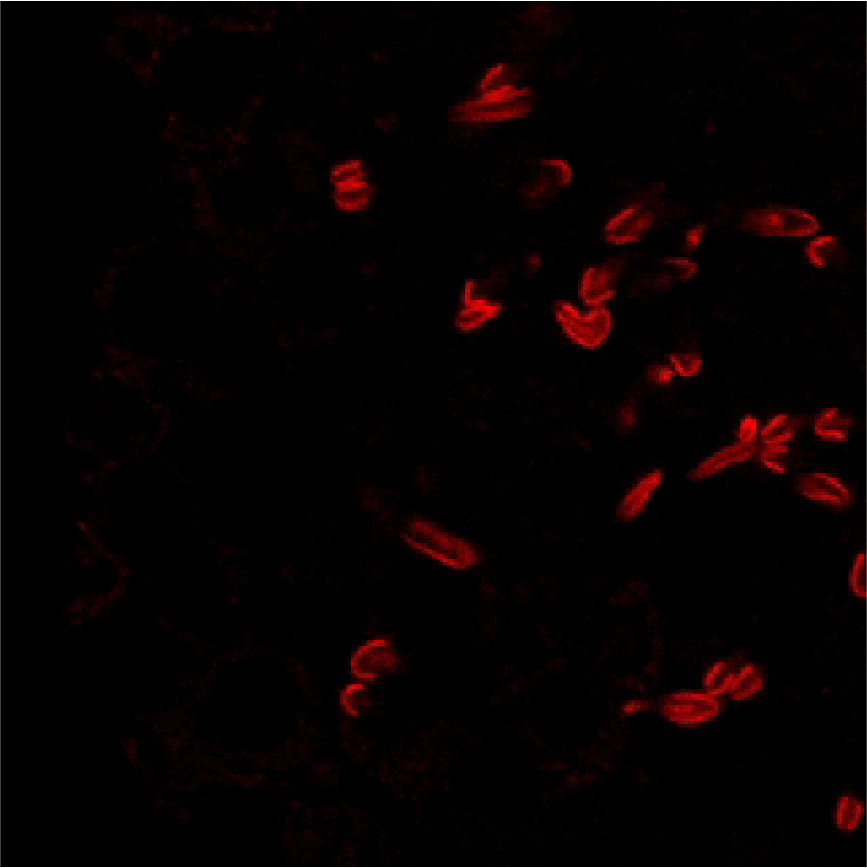

Supplement: Supplementary file 14 — Source data Fig. [file 44318_2025_659_MOESM14_ESM.zip › EMBOJ-2025-121587_Source Data/Source Data Figure 3/SD Figure 3G/Igf2bp3+/- PNA.jpg]

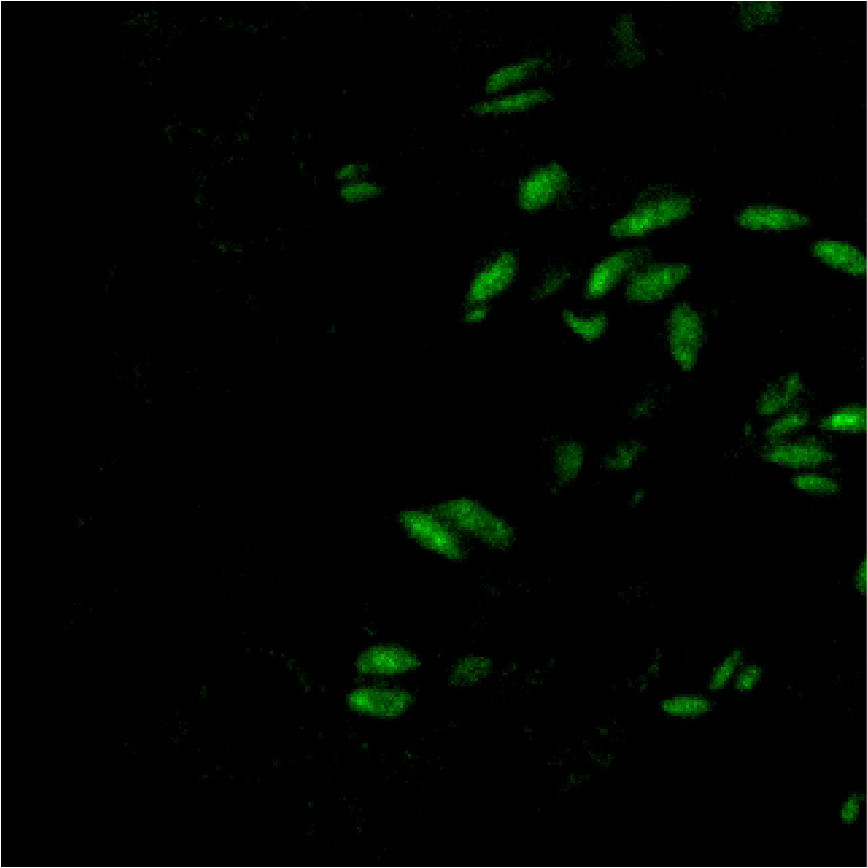

Supplement: Supplementary file 14 — Source data Fig. [file 44318_2025_659_MOESM14_ESM.zip › EMBOJ-2025-121587_Source Data/Source Data Figure 3/SD Figure 3G/Igf2bp3+/- PRM1.jpg]

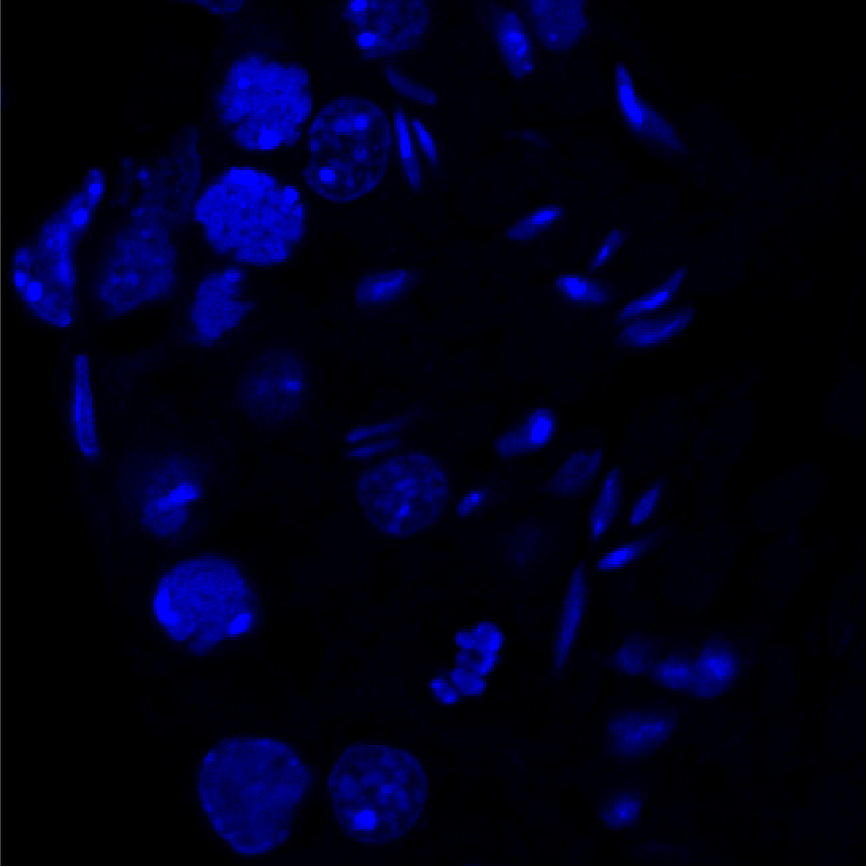

Supplement: Supplementary file 14 — Source data Fig. [file 44318_2025_659_MOESM14_ESM.zip › EMBOJ-2025-121587_Source Data/Source Data Figure 3/SD Figure 3G/Igf2bp3-/- Hoechst.jpg]

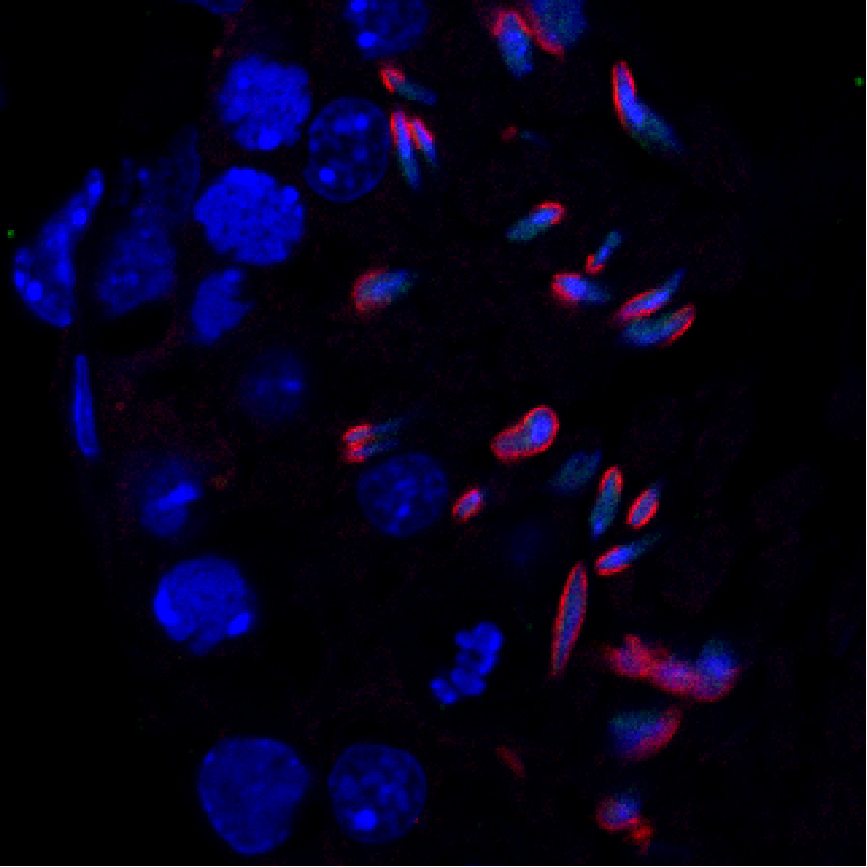

Supplement: Supplementary file 14 — Source data Fig. [file 44318_2025_659_MOESM14_ESM.zip › EMBOJ-2025-121587_Source Data/Source Data Figure 3/SD Figure 3G/Igf2bp3-/- Merge.jpg]

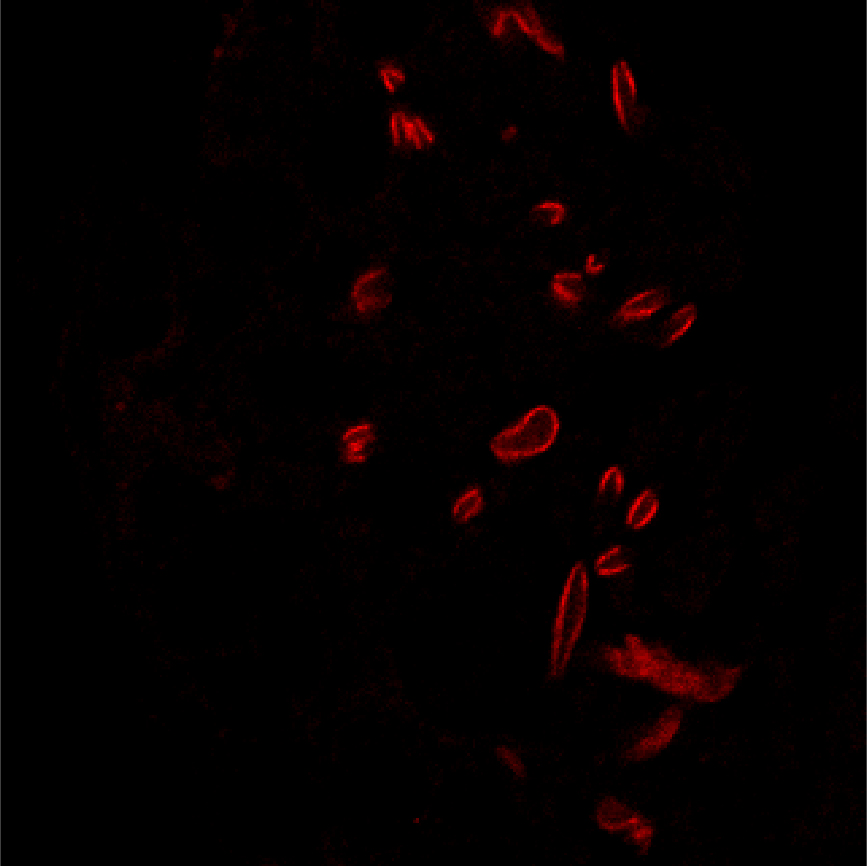

Supplement: Supplementary file 14 — Source data Fig. [file 44318_2025_659_MOESM14_ESM.zip › EMBOJ-2025-121587_Source Data/Source Data Figure 3/SD Figure 3G/Igf2bp3-/- PNA.jpg]

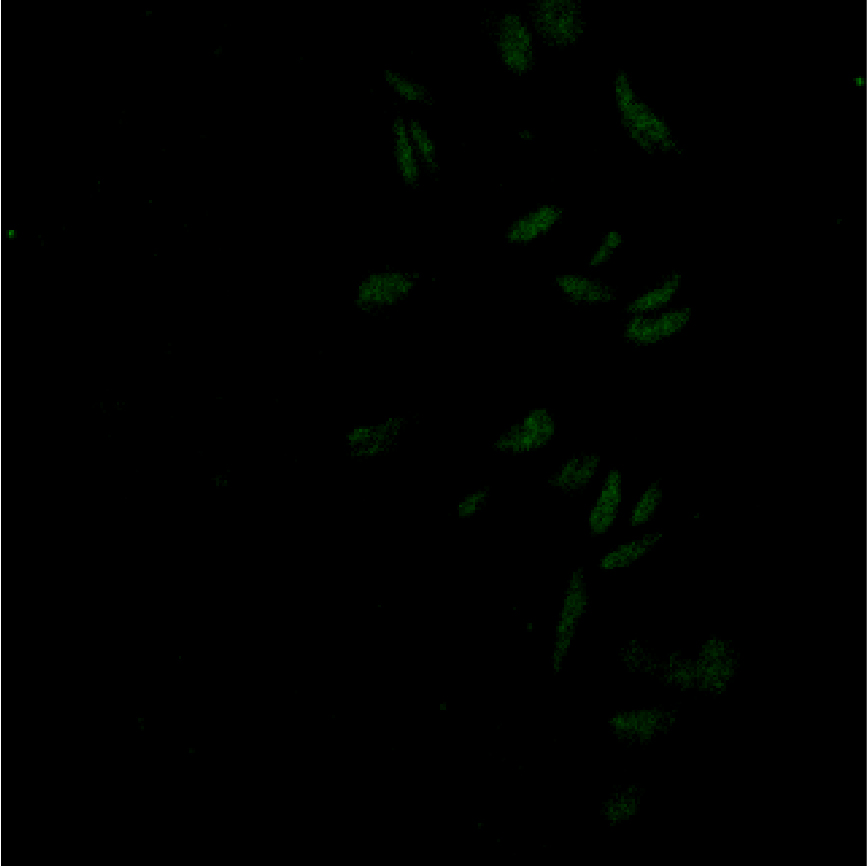

Supplement: Supplementary file 14 — Source data Fig. [file 44318_2025_659_MOESM14_ESM.zip › EMBOJ-2025-121587_Source Data/Source Data Figure 3/SD Figure 3G/Igf2bp3-/- PRM1.jpg]

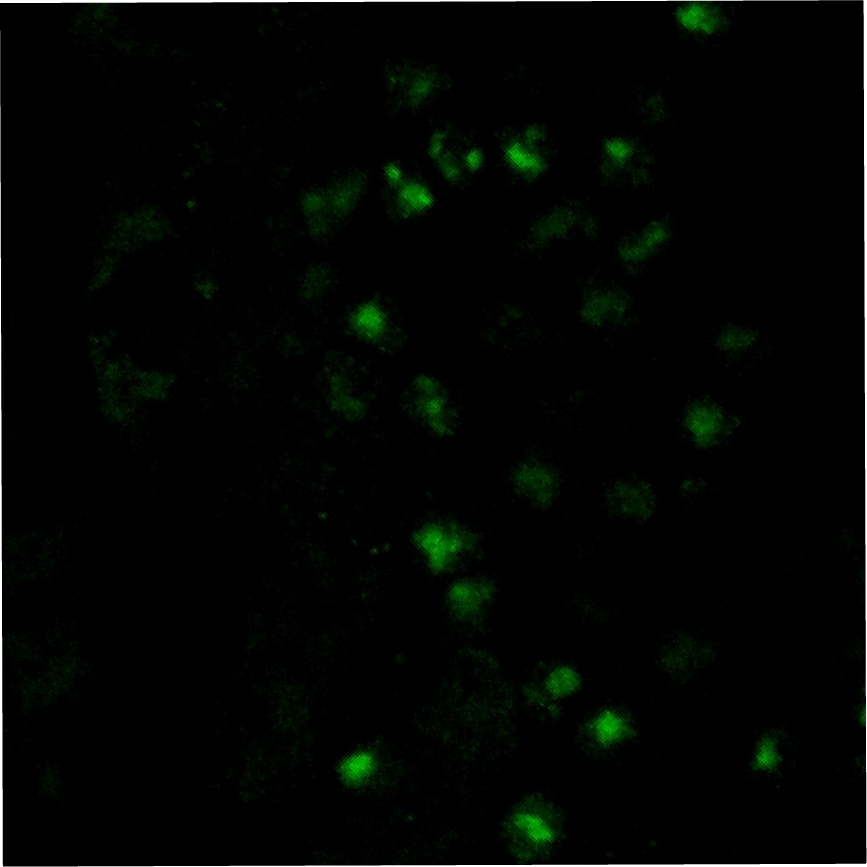

Supplement: Supplementary file 14 — Source data Fig. [file 44318_2025_659_MOESM14_ESM.zip › EMBOJ-2025-121587_Source Data/Source Data Figure 4/SD Figure 4I/SD Figure 4I Igf2bp3+/- Dot1l.jpg]

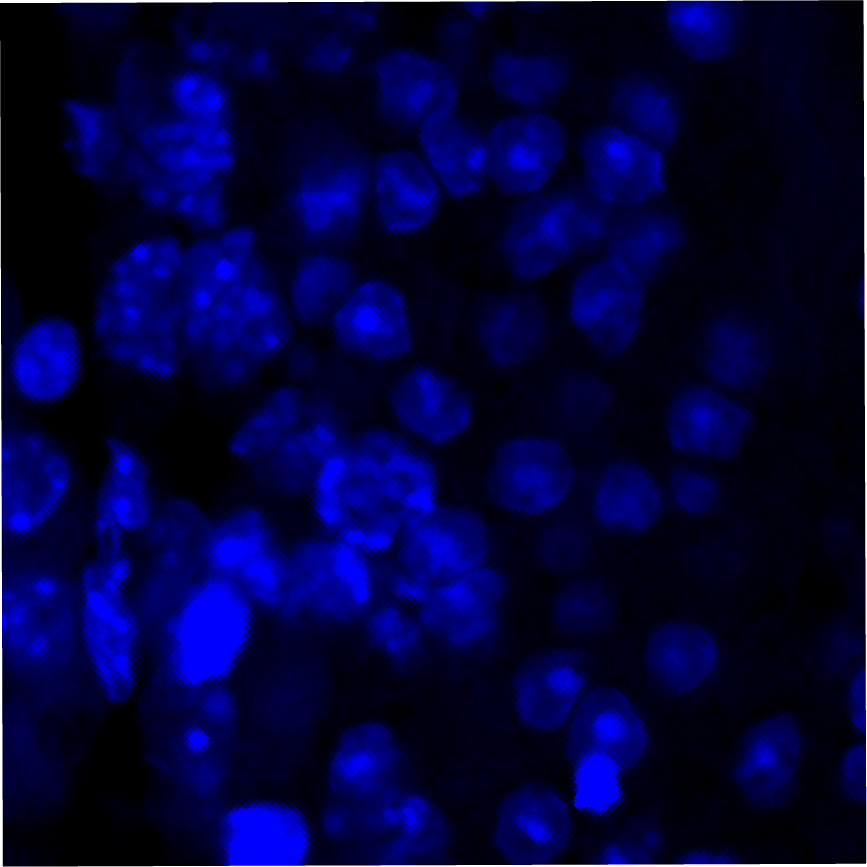

Supplement: Supplementary file 14 — Source data Fig. [file 44318_2025_659_MOESM14_ESM.zip › EMBOJ-2025-121587_Source Data/Source Data Figure 4/SD Figure 4I/SD Figure 4I Igf2bp3+/- Hoechst.jpg]

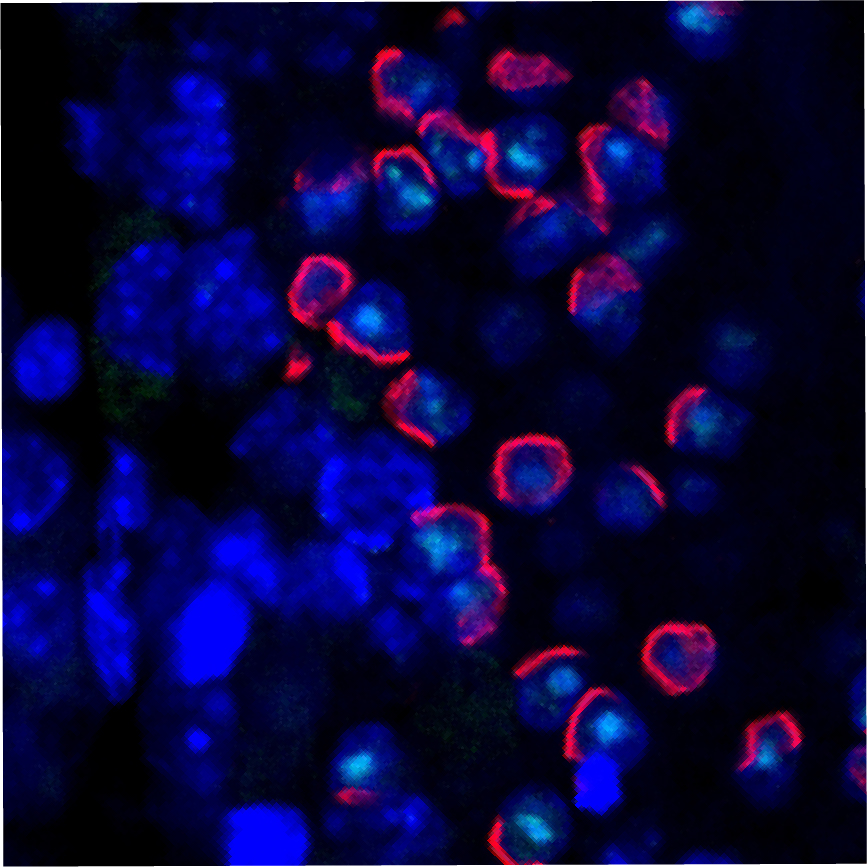

Supplement: Supplementary file 14 — Source data Fig. [file 44318_2025_659_MOESM14_ESM.zip › EMBOJ-2025-121587_Source Data/Source Data Figure 4/SD Figure 4I/SD Figure 4I Igf2bp3+/- Merge.jpg]

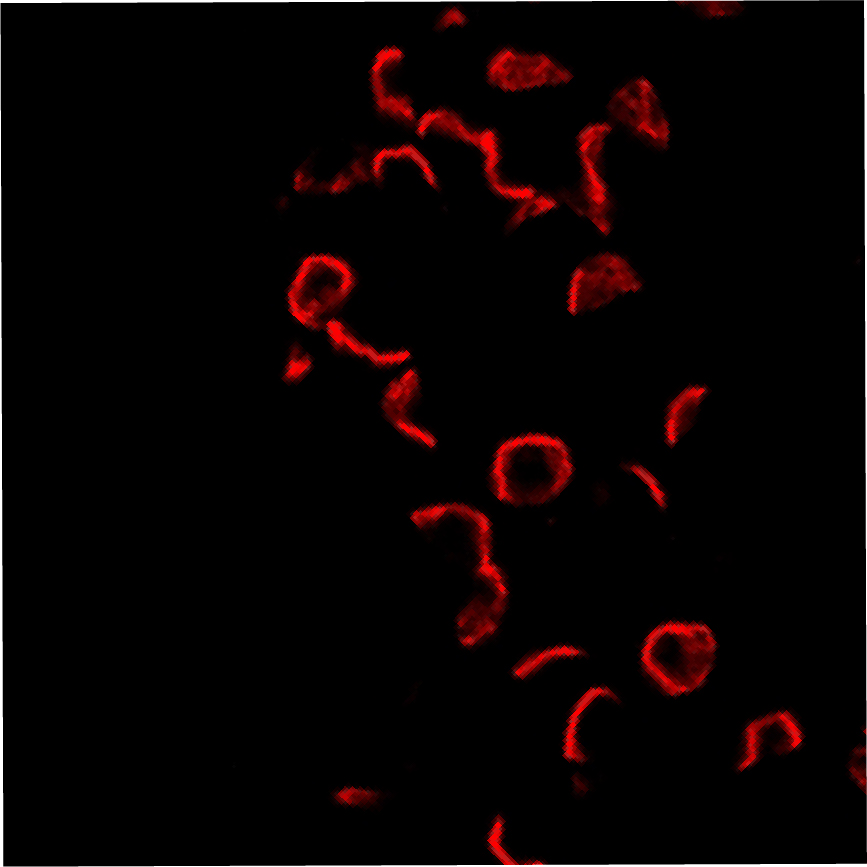

Supplement: Supplementary file 14 — Source data Fig. [file 44318_2025_659_MOESM14_ESM.zip › EMBOJ-2025-121587_Source Data/Source Data Figure 4/SD Figure 4I/SD Figure 4I Igf2bp3+/- PNA.jpg]

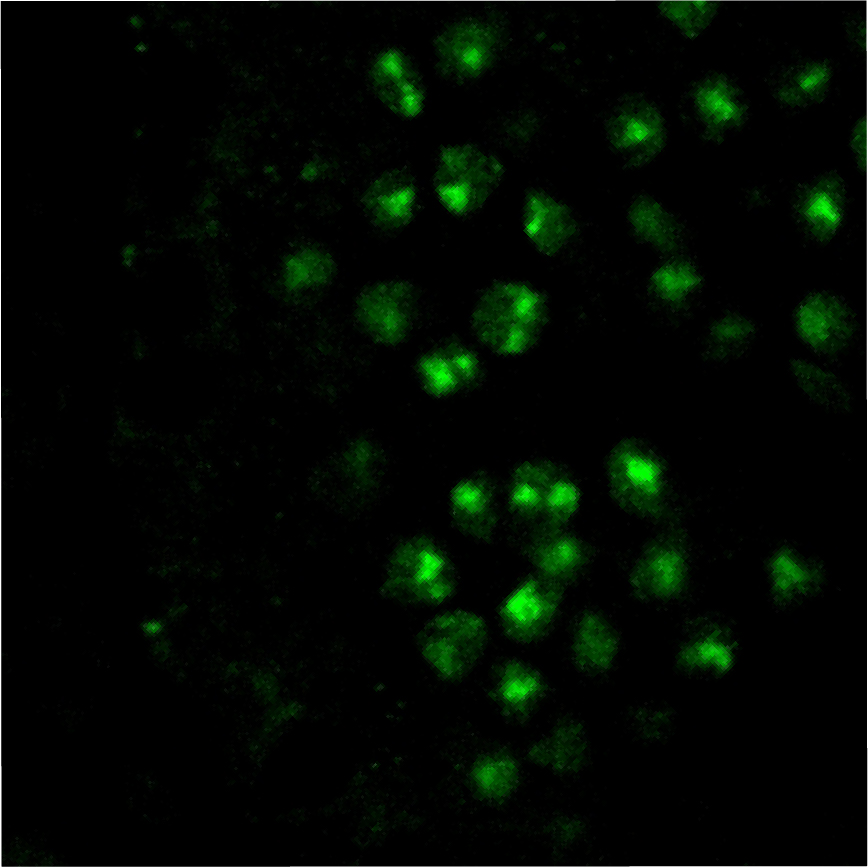

Supplement: Supplementary file 14 — Source data Fig. [file 44318_2025_659_MOESM14_ESM.zip › EMBOJ-2025-121587_Source Data/Source Data Figure 4/SD Figure 4I/SD Figure 4I Igf2bp3-/- Dot1l.jpg]

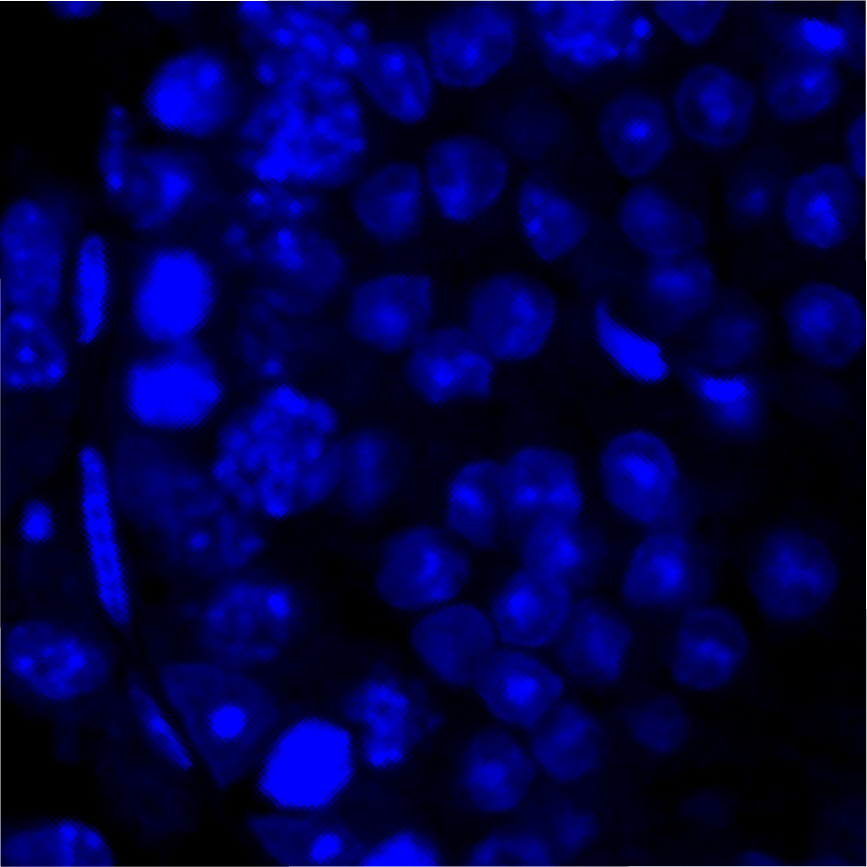

Supplement: Supplementary file 14 — Source data Fig. [file 44318_2025_659_MOESM14_ESM.zip › EMBOJ-2025-121587_Source Data/Source Data Figure 4/SD Figure 4I/SD Figure 4I Igf2bp3-/- Hoechst.jpg]

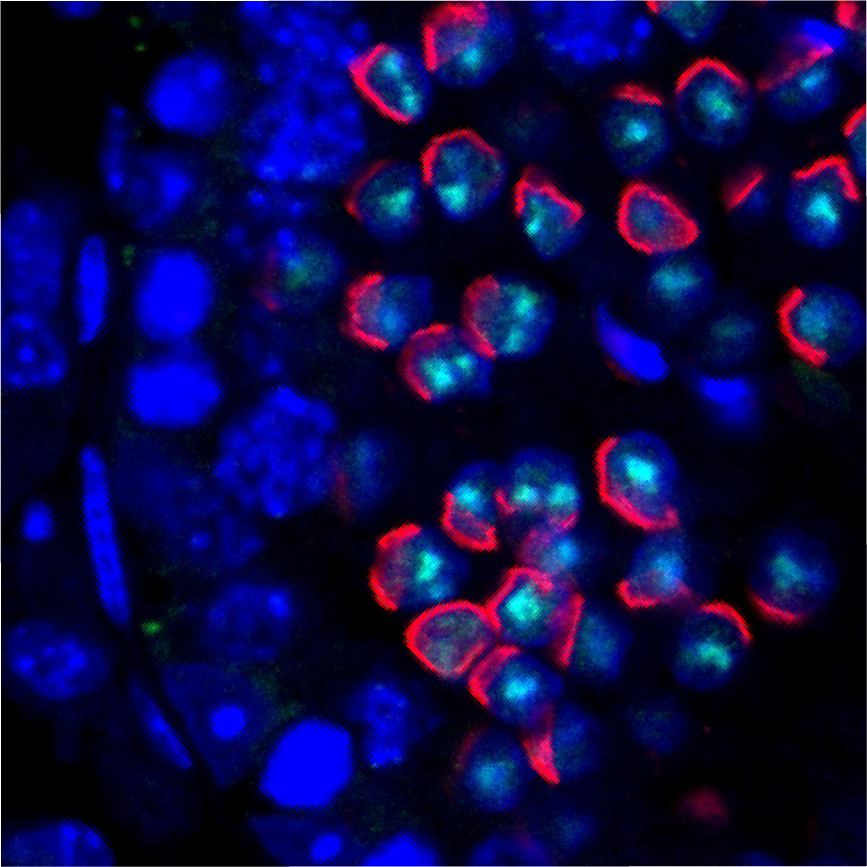

Supplement: Supplementary file 14 — Source data Fig. [file 44318_2025_659_MOESM14_ESM.zip › EMBOJ-2025-121587_Source Data/Source Data Figure 4/SD Figure 4I/SD Figure 4I Igf2bp3-/- Merge.jpg]

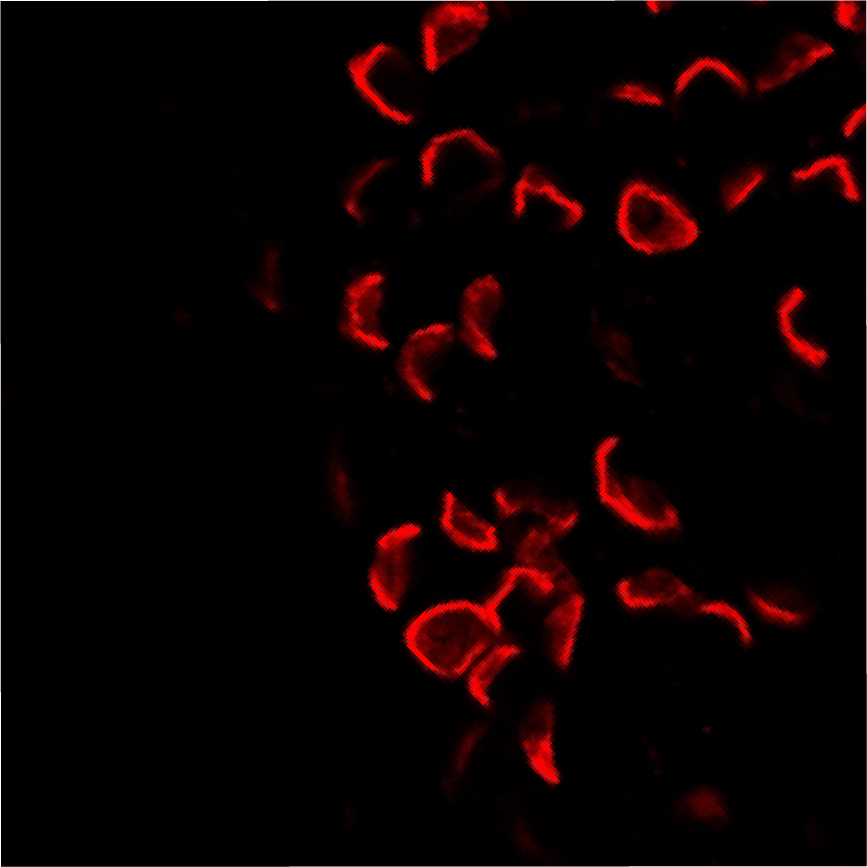

Supplement: Supplementary file 14 — Source data Fig. [file 44318_2025_659_MOESM14_ESM.zip › EMBOJ-2025-121587_Source Data/Source Data Figure 4/SD Figure 4I/SD Figure 4I Igf2bp3-/- PNA.jpg]

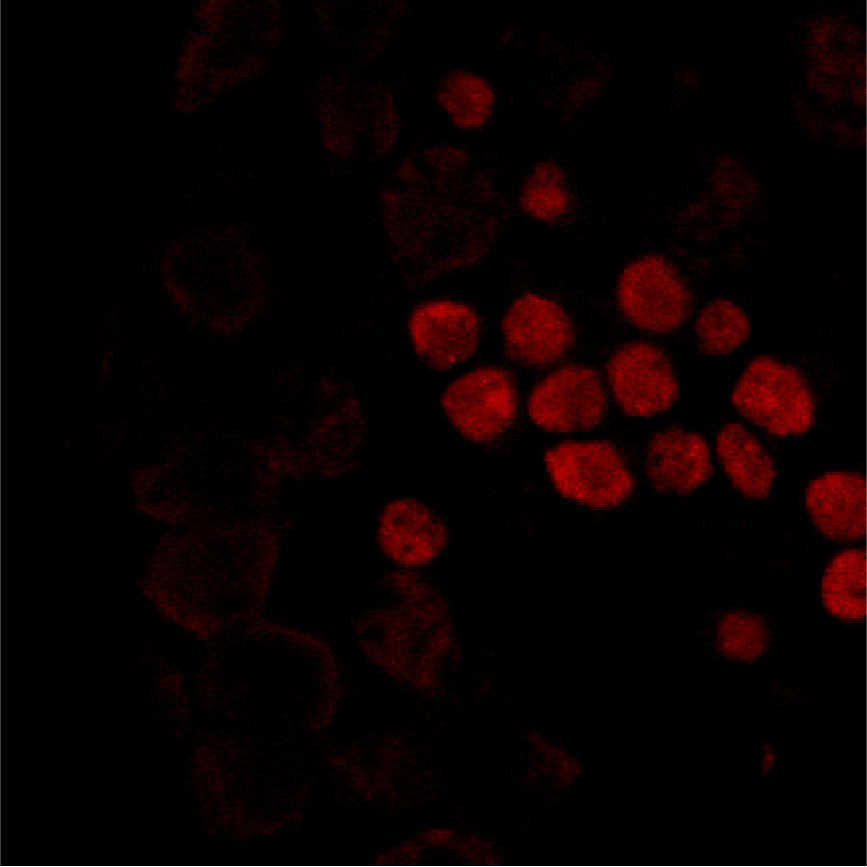

Supplement: Supplementary file 14 — Source data Fig. [file 44318_2025_659_MOESM14_ESM.zip › EMBOJ-2025-121587_Source Data/Source Data Figure 4/SD Figure 4J/Igf2bp3+/- H4Ac.jpg]

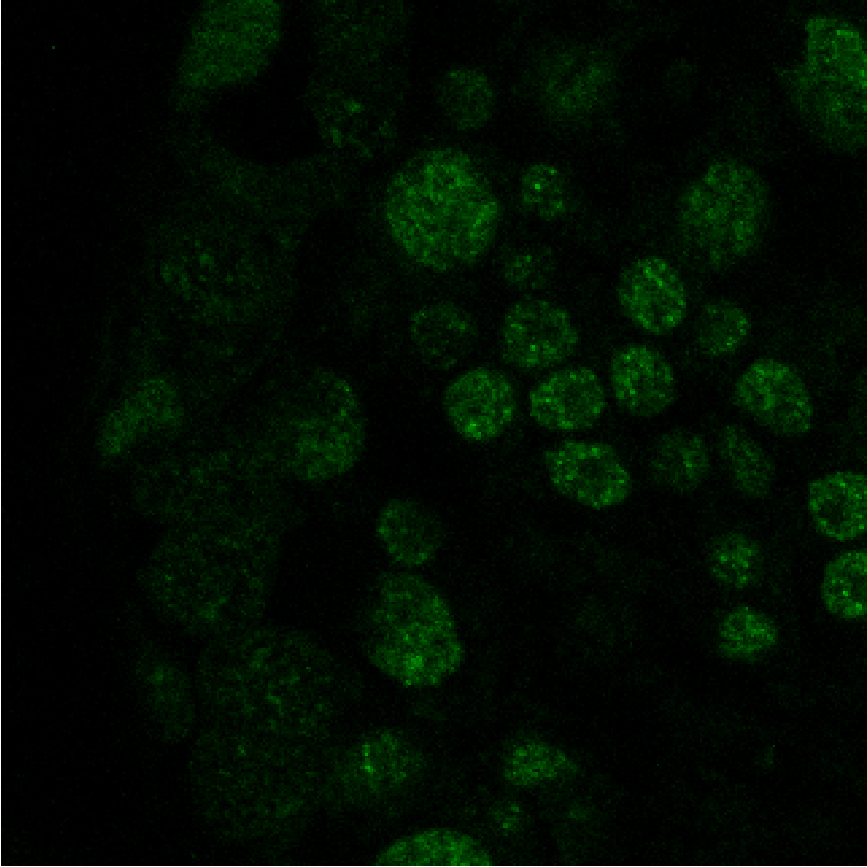

Supplement: Supplementary file 14 — Source data Fig. [file 44318_2025_659_MOESM14_ESM.zip › EMBOJ-2025-121587_Source Data/Source Data Figure 4/SD Figure 4J/Igf2bp3+/- Hdac11.jpg]

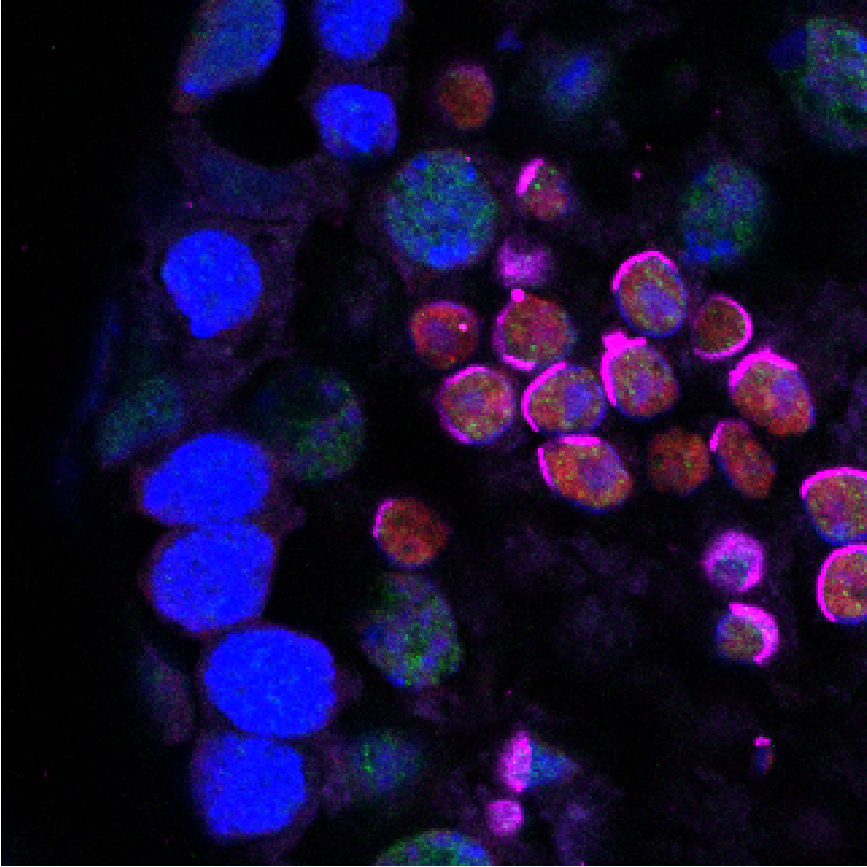

Supplement: Supplementary file 14 — Source data Fig. [file 44318_2025_659_MOESM14_ESM.zip › EMBOJ-2025-121587_Source Data/Source Data Figure 4/SD Figure 4J/Igf2bp3+/- Merge.jpg]

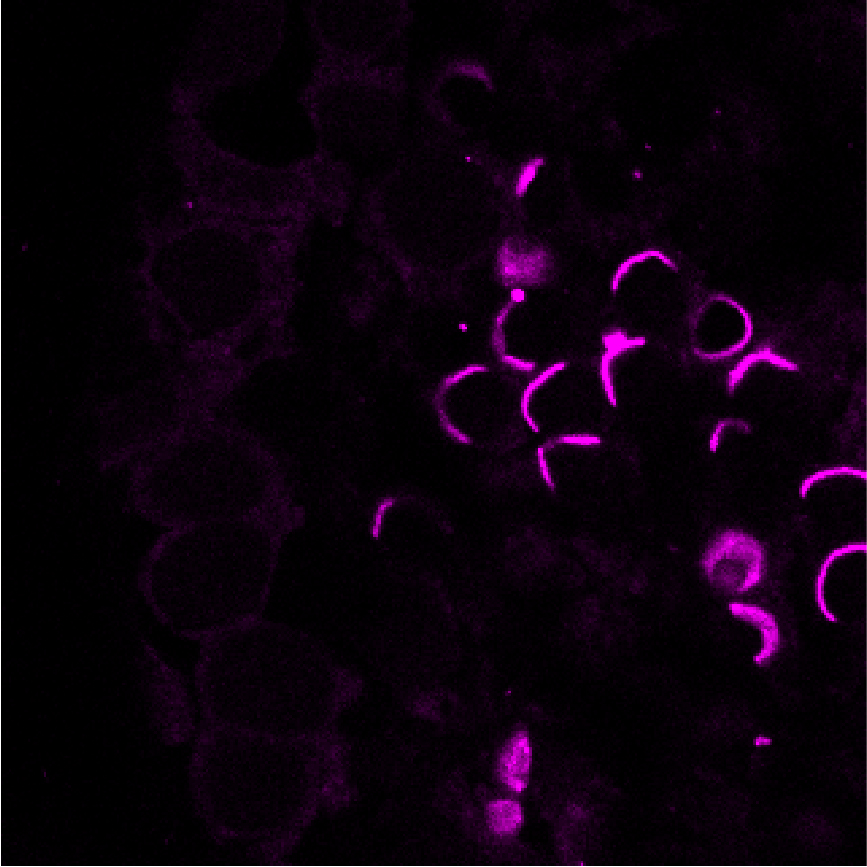

Supplement: Supplementary file 14 — Source data Fig. [file 44318_2025_659_MOESM14_ESM.zip › EMBOJ-2025-121587_Source Data/Source Data Figure 4/SD Figure 4J/Igf2bp3+/- PNA.jpg]

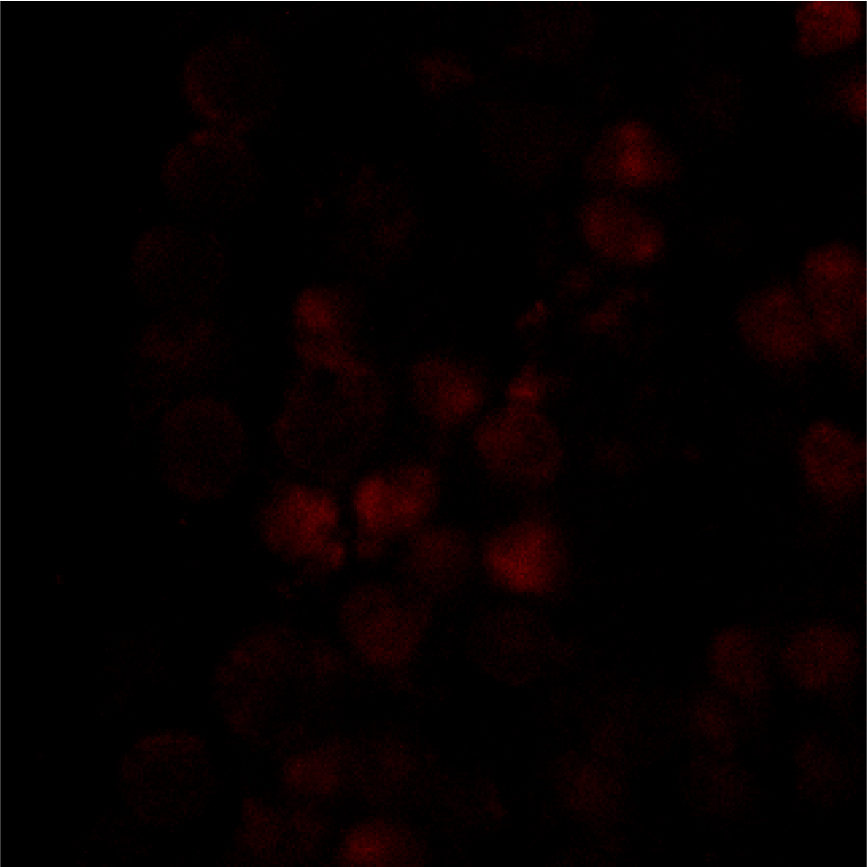

Supplement: Supplementary file 14 — Source data Fig. [file 44318_2025_659_MOESM14_ESM.zip › EMBOJ-2025-121587_Source Data/Source Data Figure 4/SD Figure 4J/Igf2bp3-/- H4Ac.jpg]

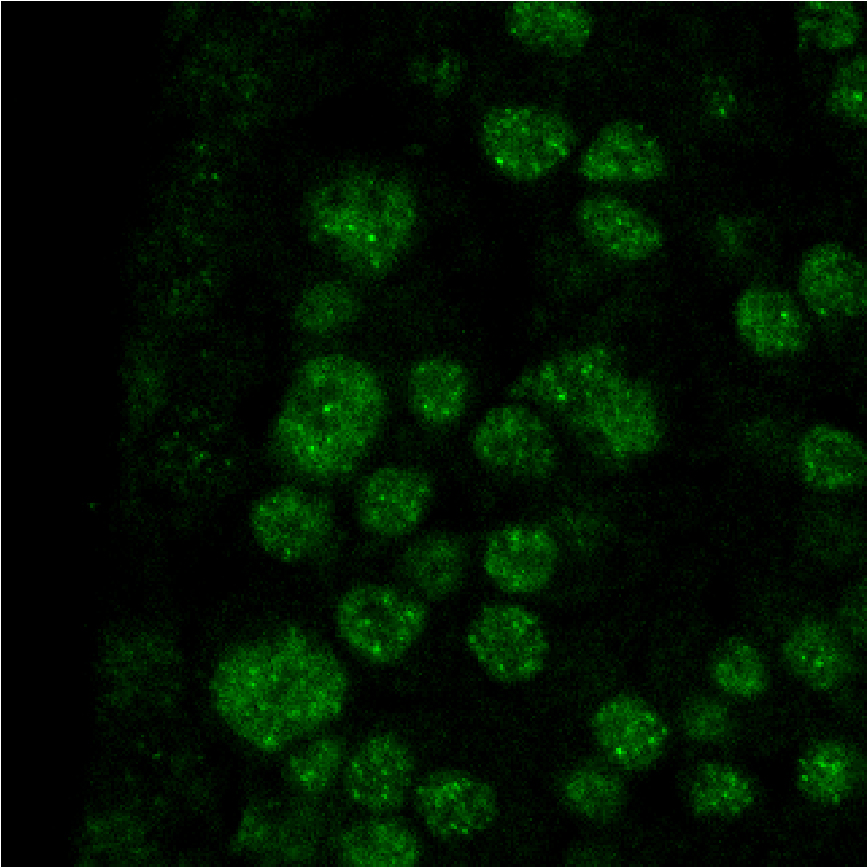

Supplement: Supplementary file 14 — Source data Fig. [file 44318_2025_659_MOESM14_ESM.zip › EMBOJ-2025-121587_Source Data/Source Data Figure 4/SD Figure 4J/Igf2bp3-/- Hdac11.jpg]

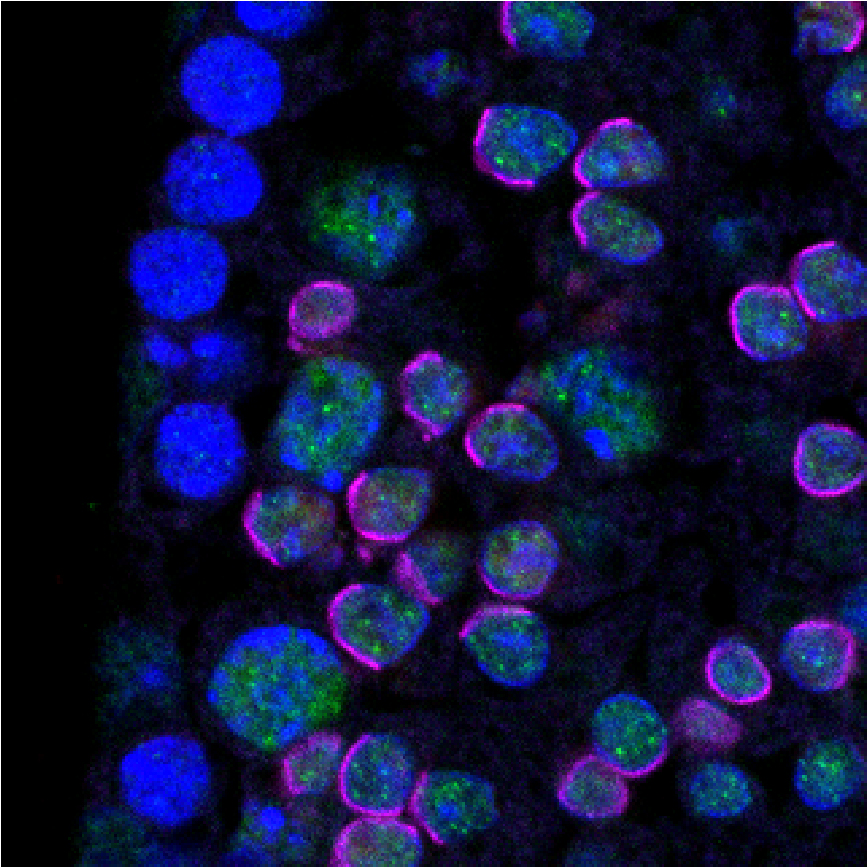

Supplement: Supplementary file 14 — Source data Fig. [file 44318_2025_659_MOESM14_ESM.zip › EMBOJ-2025-121587_Source Data/Source Data Figure 4/SD Figure 4J/Igf2bp3-/- Merge.jpg]

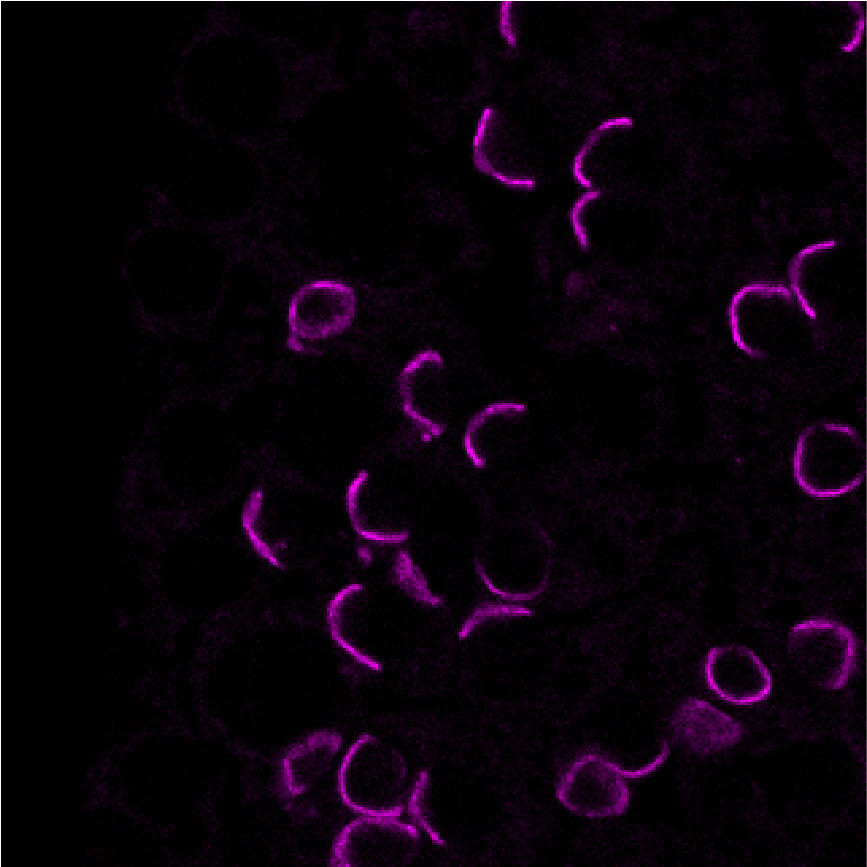

Supplement: Supplementary file 14 — Source data Fig. [file 44318_2025_659_MOESM14_ESM.zip › EMBOJ-2025-121587_Source Data/Source Data Figure 4/SD Figure 4J/Igf2bp3-/- PNA.jpg]

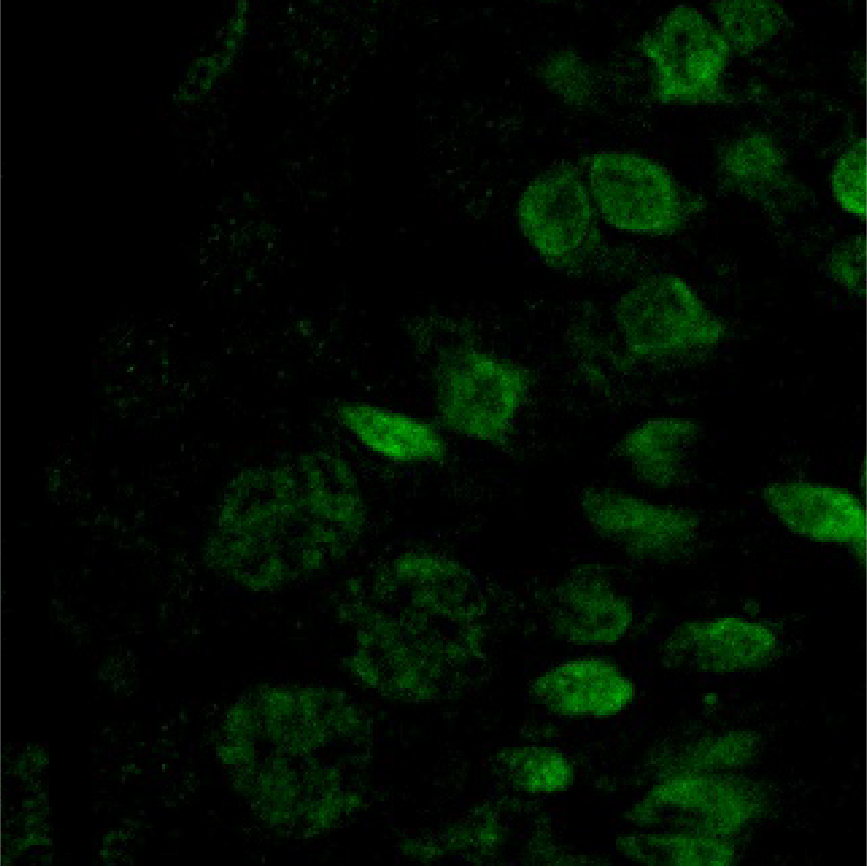

Supplement: Supplementary file 14 — Source data Fig. [file 44318_2025_659_MOESM14_ESM.zip › EMBOJ-2025-121587_Source Data/Source Data Figure 4/SD Figure 4K/Igf2bp3+/- H3K79me2.jpg]

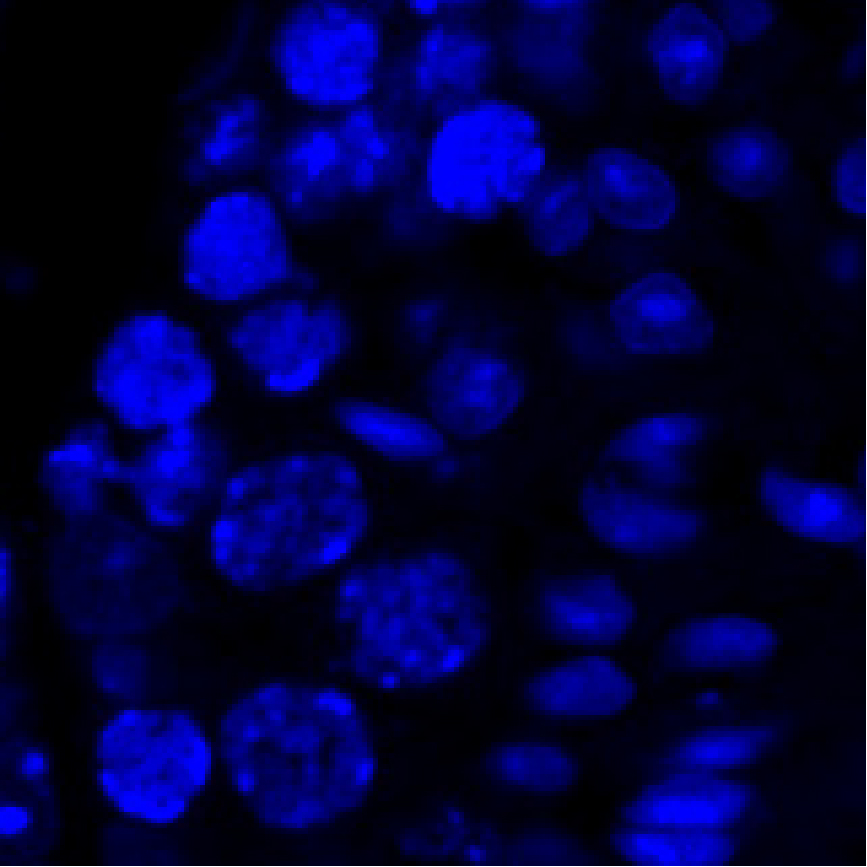

Supplement: Supplementary file 14 — Source data Fig. [file 44318_2025_659_MOESM14_ESM.zip › EMBOJ-2025-121587_Source Data/Source Data Figure 4/SD Figure 4K/Igf2bp3+/- Hoechst.jpg]

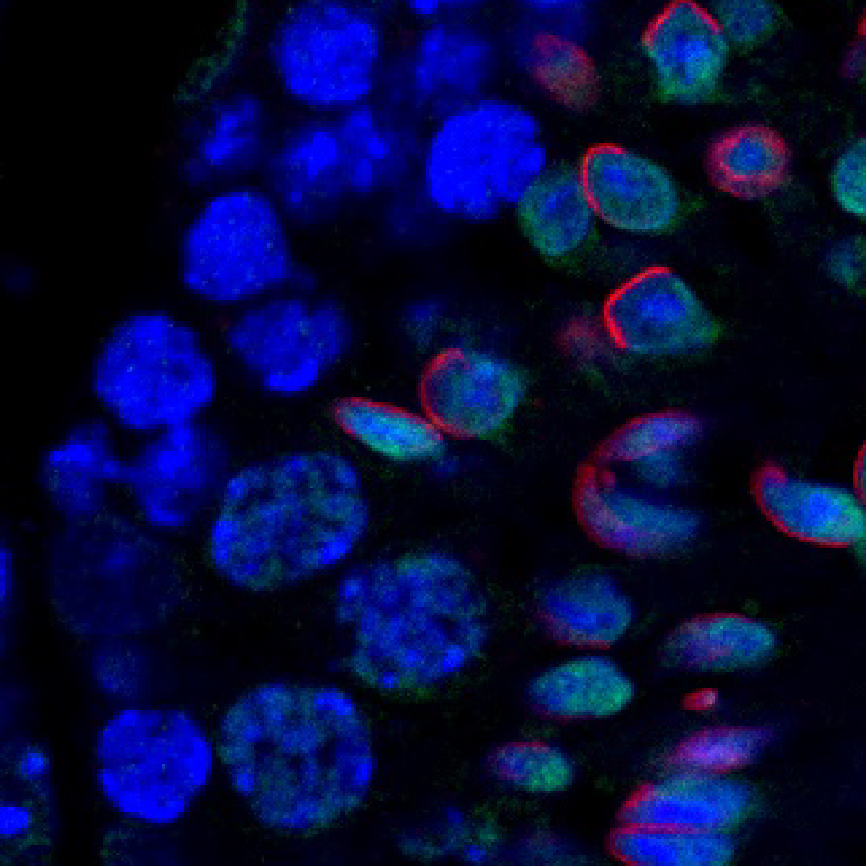

Supplement: Supplementary file 14 — Source data Fig. [file 44318_2025_659_MOESM14_ESM.zip › EMBOJ-2025-121587_Source Data/Source Data Figure 4/SD Figure 4K/Igf2bp3+/- Merge.jpg]

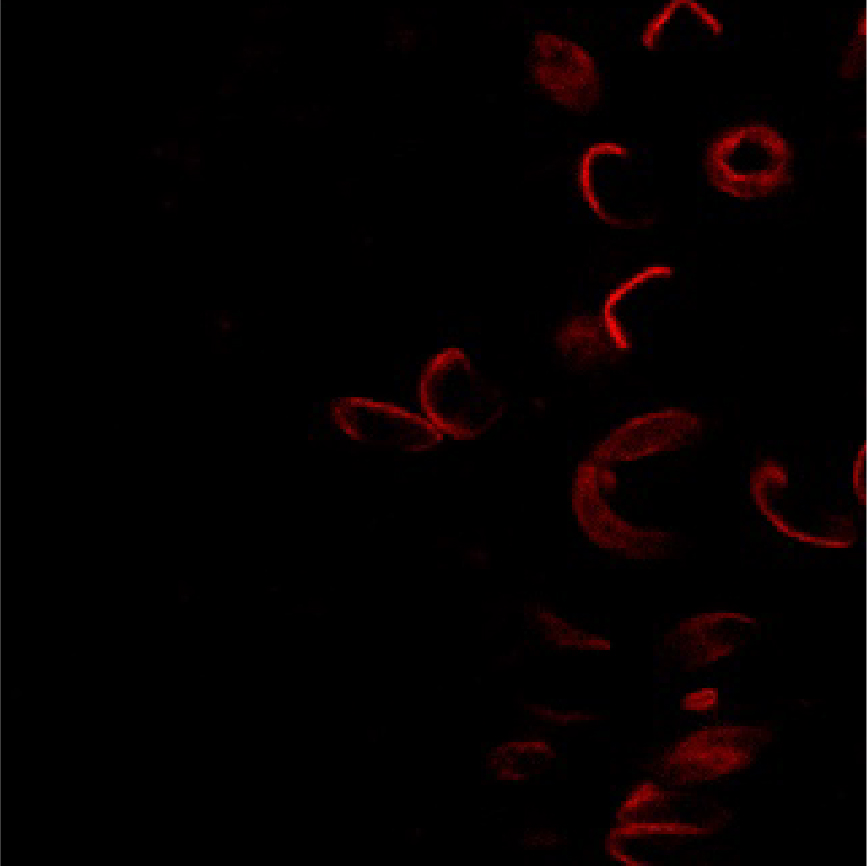

Supplement: Supplementary file 14 — Source data Fig. [file 44318_2025_659_MOESM14_ESM.zip › EMBOJ-2025-121587_Source Data/Source Data Figure 4/SD Figure 4K/Igf2bp3+/- PNA.jpg]

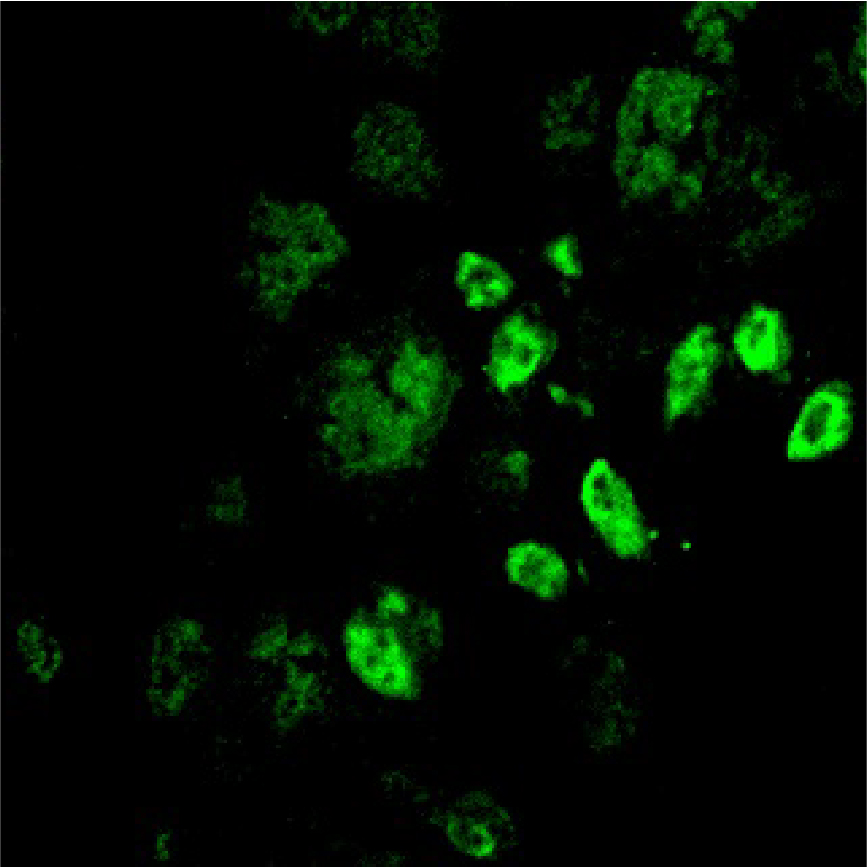

Supplement: Supplementary file 14 — Source data Fig. [file 44318_2025_659_MOESM14_ESM.zip › EMBOJ-2025-121587_Source Data/Source Data Figure 4/SD Figure 4K/Igf2bp3-/- H3K79me2.jpg]

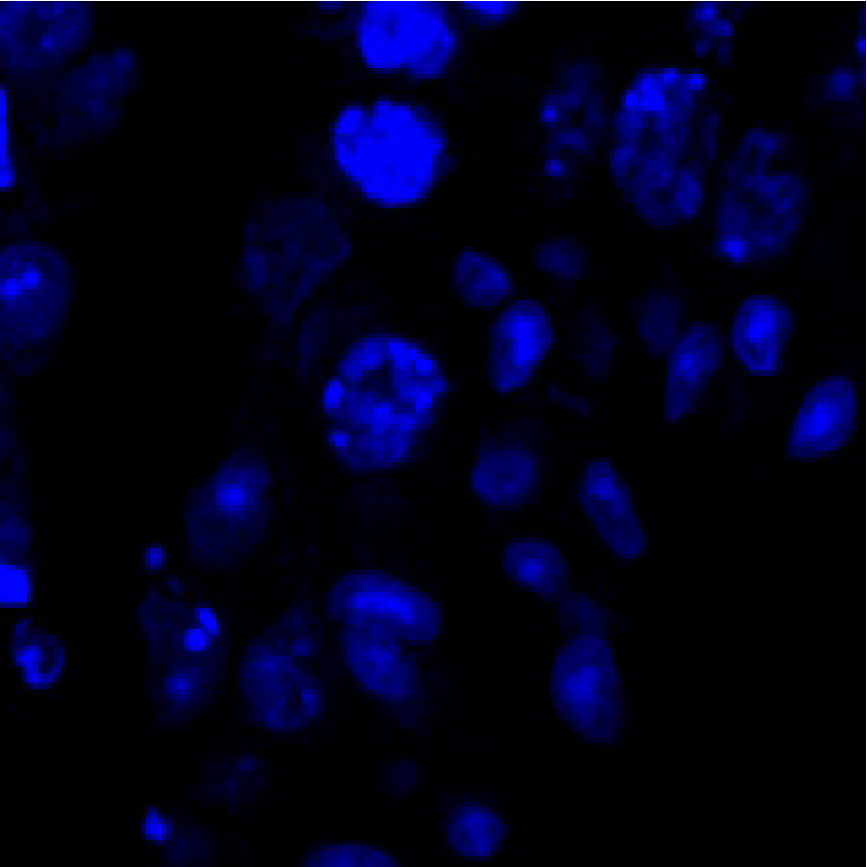

Supplement: Supplementary file 14 — Source data Fig. [file 44318_2025_659_MOESM14_ESM.zip › EMBOJ-2025-121587_Source Data/Source Data Figure 4/SD Figure 4K/Igf2bp3-/- Hoechst.jpg]

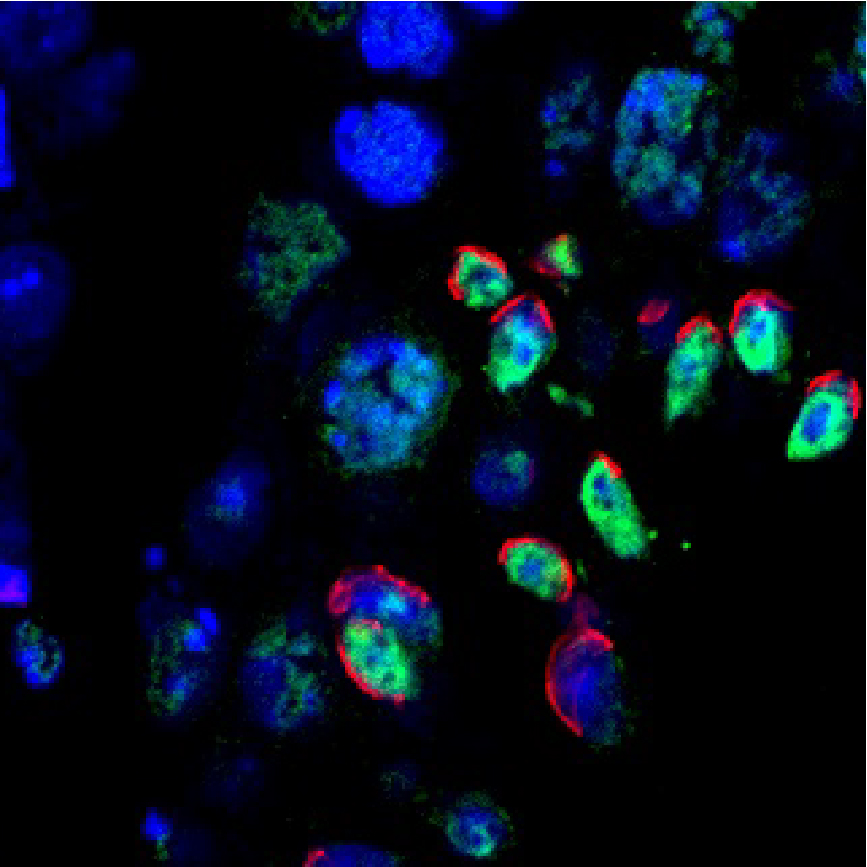

Supplement: Supplementary file 14 — Source data Fig. [file 44318_2025_659_MOESM14_ESM.zip › EMBOJ-2025-121587_Source Data/Source Data Figure 4/SD Figure 4K/Igf2bp3-/- Merge.jpg]

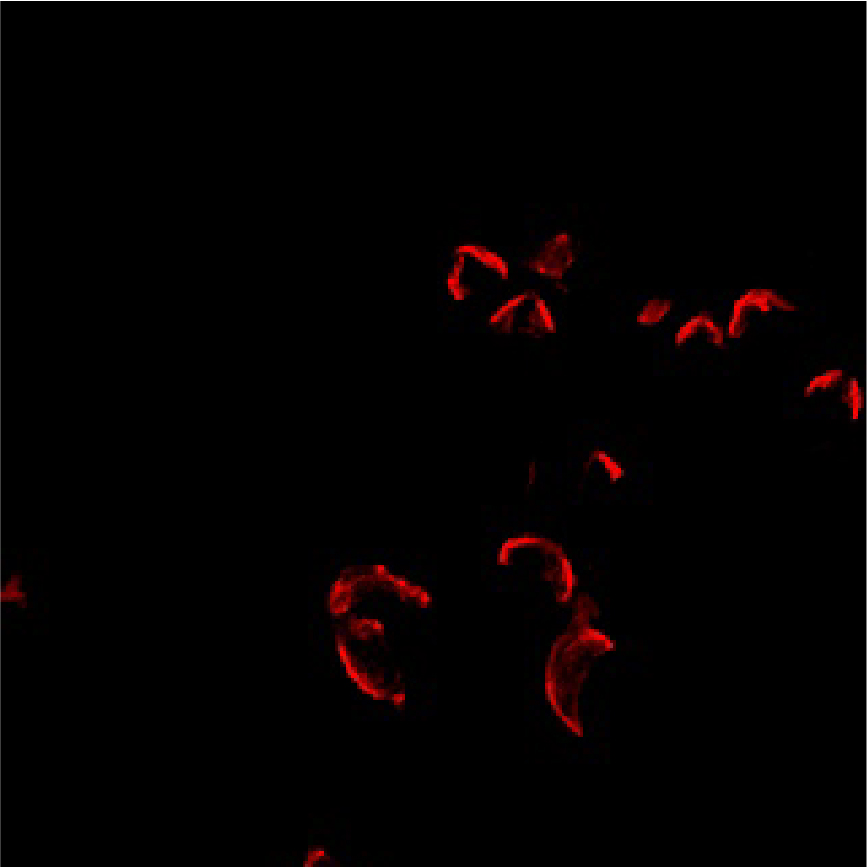

Supplement: Supplementary file 14 — Source data Fig. [file 44318_2025_659_MOESM14_ESM.zip › EMBOJ-2025-121587_Source Data/Source Data Figure 4/SD Figure 4K/Igf2bp3-/- PNA.jpg]

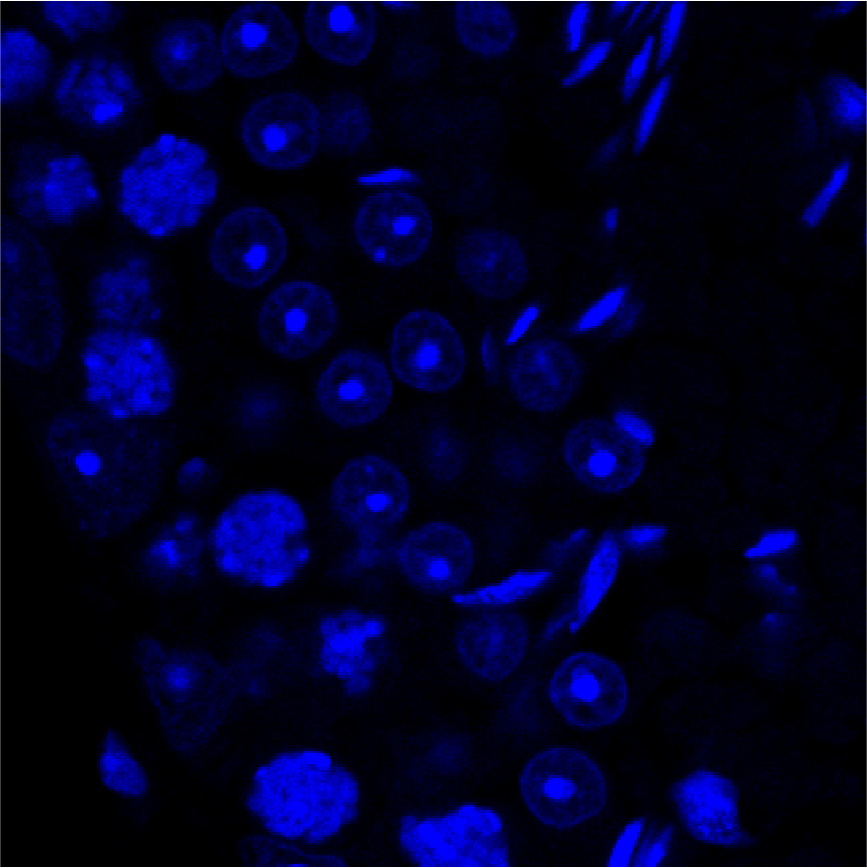

Supplement: Supplementary file 14 — Source data Fig. [file 44318_2025_659_MOESM14_ESM.zip › EMBOJ-2025-121587_Source Data/Source Data Figure 5/SD Figure 5B/Stage I-II Hoechst.jpg]

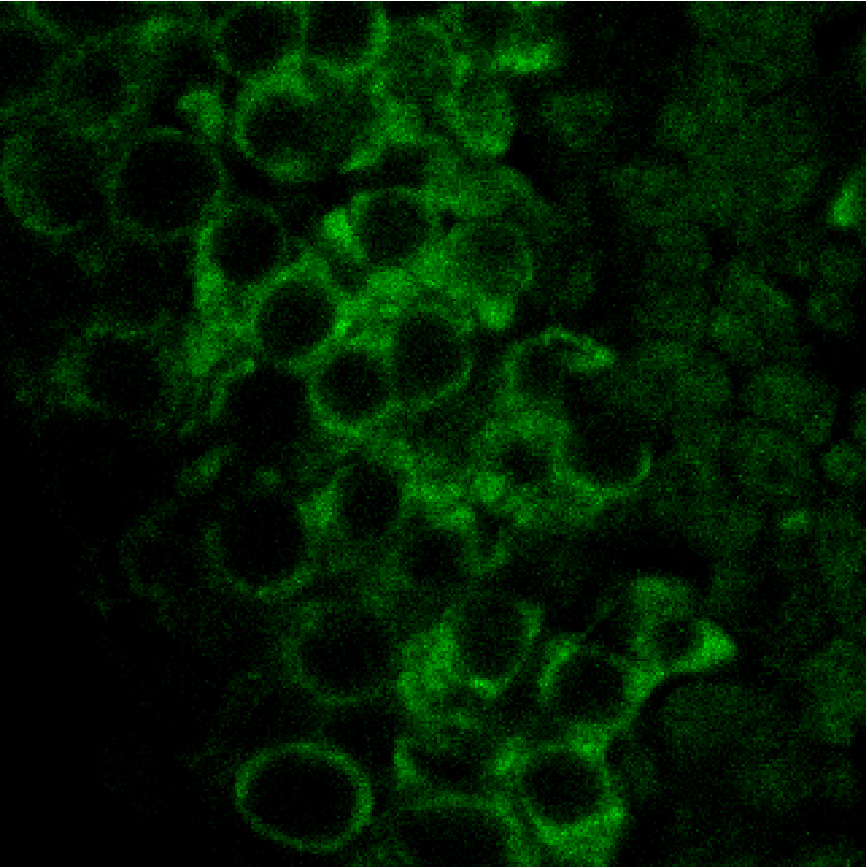

Supplement: Supplementary file 14 — Source data Fig. [file 44318_2025_659_MOESM14_ESM.zip › EMBOJ-2025-121587_Source Data/Source Data Figure 5/SD Figure 5B/Stage I-II IGF2BP3.jpg]

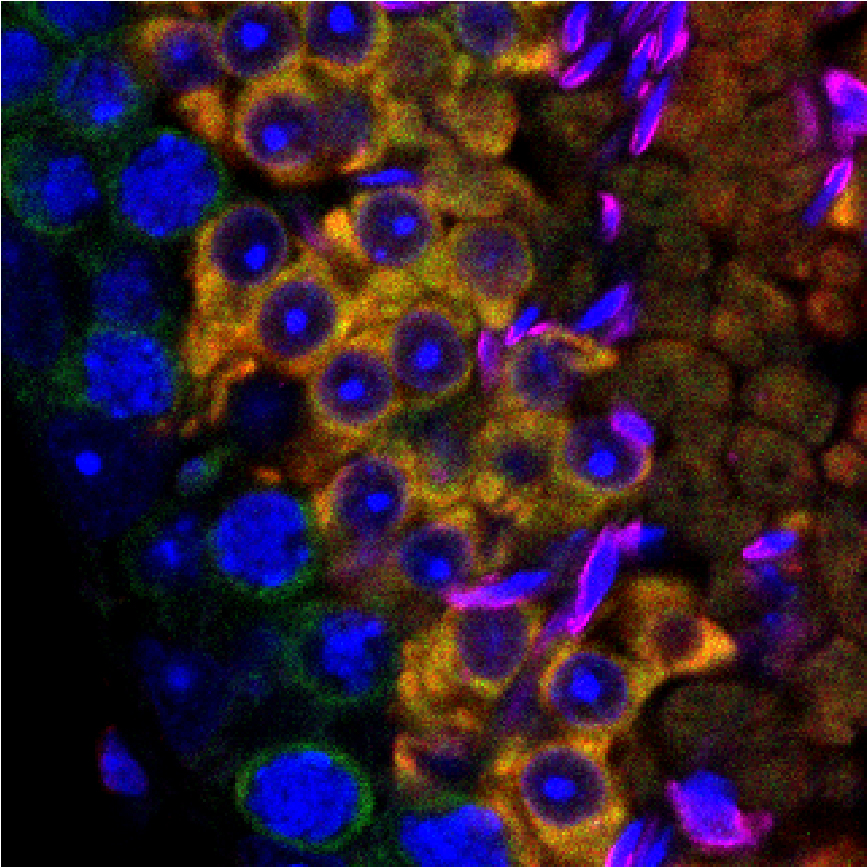

Supplement: Supplementary file 14 — Source data Fig. [file 44318_2025_659_MOESM14_ESM.zip › EMBOJ-2025-121587_Source Data/Source Data Figure 5/SD Figure 5B/Stage I-II Merge.jpg]

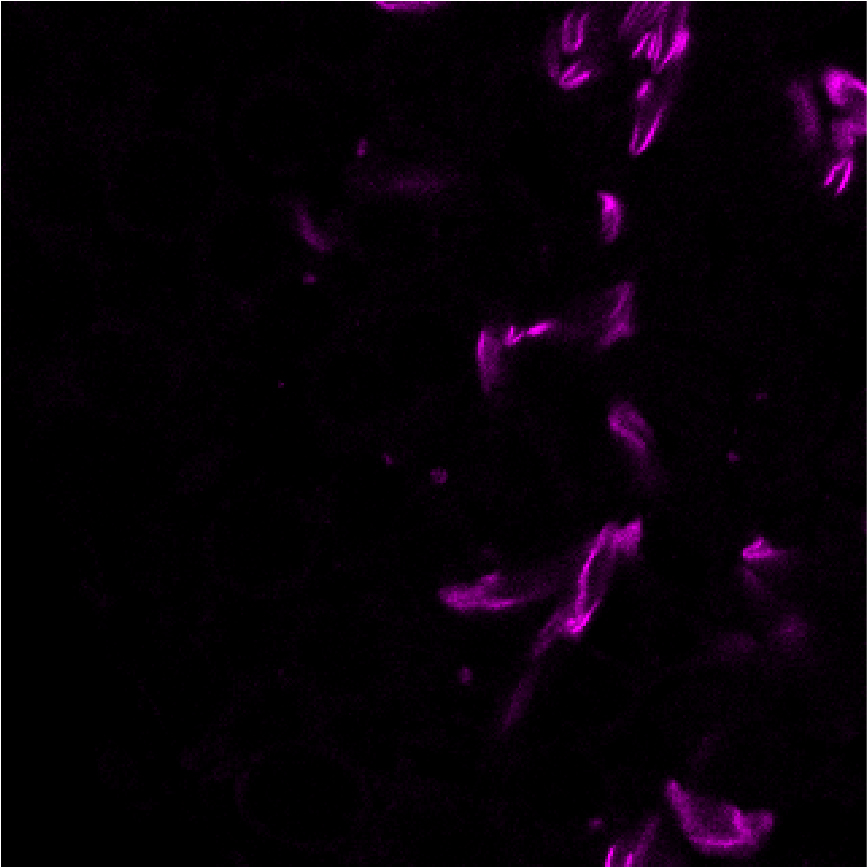

Supplement: Supplementary file 14 — Source data Fig. [file 44318_2025_659_MOESM14_ESM.zip › EMBOJ-2025-121587_Source Data/Source Data Figure 5/SD Figure 5B/Stage I-II PNA.jpg]

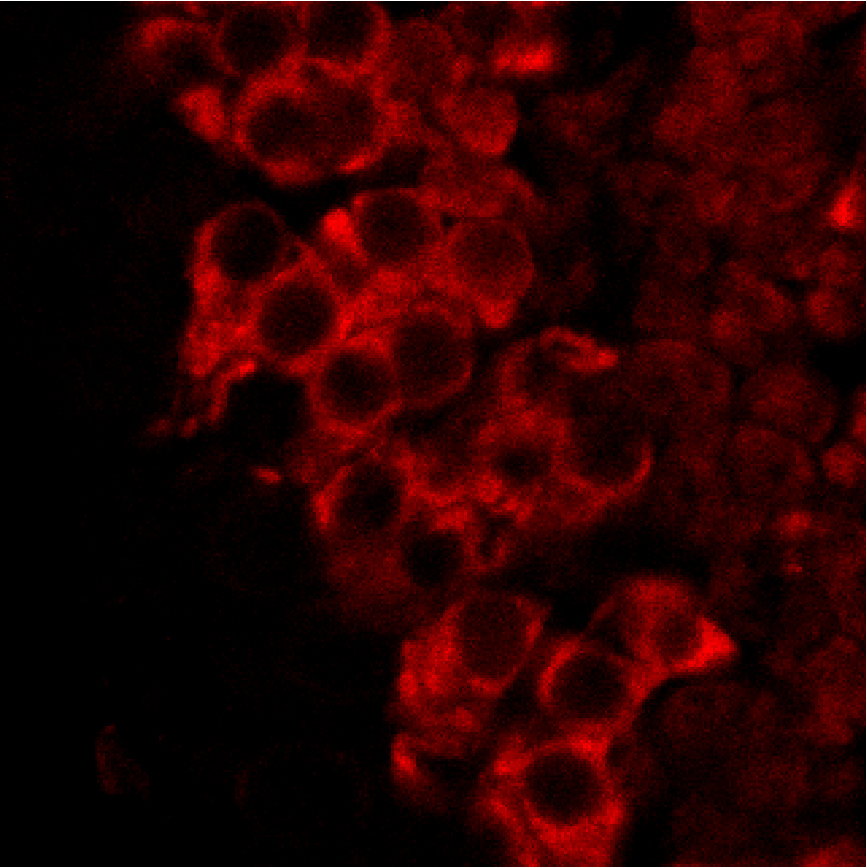

Supplement: Supplementary file 14 — Source data Fig. [file 44318_2025_659_MOESM14_ESM.zip › EMBOJ-2025-121587_Source Data/Source Data Figure 5/SD Figure 5B/Stage I-II YBX2.jpg]

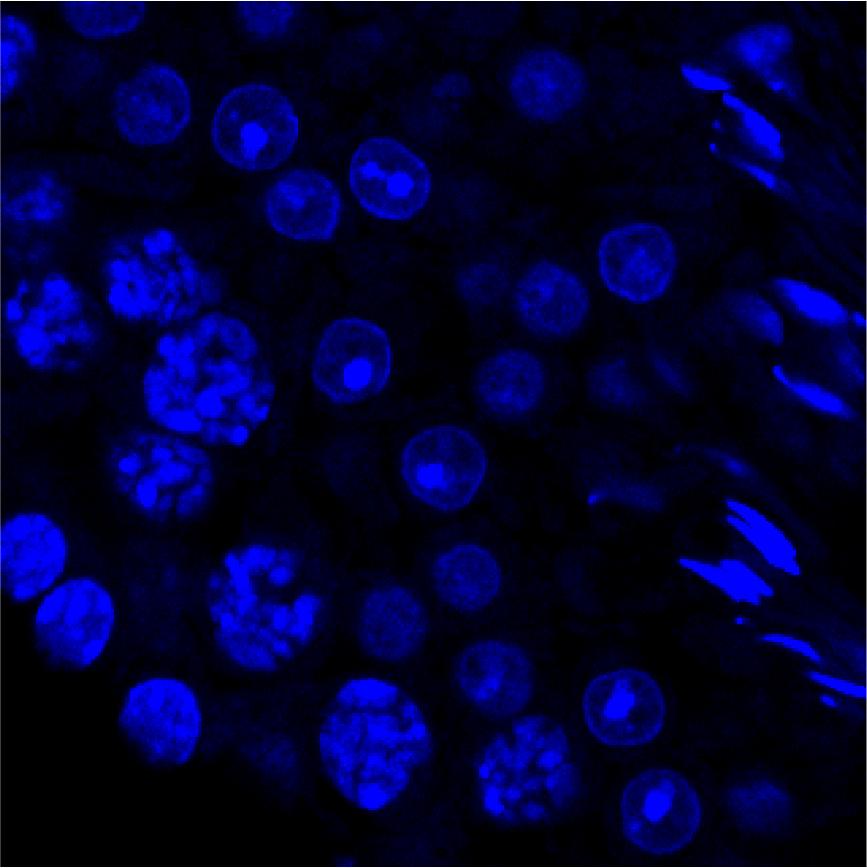

Supplement: Supplementary file 14 — Source data Fig. [file 44318_2025_659_MOESM14_ESM.zip › EMBOJ-2025-121587_Source Data/Source Data Figure 5/SD Figure 5B/Stage VI-VII Hoechst.jpg]

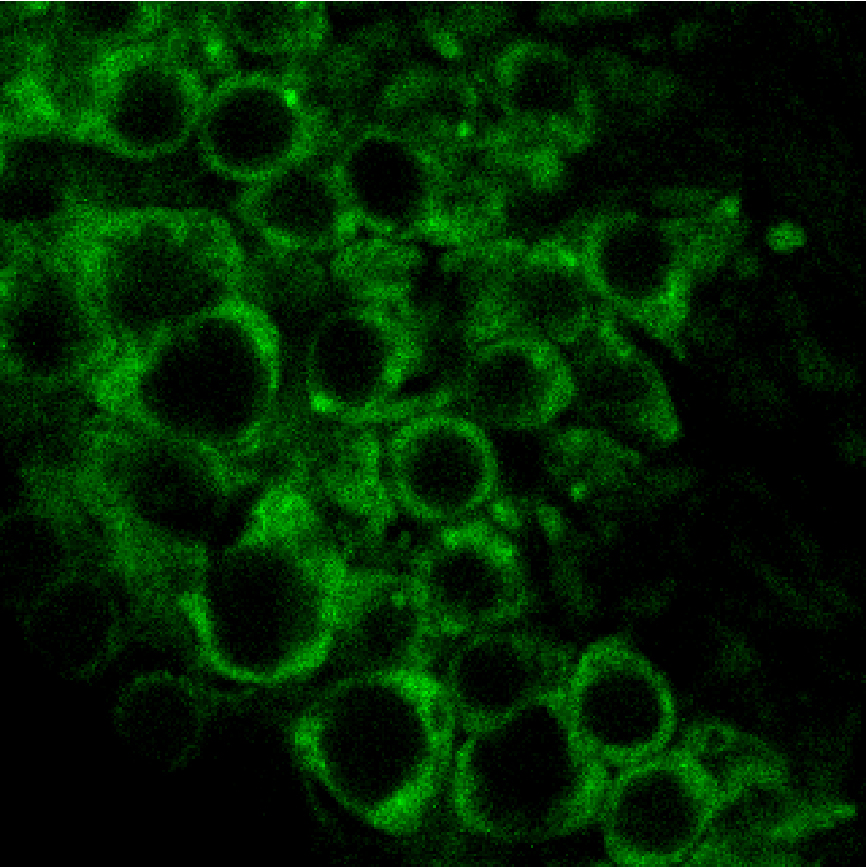

Supplement: Supplementary file 14 — Source data Fig. [file 44318_2025_659_MOESM14_ESM.zip › EMBOJ-2025-121587_Source Data/Source Data Figure 5/SD Figure 5B/Stage VI-VII IGF2BP3.jpg]

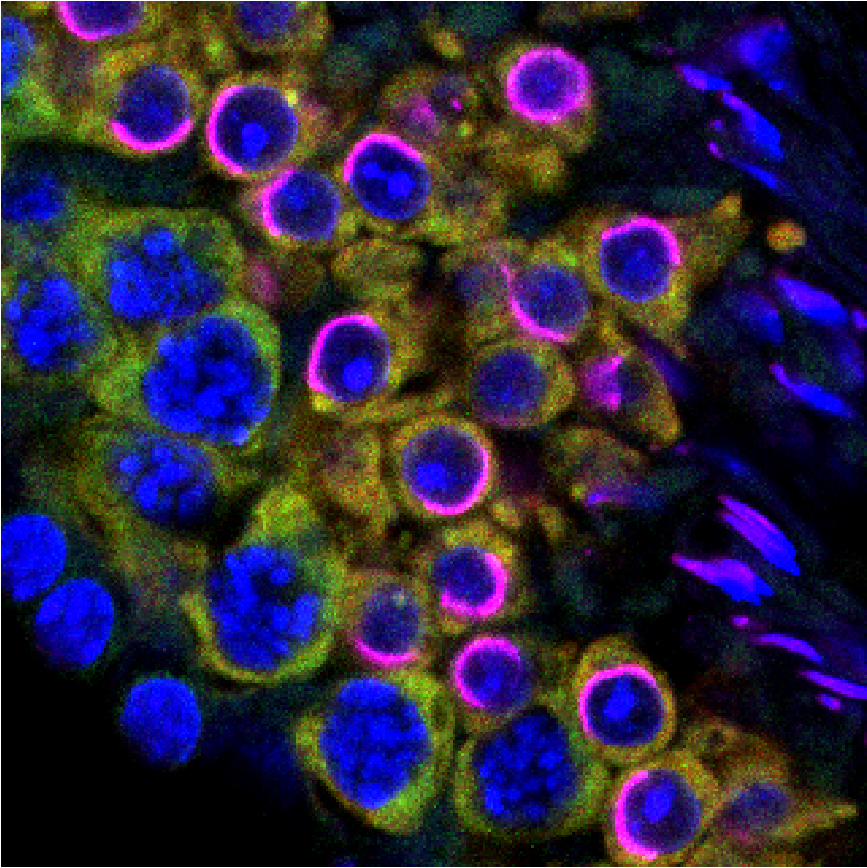

Supplement: Supplementary file 14 — Source data Fig. [file 44318_2025_659_MOESM14_ESM.zip › EMBOJ-2025-121587_Source Data/Source Data Figure 5/SD Figure 5B/Stage VI-VII Merge.jpg]

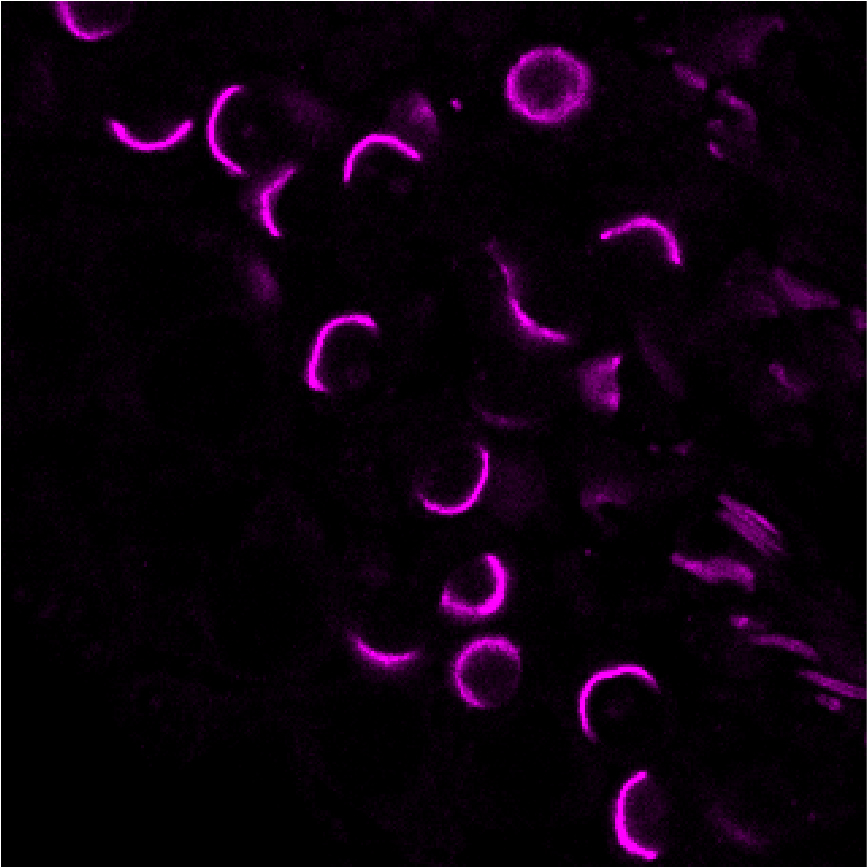

Supplement: Supplementary file 14 — Source data Fig. [file 44318_2025_659_MOESM14_ESM.zip › EMBOJ-2025-121587_Source Data/Source Data Figure 5/SD Figure 5B/Stage VI-VII PNA.jpg]

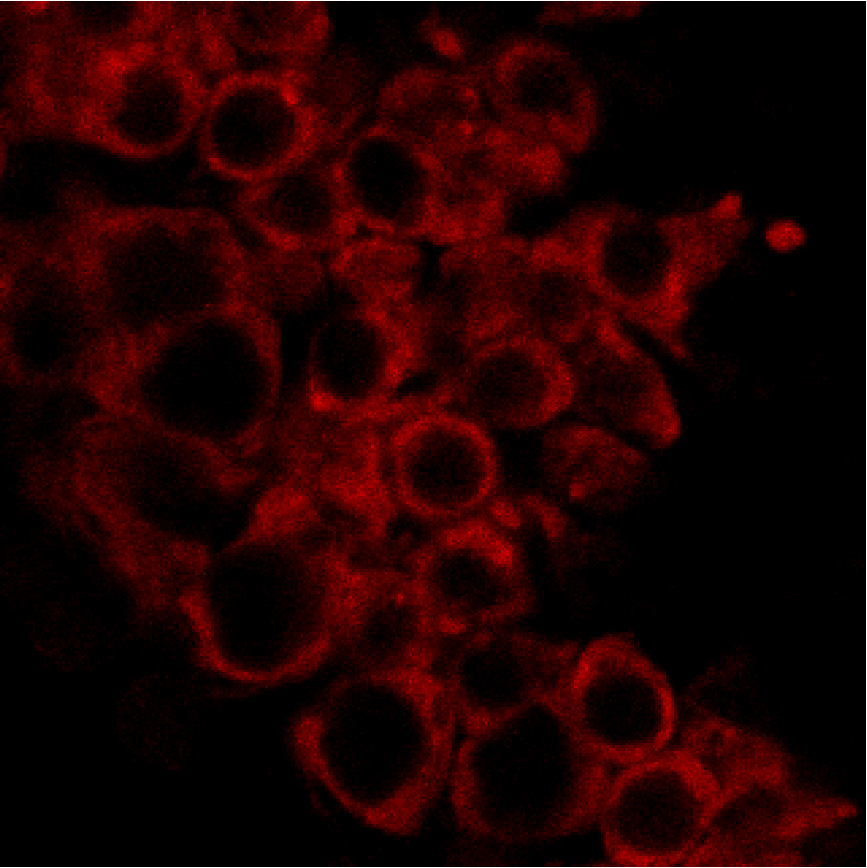

Supplement: Supplementary file 14 — Source data Fig. [file 44318_2025_659_MOESM14_ESM.zip › EMBOJ-2025-121587_Source Data/Source Data Figure 5/SD Figure 5B/Stage VI-VII YBX2.jpg]

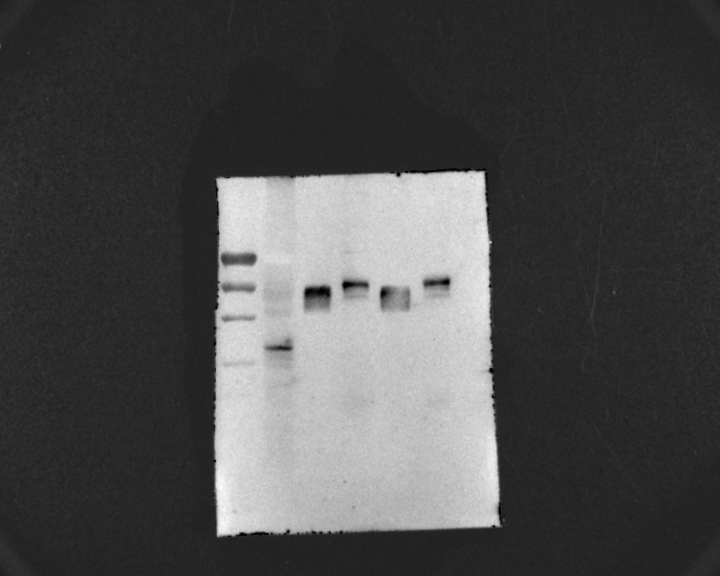

Supplement: Supplementary file 14 — Source data Fig. [file 44318_2025_659_MOESM14_ESM.zip › EMBOJ-2025-121587_Source Data/Source Data Figure 5/SD Figure 5C/SD Figure 5C-GAPDH.tif]

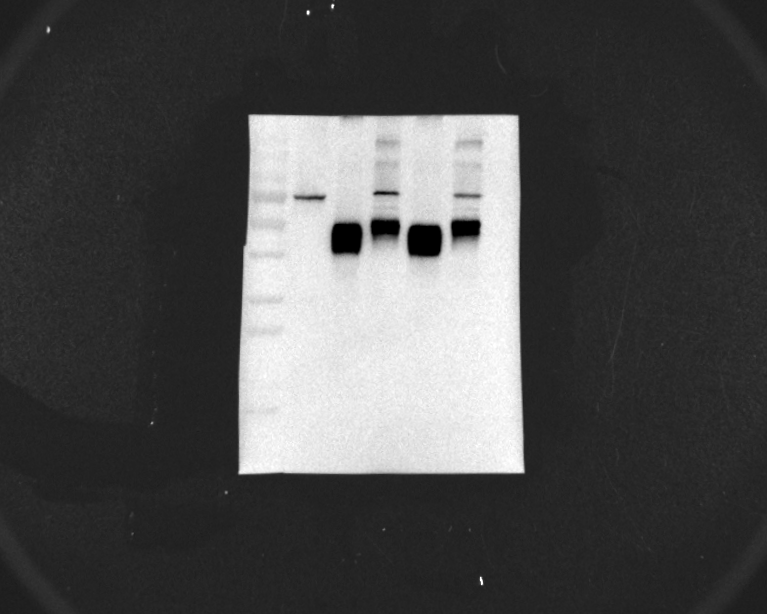

Supplement: Supplementary file 14 — Source data Fig. [file 44318_2025_659_MOESM14_ESM.zip › EMBOJ-2025-121587_Source Data/Source Data Figure 5/SD Figure 5C/SD Figure 5C-IGF2BP3.tif]

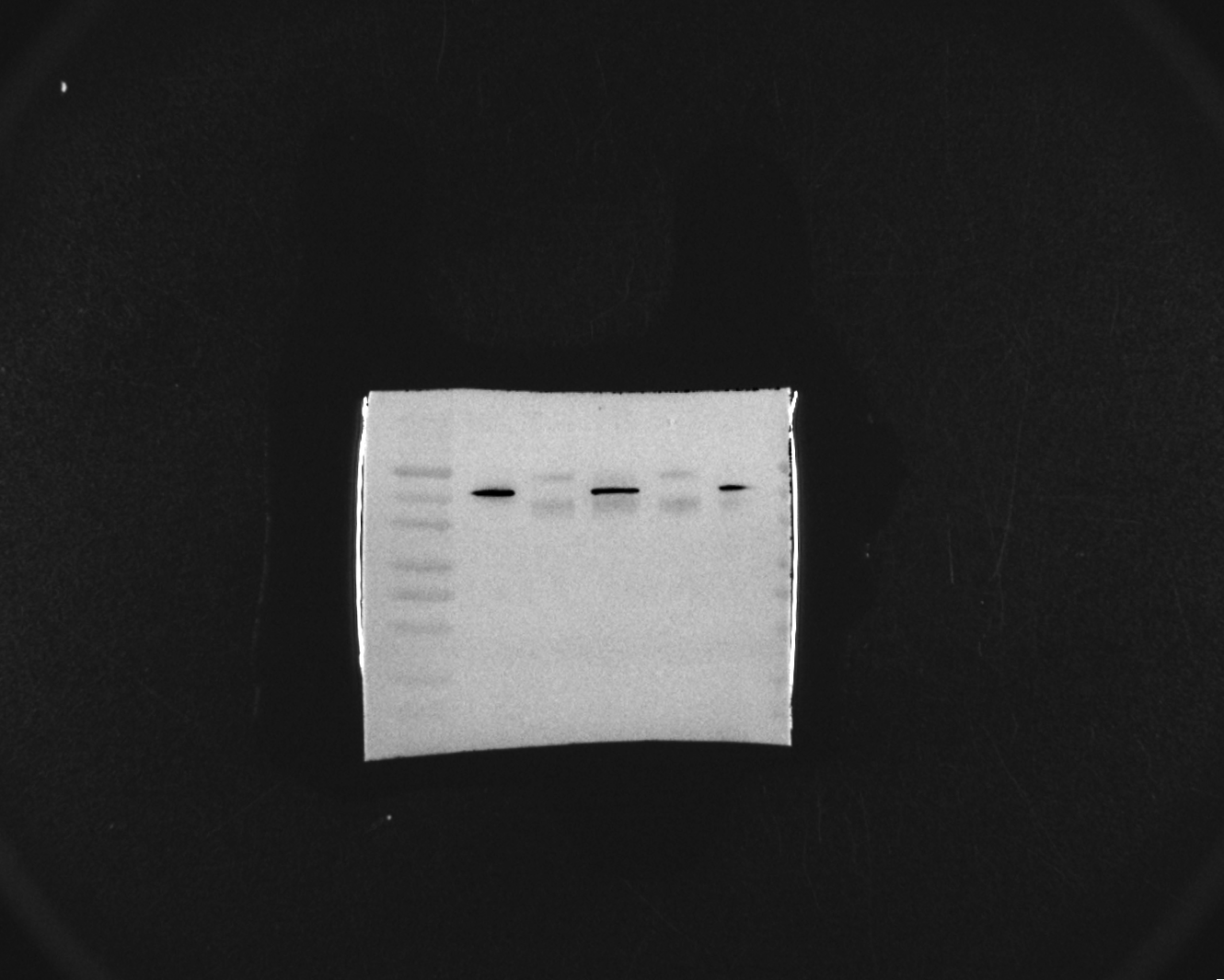

Supplement: Supplementary file 14 — Source data Fig. [file 44318_2025_659_MOESM14_ESM.zip › EMBOJ-2025-121587_Source Data/Source Data Figure 5/SD Figure 5C/SD Figure 5C-YBX2.tif]

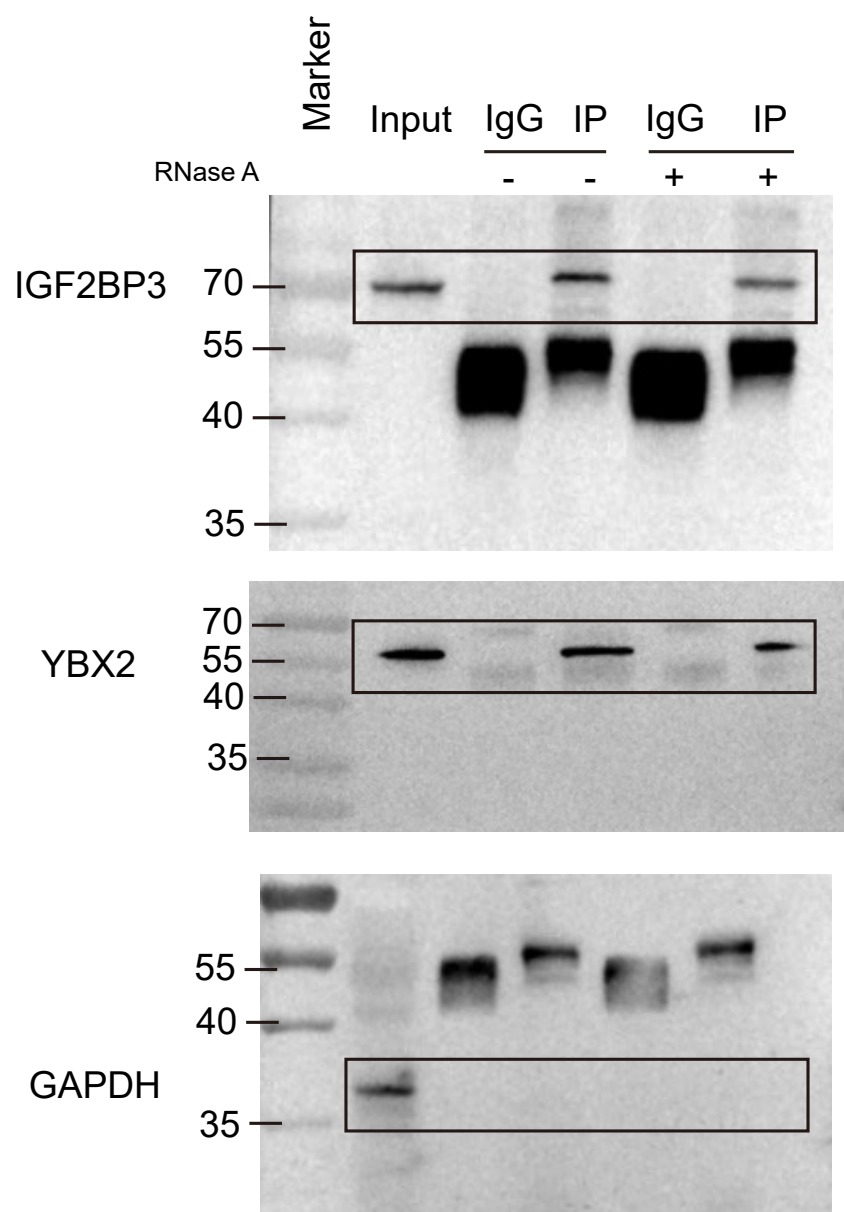

Supplement: Supplementary file 14 — Source data Fig. [file 44318_2025_659_MOESM14_ESM.zip › EMBOJ-2025-121587_Source Data/Source Data Figure 5/SD Figure 5C/SD Figure 5C.pdf]

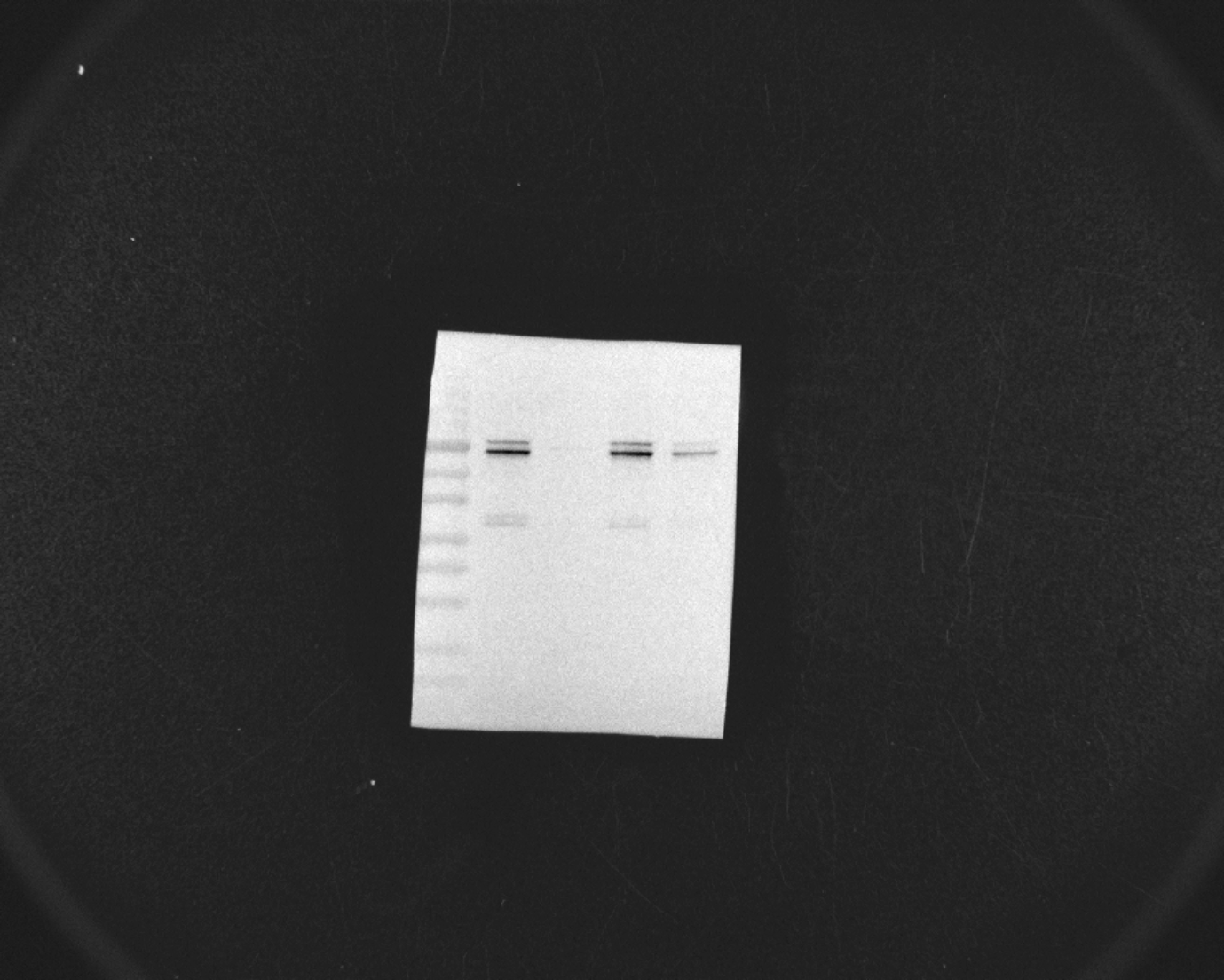

Supplement: Supplementary file 14 — Source data Fig. [file 44318_2025_659_MOESM14_ESM.zip › EMBOJ-2025-121587_Source Data/Source Data Figure 5/SD Figure 5F/SD Figure 5F-FLAG-IGF2BP3.tif]

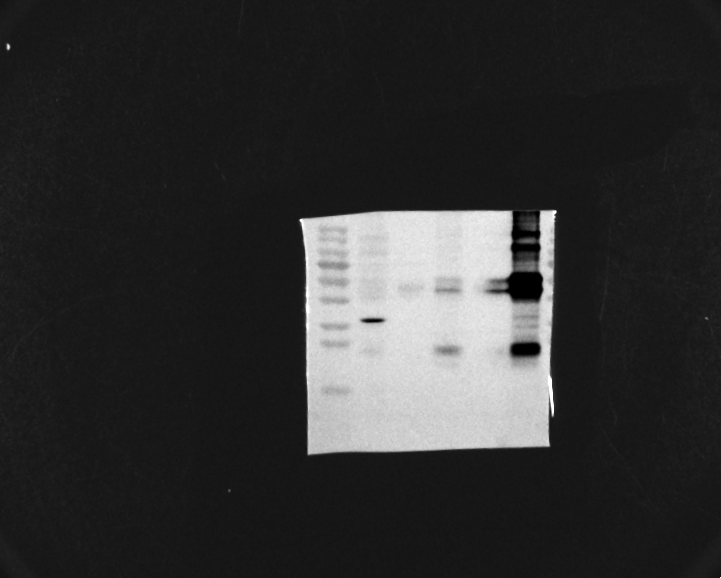

Supplement: Supplementary file 14 — Source data Fig. [file 44318_2025_659_MOESM14_ESM.zip › EMBOJ-2025-121587_Source Data/Source Data Figure 5/SD Figure 5F/SD Figure 5F-GAPDH.tif]

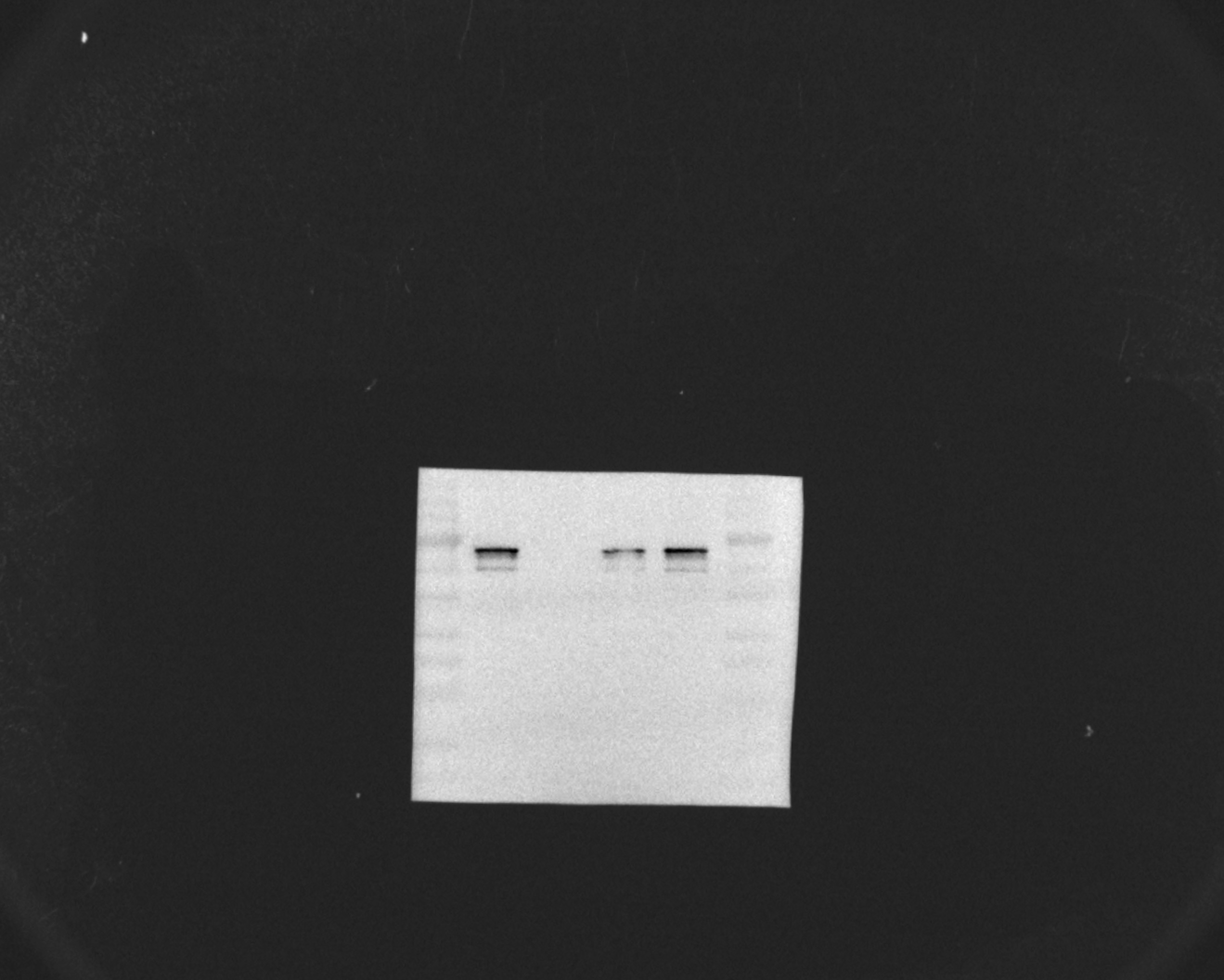

Supplement: Supplementary file 14 — Source data Fig. [file 44318_2025_659_MOESM14_ESM.zip › EMBOJ-2025-121587_Source Data/Source Data Figure 5/SD Figure 5F/SD Figure 5F-MYC-YBX2.tif]

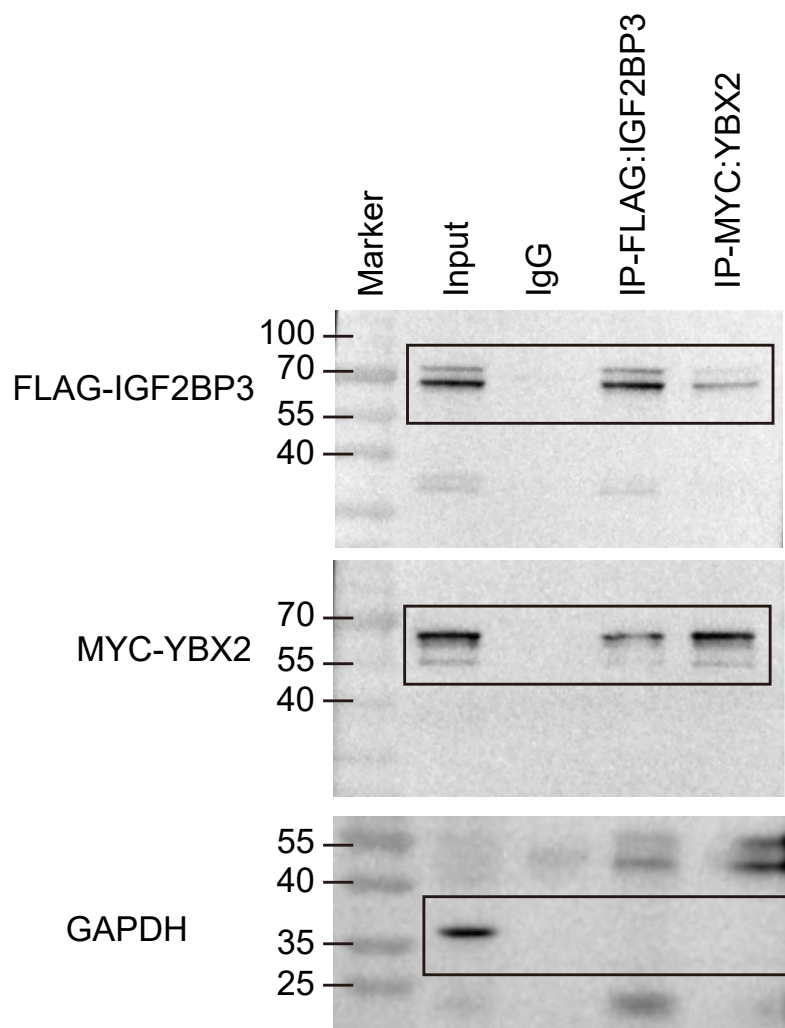

Supplement: Supplementary file 14 — Source data Fig. [file 44318_2025_659_MOESM14_ESM.zip › EMBOJ-2025-121587_Source Data/Source Data Figure 5/SD Figure 5F/SD Figure 5F.pdf]

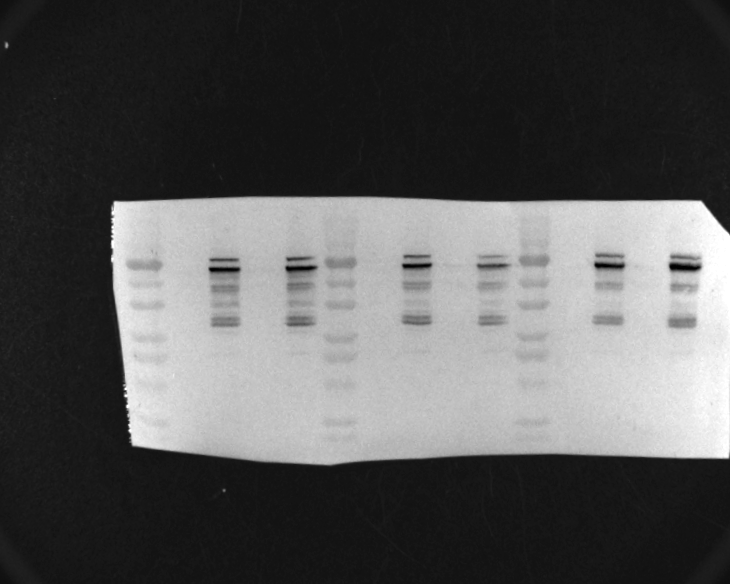

Supplement: Supplementary file 14 — Source data Fig. [file 44318_2025_659_MOESM14_ESM.zip › EMBOJ-2025-121587_Source Data/Source Data Figure 5/SD Figure 5H/SD Figure 5H-FLAG-IGF2BP3.tif]

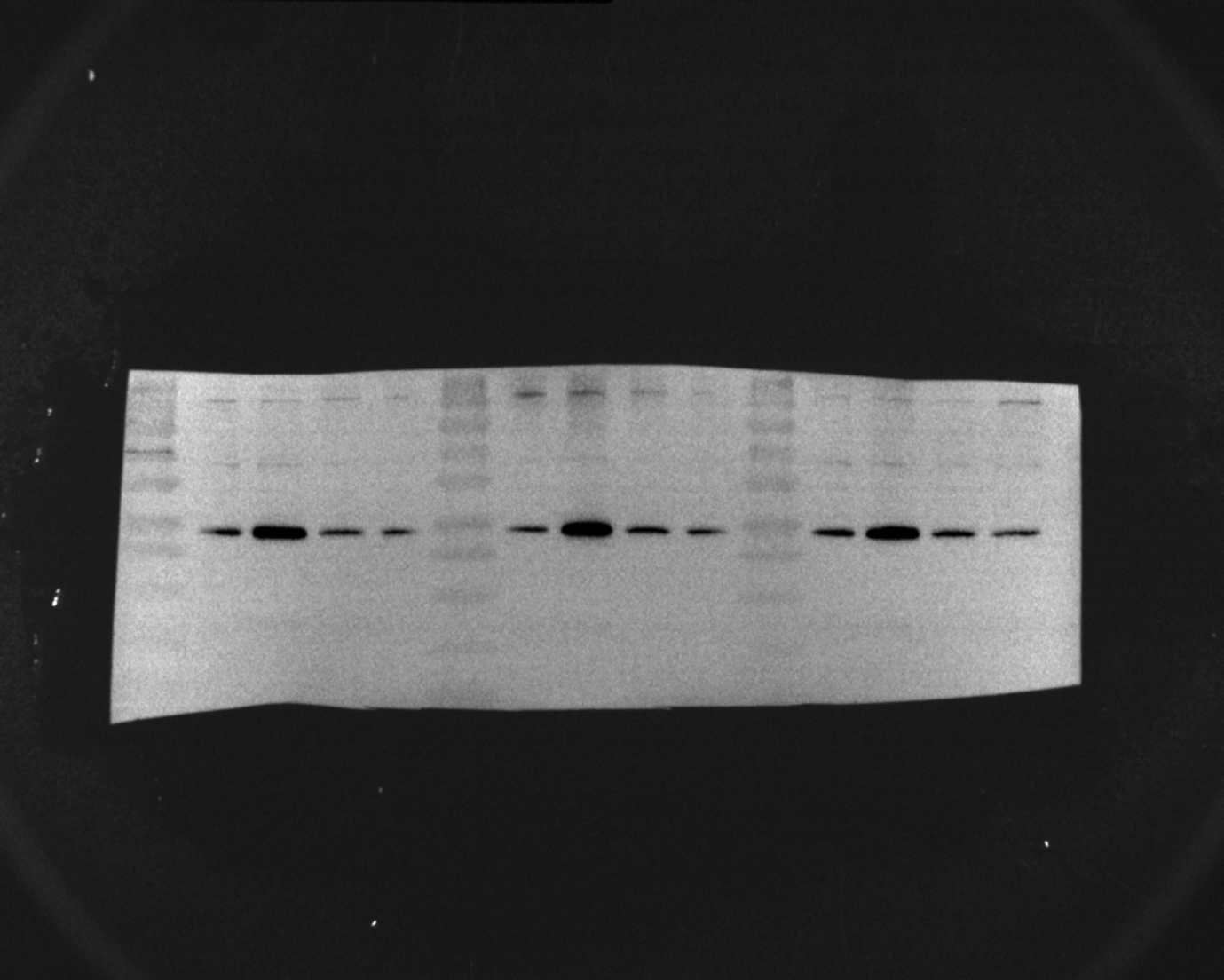

Supplement: Supplementary file 14 — Source data Fig. [file 44318_2025_659_MOESM14_ESM.zip › EMBOJ-2025-121587_Source Data/Source Data Figure 5/SD Figure 5H/SD Figure 5H-GFP.tif]

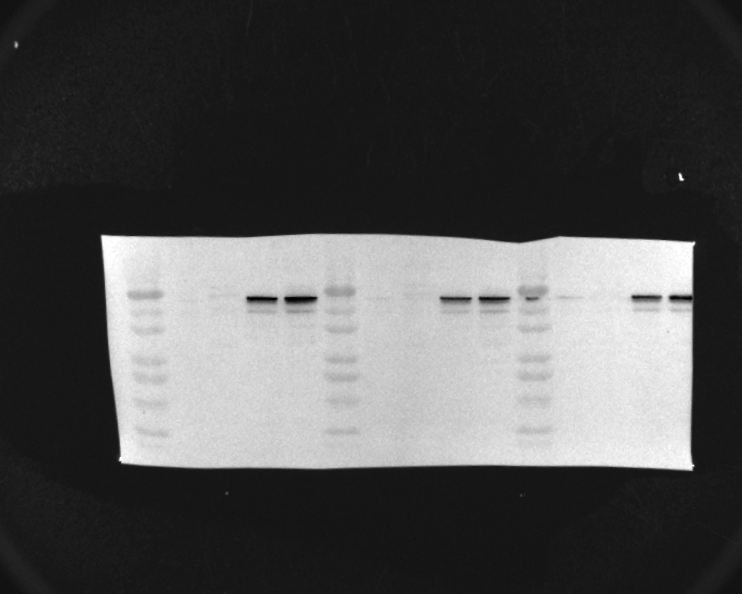

Supplement: Supplementary file 14 — Source data Fig. [file 44318_2025_659_MOESM14_ESM.zip › EMBOJ-2025-121587_Source Data/Source Data Figure 5/SD Figure 5H/SD Figure 5H-MYC-YBX2.tif]

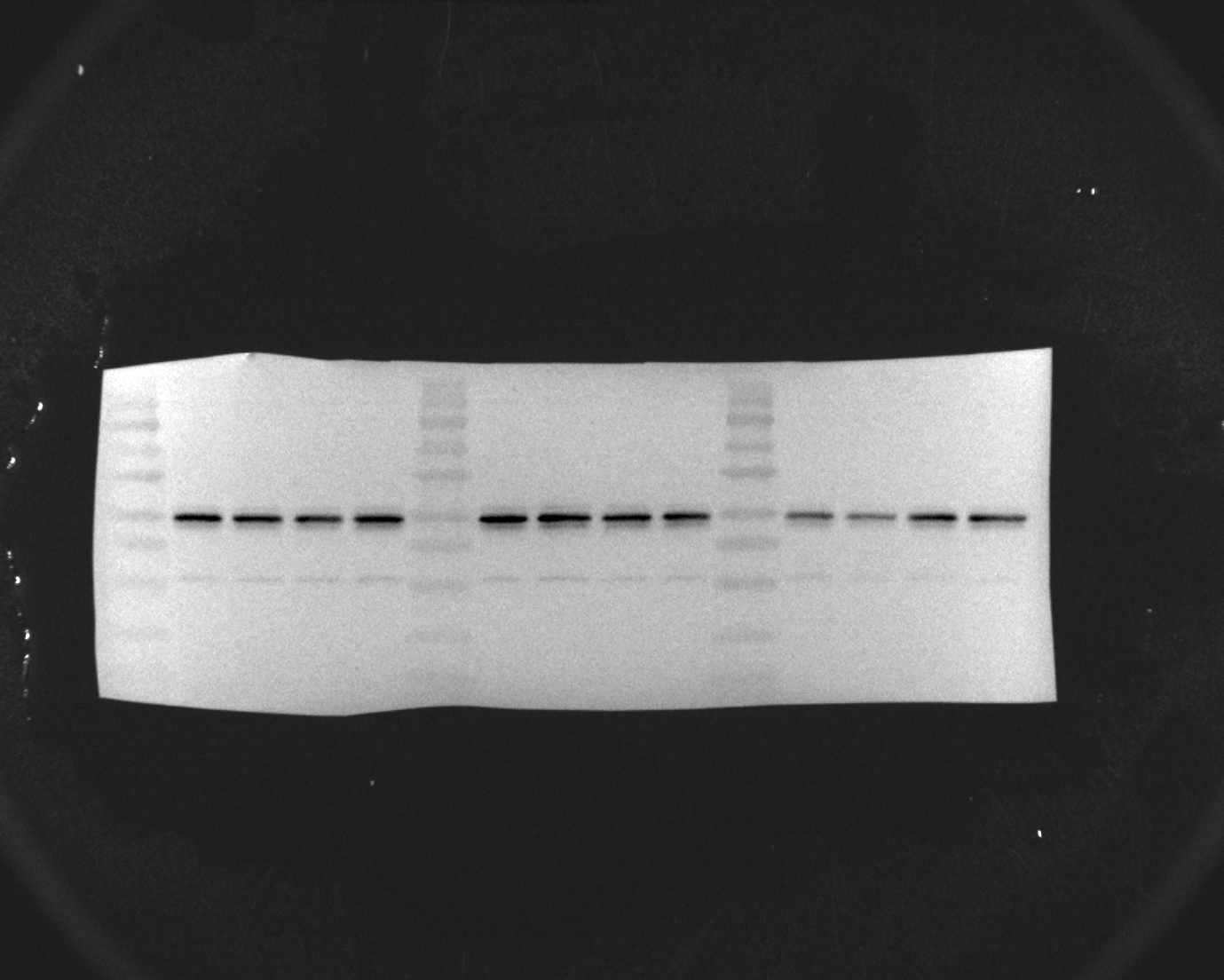

Supplement: Supplementary file 14 — Source data Fig. [file 44318_2025_659_MOESM14_ESM.zip › EMBOJ-2025-121587_Source Data/Source Data Figure 5/SD Figure 5H/SD Figure 5H-mcherry.tif]

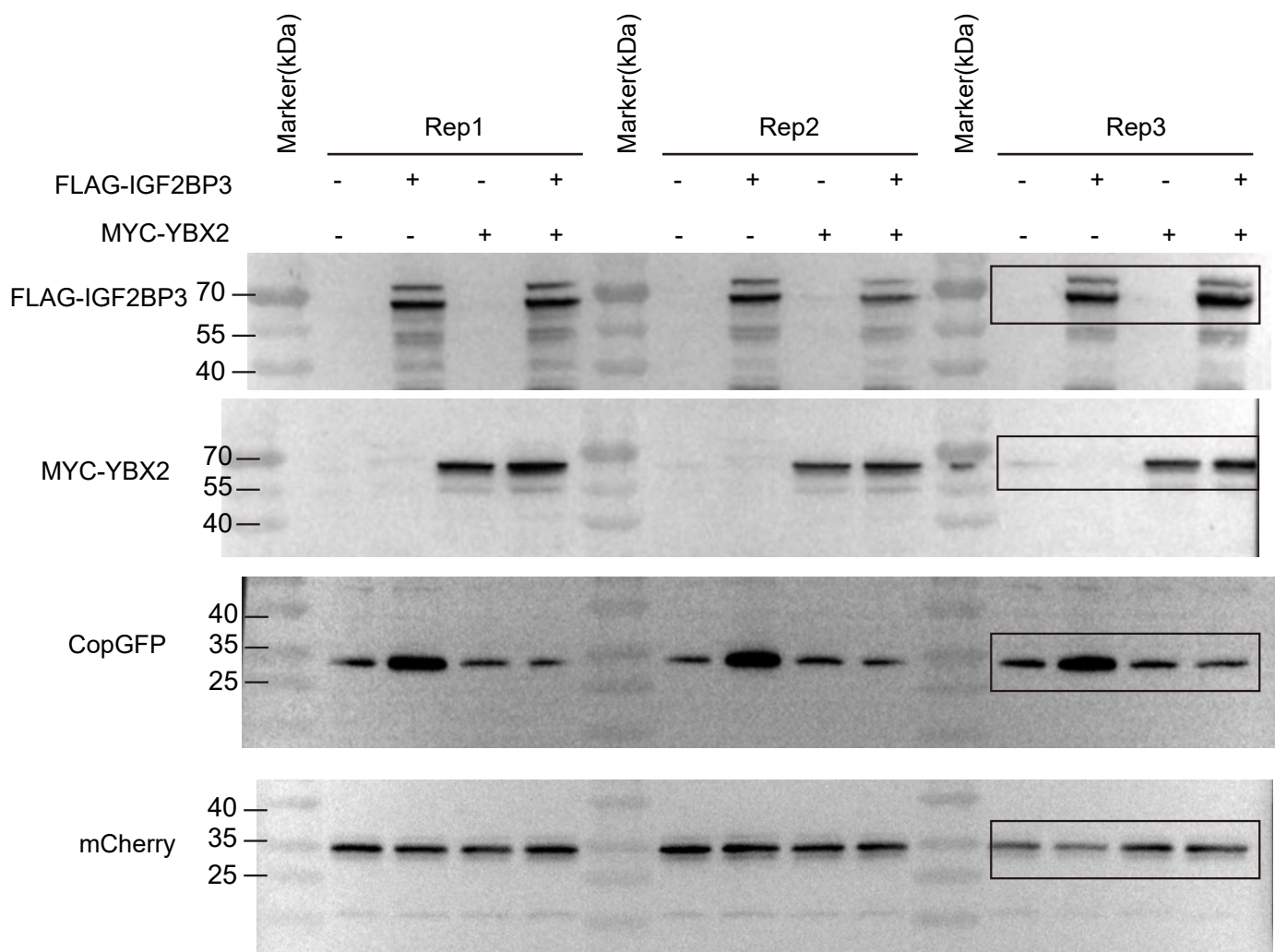

Supplement: Supplementary file 14 — Source data Fig. [file 44318_2025_659_MOESM14_ESM.zip › EMBOJ-2025-121587_Source Data/Source Data Figure 5/SD Figure 5H/SD Figure 5H.pdf]

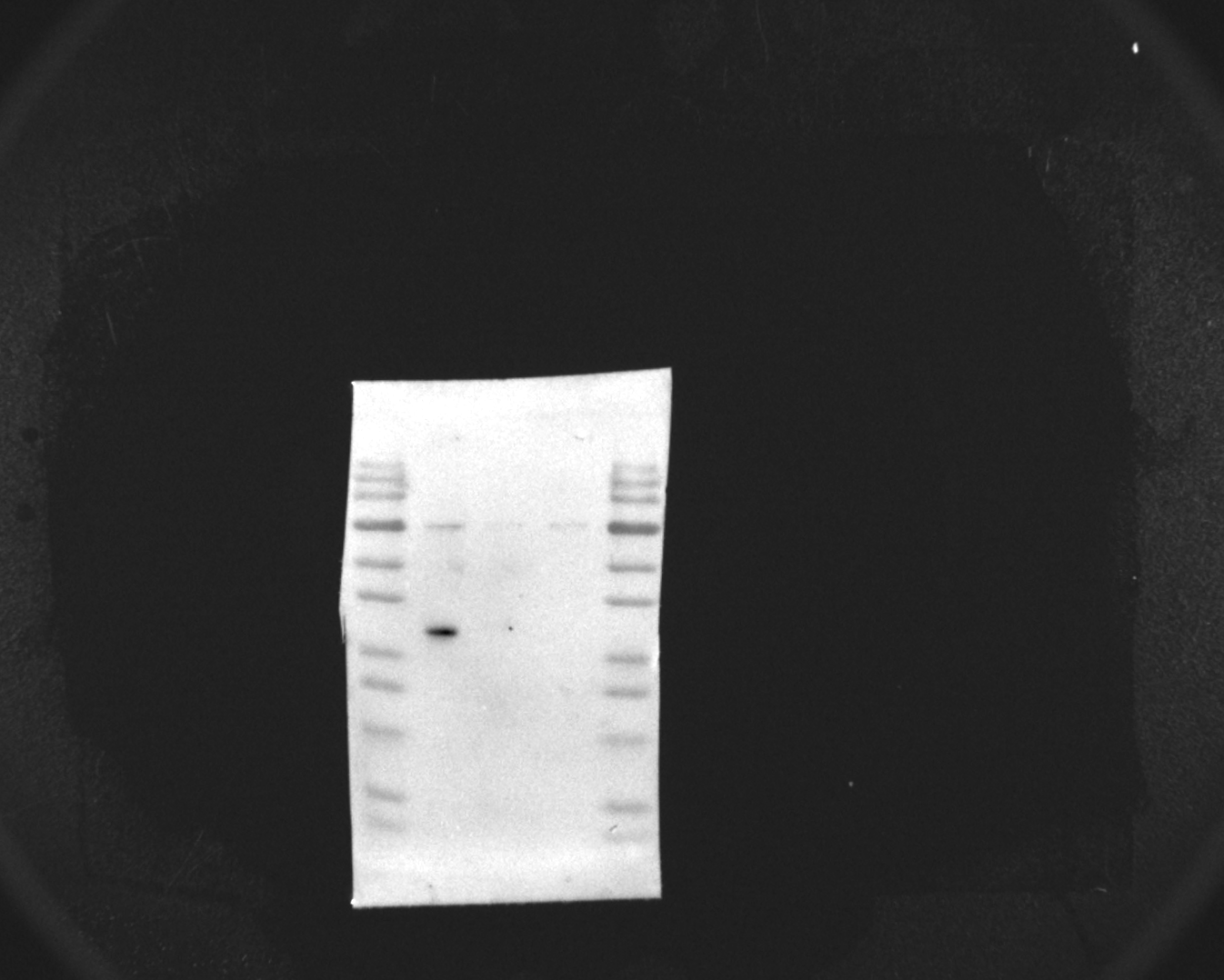

Supplement: Supplementary file 14 — Source data Fig. [file 44318_2025_659_MOESM14_ESM.zip › EMBOJ-2025-121587_Source Data/Source Data Figure 6/SD Figure 6A/SD Figure 6A-GAPDH.tif]

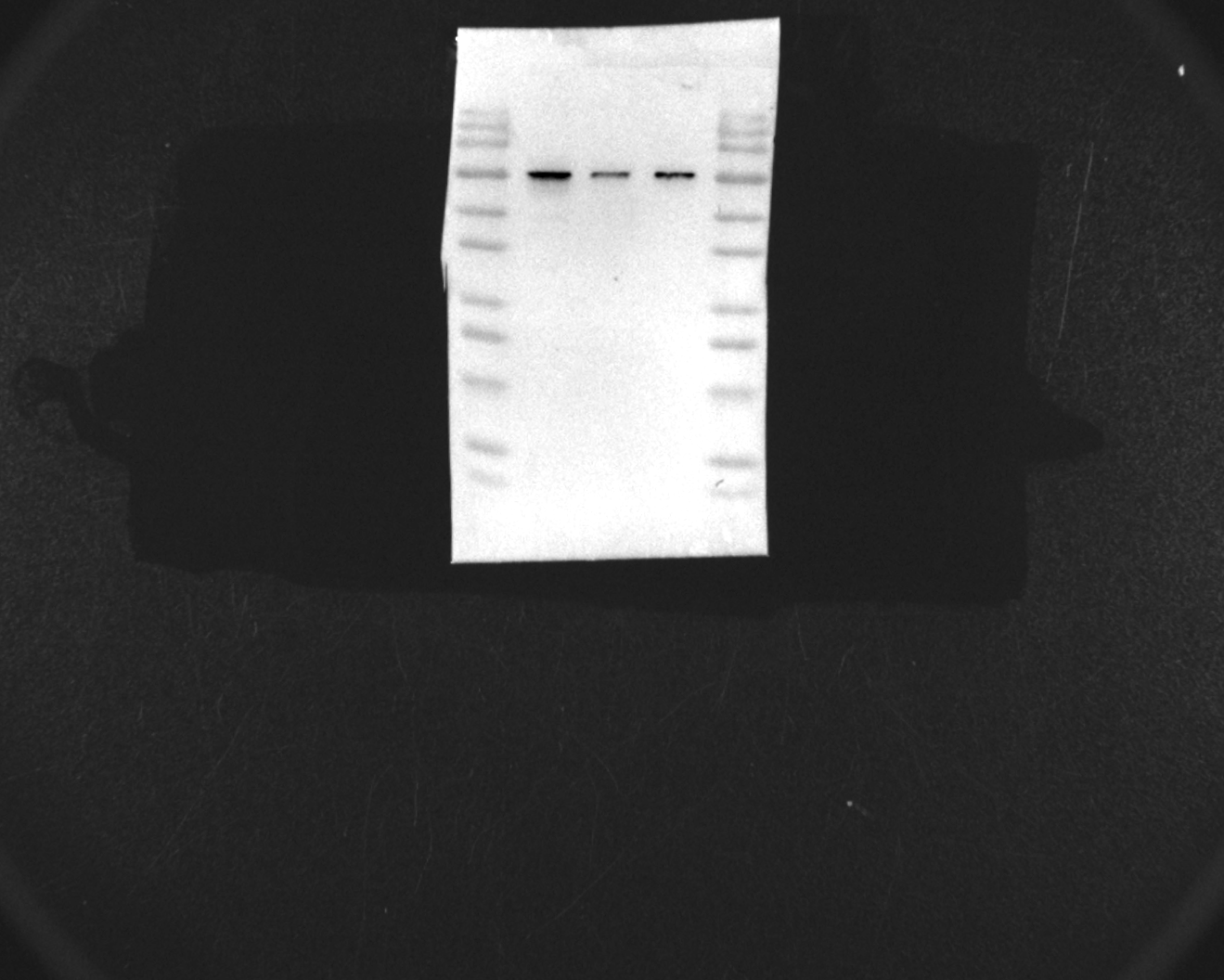

Supplement: Supplementary file 14 — Source data Fig. [file 44318_2025_659_MOESM14_ESM.zip › EMBOJ-2025-121587_Source Data/Source Data Figure 6/SD Figure 6A/SD Figure 6A-IGF2BP3.tif]

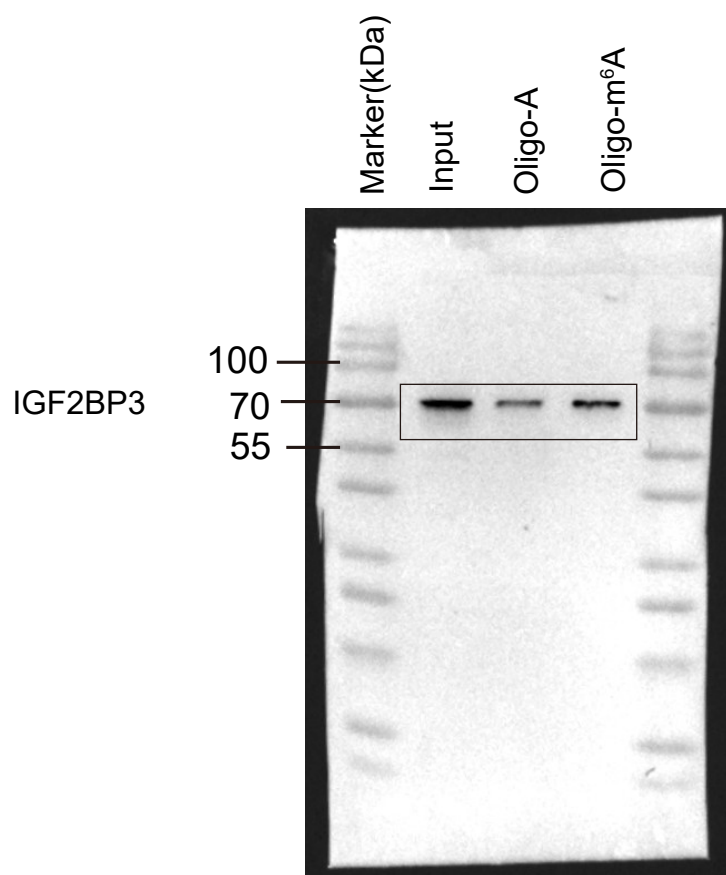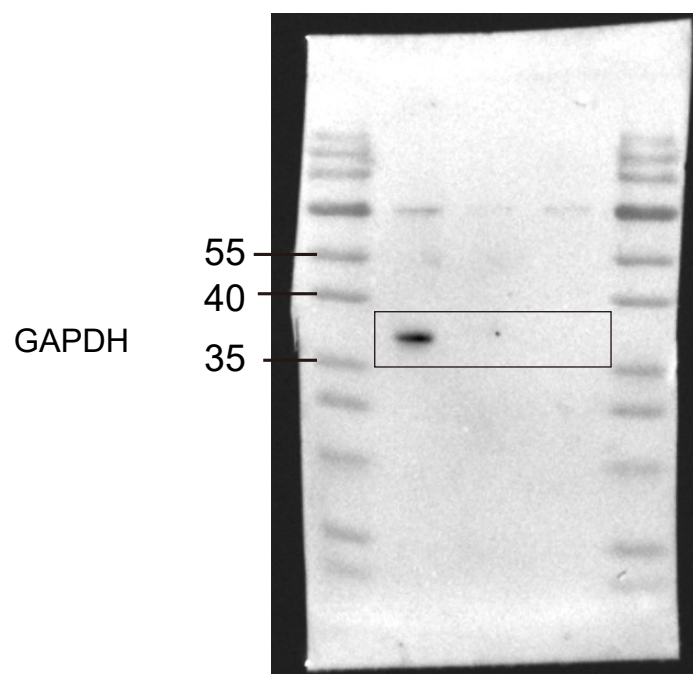

Supplement: Supplementary file 14 — Source data Fig. [file 44318_2025_659_MOESM14_ESM.zip › EMBOJ-2025-121587_Source Data/Source Data Figure 6/SD Figure 6A/SD Figure 6A.pdf]

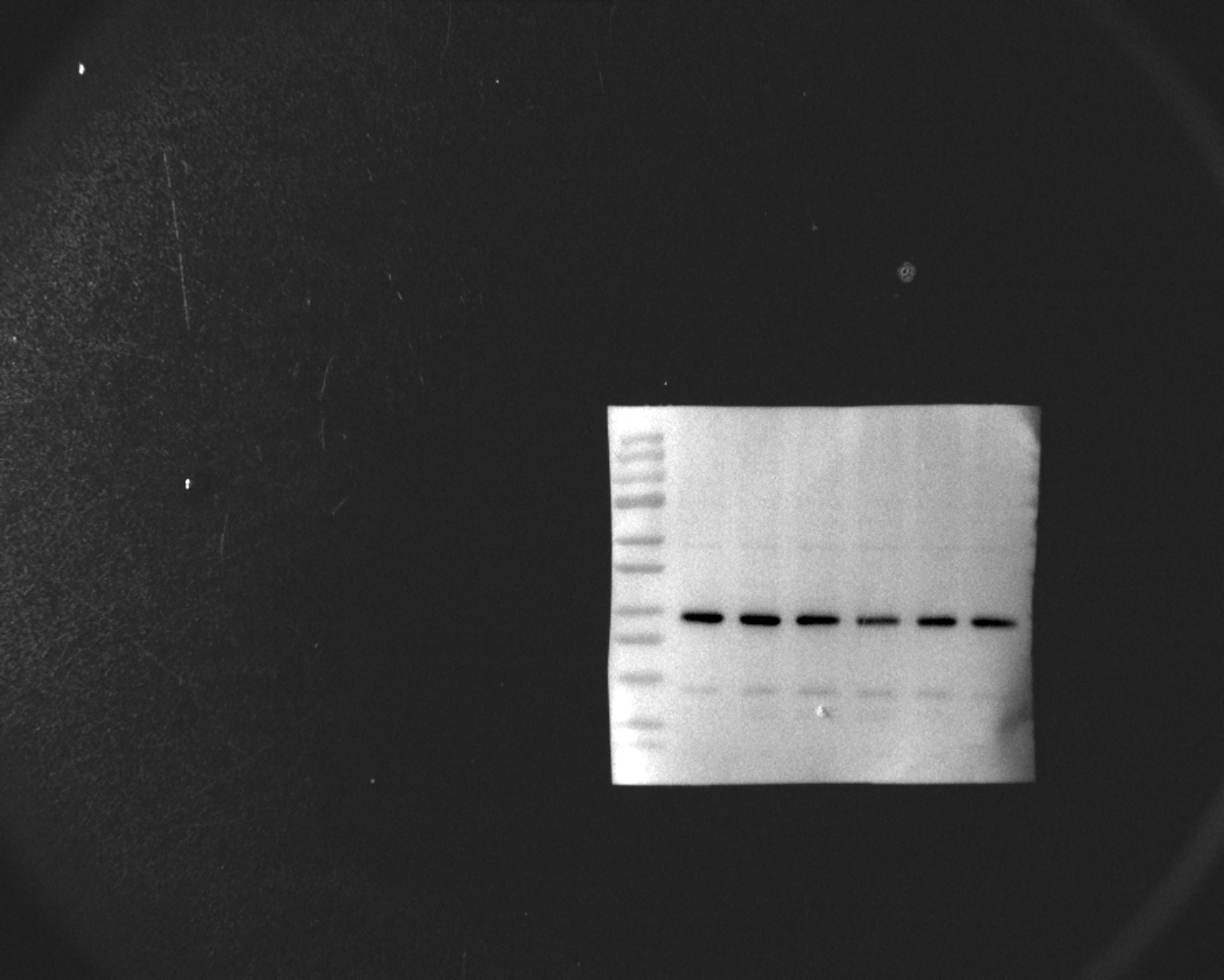

Supplement: Supplementary file 14 — Source data Fig. [file 44318_2025_659_MOESM14_ESM.zip › EMBOJ-2025-121587_Source Data/Source Data Figure 6/SD Figure 6K/SD Figure 6K-GFP.tif]

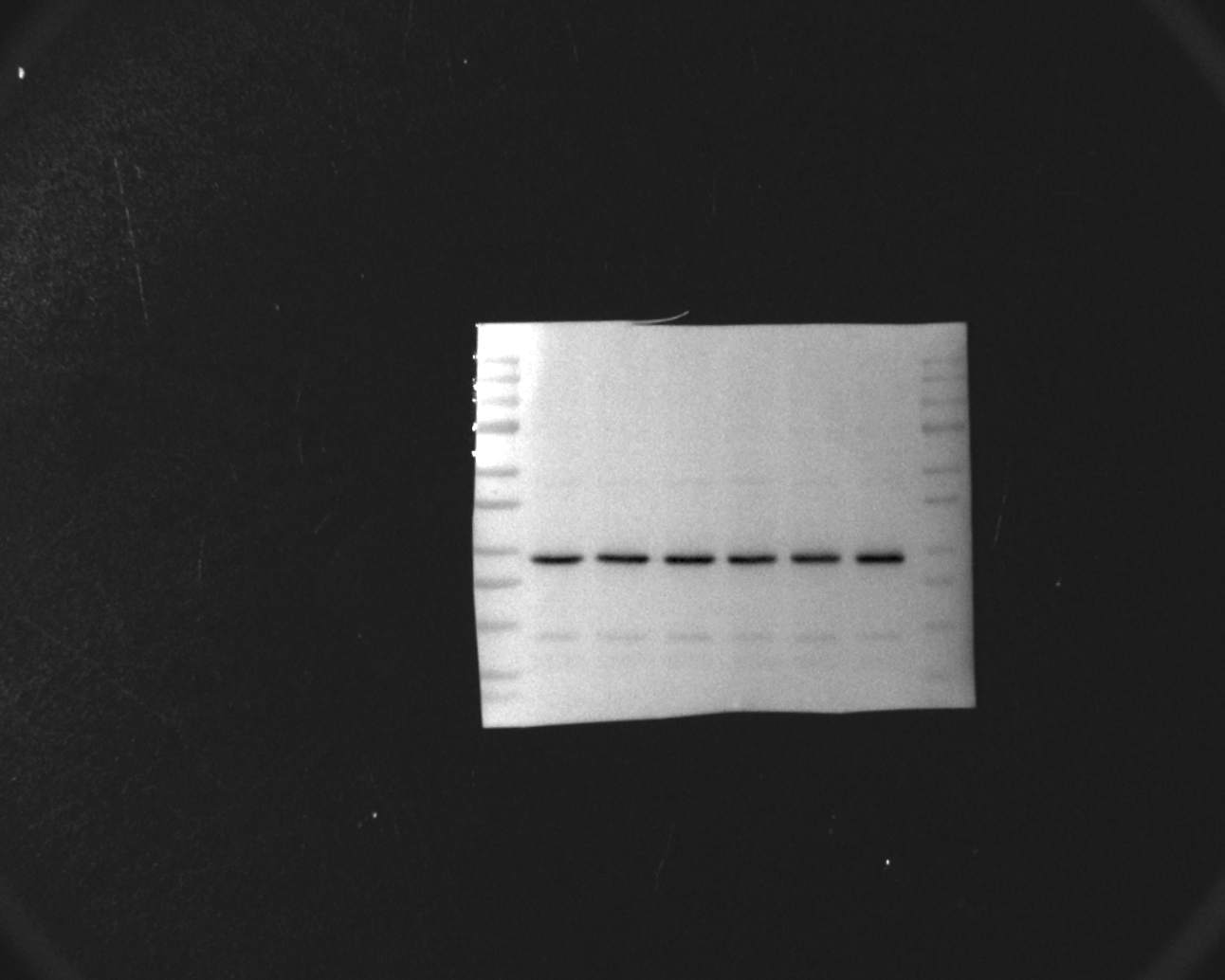

Supplement: Supplementary file 14 — Source data Fig. [file 44318_2025_659_MOESM14_ESM.zip › EMBOJ-2025-121587_Source Data/Source Data Figure 6/SD Figure 6K/SD Figure 6K-mCherry.tif]

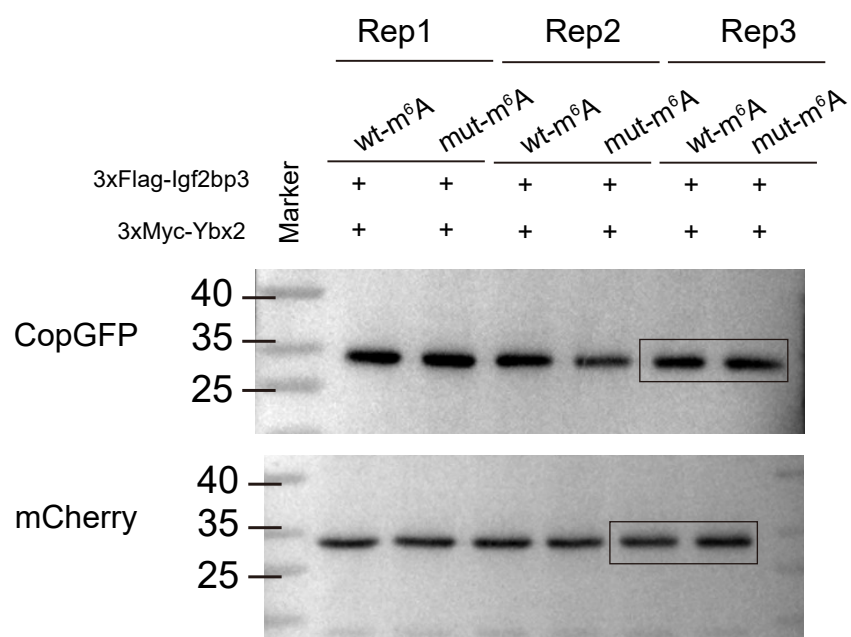

Supplement: Supplementary file 14 — Source data Fig. [file 44318_2025_659_MOESM14_ESM.zip › EMBOJ-2025-121587_Source Data/Source Data Figure 6/SD Figure 6K/SD Figure 6K.pdf]

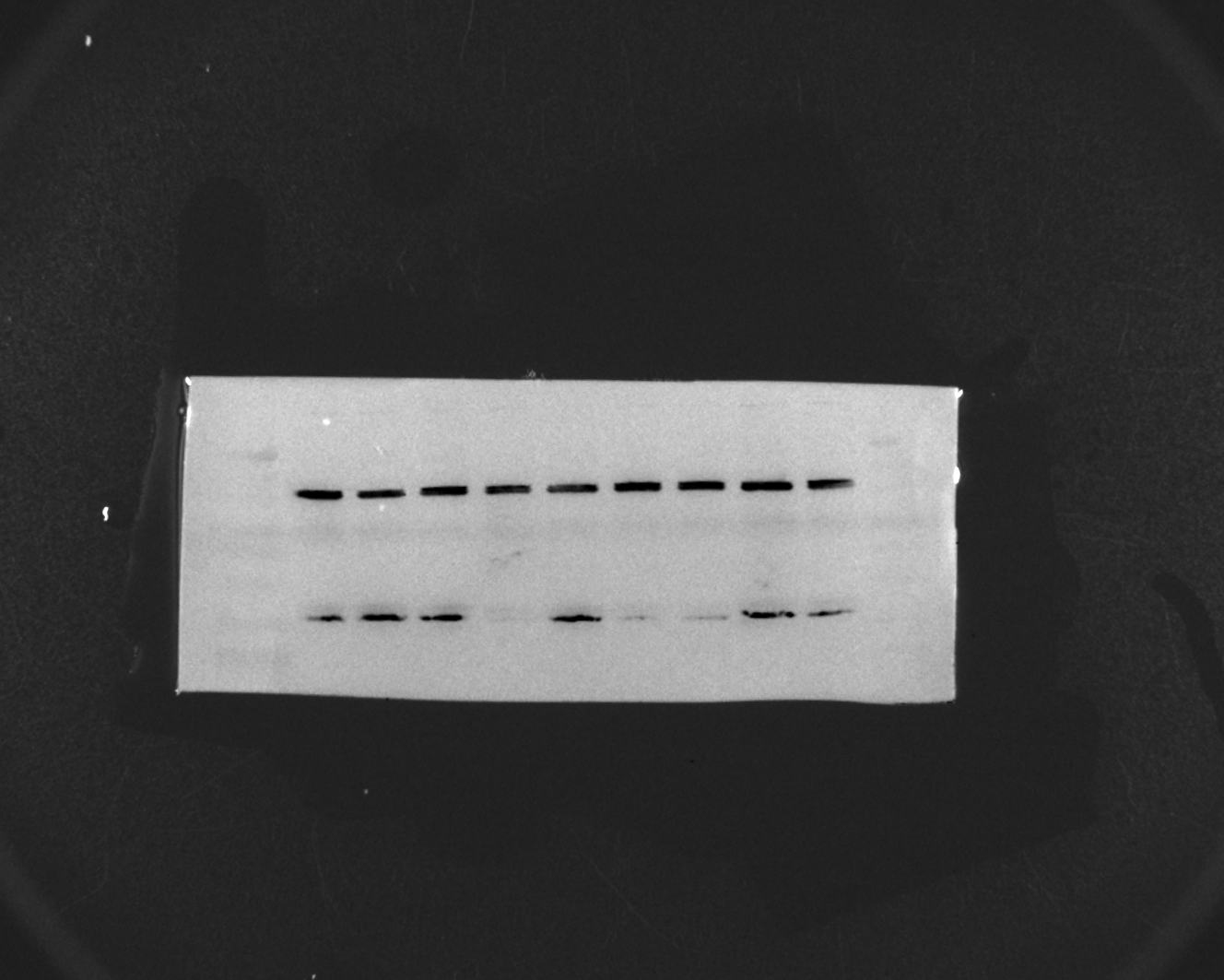

Supplement: Supplementary file 14 — Source data Fig. [file 44318_2025_659_MOESM14_ESM.zip › EMBOJ-2025-121587_Source Data/Source Data Figure 7/SD FIgure 7B/SD Figure 7B-ACTB.tif]

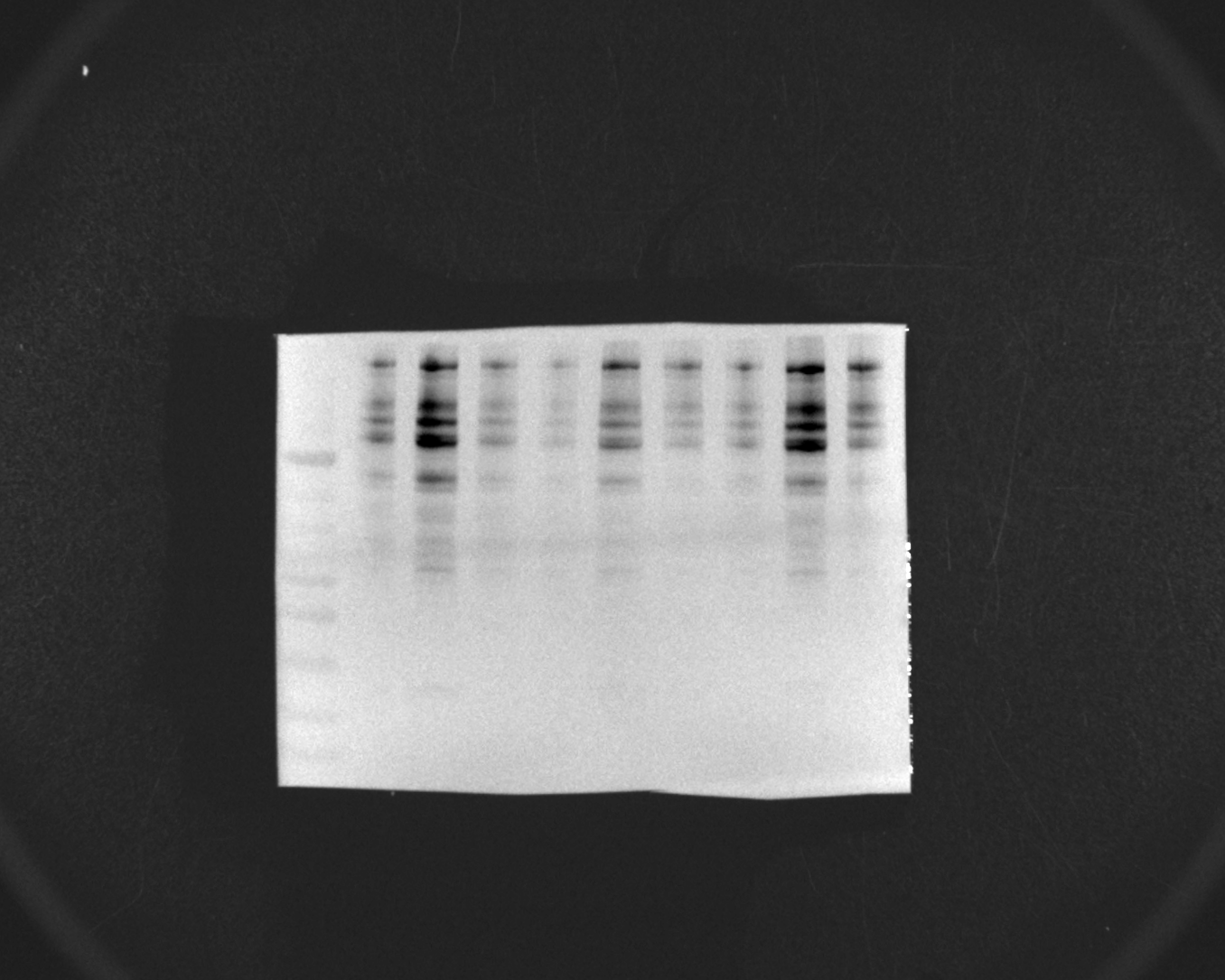

Supplement: Supplementary file 14 — Source data Fig. [file 44318_2025_659_MOESM14_ESM.zip › EMBOJ-2025-121587_Source Data/Source Data Figure 7/SD FIgure 7B/SD Figure 7B-DOT1L.tif]

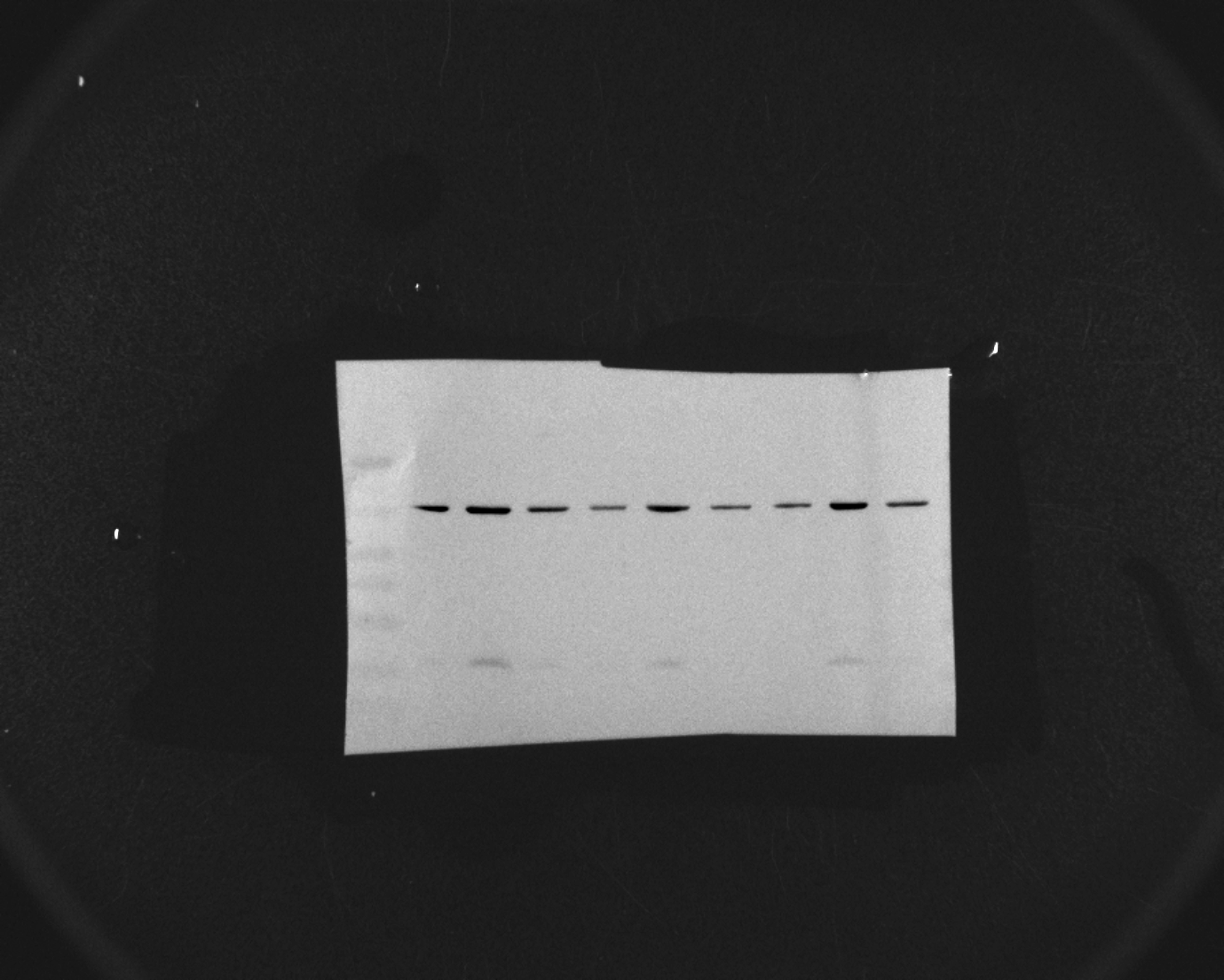

Supplement: Supplementary file 14 — Source data Fig. [file 44318_2025_659_MOESM14_ESM.zip › EMBOJ-2025-121587_Source Data/Source Data Figure 7/SD FIgure 7B/SD Figure 7B-HDAC11.tif]

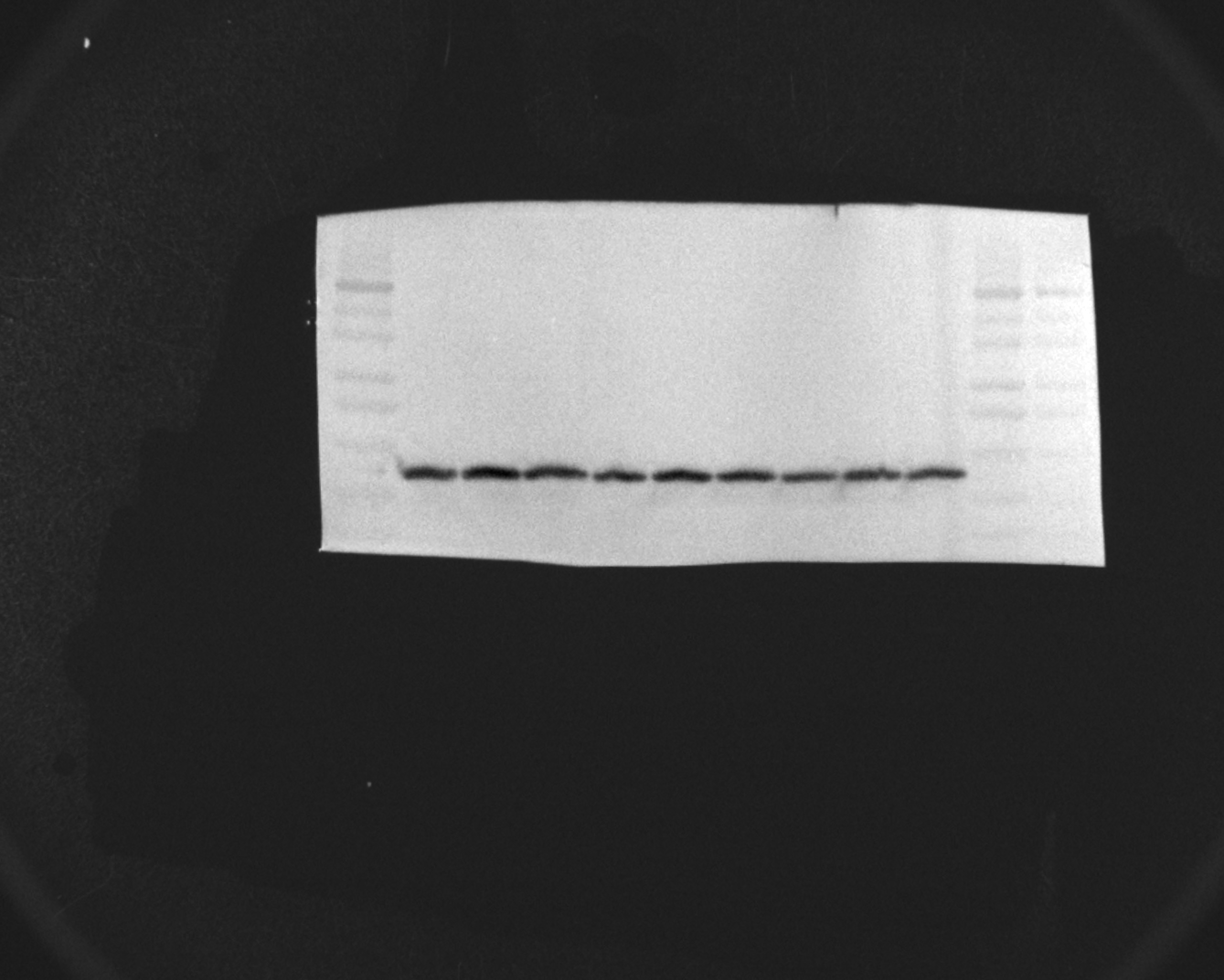

Supplement: Supplementary file 14 — Source data Fig. [file 44318_2025_659_MOESM14_ESM.zip › EMBOJ-2025-121587_Source Data/Source Data Figure 7/SD Figure 7C/SD Figure 7C-H3.tif]

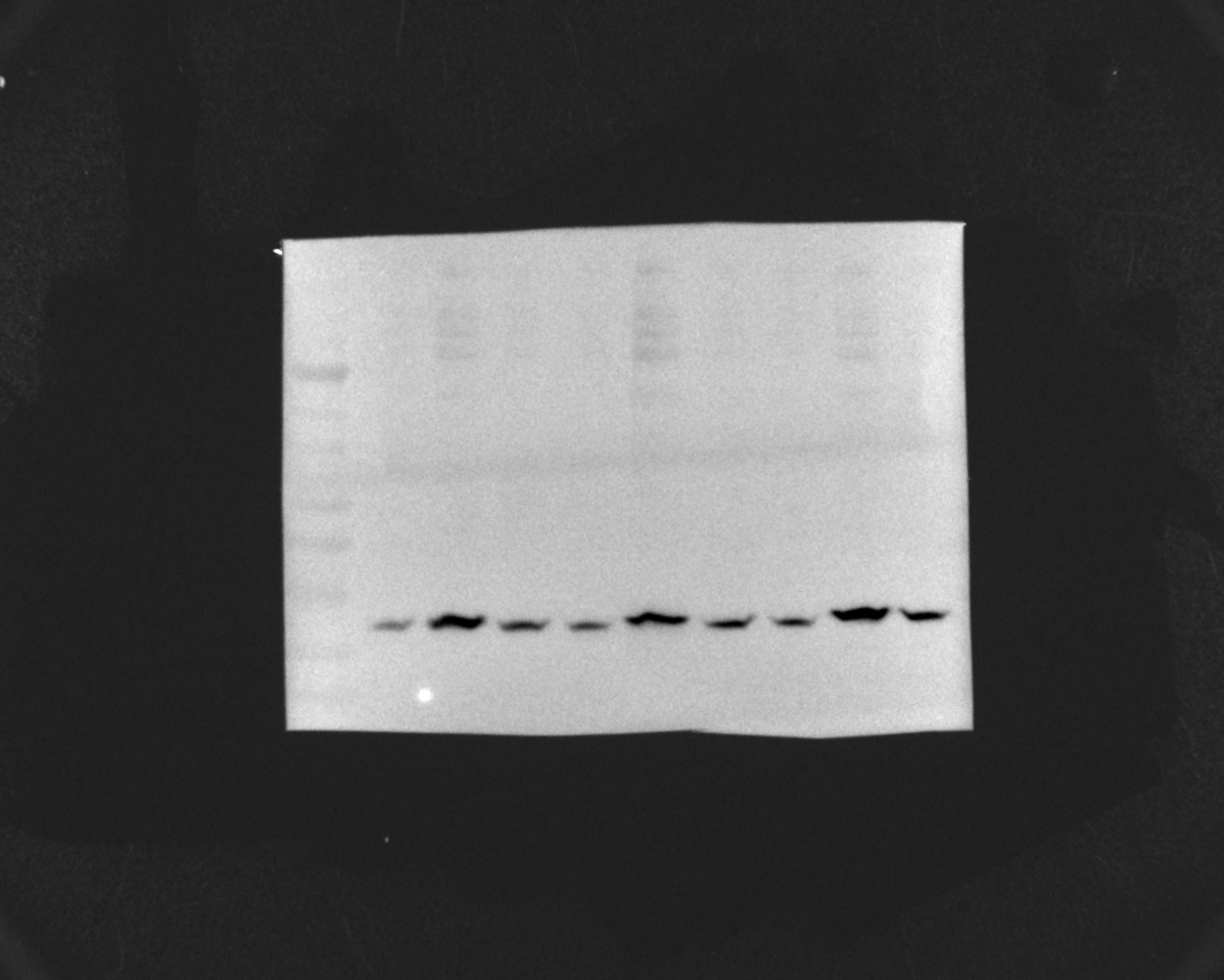

Supplement: Supplementary file 14 — Source data Fig. [file 44318_2025_659_MOESM14_ESM.zip › EMBOJ-2025-121587_Source Data/Source Data Figure 7/SD Figure 7C/SD Figure 7C-H3K79ME2.tif]

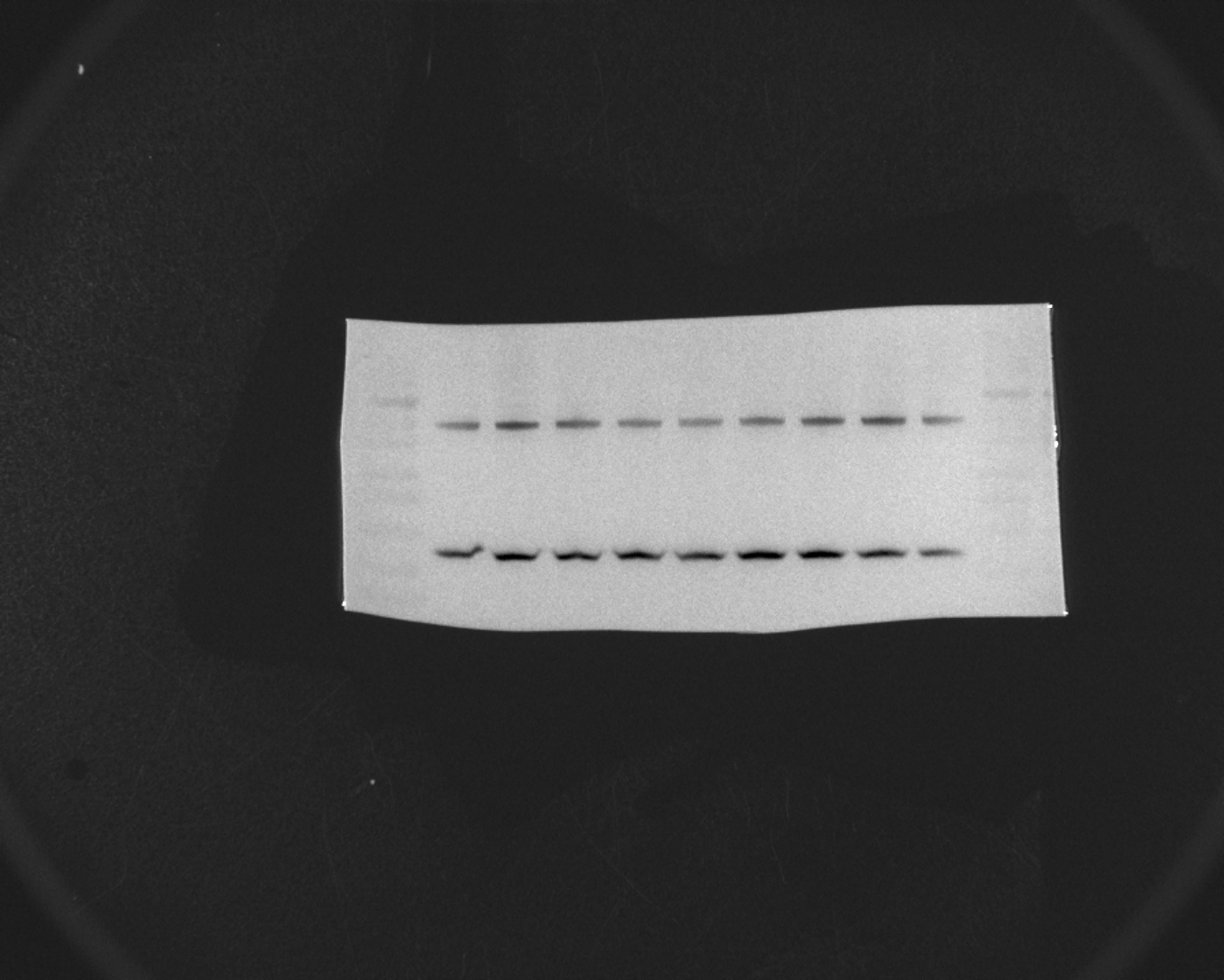

Supplement: Supplementary file 14 — Source data Fig. [file 44318_2025_659_MOESM14_ESM.zip › EMBOJ-2025-121587_Source Data/Source Data Figure 7/SD Figure 7C/SD Figure 7C-H4.tif]

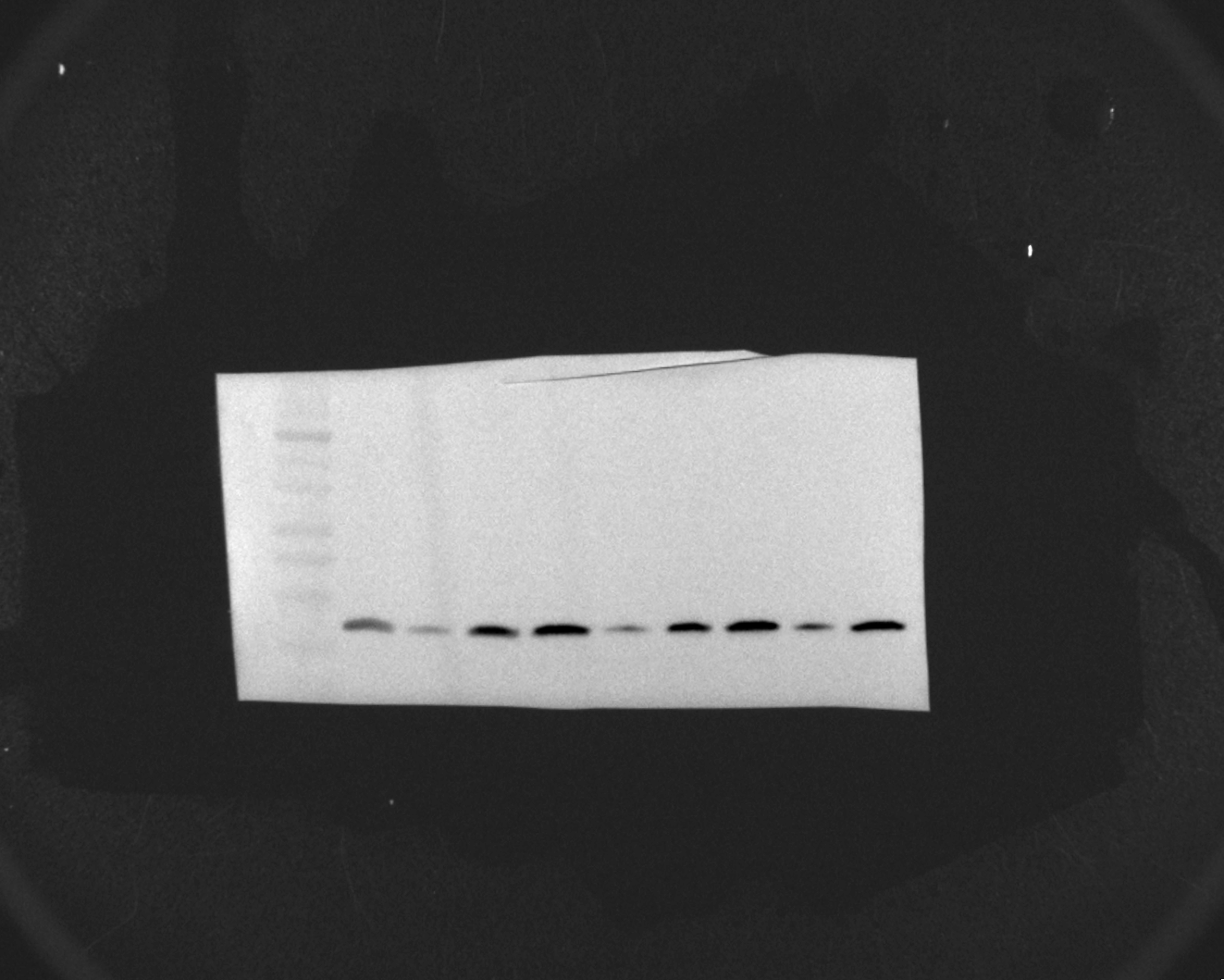

Supplement: Supplementary file 14 — Source data Fig. [file 44318_2025_659_MOESM14_ESM.zip › EMBOJ-2025-121587_Source Data/Source Data Figure 7/SD Figure 7C/SD Figure 7C-H4AC.tif]

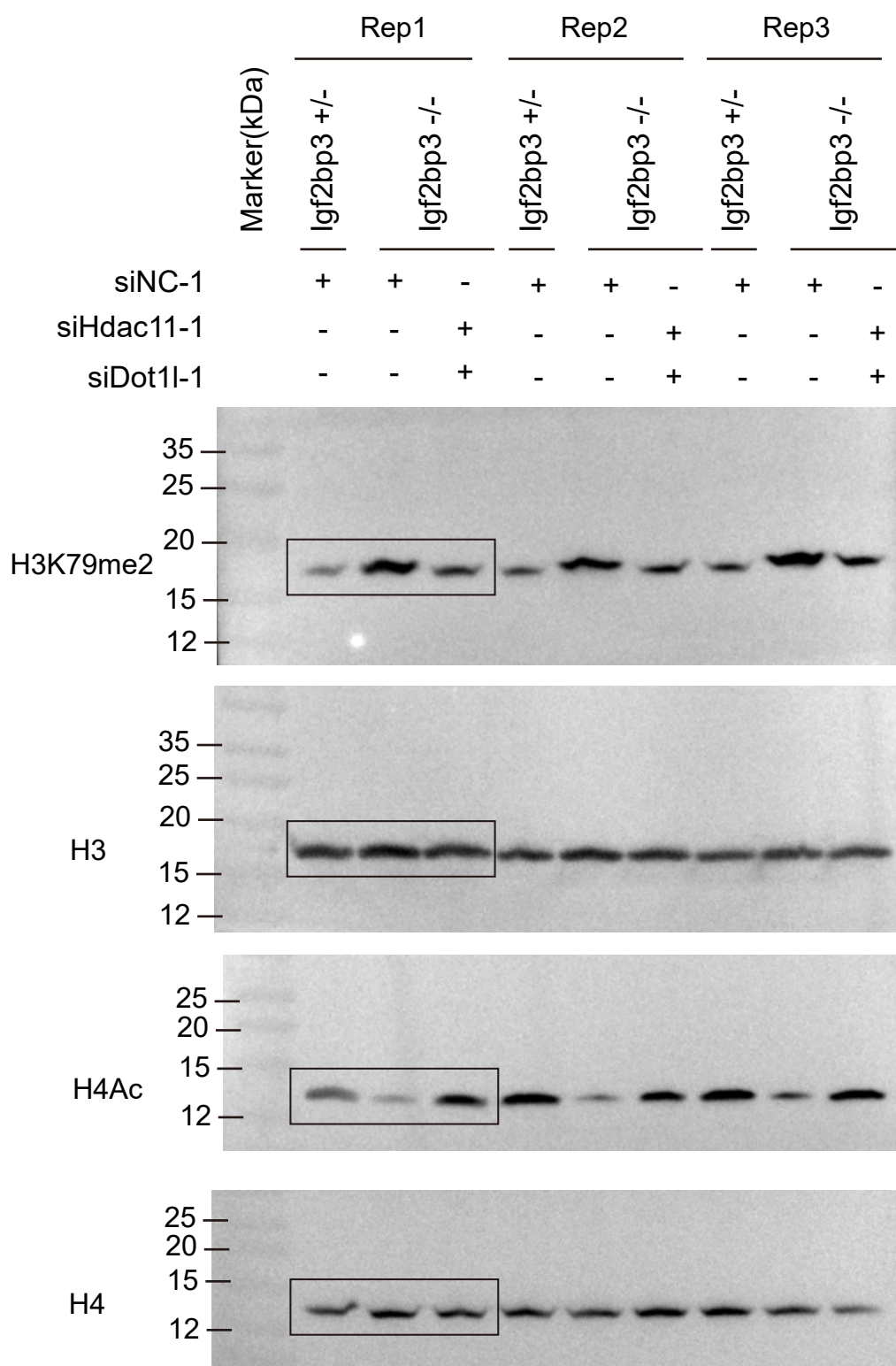

Supplement: Supplementary file 14 — Source data Fig. [file 44318_2025_659_MOESM14_ESM.zip › EMBOJ-2025-121587_Source Data/Source Data Figure 7/SD Figure 7C/SD Figure 7C.pdf]

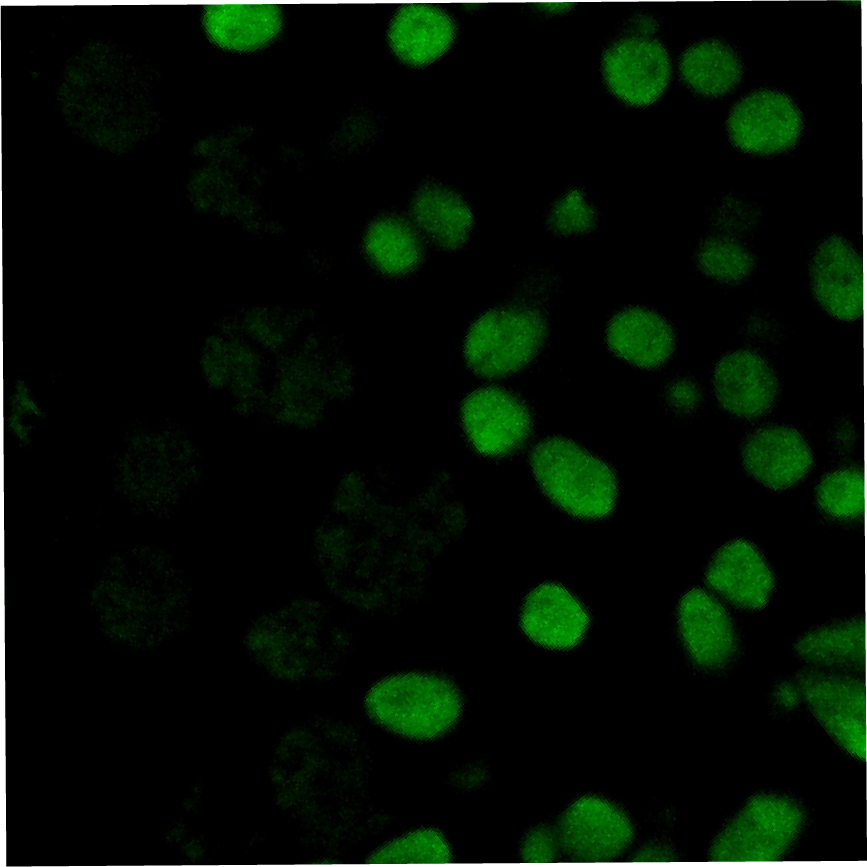

Supplement: Supplementary file 14 — Source data Fig. [file 44318_2025_659_MOESM14_ESM.zip › EMBOJ-2025-121587_Source Data/Source Data Figure 7/SD Figure 7D/Igf2bp3+/- siNC-1 H4Ac.jpg]

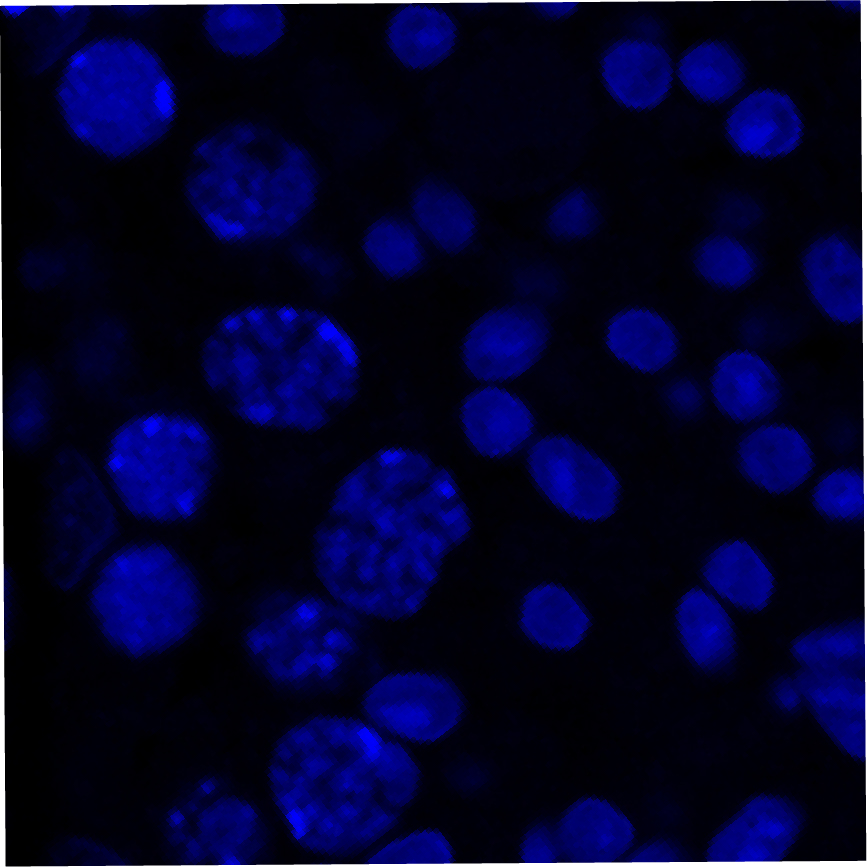

Supplement: Supplementary file 14 — Source data Fig. [file 44318_2025_659_MOESM14_ESM.zip › EMBOJ-2025-121587_Source Data/Source Data Figure 7/SD Figure 7D/Igf2bp3+/- siNC-1 Hoechst.jpg]

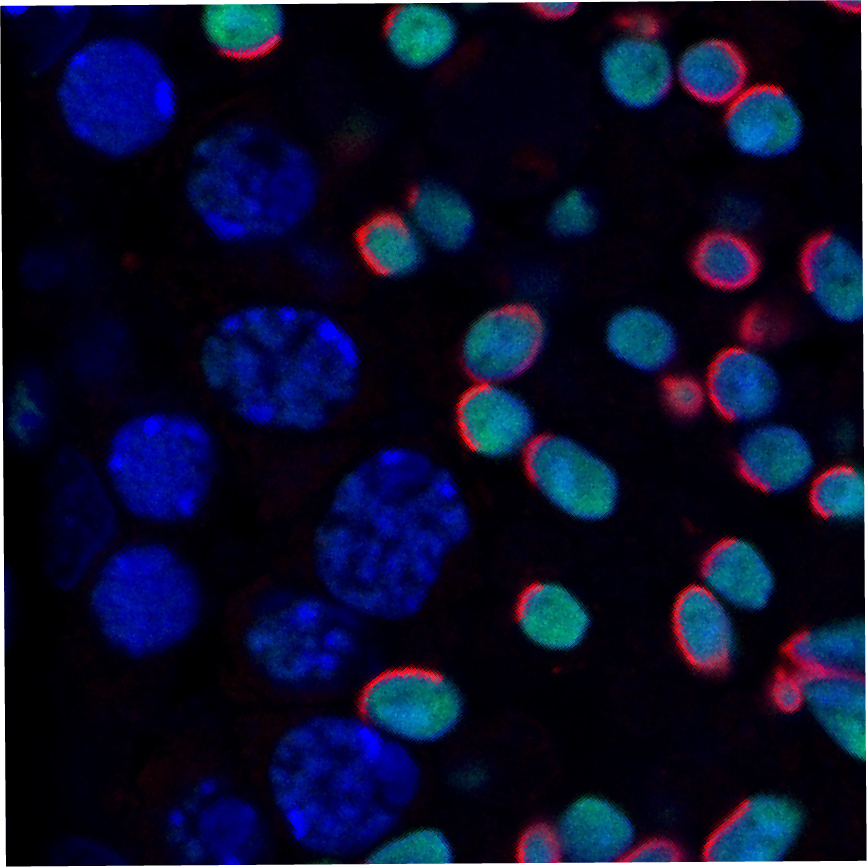

Supplement: Supplementary file 14 — Source data Fig. [file 44318_2025_659_MOESM14_ESM.zip › EMBOJ-2025-121587_Source Data/Source Data Figure 7/SD Figure 7D/Igf2bp3+/- siNC-1 Merge.jpg]

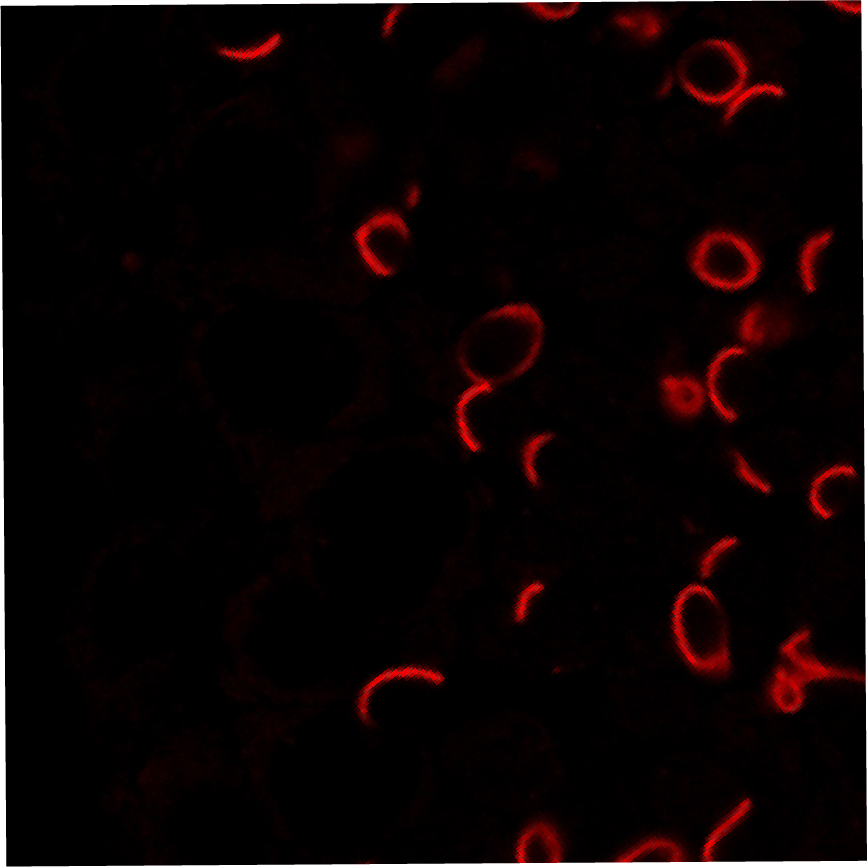

Supplement: Supplementary file 14 — Source data Fig. [file 44318_2025_659_MOESM14_ESM.zip › EMBOJ-2025-121587_Source Data/Source Data Figure 7/SD Figure 7D/Igf2bp3+/- siNC-1 PNA.jpg]

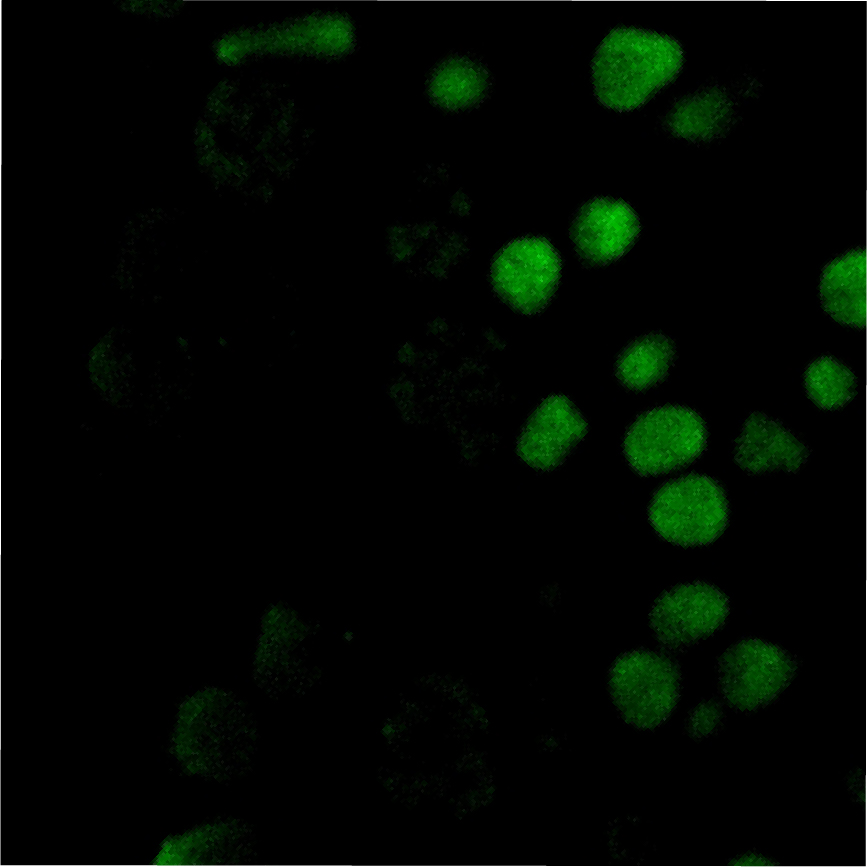

Supplement: Supplementary file 14 — Source data Fig. [file 44318_2025_659_MOESM14_ESM.zip › EMBOJ-2025-121587_Source Data/Source Data Figure 7/SD Figure 7D/Igf2bp3-/- siDot1l/Hdac11-1 H4Ac.jpg]

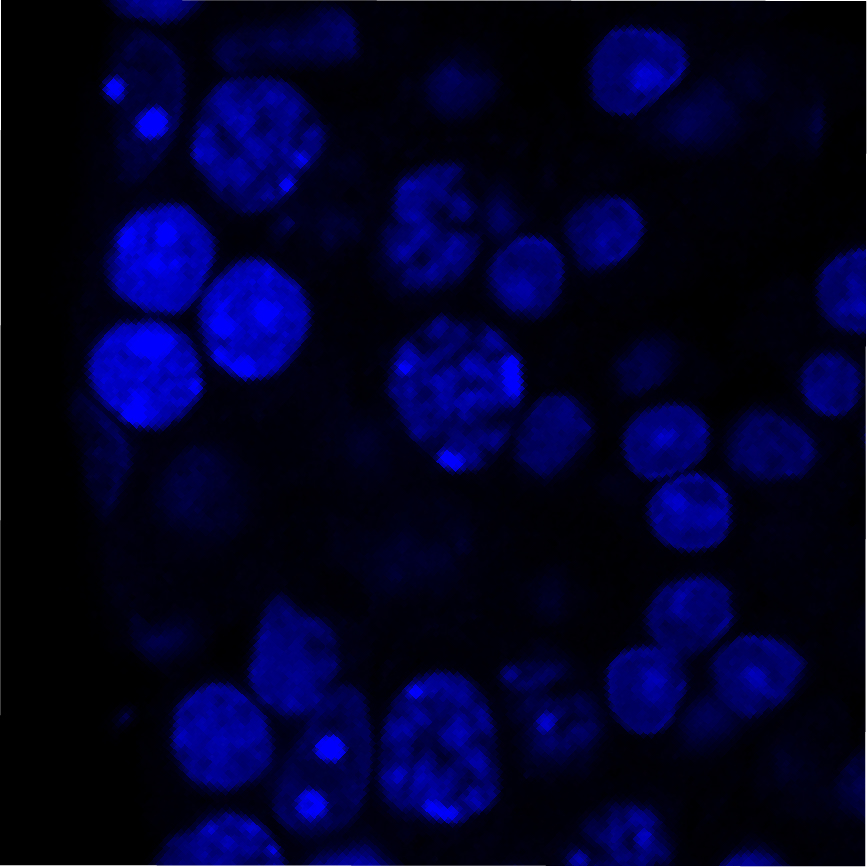

Supplement: Supplementary file 14 — Source data Fig. [file 44318_2025_659_MOESM14_ESM.zip › EMBOJ-2025-121587_Source Data/Source Data Figure 7/SD Figure 7D/Igf2bp3-/- siDot1l/Hdac11-1 Hoechst.jpg]

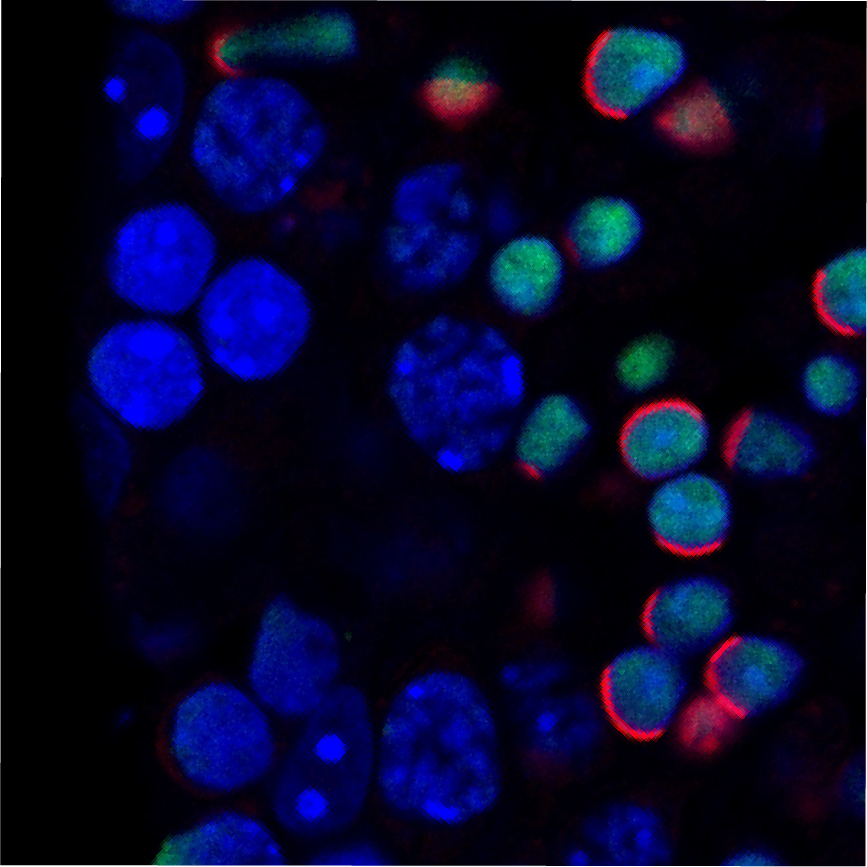

Supplement: Supplementary file 14 — Source data Fig. [file 44318_2025_659_MOESM14_ESM.zip › EMBOJ-2025-121587_Source Data/Source Data Figure 7/SD Figure 7D/Igf2bp3-/- siDot1l/Hdac11-1 Merge.jpg]

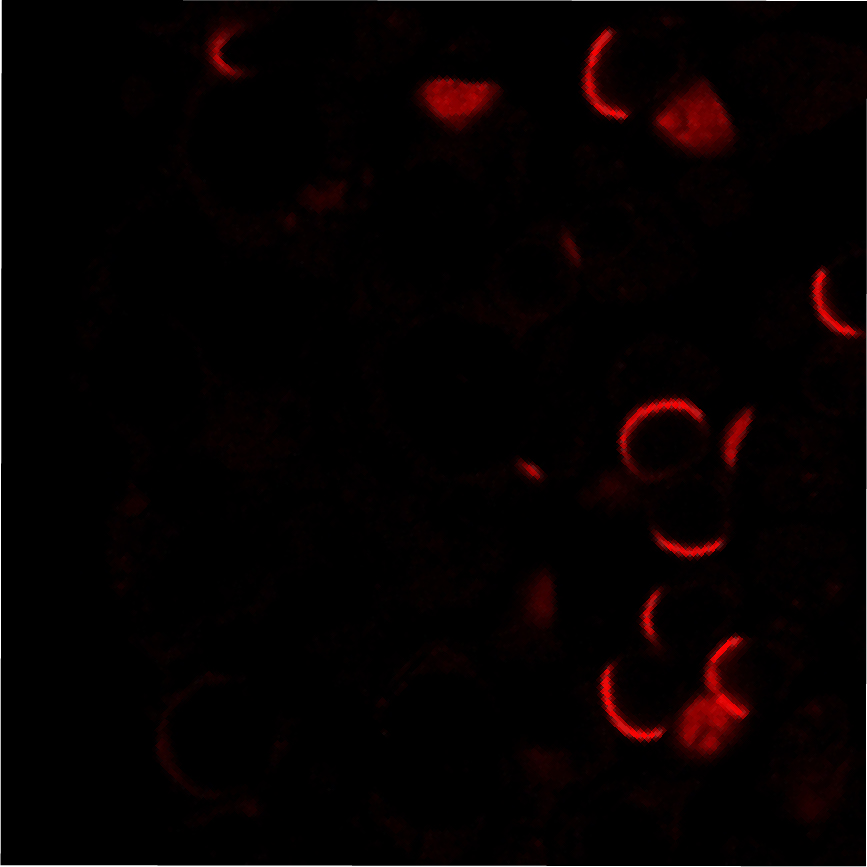

Supplement: Supplementary file 14 — Source data Fig. [file 44318_2025_659_MOESM14_ESM.zip › EMBOJ-2025-121587_Source Data/Source Data Figure 7/SD Figure 7D/Igf2bp3-/- siDot1l/Hdac11-1 PNA.jpg]

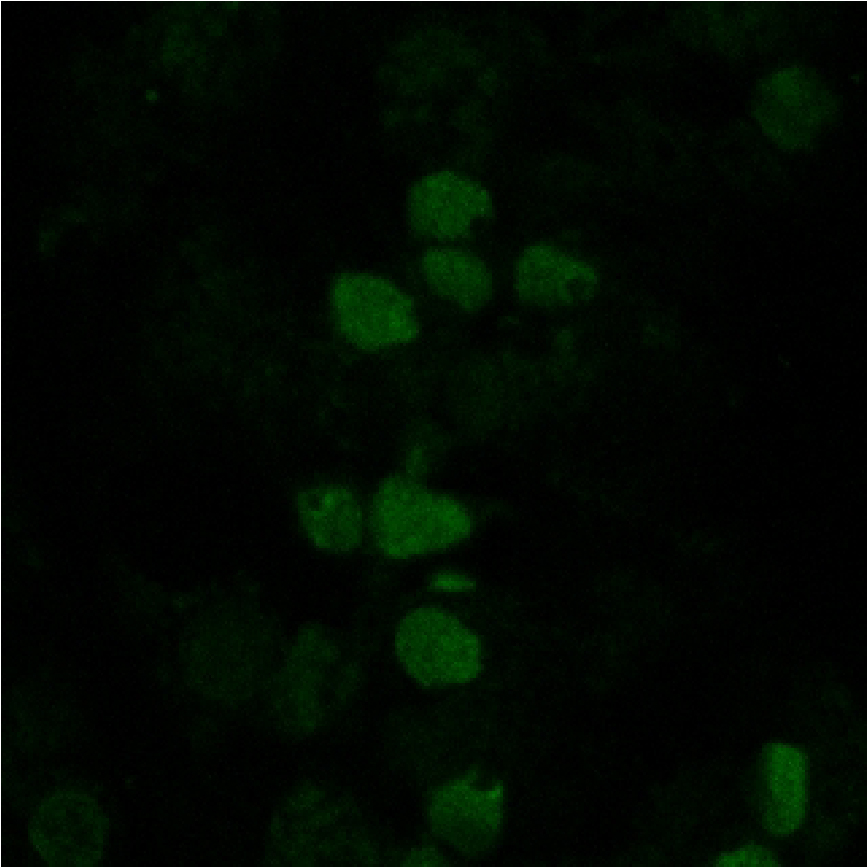

Supplement: Supplementary file 14 — Source data Fig. [file 44318_2025_659_MOESM14_ESM.zip › EMBOJ-2025-121587_Source Data/Source Data Figure 7/SD Figure 7D/Igf2bp3-/- siNC-1 H4Ac.jpg]

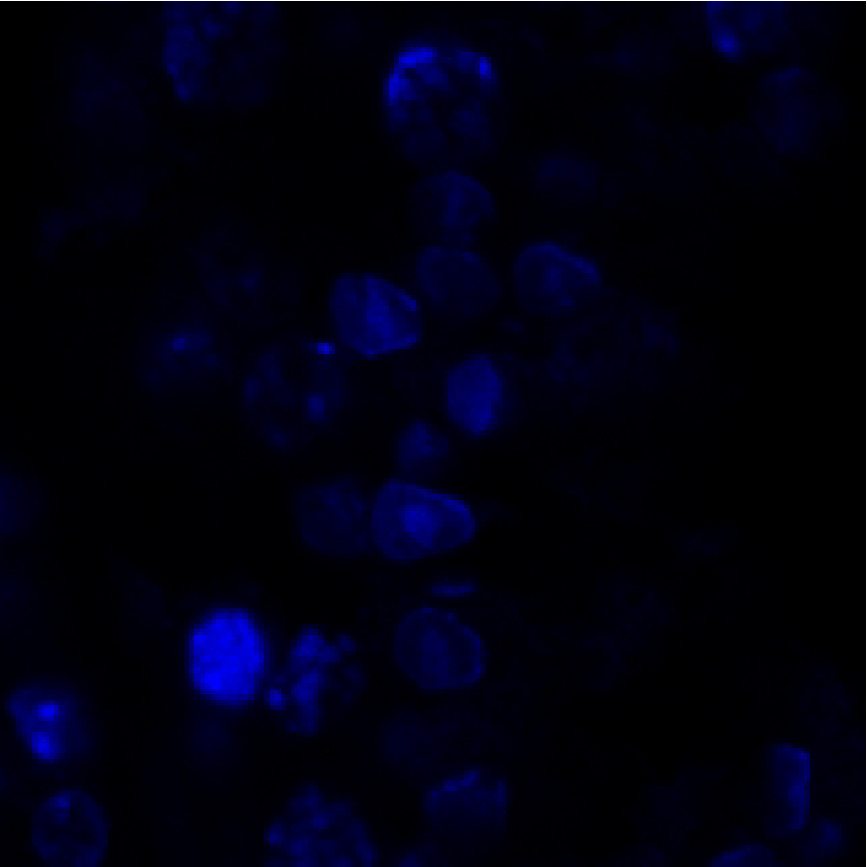

Supplement: Supplementary file 14 — Source data Fig. [file 44318_2025_659_MOESM14_ESM.zip › EMBOJ-2025-121587_Source Data/Source Data Figure 7/SD Figure 7D/Igf2bp3-/- siNC-1 Hoechst.jpg]

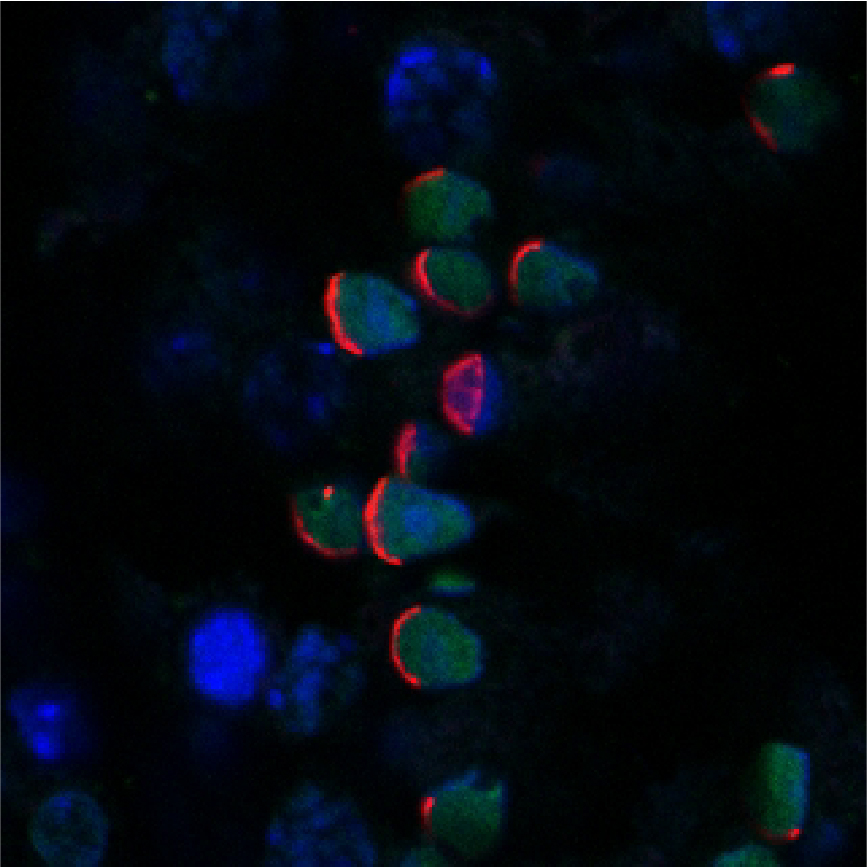

Supplement: Supplementary file 14 — Source data Fig. [file 44318_2025_659_MOESM14_ESM.zip › EMBOJ-2025-121587_Source Data/Source Data Figure 7/SD Figure 7D/Igf2bp3-/- siNC-1 Merge.jpg]

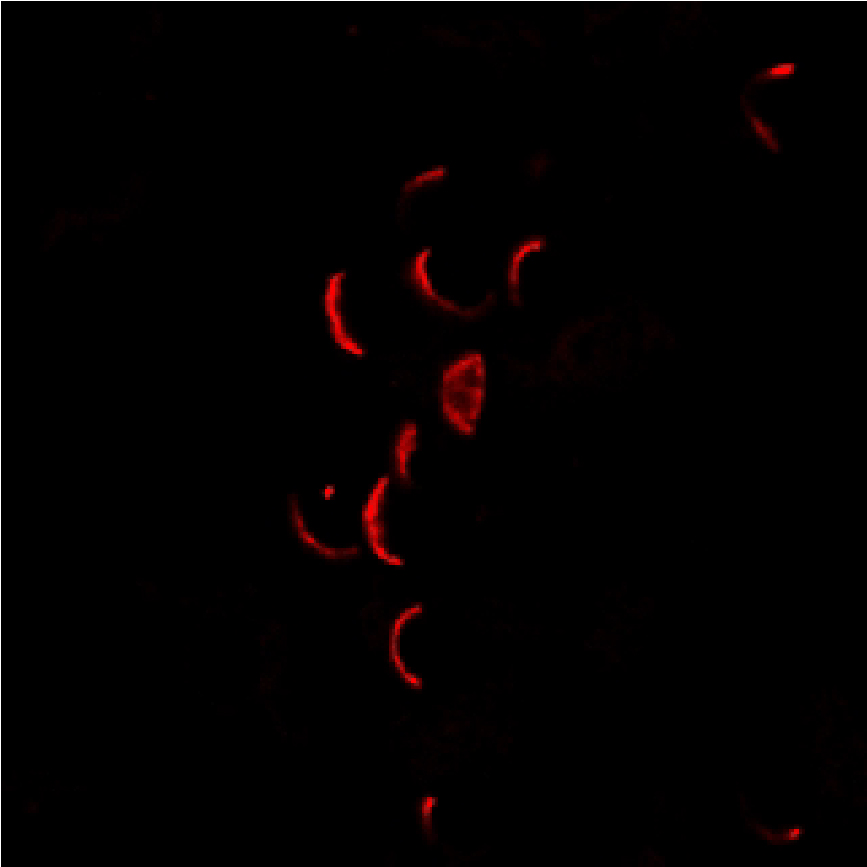

Supplement: Supplementary file 14 — Source data Fig. [file 44318_2025_659_MOESM14_ESM.zip › EMBOJ-2025-121587_Source Data/Source Data Figure 7/SD Figure 7D/Igf2bp3-/- siNC-1 PNA.jpg]

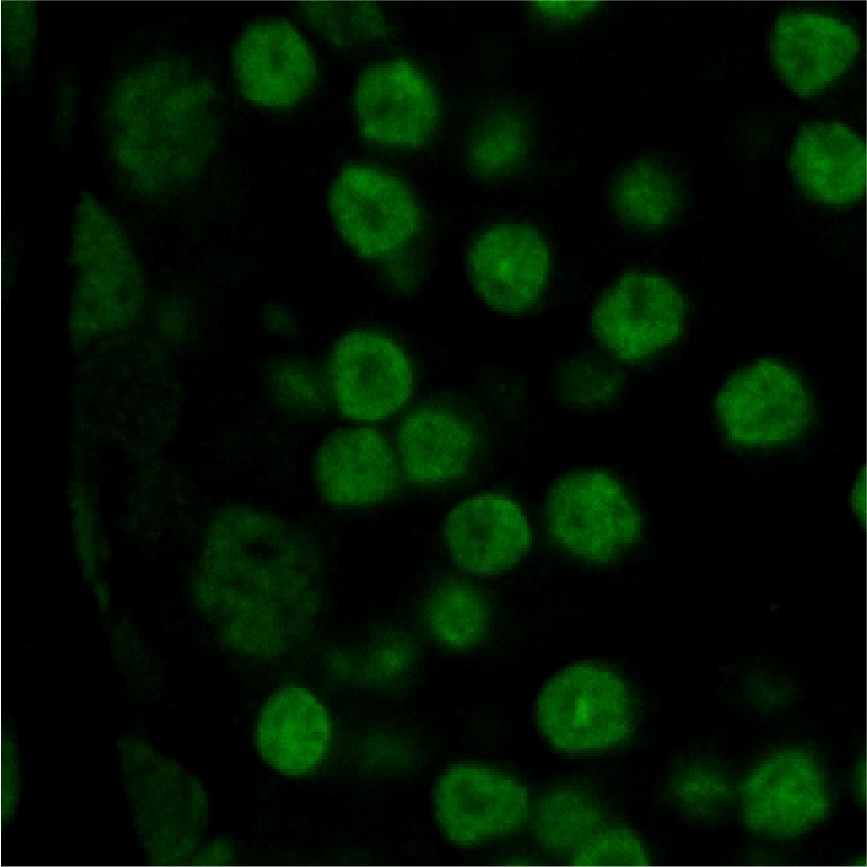

Supplement: Supplementary file 14 — Source data Fig. [file 44318_2025_659_MOESM14_ESM.zip › EMBOJ-2025-121587_Source Data/Source Data Figure 7/SD Figure 7E/Igf2bp3+/- siNC-1 H3K79me2.jpg]

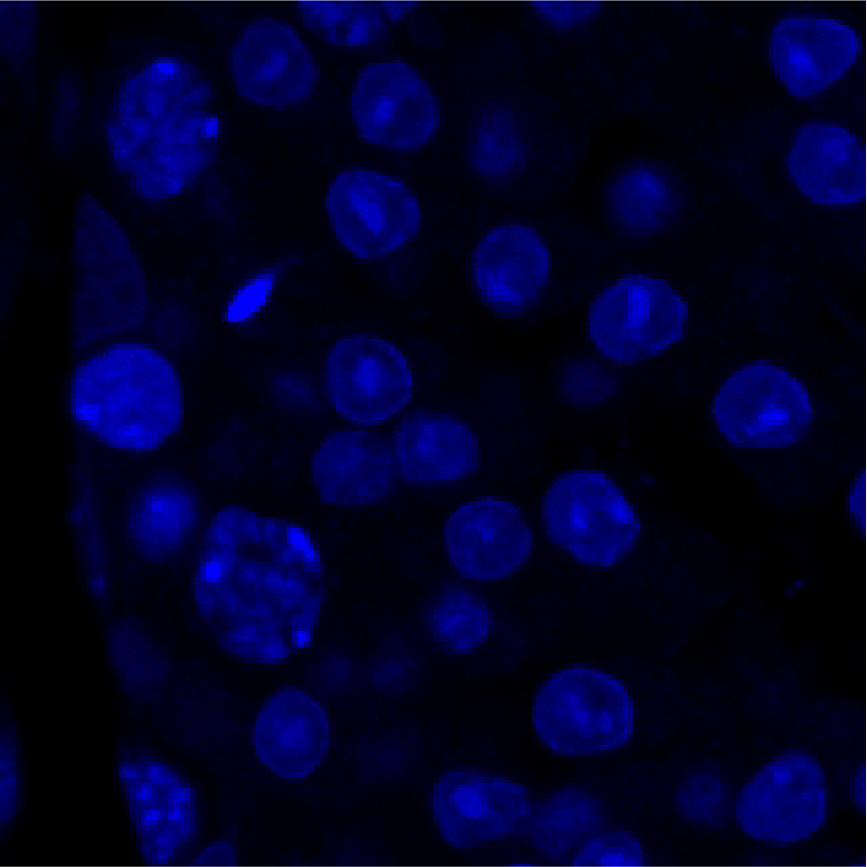

Supplement: Supplementary file 14 — Source data Fig. [file 44318_2025_659_MOESM14_ESM.zip › EMBOJ-2025-121587_Source Data/Source Data Figure 7/SD Figure 7E/Igf2bp3+/- siNC-1 Hoechst.jpg]

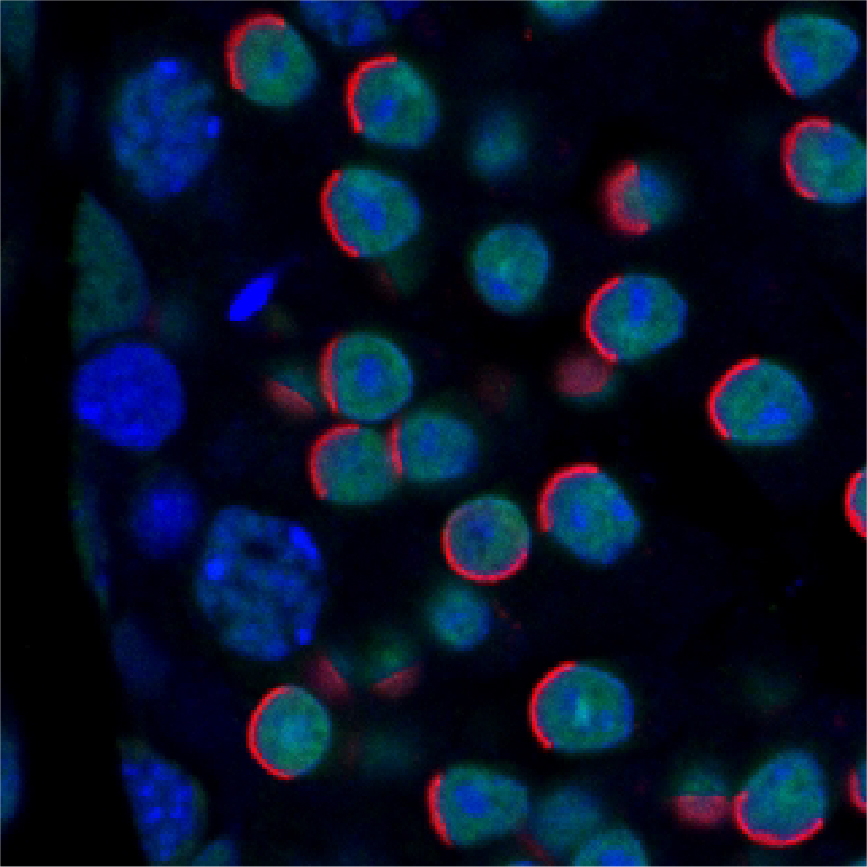

Supplement: Supplementary file 14 — Source data Fig. [file 44318_2025_659_MOESM14_ESM.zip › EMBOJ-2025-121587_Source Data/Source Data Figure 7/SD Figure 7E/Igf2bp3+/- siNC-1 Merge.jpg]

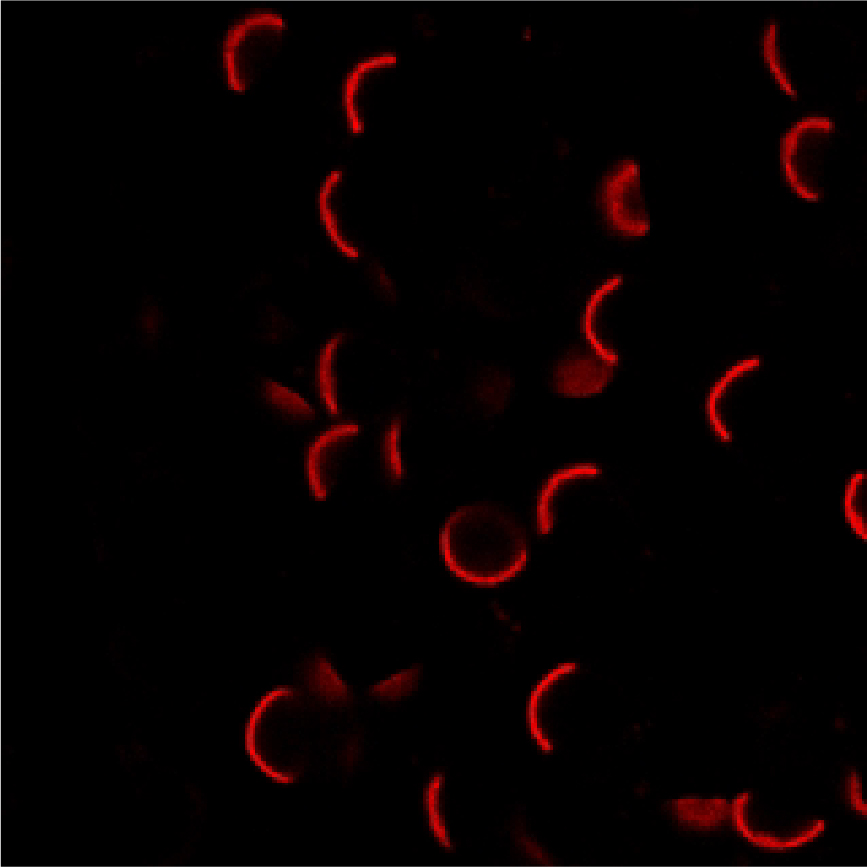

Supplement: Supplementary file 21 — Source data Fig. 7 [file 44318_2025_659_MOESM21_ESM.zip › Source Data Figure 7/SD Figure 7E/Igf2bp3+/- siNC-1 PNA.jpg]

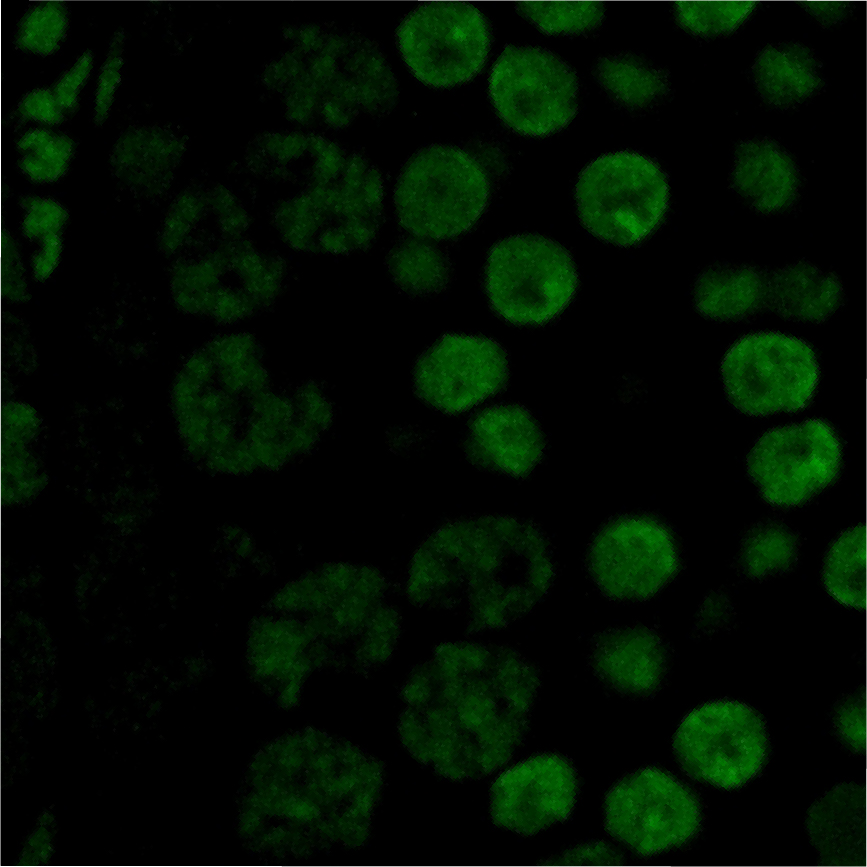

Supplement: Supplementary file 21 — Source data Fig. 7 [file 44318_2025_659_MOESM21_ESM.zip › Source Data Figure 7/SD Figure 7E/Igf2bp3-/- siDot1l/Hdac11-1 H3K79me2.jpg]

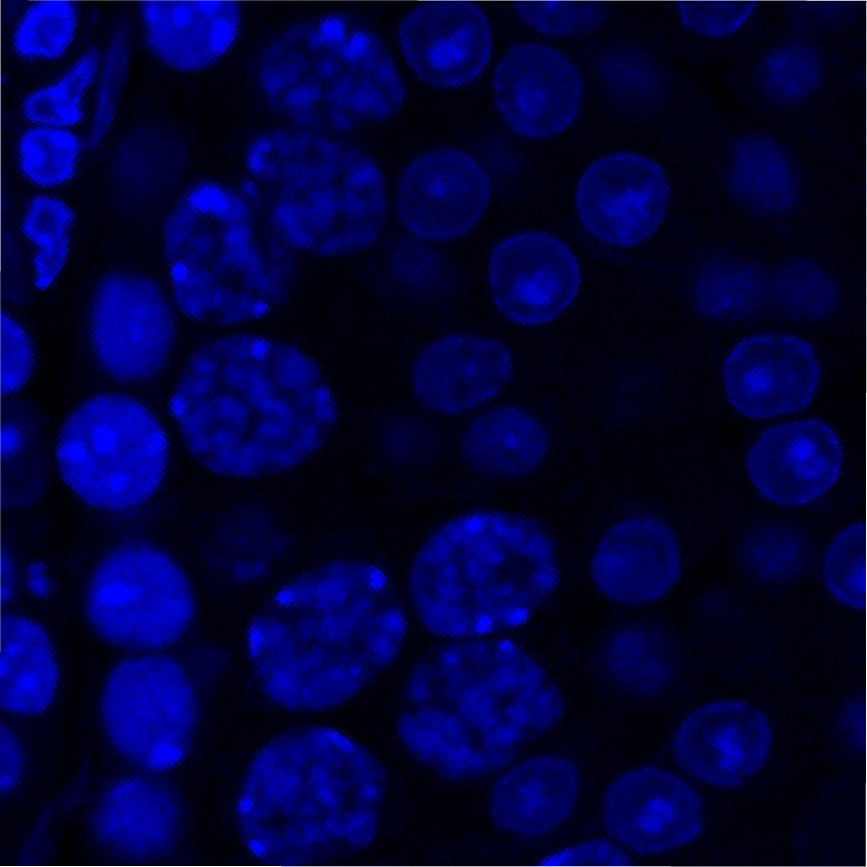

Supplement: Supplementary file 21 — Source data Fig. 7 [file 44318_2025_659_MOESM21_ESM.zip › Source Data Figure 7/SD Figure 7E/Igf2bp3-/- siDot1l/Hdac11-1 Hoechst.jpg]

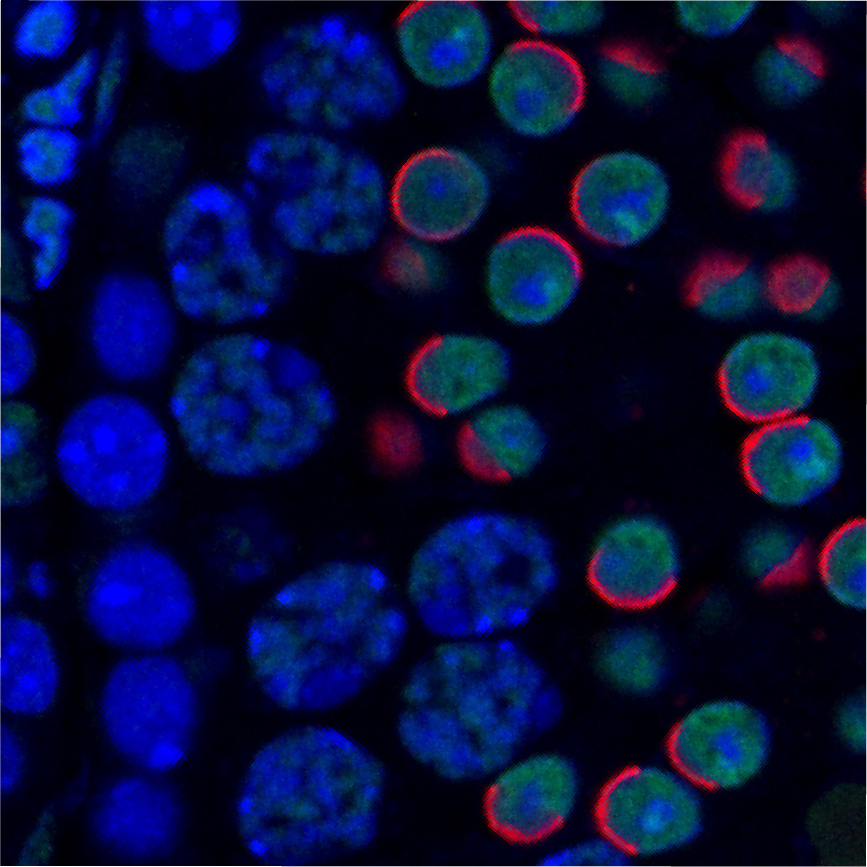

Supplement: Supplementary file 21 — Source data Fig. 7 [file 44318_2025_659_MOESM21_ESM.zip › Source Data Figure 7/SD Figure 7E/Igf2bp3-/- siDot1l/Hdac11-1 Merge.jpg]

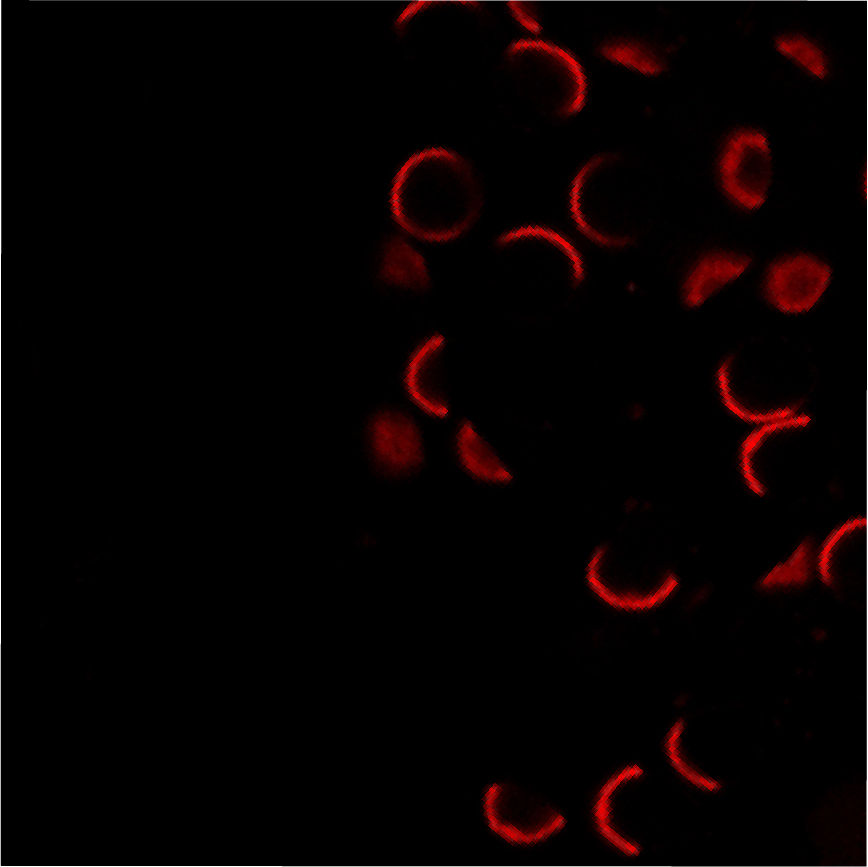

Supplement: Supplementary file 21 — Source data Fig. 7 [file 44318_2025_659_MOESM21_ESM.zip › Source Data Figure 7/SD Figure 7E/Igf2bp3-/- siDot1l/Hdac11-1 PNA.jpg]

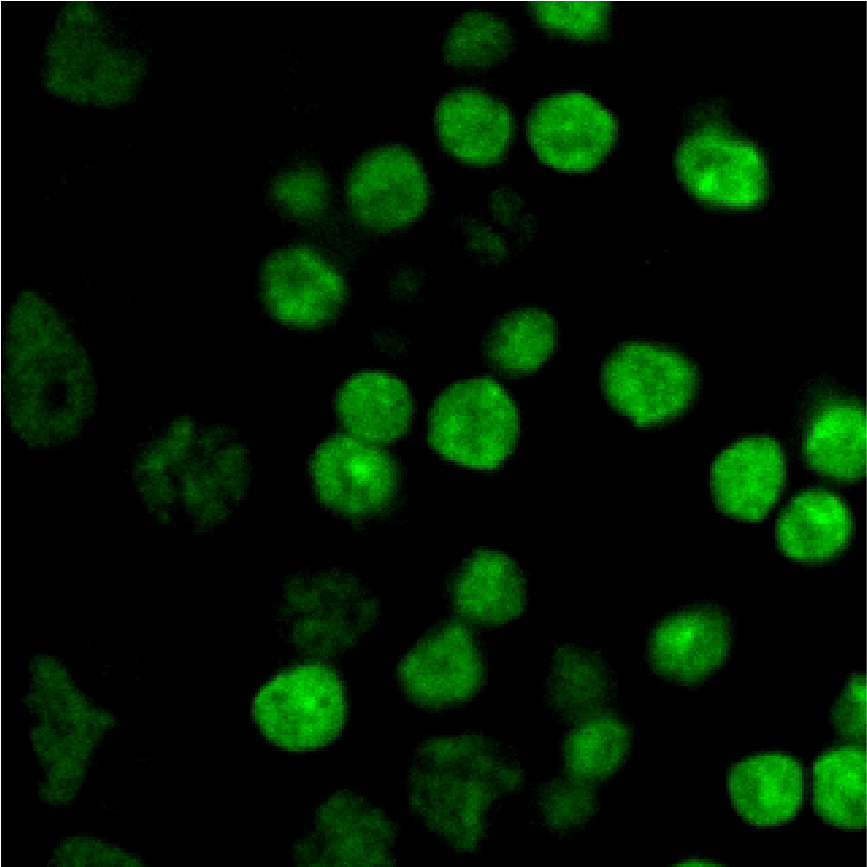

Supplement: Supplementary file 21 — Source data Fig. 7 [file 44318_2025_659_MOESM21_ESM.zip › Source Data Figure 7/SD Figure 7E/Igf2bp3-/- siNC-1 H3K79me2.jpg]

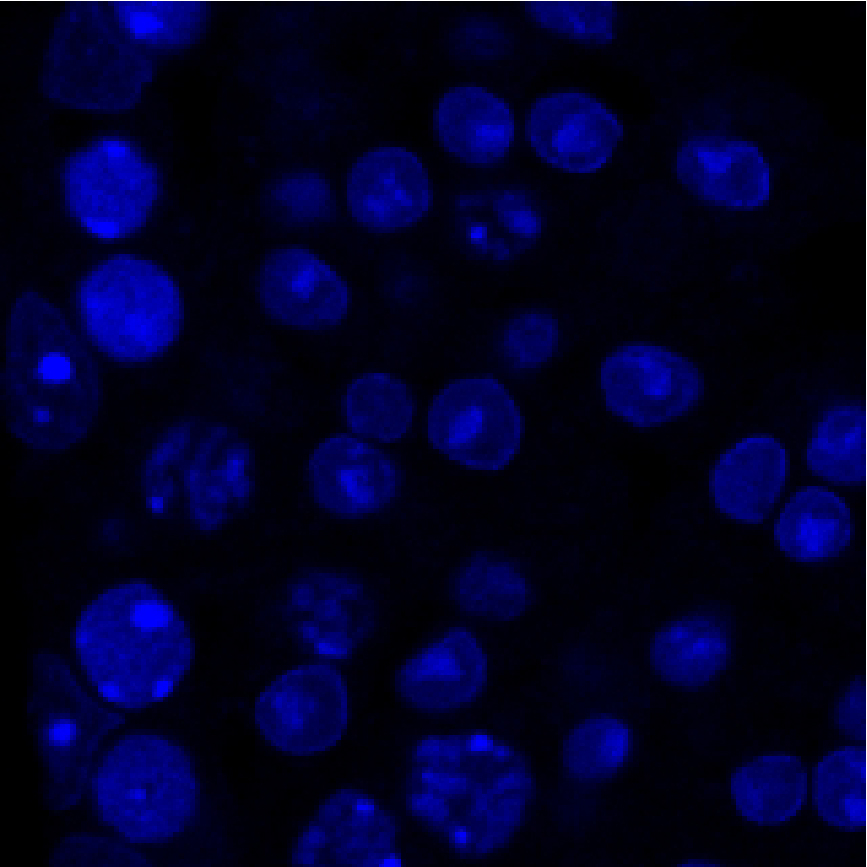

Supplement: Supplementary file 21 — Source data Fig. 7 [file 44318_2025_659_MOESM21_ESM.zip › Source Data Figure 7/SD Figure 7E/Igf2bp3-/- siNC-1 Hoechst.jpg]

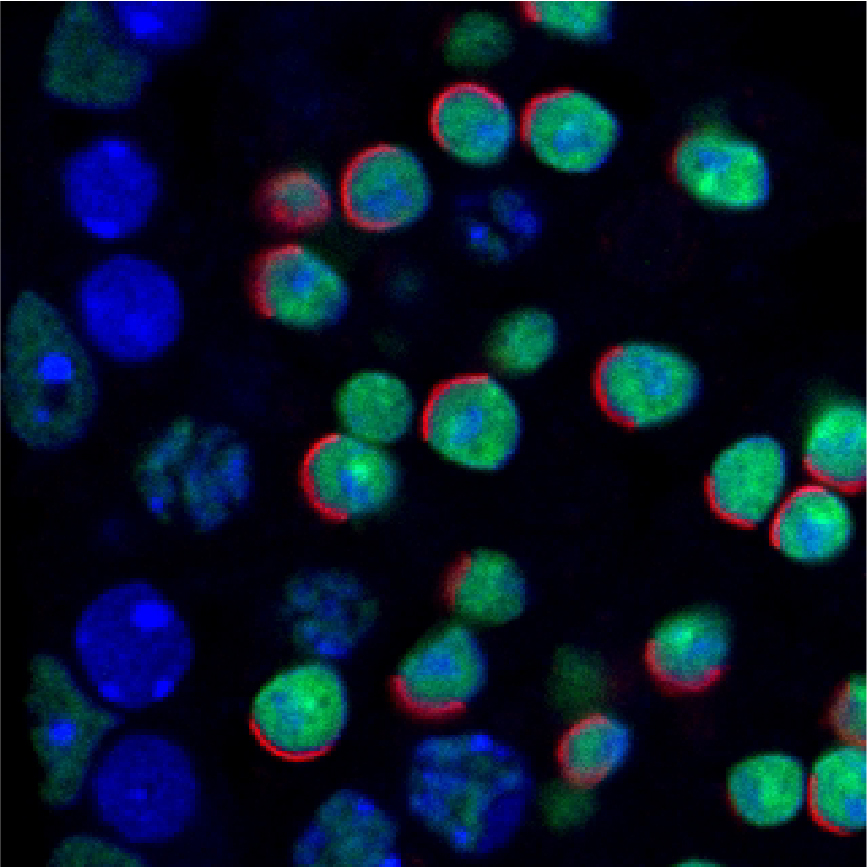

Supplement: Supplementary file 21 — Source data Fig. 7 [file 44318_2025_659_MOESM21_ESM.zip › Source Data Figure 7/SD Figure 7E/Igf2bp3-/- siNC-1 Merge.jpg]

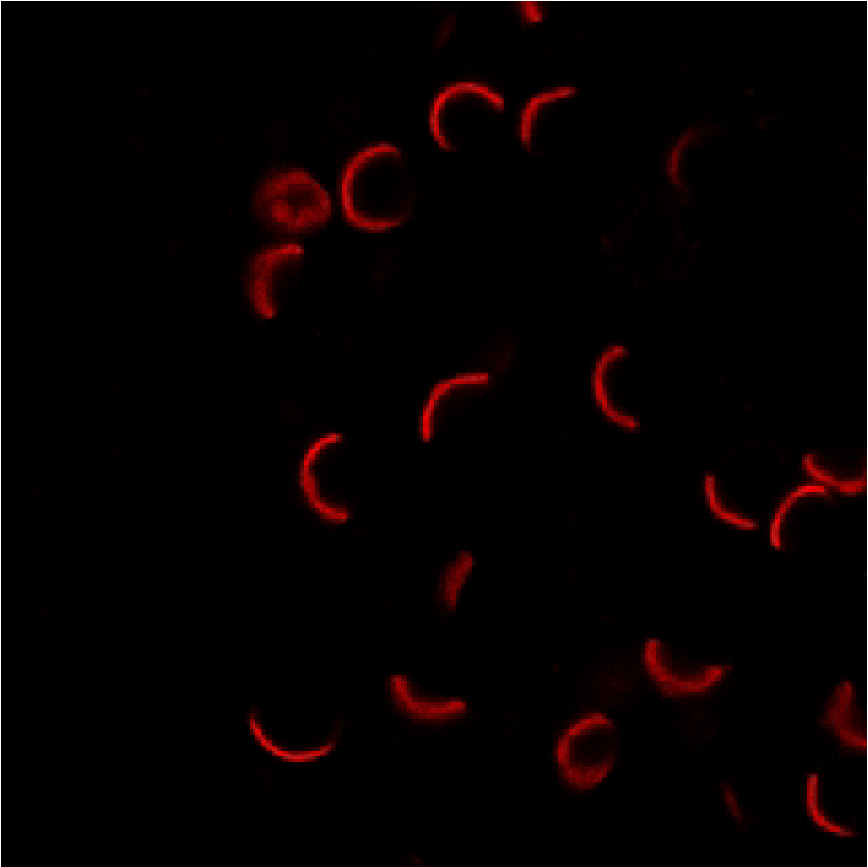

Supplement: Supplementary file 21 — Source data Fig. 7 [file 44318_2025_659_MOESM21_ESM.zip › Source Data Figure 7/SD Figure 7E/Igf2bp3-/- siNC-1 PNA.jpg]
