# Supplementary figures and images for: IGF2BP3 recognizes m6A to regulate histone-to-protamine replacement during mouse sperm development (part 1 of 3)
Source: EMBO J. 2025 Dec 5;45(2):504–36. doi: 10.1038/s44318-025-00659-y (PMC12811620; doi:10.1038/s44318-025-00659-y)

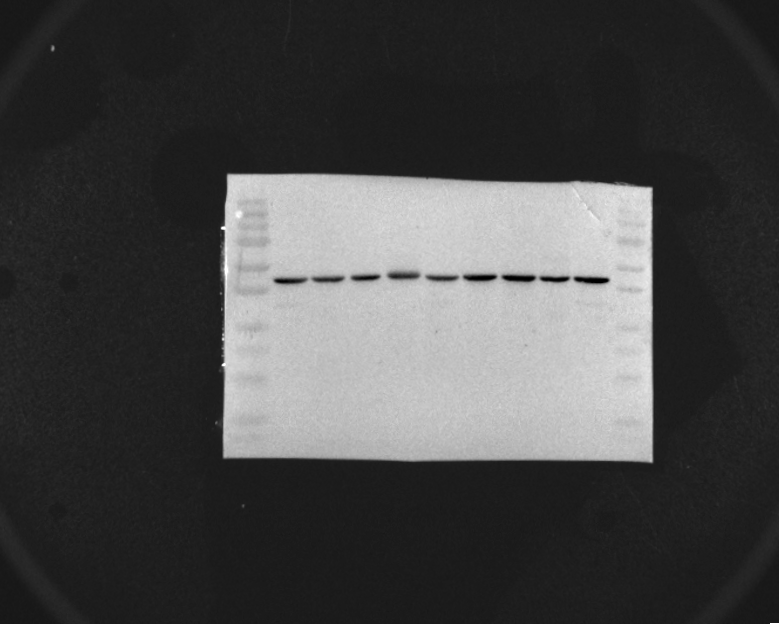

Supplement: Supplementary file 14 — Source data Fig. [file 44318_2025_659_MOESM14_ESM.zip › EMBOJ-2025-121587_Source Data/Source Data Figure 1/SD Figure 1A/SD Figure 1A-ACTB.tif]

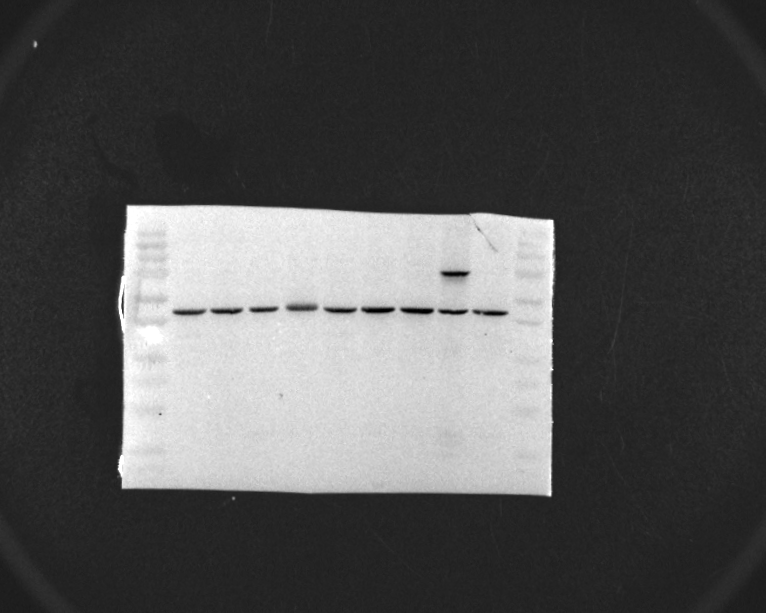

Supplement: Supplementary file 14 — Source data Fig. [file 44318_2025_659_MOESM14_ESM.zip › EMBOJ-2025-121587_Source Data/Source Data Figure 1/SD Figure 1A/SD Figure 1A-IGF2BP3.tif]

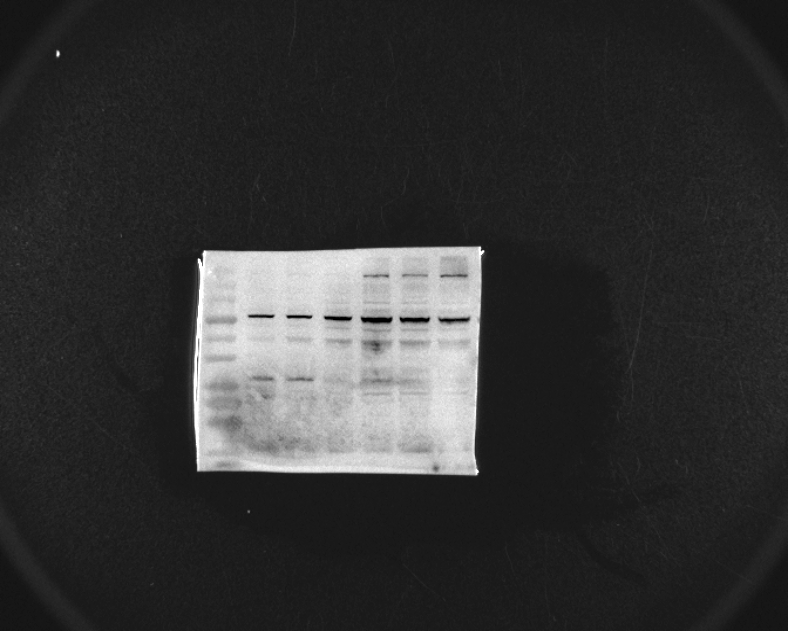

Supplement: Supplementary file 14 — Source data Fig. [file 44318_2025_659_MOESM14_ESM.zip › EMBOJ-2025-121587_Source Data/Source Data Figure 1/SD Figure 1B/F1B-1.tif]

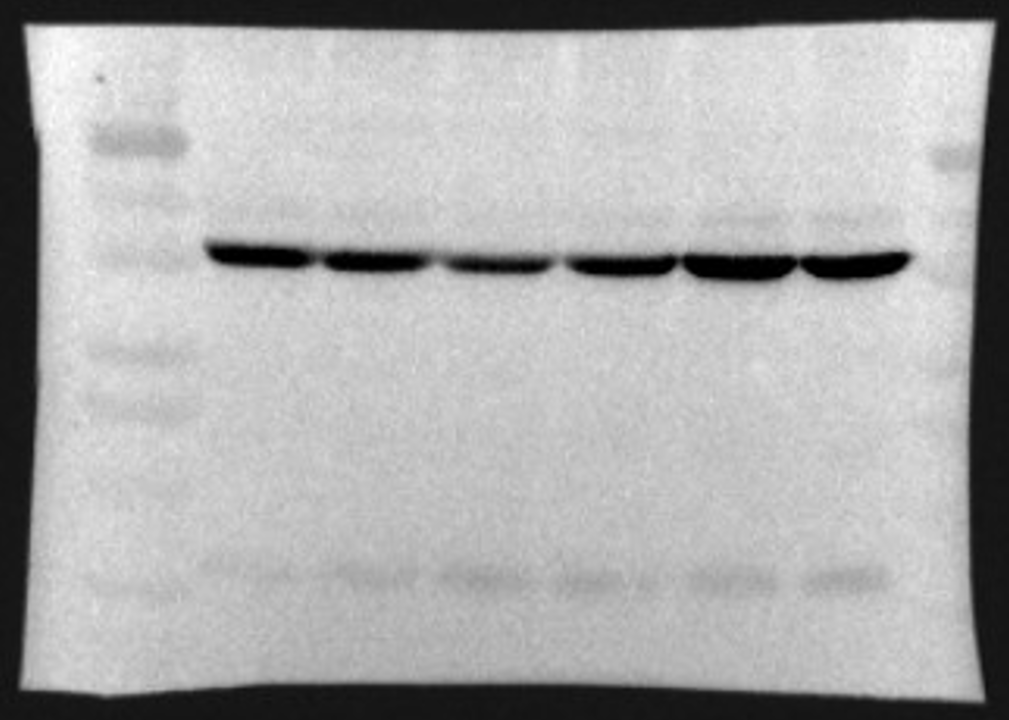

Supplement: Supplementary file 14 — Source data Fig. [file 44318_2025_659_MOESM14_ESM.zip › EMBOJ-2025-121587_Source Data/Source Data Figure 1/SD Figure 1B/F1B-ACTB.png]

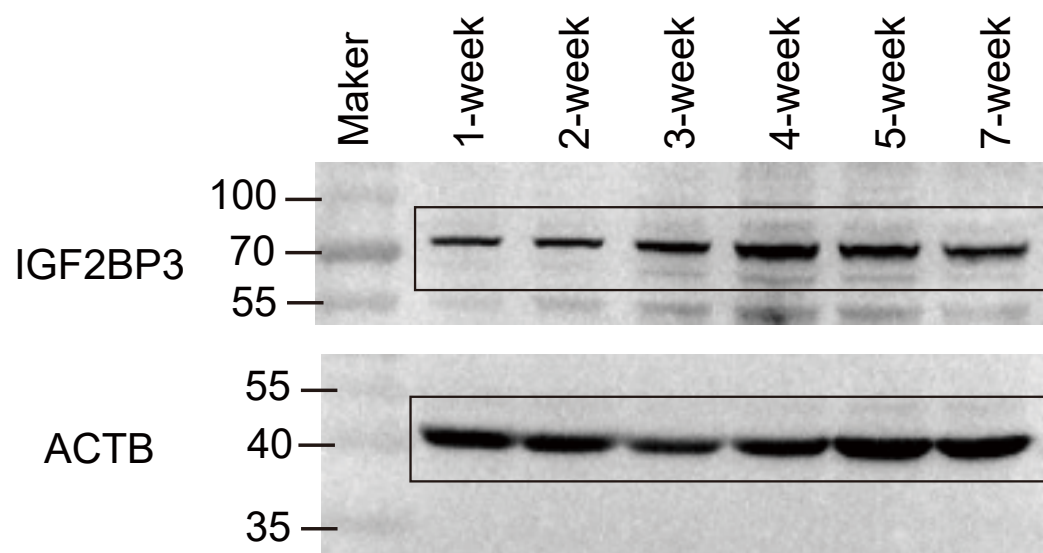

Supplement: Supplementary file 14 — Source data Fig. [file 44318_2025_659_MOESM14_ESM.zip › EMBOJ-2025-121587_Source Data/Source Data Figure 1/SD Figure 1B/SD Figure 1B.pdf]

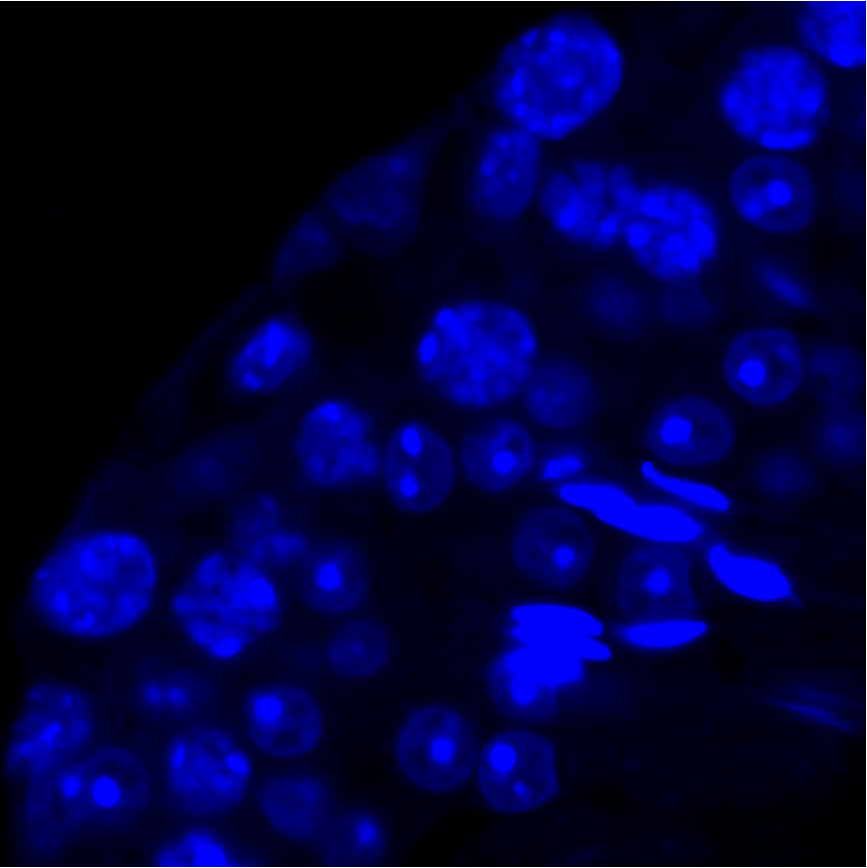

Supplement: Supplementary file 14 — Source data Fig. [file 44318_2025_659_MOESM14_ESM.zip › EMBOJ-2025-121587_Source Data/Source Data Figure 1/SD Figure 1C/Hoechst.jpg]

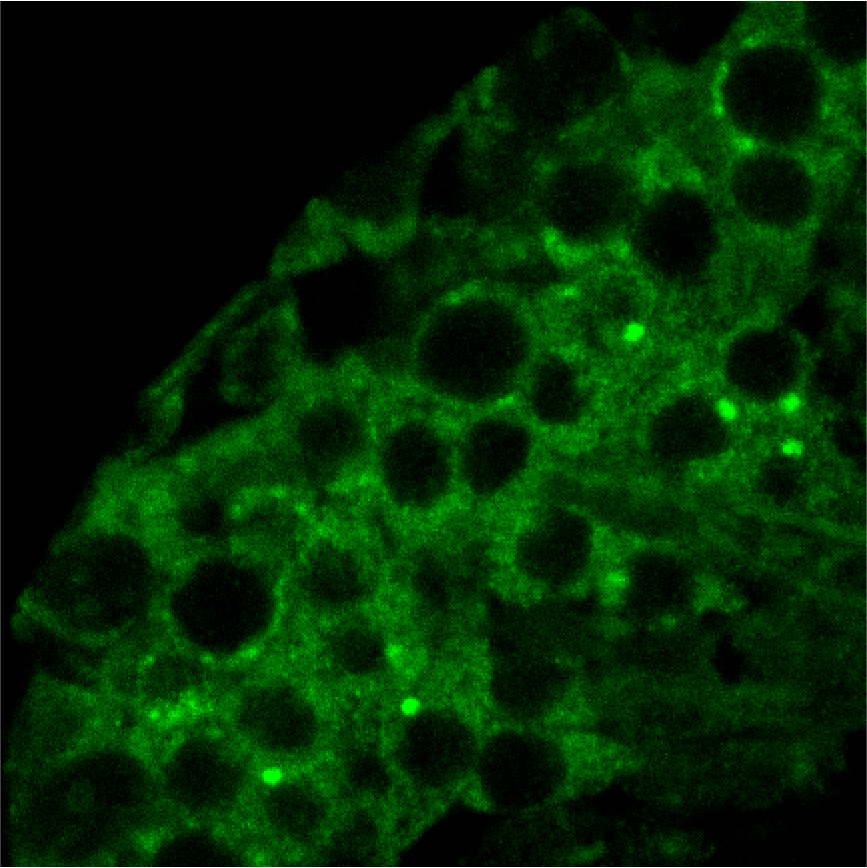

Supplement: Supplementary file 14 — Source data Fig. [file 44318_2025_659_MOESM14_ESM.zip › EMBOJ-2025-121587_Source Data/Source Data Figure 1/SD Figure 1C/IGF2BP3.jpg]

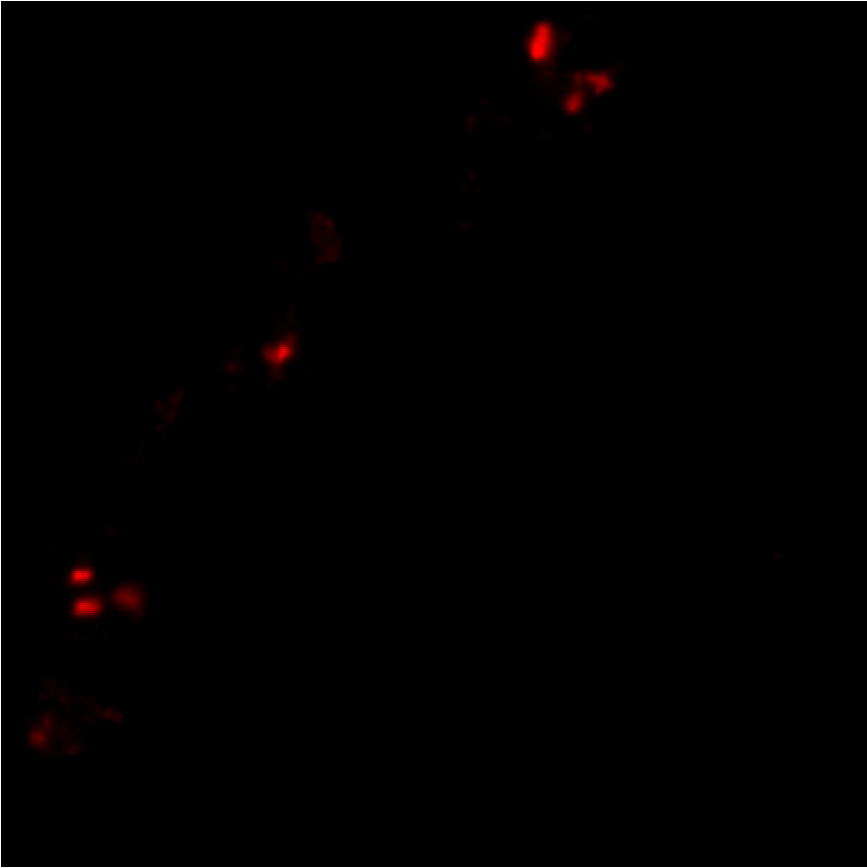

Supplement: Supplementary file 14 — Source data Fig. [file 44318_2025_659_MOESM14_ESM.zip › EMBOJ-2025-121587_Source Data/Source Data Figure 1/SD Figure 1C/LIN28A.jpg]

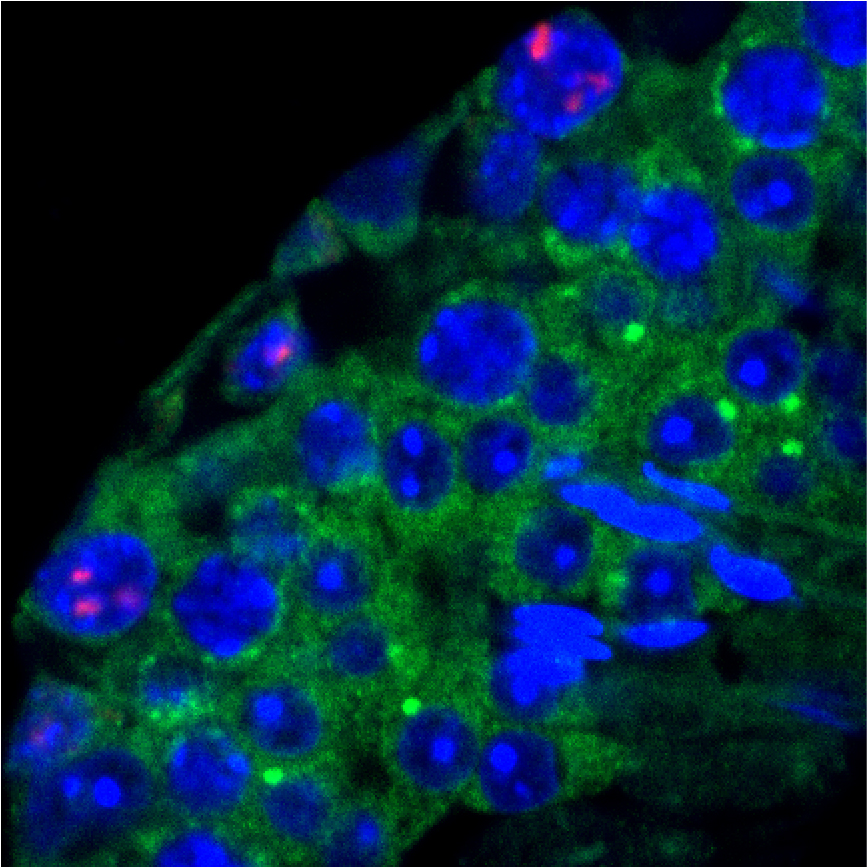

Supplement: Supplementary file 14 — Source data Fig. [file 44318_2025_659_MOESM14_ESM.zip › EMBOJ-2025-121587_Source Data/Source Data Figure 1/SD Figure 1C/Merge.jpg]

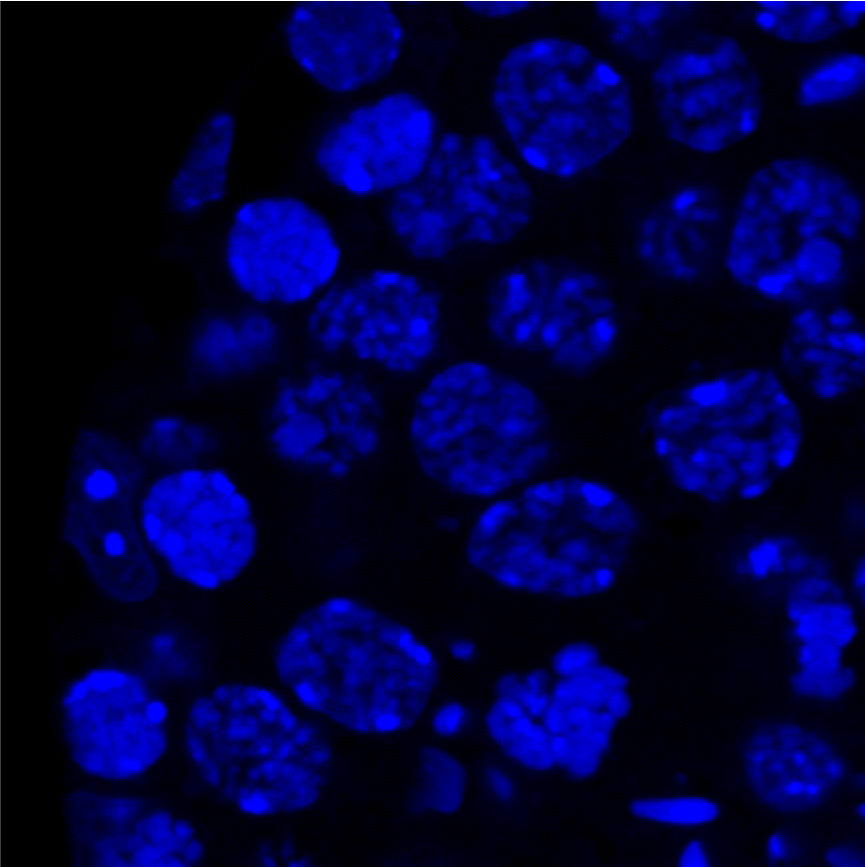

Supplement: Supplementary file 14 — Source data Fig. [file 44318_2025_659_MOESM14_ESM.zip › EMBOJ-2025-121587_Source Data/Source Data Figure 1/SD Figure 1D/Hoechst.jpg]

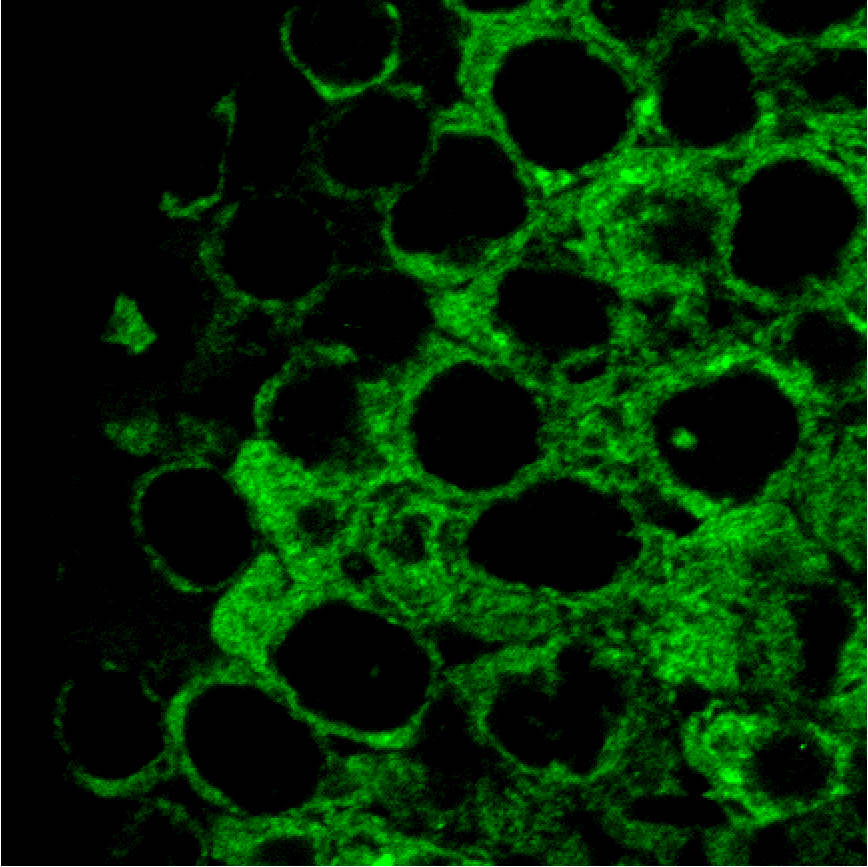

Supplement: Supplementary file 14 — Source data Fig. [file 44318_2025_659_MOESM14_ESM.zip › EMBOJ-2025-121587_Source Data/Source Data Figure 1/SD Figure 1D/IGF2BP3.jpg]

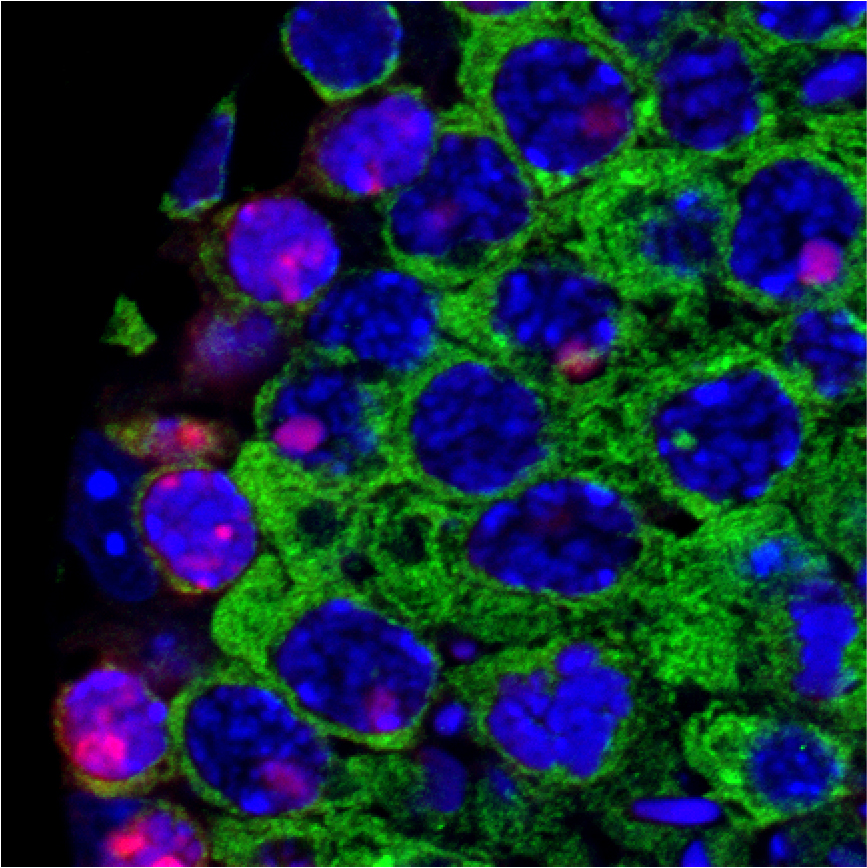

Supplement: Supplementary file 14 — Source data Fig. [file 44318_2025_659_MOESM14_ESM.zip › EMBOJ-2025-121587_Source Data/Source Data Figure 1/SD Figure 1D/Merge.jpg]

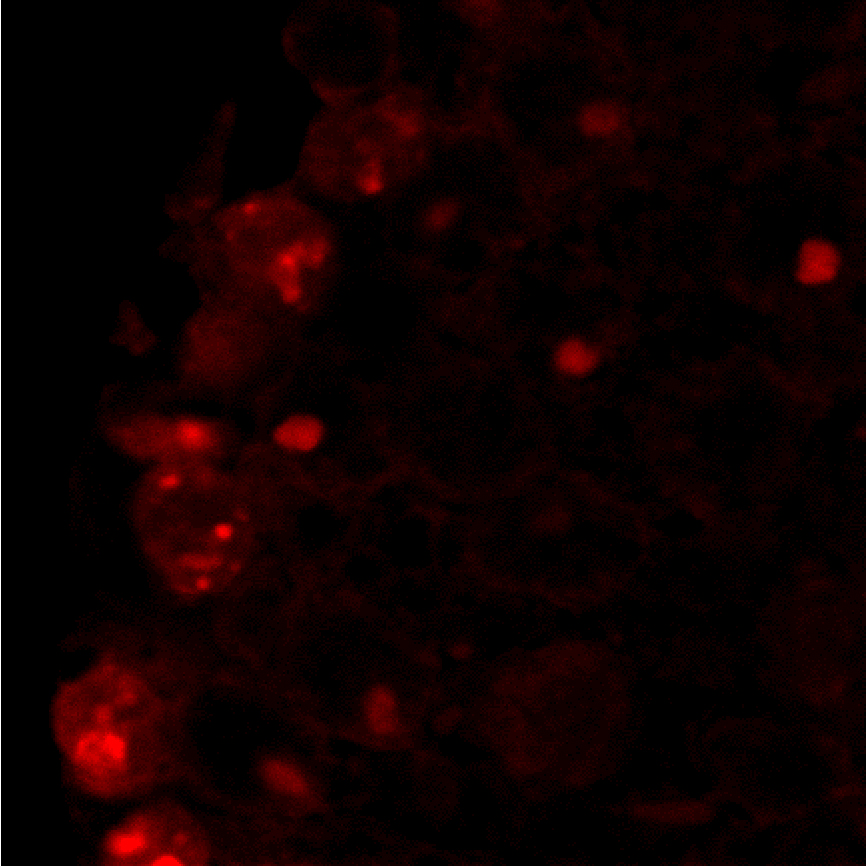

Supplement: Supplementary file 14 — Source data Fig. [file 44318_2025_659_MOESM14_ESM.zip › EMBOJ-2025-121587_Source Data/Source Data Figure 1/SD Figure 1D/γH2AX.jpg]

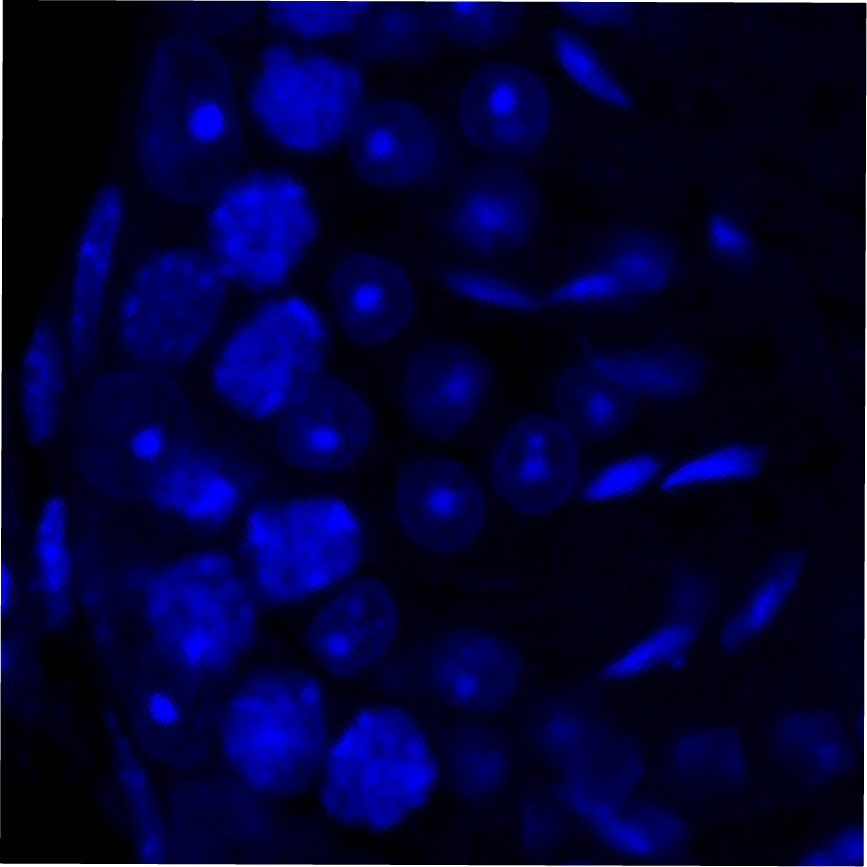

Supplement: Supplementary file 14 — Source data Fig. [file 44318_2025_659_MOESM14_ESM.zip › EMBOJ-2025-121587_Source Data/Source Data Figure 1/SD Figure 1E/Stage I Hoechst.jpg]

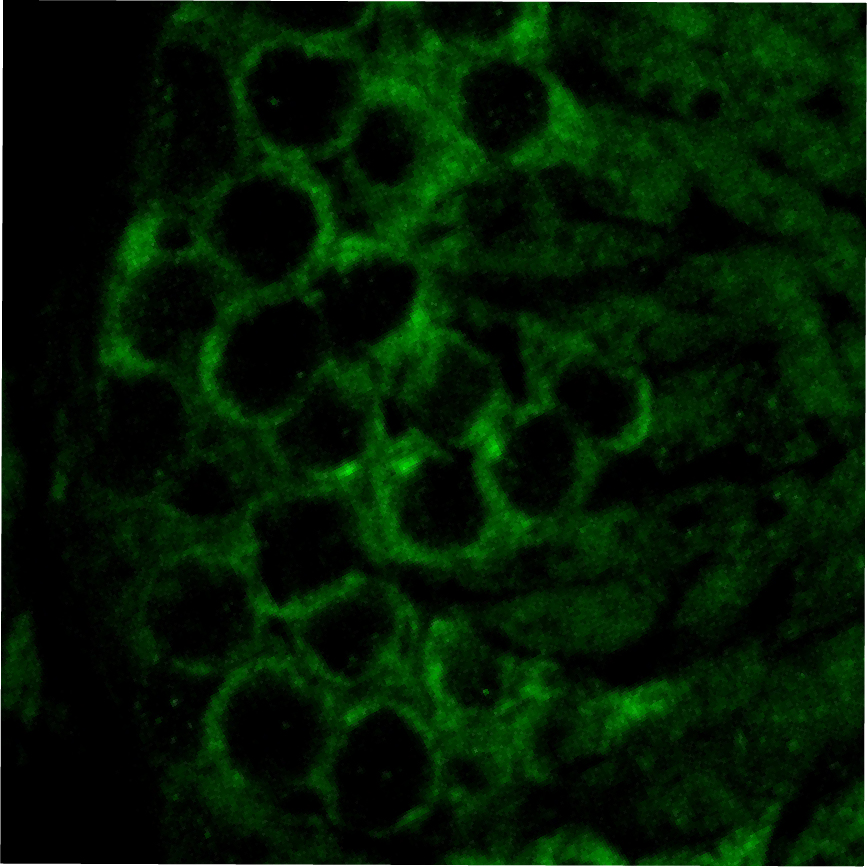

Supplement: Supplementary file 14 — Source data Fig. [file 44318_2025_659_MOESM14_ESM.zip › EMBOJ-2025-121587_Source Data/Source Data Figure 1/SD Figure 1E/Stage I IGF2BP3.jpg]

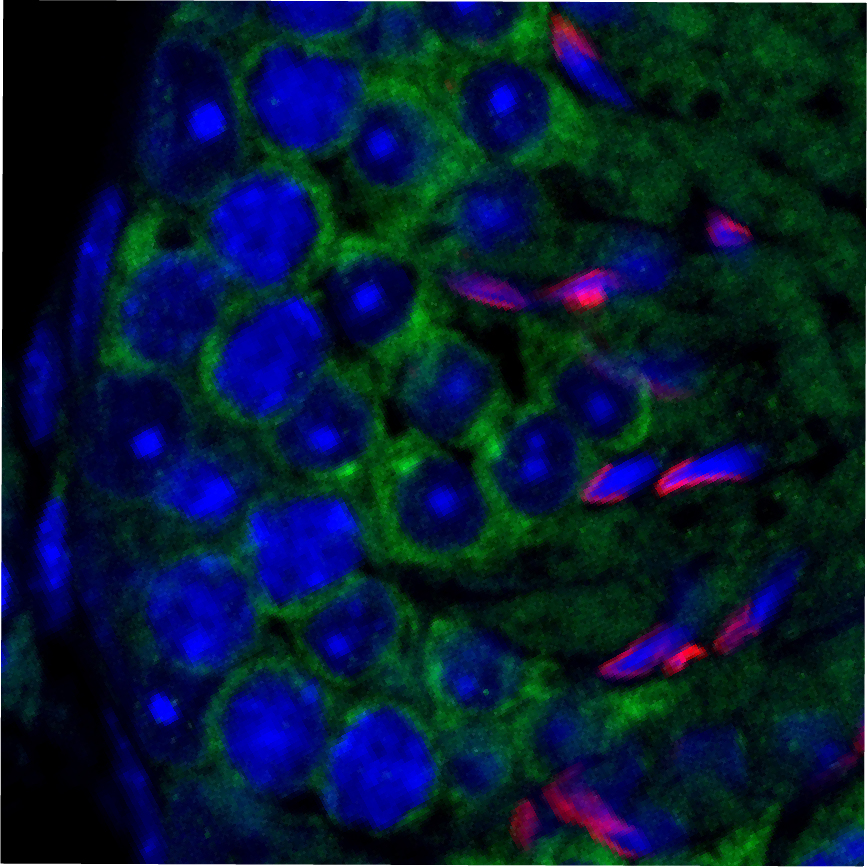

Supplement: Supplementary file 14 — Source data Fig. [file 44318_2025_659_MOESM14_ESM.zip › EMBOJ-2025-121587_Source Data/Source Data Figure 1/SD Figure 1E/Stage I Merge.jpg]

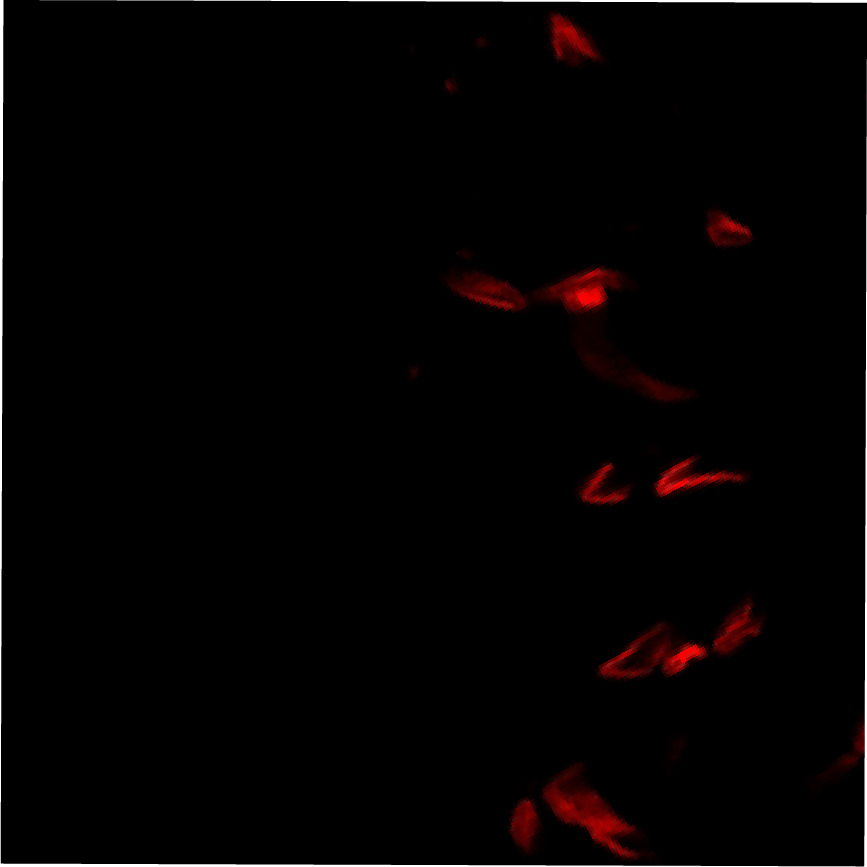

Supplement: Supplementary file 14 — Source data Fig. [file 44318_2025_659_MOESM14_ESM.zip › EMBOJ-2025-121587_Source Data/Source Data Figure 1/SD Figure 1E/Stage I PNA.jpg]

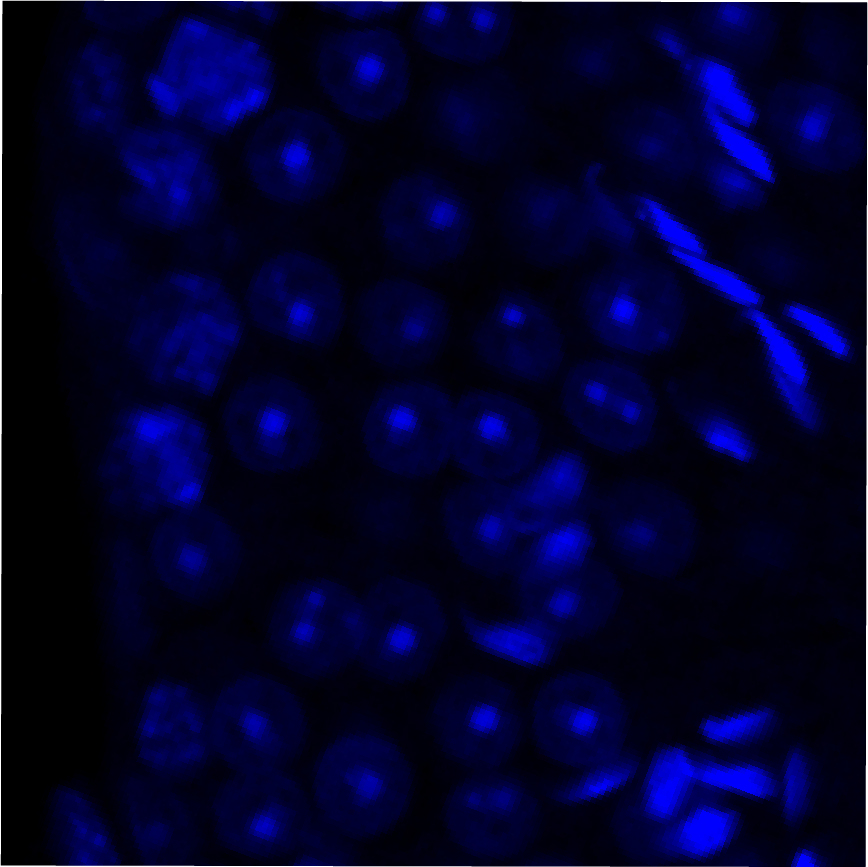

Supplement: Supplementary file 14 — Source data Fig. [file 44318_2025_659_MOESM14_ESM.zip › EMBOJ-2025-121587_Source Data/Source Data Figure 1/SD Figure 1E/Stage II-III Hoechst.jpg]

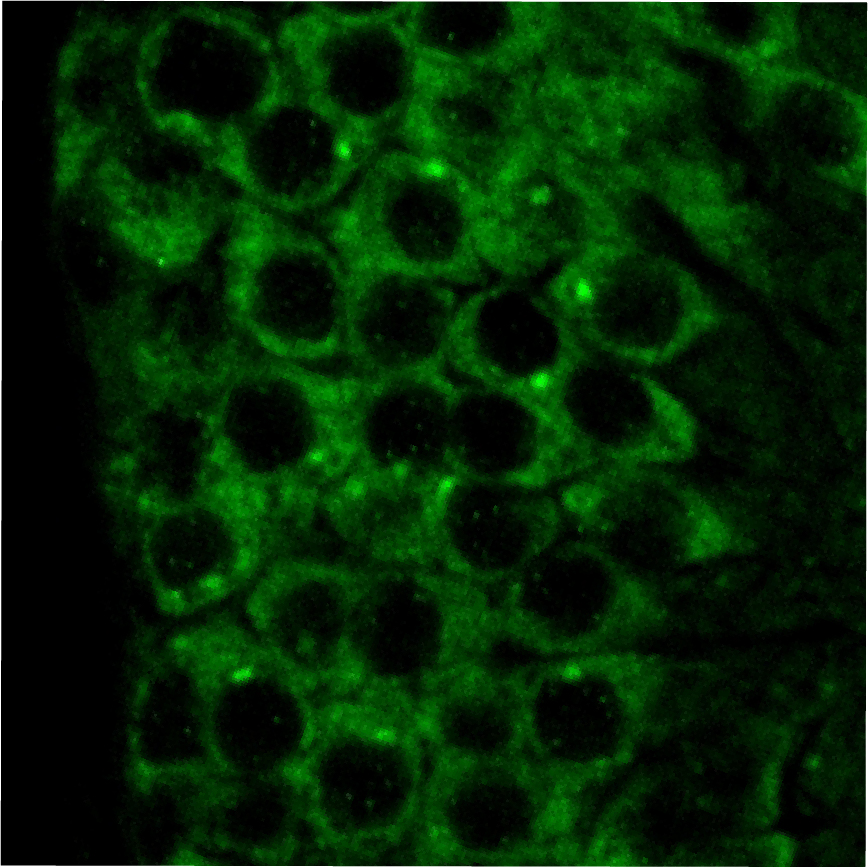

Supplement: Supplementary file 14 — Source data Fig. [file 44318_2025_659_MOESM14_ESM.zip › EMBOJ-2025-121587_Source Data/Source Data Figure 1/SD Figure 1E/Stage II-III IGF2BP3.jpg]

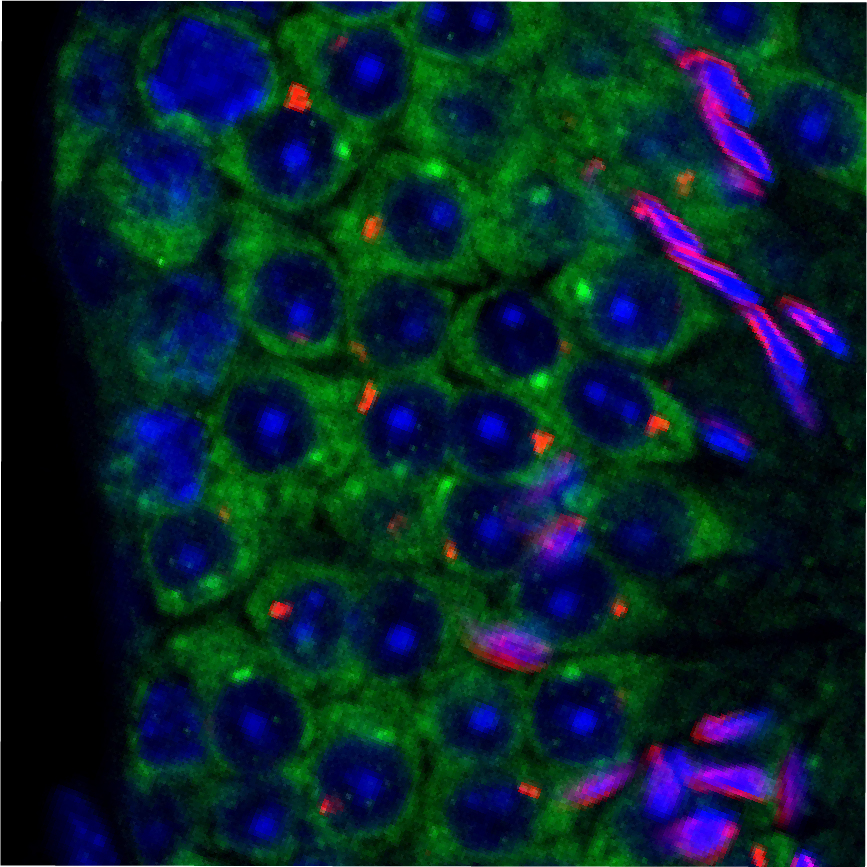

Supplement: Supplementary file 14 — Source data Fig. [file 44318_2025_659_MOESM14_ESM.zip › EMBOJ-2025-121587_Source Data/Source Data Figure 1/SD Figure 1E/Stage II-III Merge.jpg]

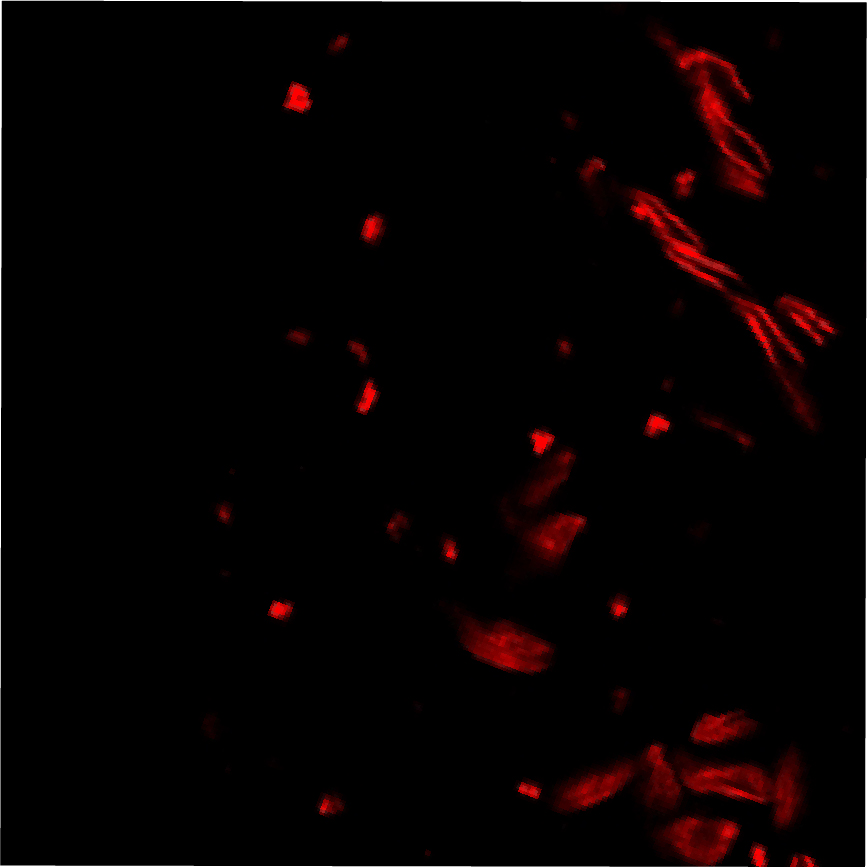

Supplement: Supplementary file 14 — Source data Fig. [file 44318_2025_659_MOESM14_ESM.zip › EMBOJ-2025-121587_Source Data/Source Data Figure 1/SD Figure 1E/Stage II-III PNA.jpg]

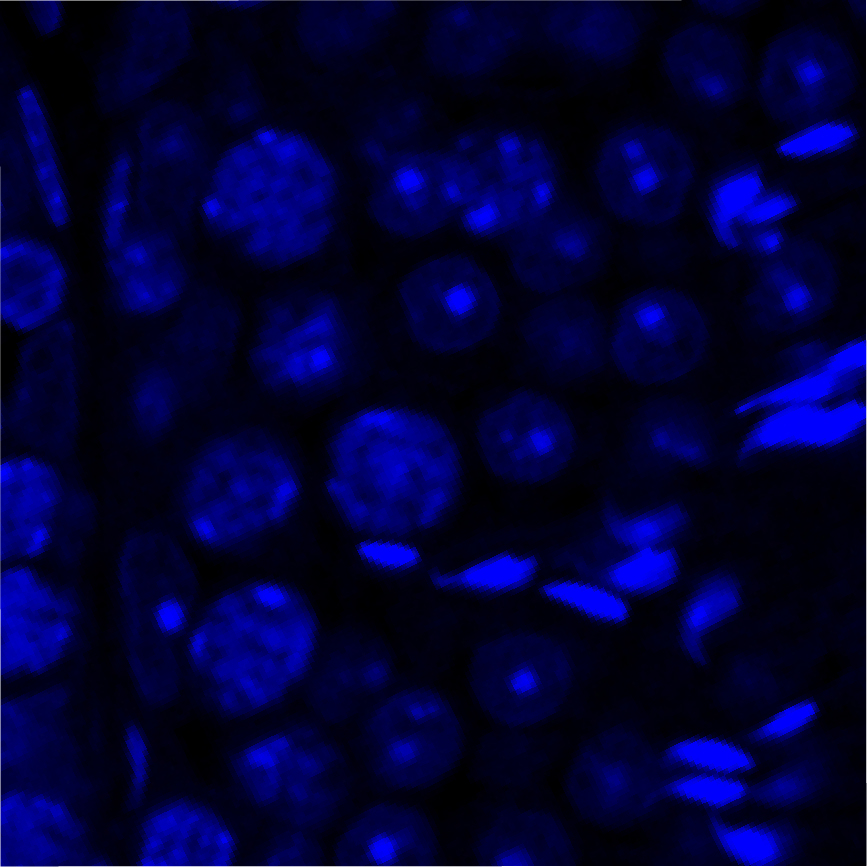

Supplement: Supplementary file 14 — Source data Fig. [file 44318_2025_659_MOESM14_ESM.zip › EMBOJ-2025-121587_Source Data/Source Data Figure 1/SD Figure 1E/Stage IV-VI Hoechst.jpg]

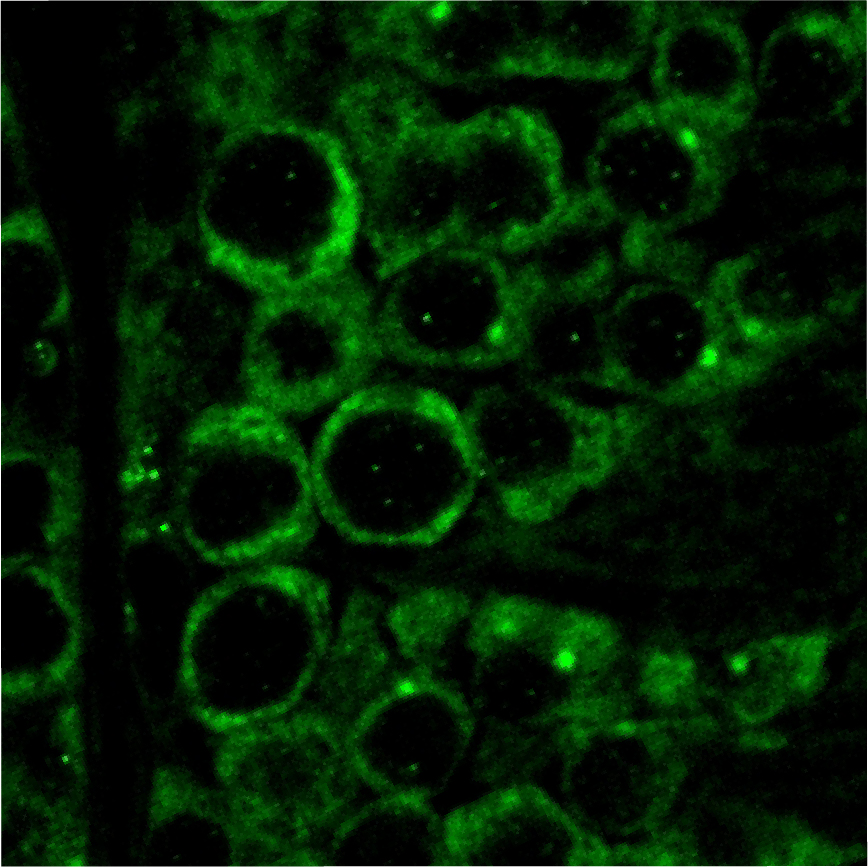

Supplement: Supplementary file 14 — Source data Fig. [file 44318_2025_659_MOESM14_ESM.zip › EMBOJ-2025-121587_Source Data/Source Data Figure 1/SD Figure 1E/Stage IV-VI IGF2BP3.jpg]

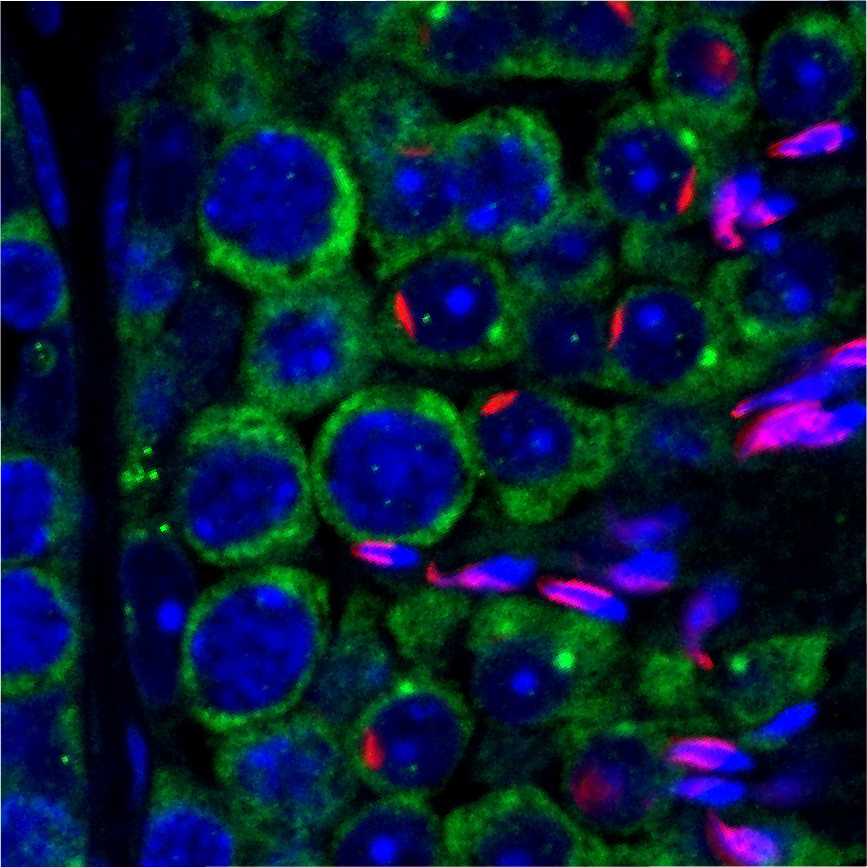

Supplement: Supplementary file 14 — Source data Fig. [file 44318_2025_659_MOESM14_ESM.zip › EMBOJ-2025-121587_Source Data/Source Data Figure 1/SD Figure 1E/Stage IV-VI Merge.jpg]

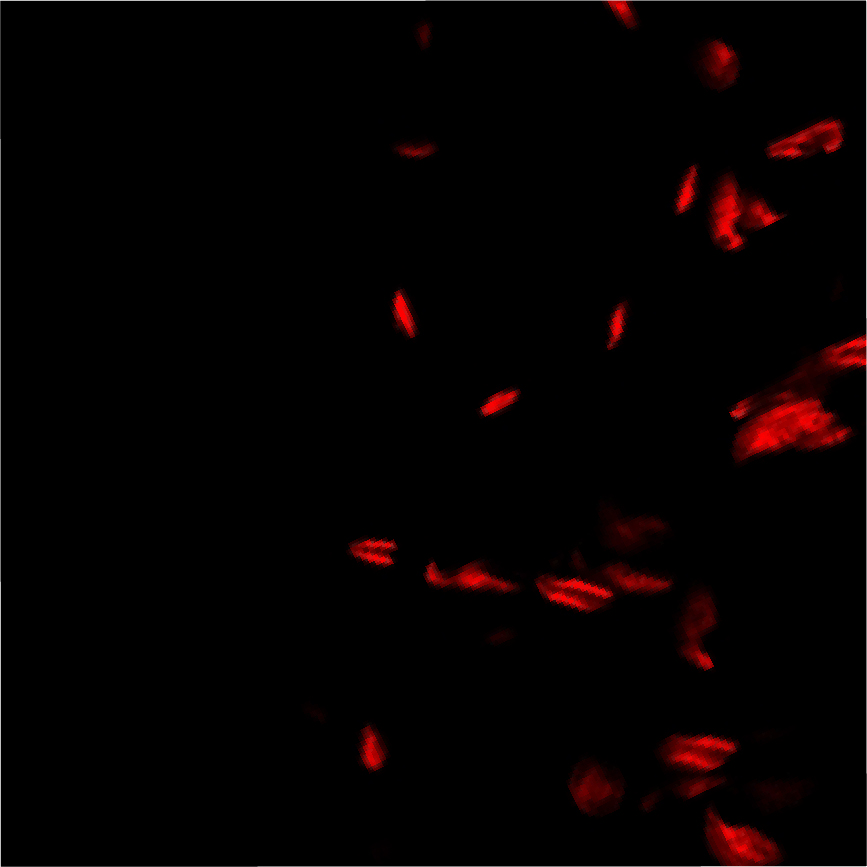

Supplement: Supplementary file 14 — Source data Fig. [file 44318_2025_659_MOESM14_ESM.zip › EMBOJ-2025-121587_Source Data/Source Data Figure 1/SD Figure 1E/Stage IV-VI PNA.jpg]

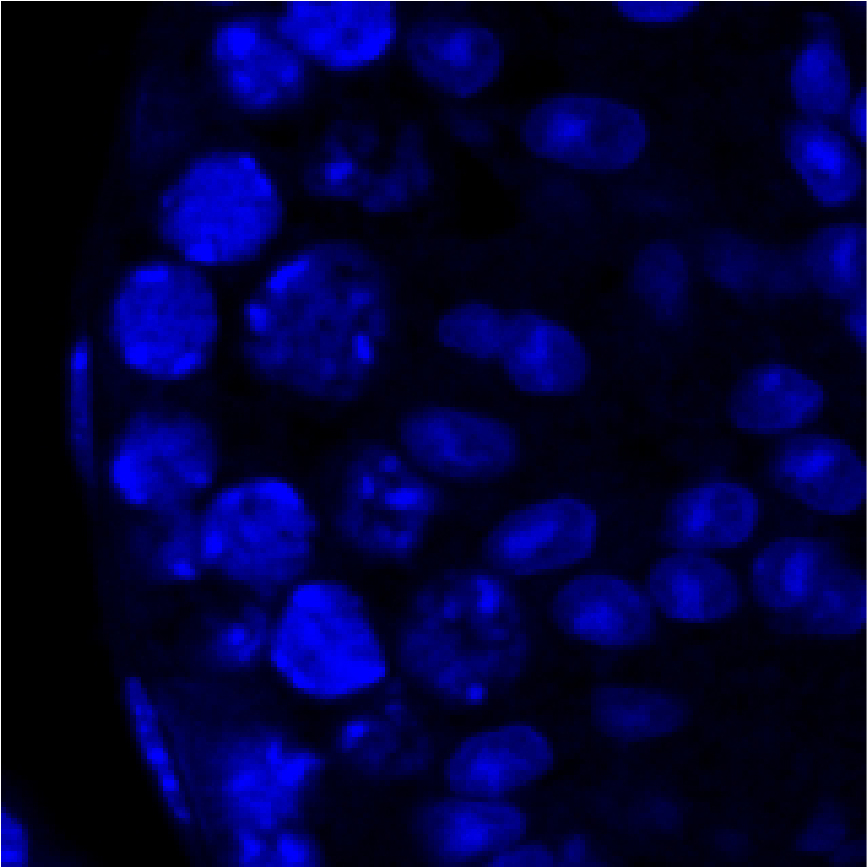

Supplement: Supplementary file 14 — Source data Fig. [file 44318_2025_659_MOESM14_ESM.zip › EMBOJ-2025-121587_Source Data/Source Data Figure 1/SD Figure 1E/Stage IX-X Hoechst.jpg]

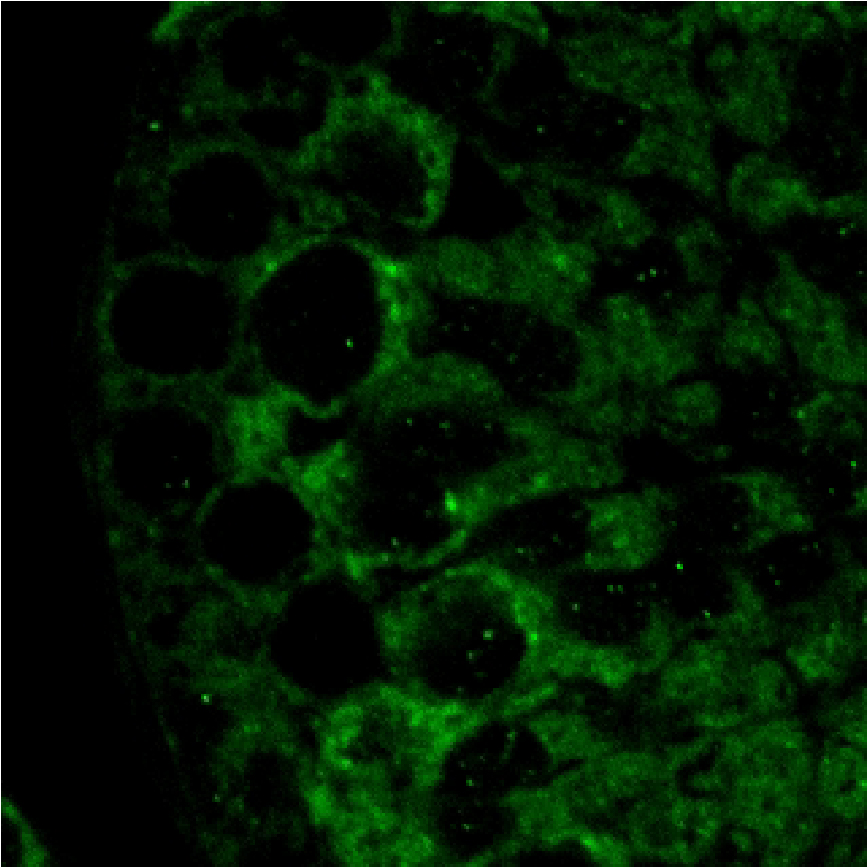

Supplement: Supplementary file 14 — Source data Fig. [file 44318_2025_659_MOESM14_ESM.zip › EMBOJ-2025-121587_Source Data/Source Data Figure 1/SD Figure 1E/Stage IX-X IGF2BP3 .jpg]

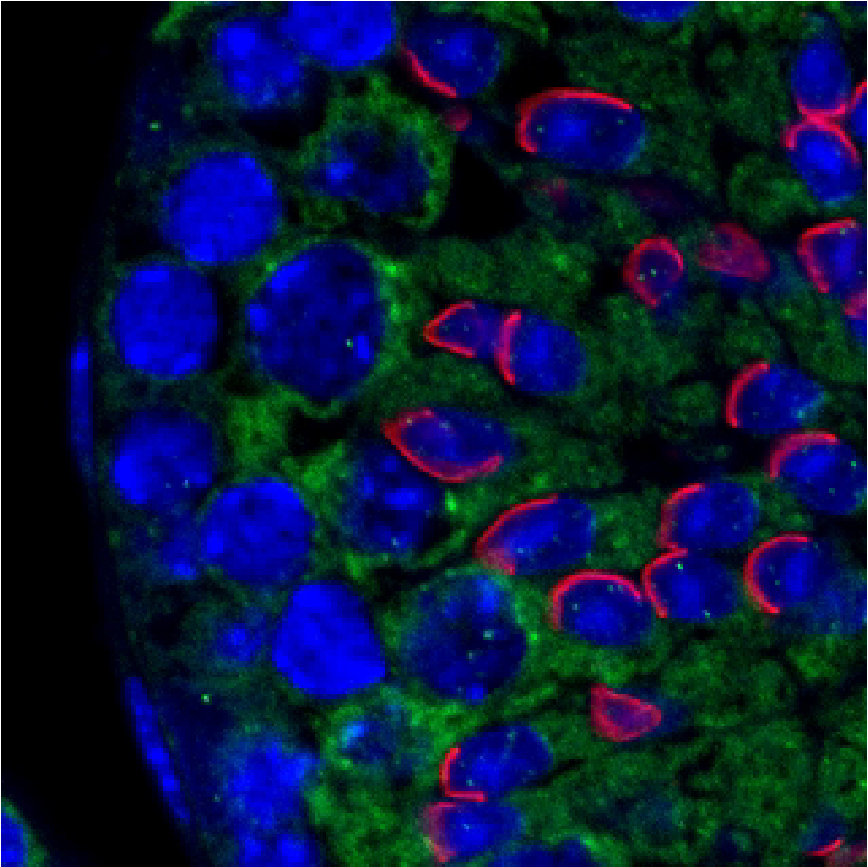

Supplement: Supplementary file 14 — Source data Fig. [file 44318_2025_659_MOESM14_ESM.zip › EMBOJ-2025-121587_Source Data/Source Data Figure 1/SD Figure 1E/Stage IX-X Merge.jpg]

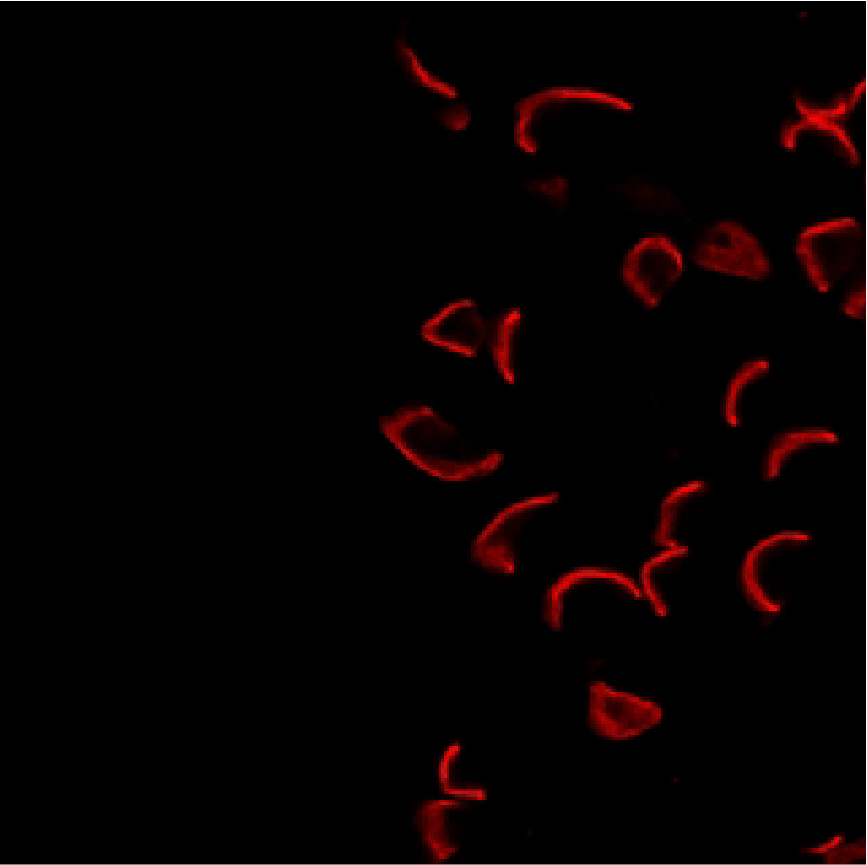

Supplement: Supplementary file 14 — Source data Fig. [file 44318_2025_659_MOESM14_ESM.zip › EMBOJ-2025-121587_Source Data/Source Data Figure 1/SD Figure 1E/Stage IX-X PNA.jpg]

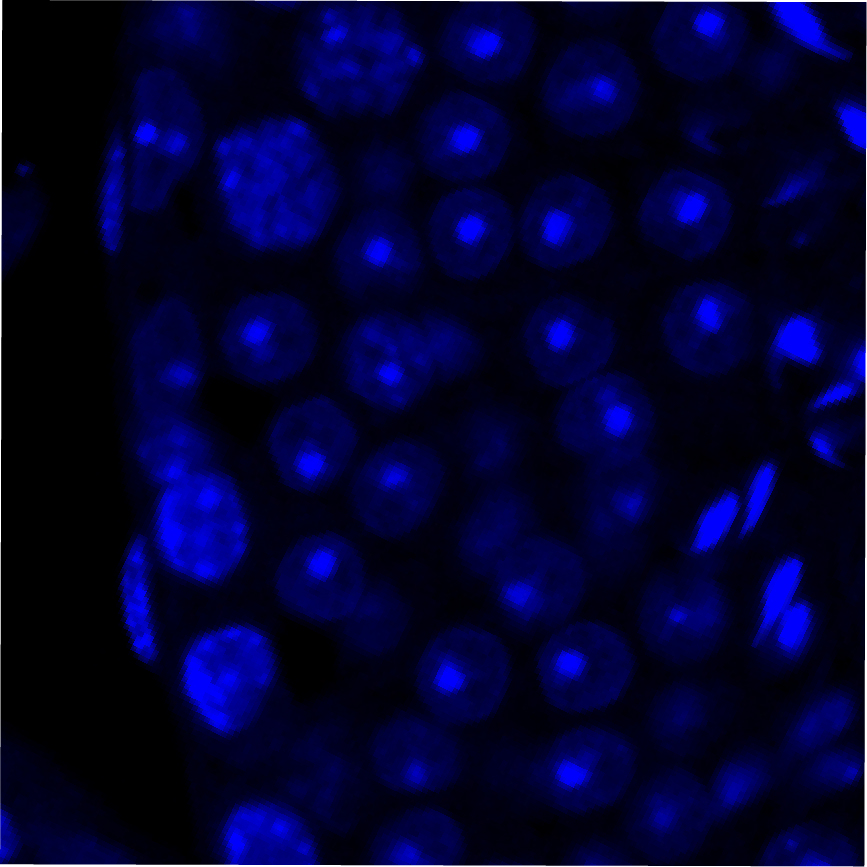

Supplement: Supplementary file 14 — Source data Fig. [file 44318_2025_659_MOESM14_ESM.zip › EMBOJ-2025-121587_Source Data/Source Data Figure 1/SD Figure 1E/Stage VII-VIII Hoechst.jpg]

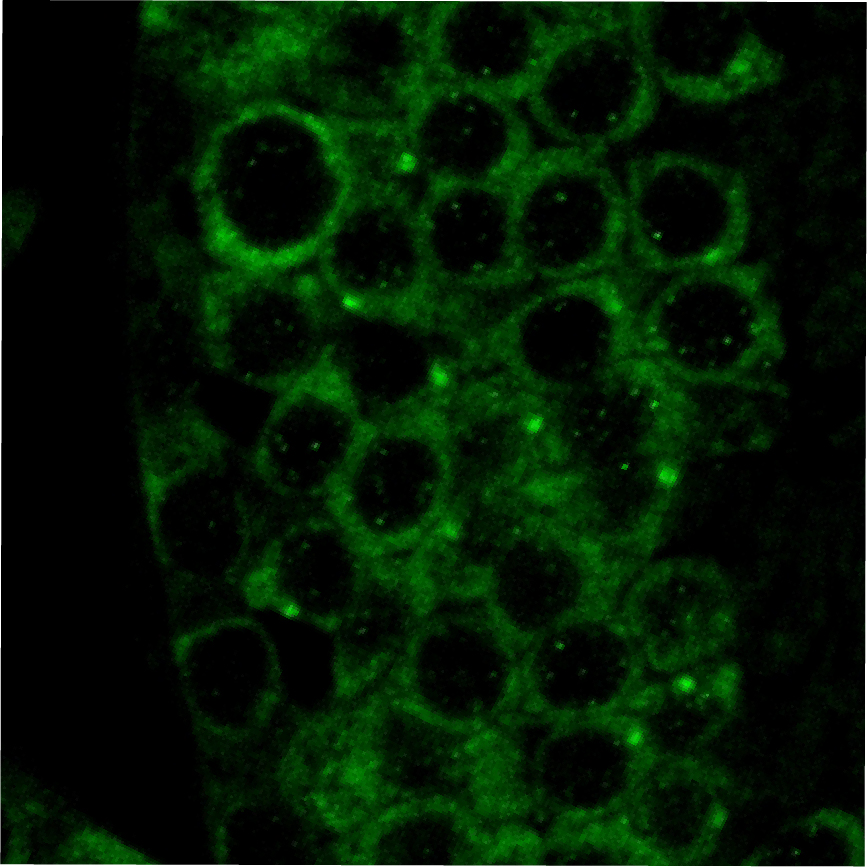

Supplement: Supplementary file 14 — Source data Fig. [file 44318_2025_659_MOESM14_ESM.zip › EMBOJ-2025-121587_Source Data/Source Data Figure 1/SD Figure 1E/Stage VII-VIII IGF2BP3.jpg]

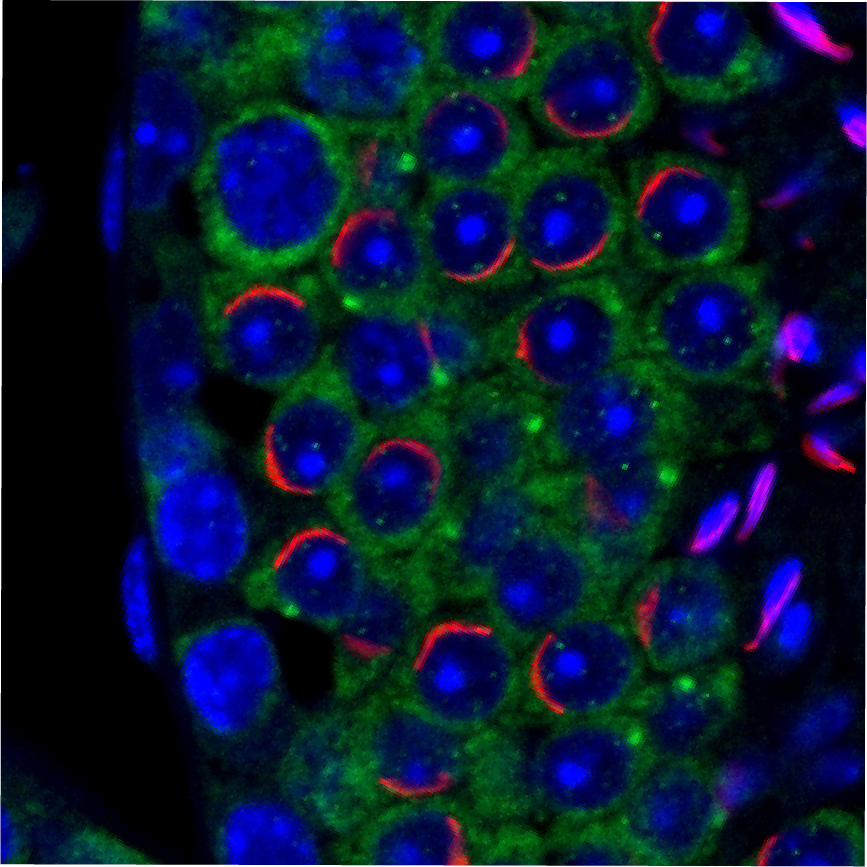

Supplement: Supplementary file 14 — Source data Fig. [file 44318_2025_659_MOESM14_ESM.zip › EMBOJ-2025-121587_Source Data/Source Data Figure 1/SD Figure 1E/Stage VII-VIII Merge.jpg]

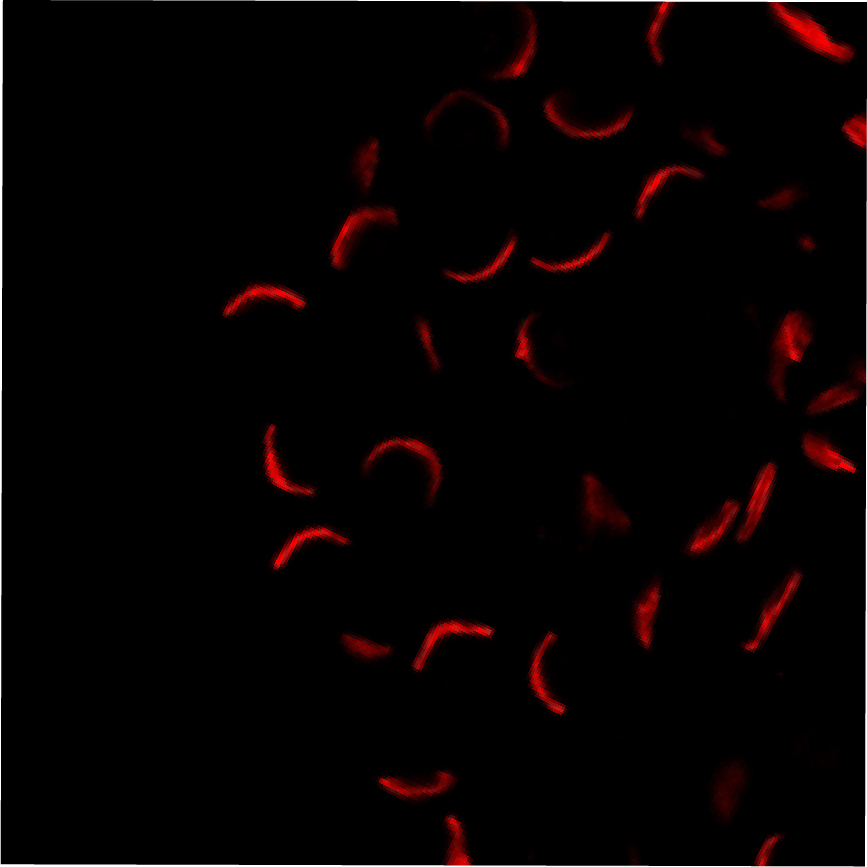

Supplement: Supplementary file 14 — Source data Fig. [file 44318_2025_659_MOESM14_ESM.zip › EMBOJ-2025-121587_Source Data/Source Data Figure 1/SD Figure 1E/Stage VII-VIII PNA.jpg]

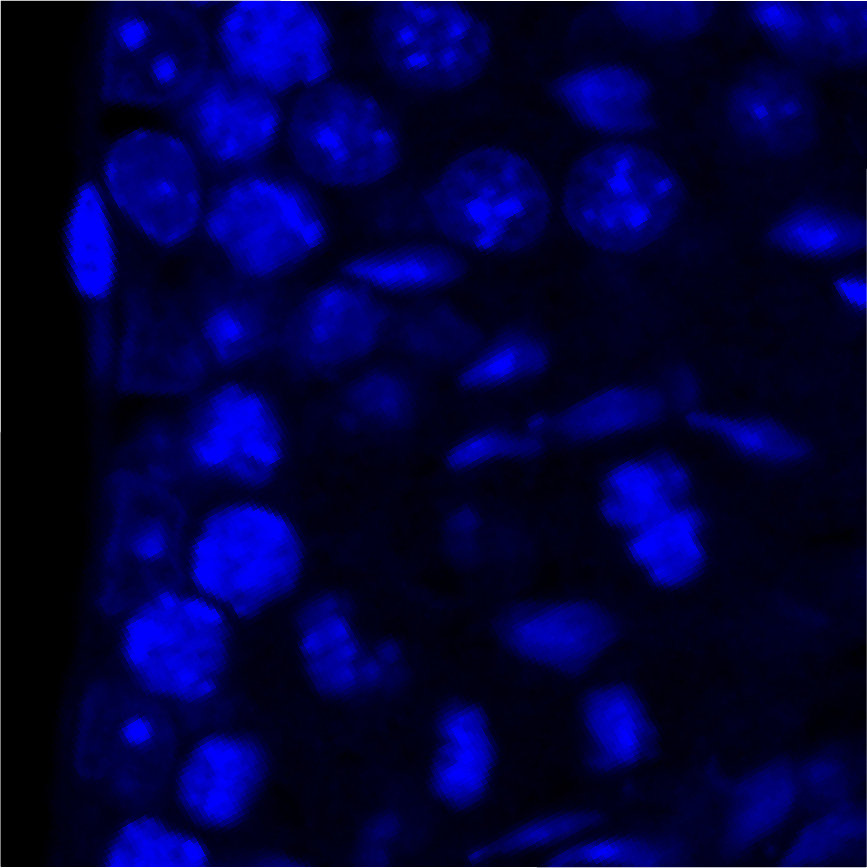

Supplement: Supplementary file 14 — Source data Fig. [file 44318_2025_659_MOESM14_ESM.zip › EMBOJ-2025-121587_Source Data/Source Data Figure 1/SD Figure 1E/Stage XI-XII Hoechst.jpg]

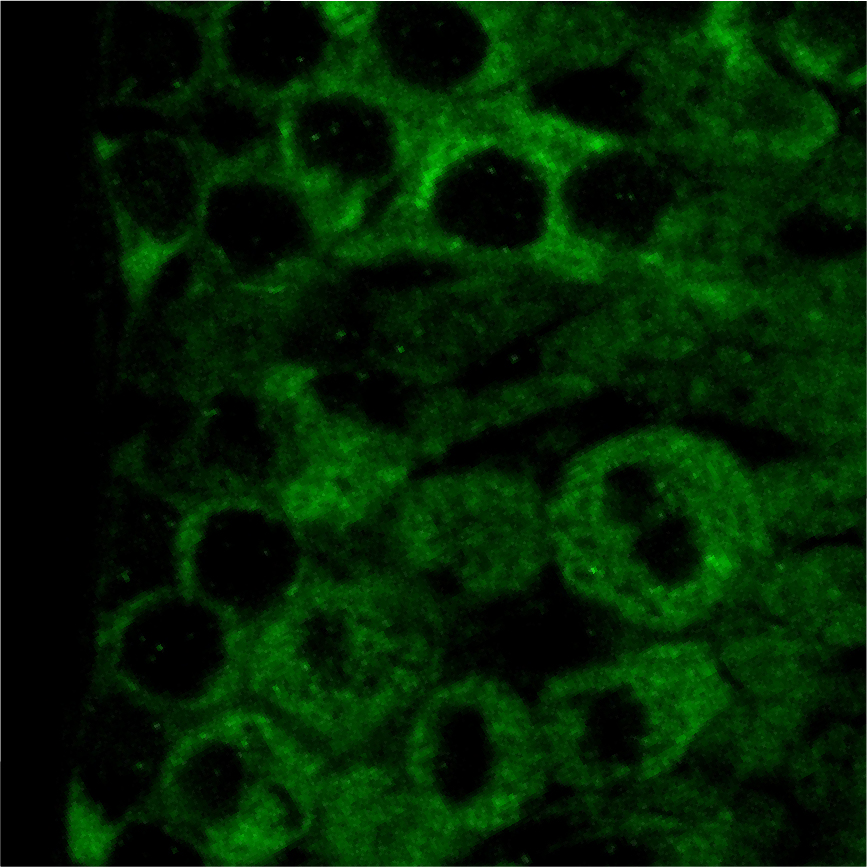

Supplement: Supplementary file 14 — Source data Fig. [file 44318_2025_659_MOESM14_ESM.zip › EMBOJ-2025-121587_Source Data/Source Data Figure 1/SD Figure 1E/Stage XI-XII IGF2BP3.jpg]

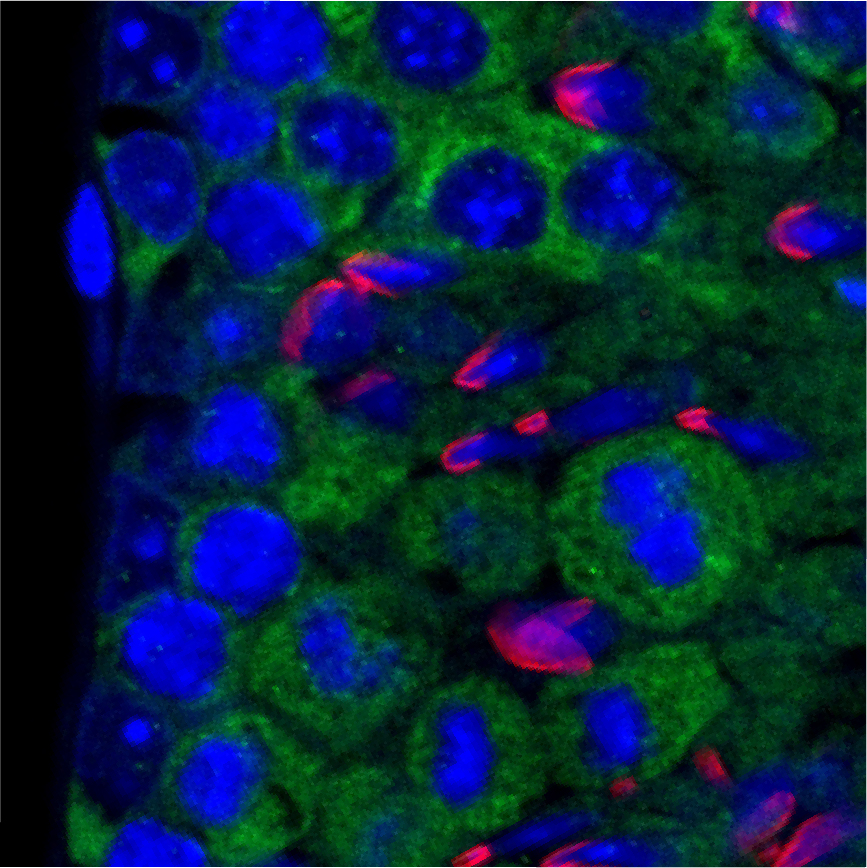

Supplement: Supplementary file 14 — Source data Fig. [file 44318_2025_659_MOESM14_ESM.zip › EMBOJ-2025-121587_Source Data/Source Data Figure 1/SD Figure 1E/Stage XI-XII Merge.jpg]

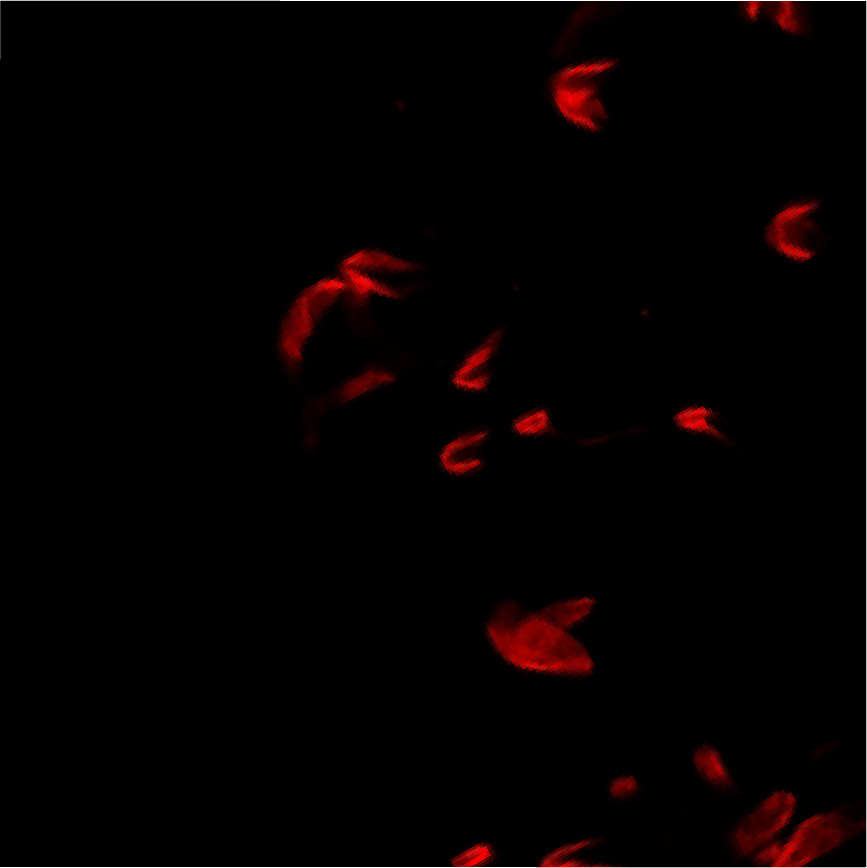

Supplement: Supplementary file 14 — Source data Fig. [file 44318_2025_659_MOESM14_ESM.zip › EMBOJ-2025-121587_Source Data/Source Data Figure 1/SD Figure 1E/Stage XI-XII PNA.jpg]

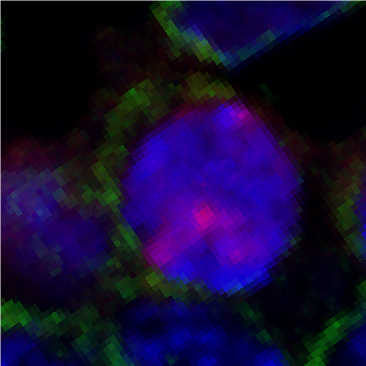

Supplement: Supplementary file 14 — Source data Fig. [file 44318_2025_659_MOESM14_ESM.zip › EMBOJ-2025-121587_Source Data/Source Data Figure 1/SD Figure 1F/L/Z.jpg]

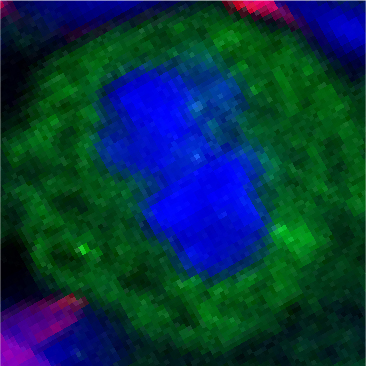

Supplement: Supplementary file 14 — Source data Fig. [file 44318_2025_659_MOESM14_ESM.zip › EMBOJ-2025-121587_Source Data/Source Data Figure 1/SD Figure 1F/MI-MII.jpg]

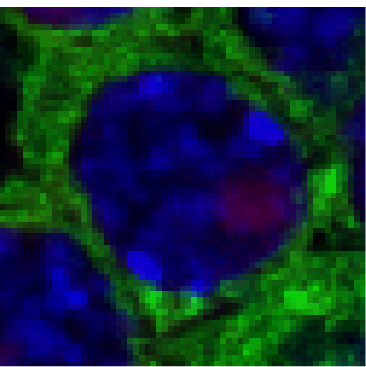

Supplement: Supplementary file 14 — Source data Fig. [file 44318_2025_659_MOESM14_ESM.zip › EMBOJ-2025-121587_Source Data/Source Data Figure 1/SD Figure 1F/P/D.jpg]

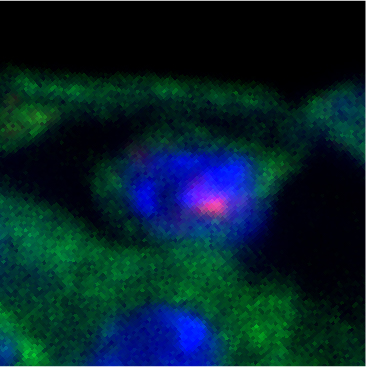

Supplement: Supplementary file 14 — Source data Fig. [file 44318_2025_659_MOESM14_ESM.zip › EMBOJ-2025-121587_Source Data/Source Data Figure 1/SD Figure 1F/SPG.jpg]

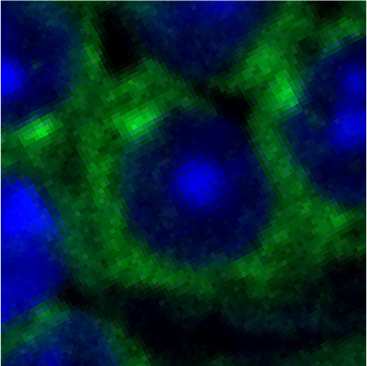

Supplement: Supplementary file 14 — Source data Fig. [file 44318_2025_659_MOESM14_ESM.zip › EMBOJ-2025-121587_Source Data/Source Data Figure 1/SD Figure 1F/Step 1.jpg]

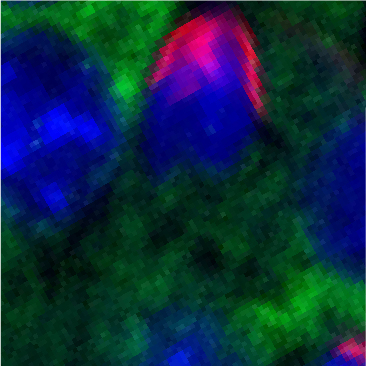

Supplement: Supplementary file 14 — Source data Fig. [file 44318_2025_659_MOESM14_ESM.zip › EMBOJ-2025-121587_Source Data/Source Data Figure 1/SD Figure 1F/Step 11-12.jpg]

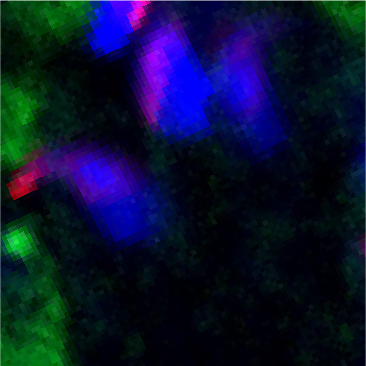

Supplement: Supplementary file 14 — Source data Fig. [file 44318_2025_659_MOESM14_ESM.zip › EMBOJ-2025-121587_Source Data/Source Data Figure 1/SD Figure 1F/Step 13-16.jpg]

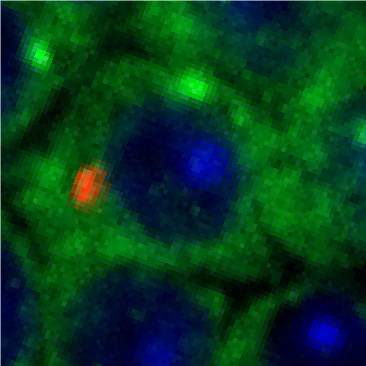

Supplement: Supplementary file 14 — Source data Fig. [file 44318_2025_659_MOESM14_ESM.zip › EMBOJ-2025-121587_Source Data/Source Data Figure 1/SD Figure 1F/Step 2-3.jpg]

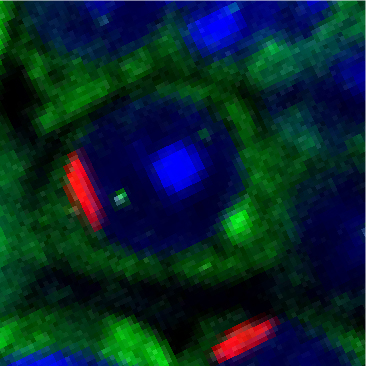

Supplement: Supplementary file 14 — Source data Fig. [file 44318_2025_659_MOESM14_ESM.zip › EMBOJ-2025-121587_Source Data/Source Data Figure 1/SD Figure 1F/Step 4-6.jpg]

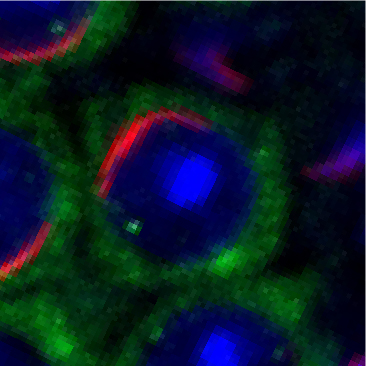

Supplement: Supplementary file 14 — Source data Fig. [file 44318_2025_659_MOESM14_ESM.zip › EMBOJ-2025-121587_Source Data/Source Data Figure 1/SD Figure 1F/Step 7-8.jpg]

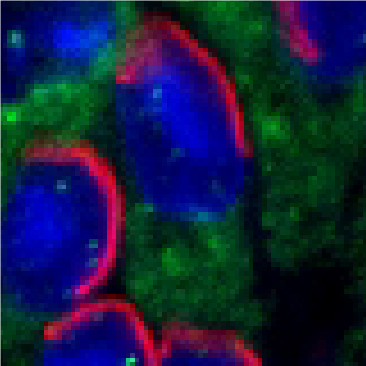

Supplement: Supplementary file 14 — Source data Fig. [file 44318_2025_659_MOESM14_ESM.zip › EMBOJ-2025-121587_Source Data/Source Data Figure 1/SD Figure 1F/Step 9-10.jpg]

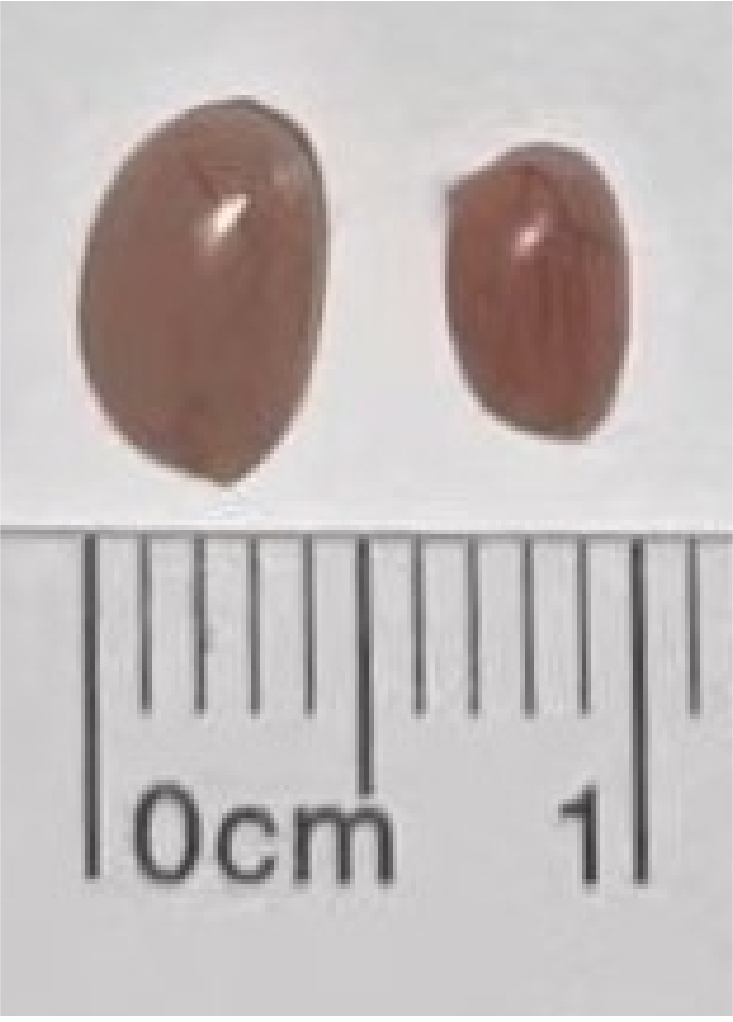

Supplement: Supplementary file 14 — Source data Fig. [file 44318_2025_659_MOESM14_ESM.zip › EMBOJ-2025-121587_Source Data/Source Data Figure 2/SD Figure 2A/SD Figure 2A.jpg]

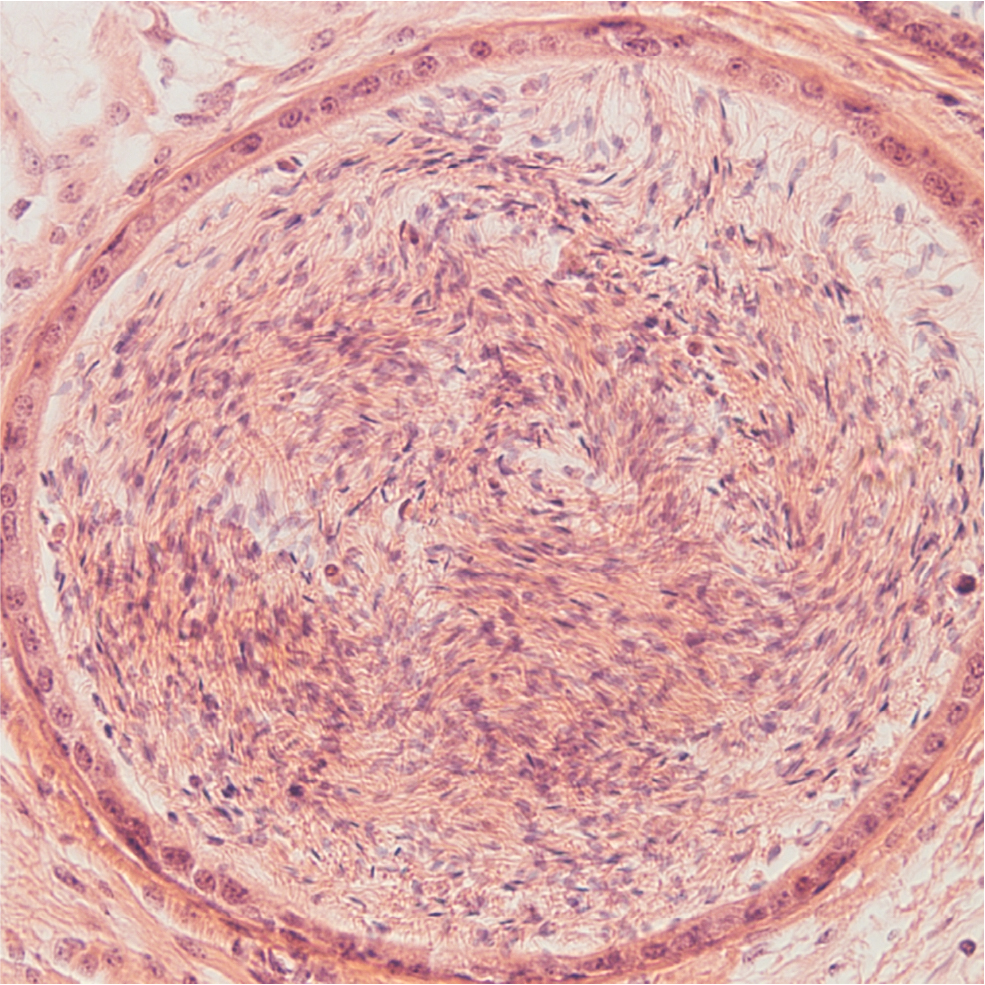

Supplement: Supplementary file 14 — Source data Fig. [file 44318_2025_659_MOESM14_ESM.zip › EMBOJ-2025-121587_Source Data/Source Data Figure 2/SD Figure 2E/Igf2bp3+/-.jpg]

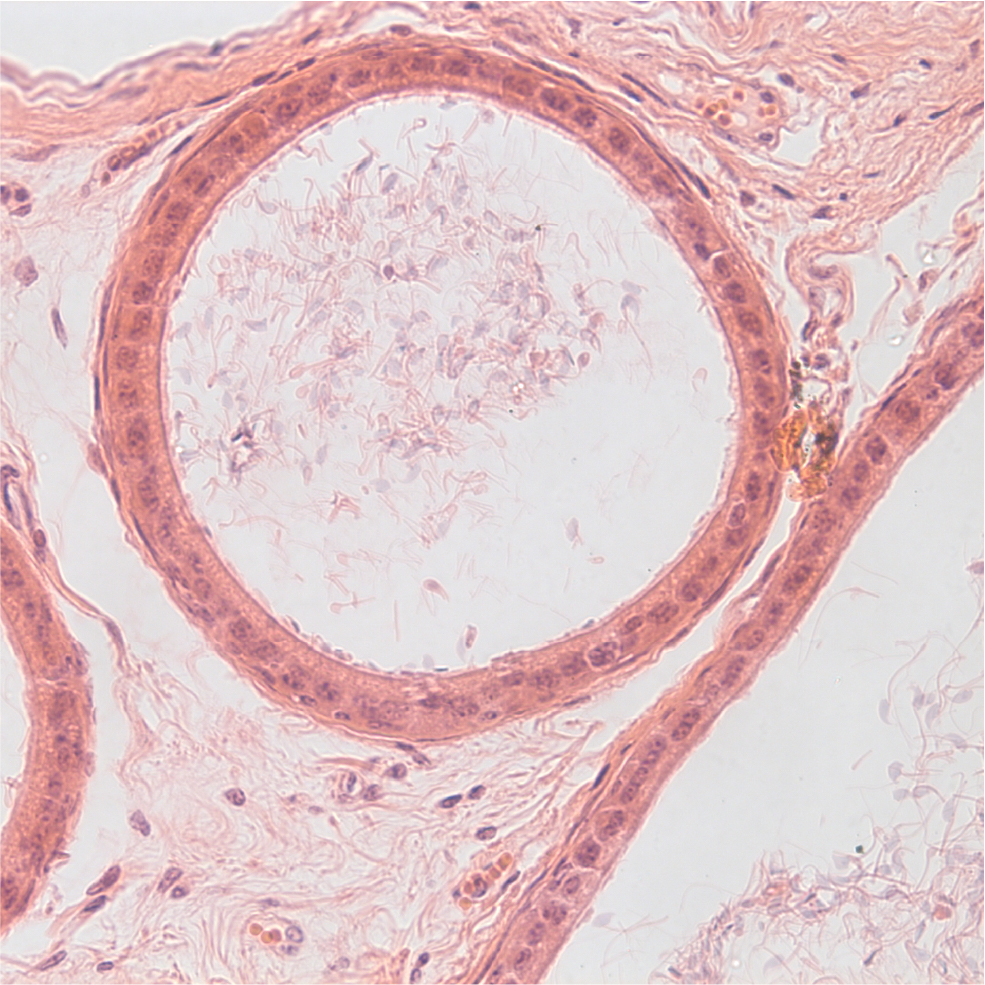

Supplement: Supplementary file 14 — Source data Fig. [file 44318_2025_659_MOESM14_ESM.zip › EMBOJ-2025-121587_Source Data/Source Data Figure 2/SD Figure 2E/Igf2bp3-/-.jpg]

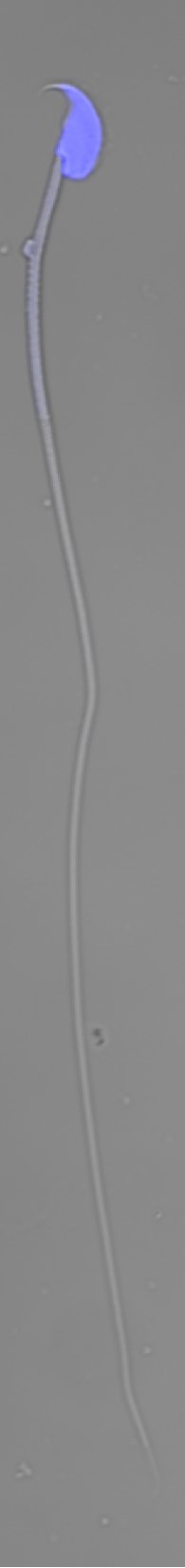

Supplement: Supplementary file 14 — Source data Fig. [file 44318_2025_659_MOESM14_ESM.zip › EMBOJ-2025-121587_Source Data/Source Data Figure 2/SD Figure 2F/Igf2bp3+/- .jpg]

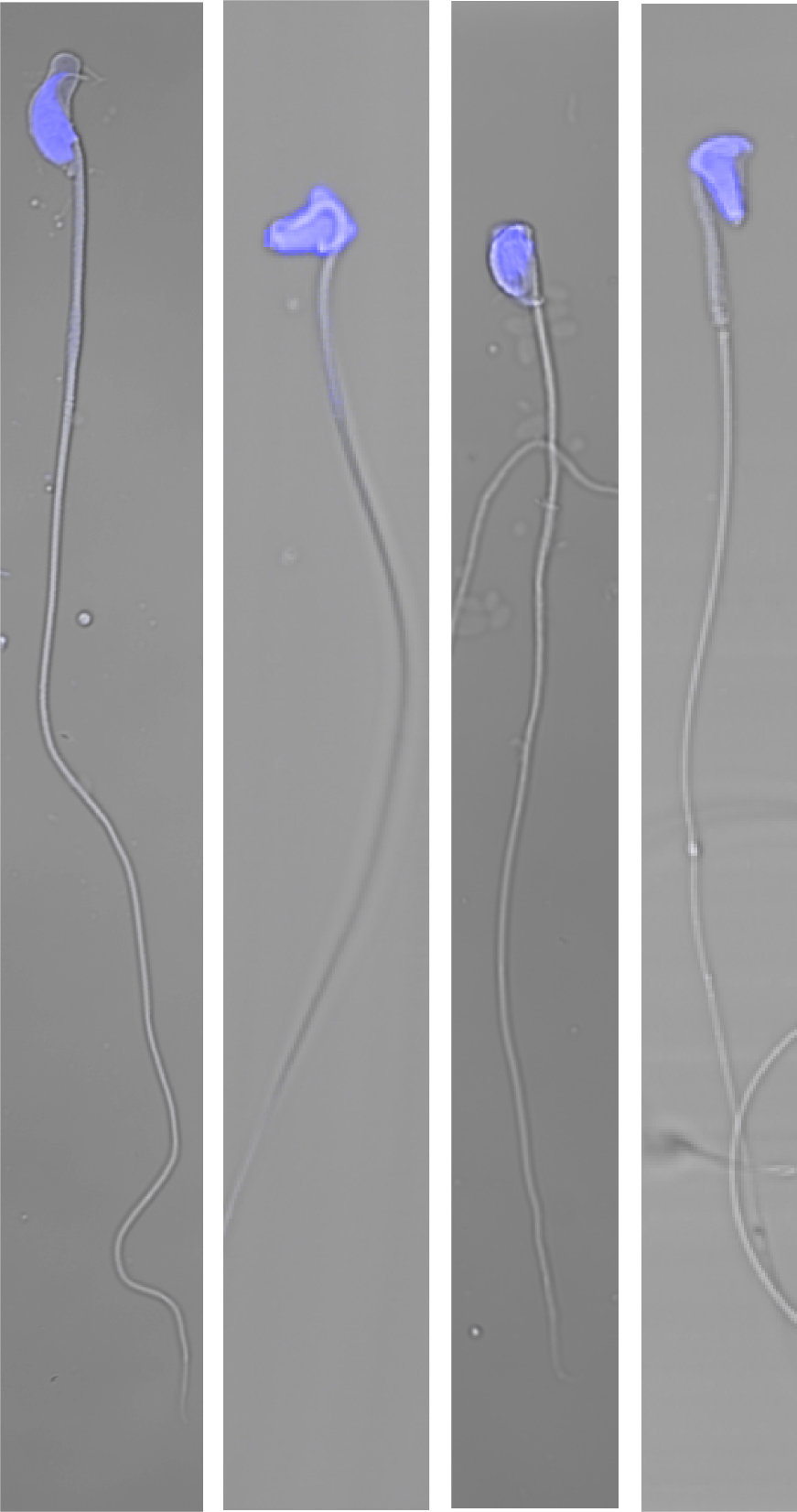

Supplement: Supplementary file 14 — Source data Fig. [file 44318_2025_659_MOESM14_ESM.zip › EMBOJ-2025-121587_Source Data/Source Data Figure 2/SD Figure 2F/Igf2bp3-/- .jpg]

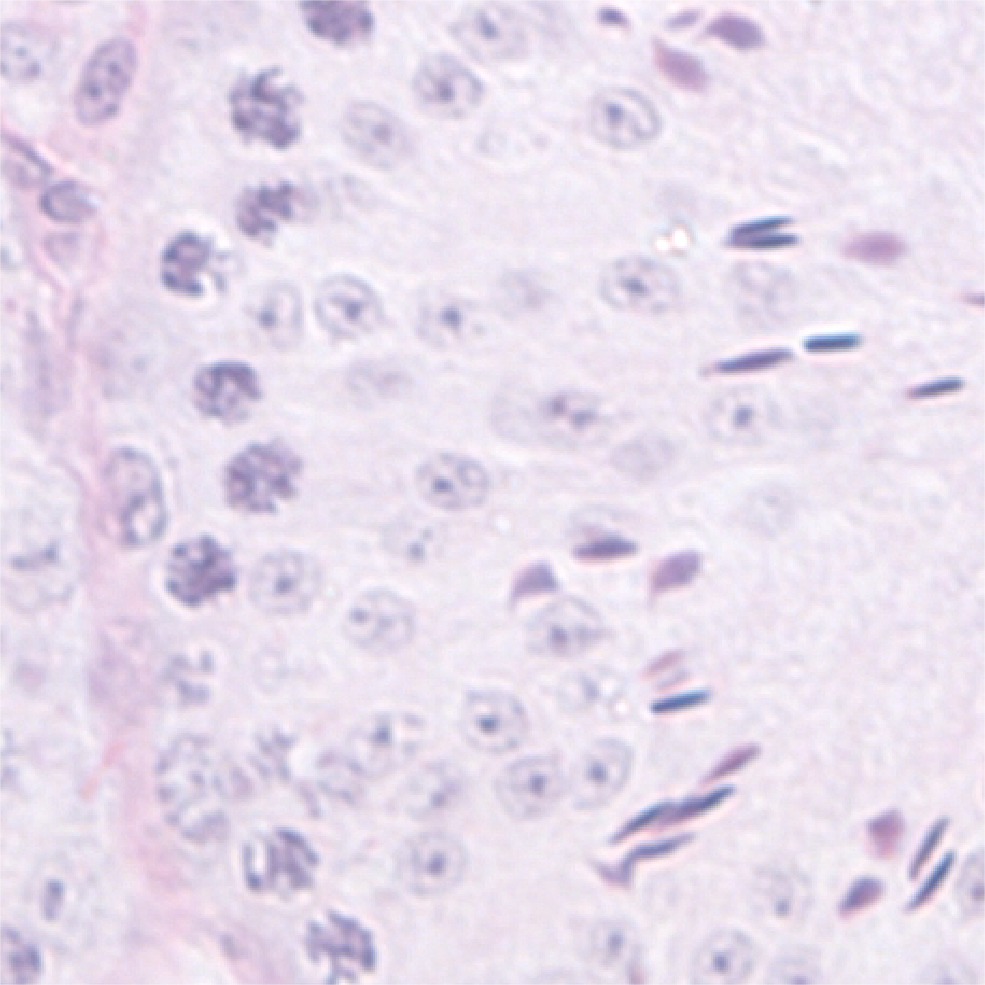

Supplement: Supplementary file 14 — Source data Fig. [file 44318_2025_659_MOESM14_ESM.zip › EMBOJ-2025-121587_Source Data/Source Data Figure 2/SD Figure 2H/Igf2bp3+/- Stage I-II.jpg]

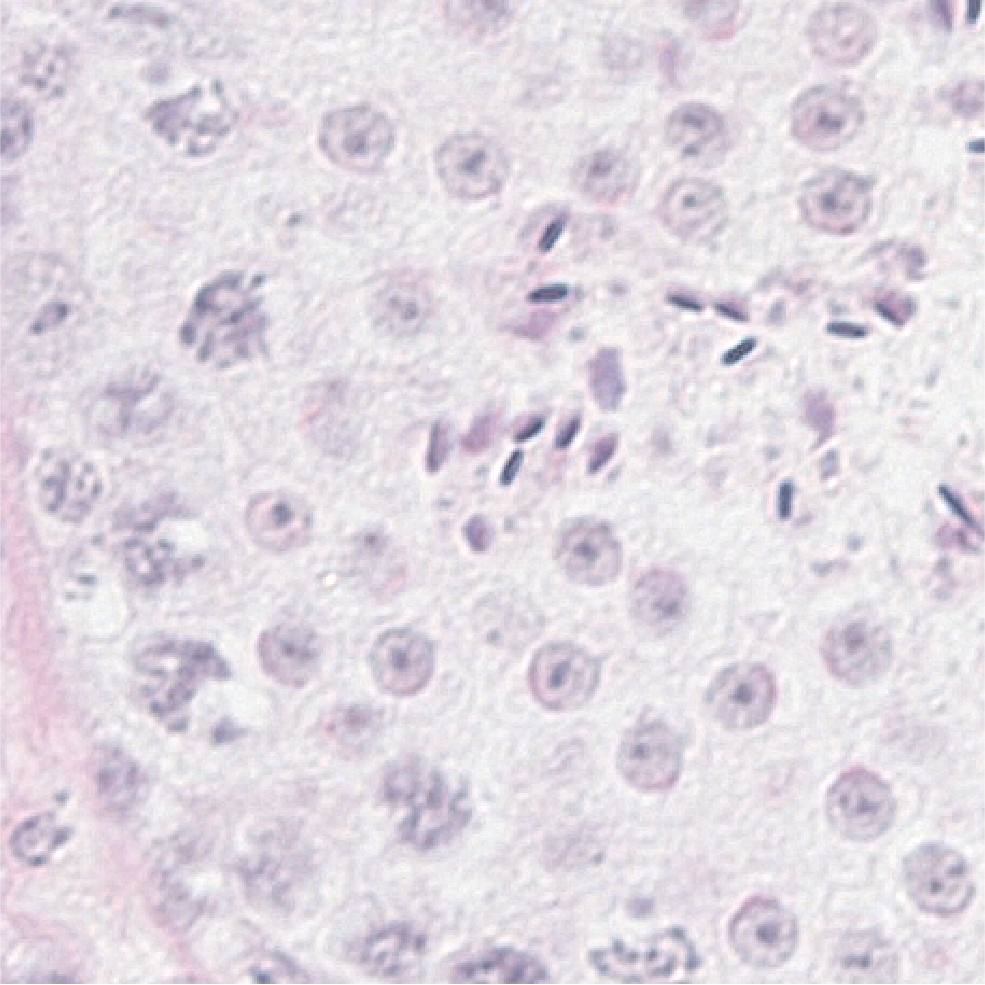

Supplement: Supplementary file 14 — Source data Fig. [file 44318_2025_659_MOESM14_ESM.zip › EMBOJ-2025-121587_Source Data/Source Data Figure 2/SD Figure 2H/Igf2bp3+/- Stage IV-VI.jpg]

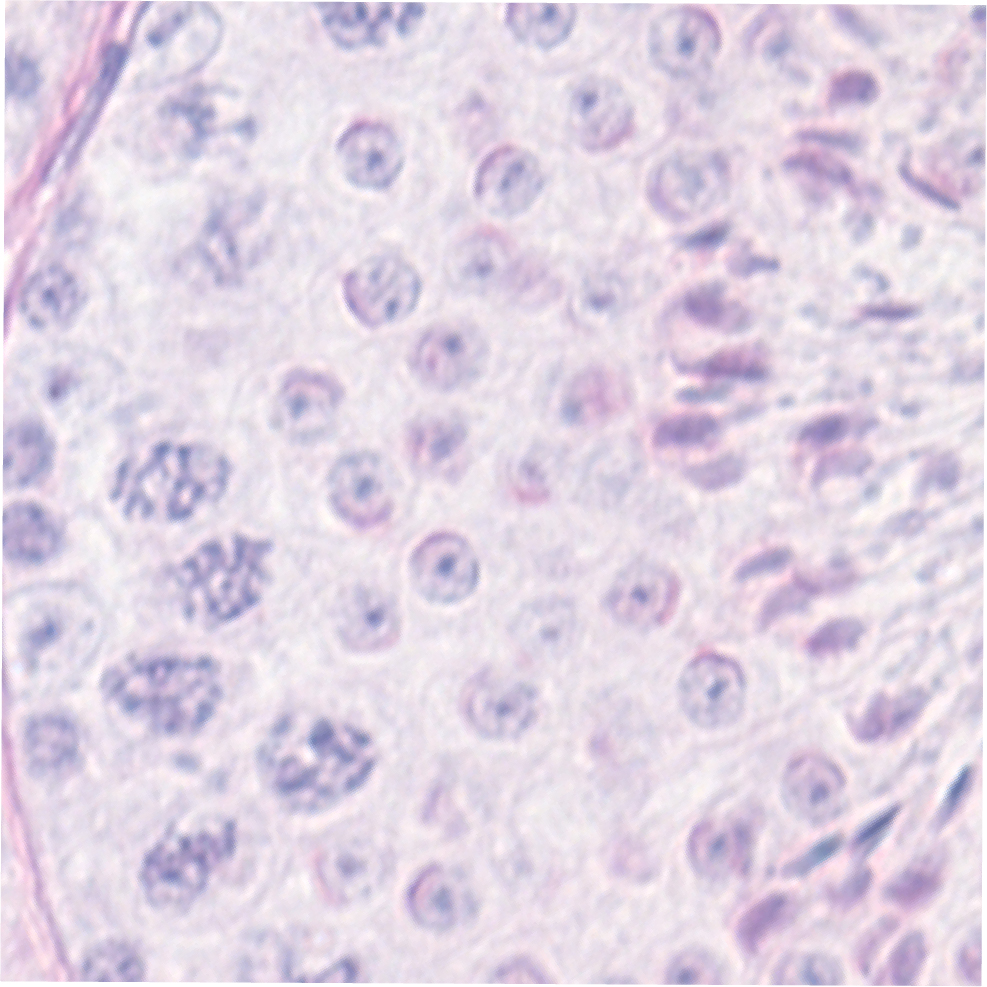

Supplement: Supplementary file 14 — Source data Fig. [file 44318_2025_659_MOESM14_ESM.zip › EMBOJ-2025-121587_Source Data/Source Data Figure 2/SD Figure 2H/Igf2bp3+/- Stage VII-VIII.jpg]

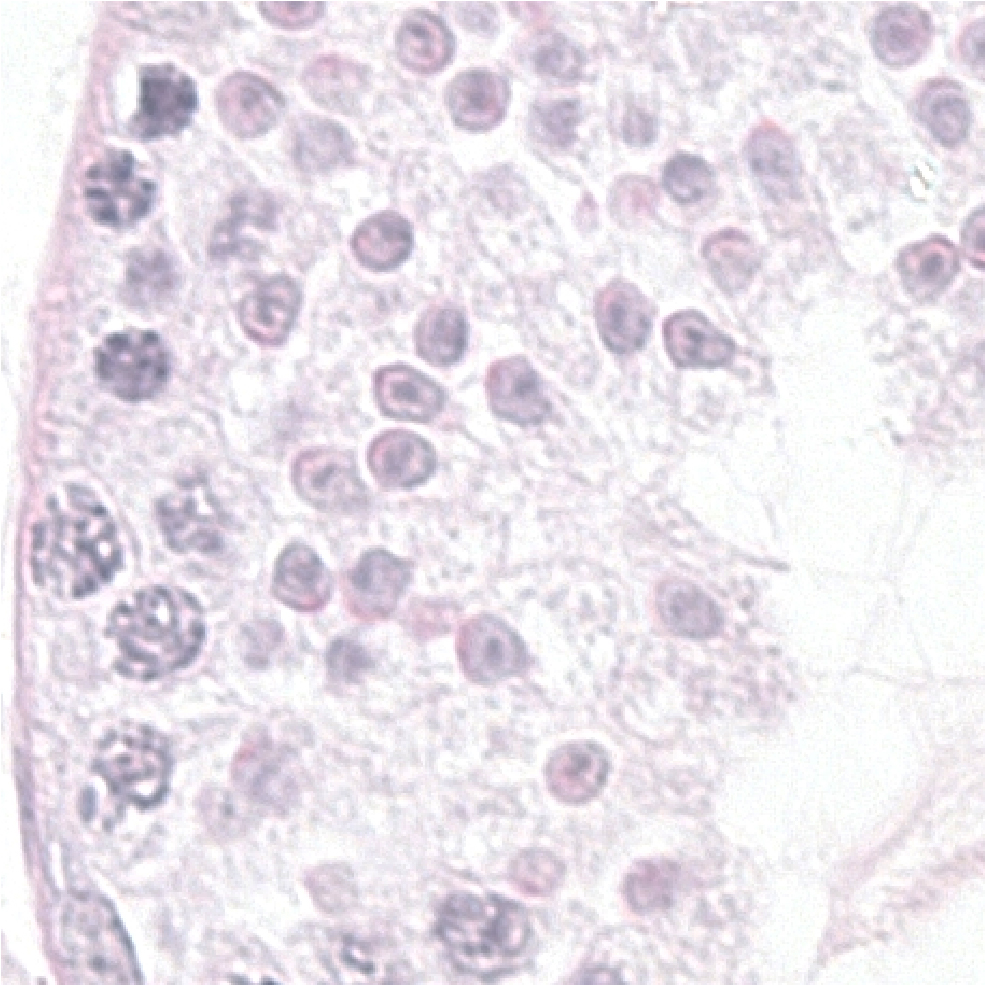

Supplement: Supplementary file 14 — Source data Fig. [file 44318_2025_659_MOESM14_ESM.zip › EMBOJ-2025-121587_Source Data/Source Data Figure 2/SD Figure 2H/Igf2bp3+/- Stage VIII-IX.jpg]

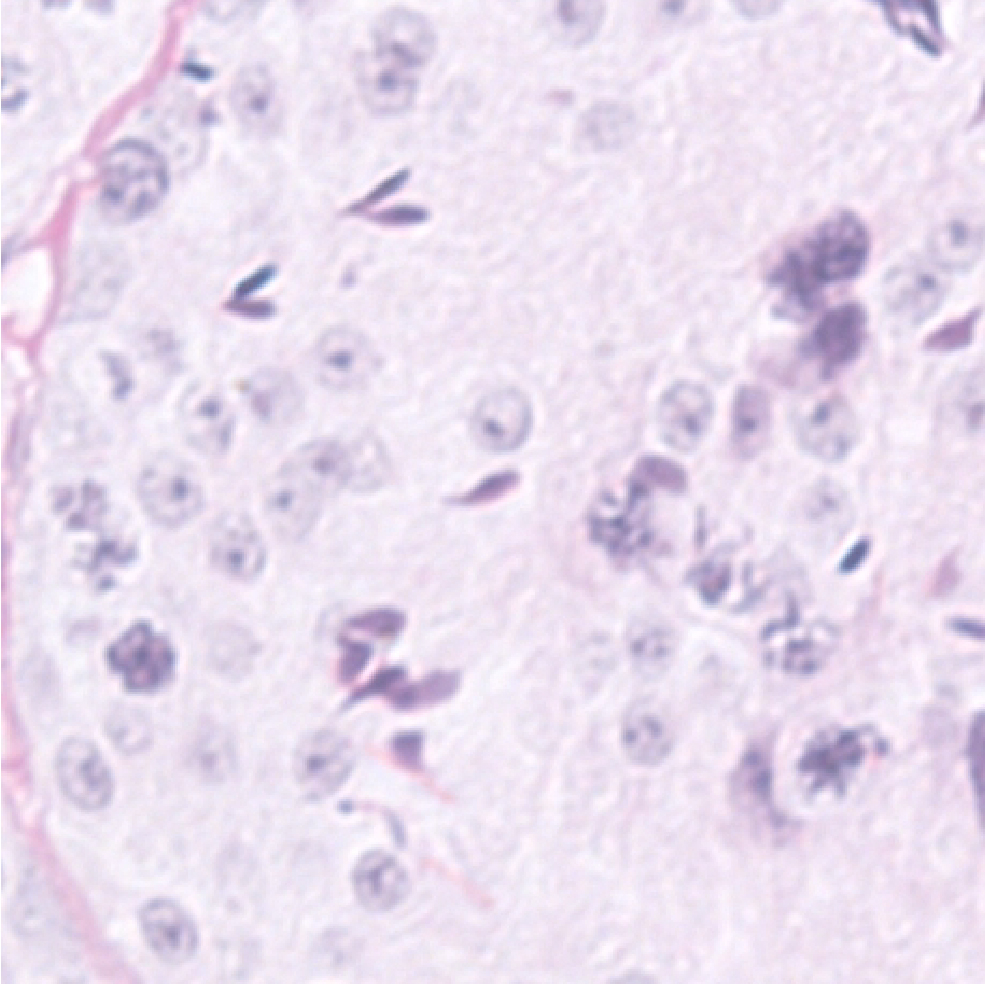

Supplement: Supplementary file 14 — Source data Fig. [file 44318_2025_659_MOESM14_ESM.zip › EMBOJ-2025-121587_Source Data/Source Data Figure 2/SD Figure 2H/Igf2bp3-/- Stage I-II.jpg]

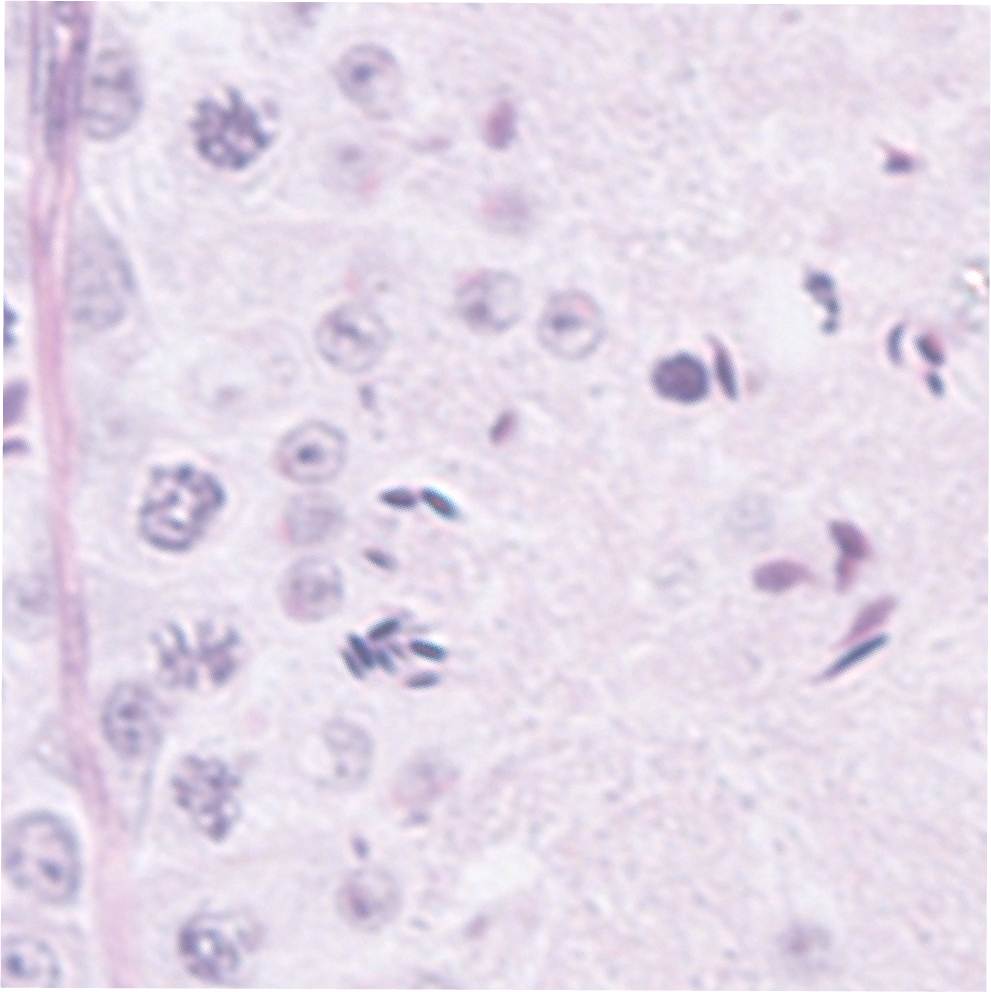

Supplement: Supplementary file 14 — Source data Fig. [file 44318_2025_659_MOESM14_ESM.zip › EMBOJ-2025-121587_Source Data/Source Data Figure 2/SD Figure 2H/Igf2bp3-/- Stage IV-VI.jpg]

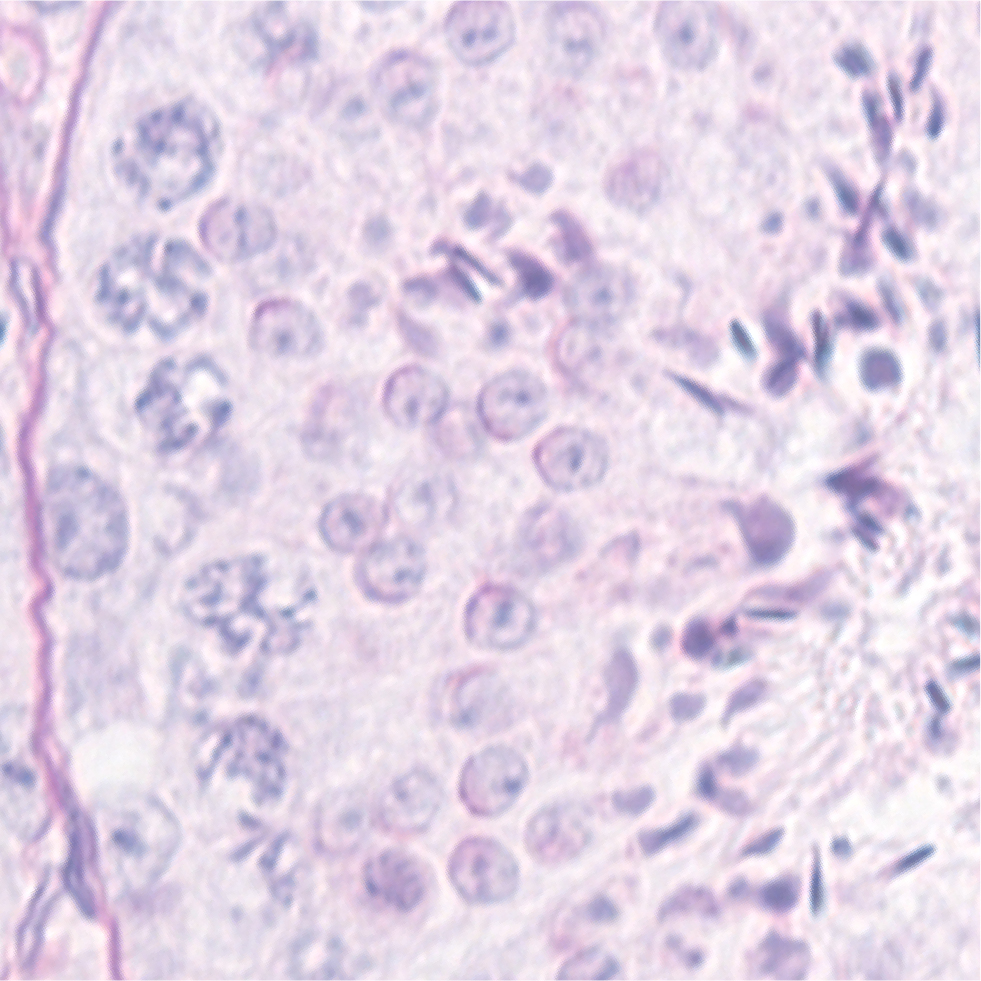

Supplement: Supplementary file 14 — Source data Fig. [file 44318_2025_659_MOESM14_ESM.zip › EMBOJ-2025-121587_Source Data/Source Data Figure 2/SD Figure 2H/Igf2bp3-/- Stage VII-VIII.jpg]

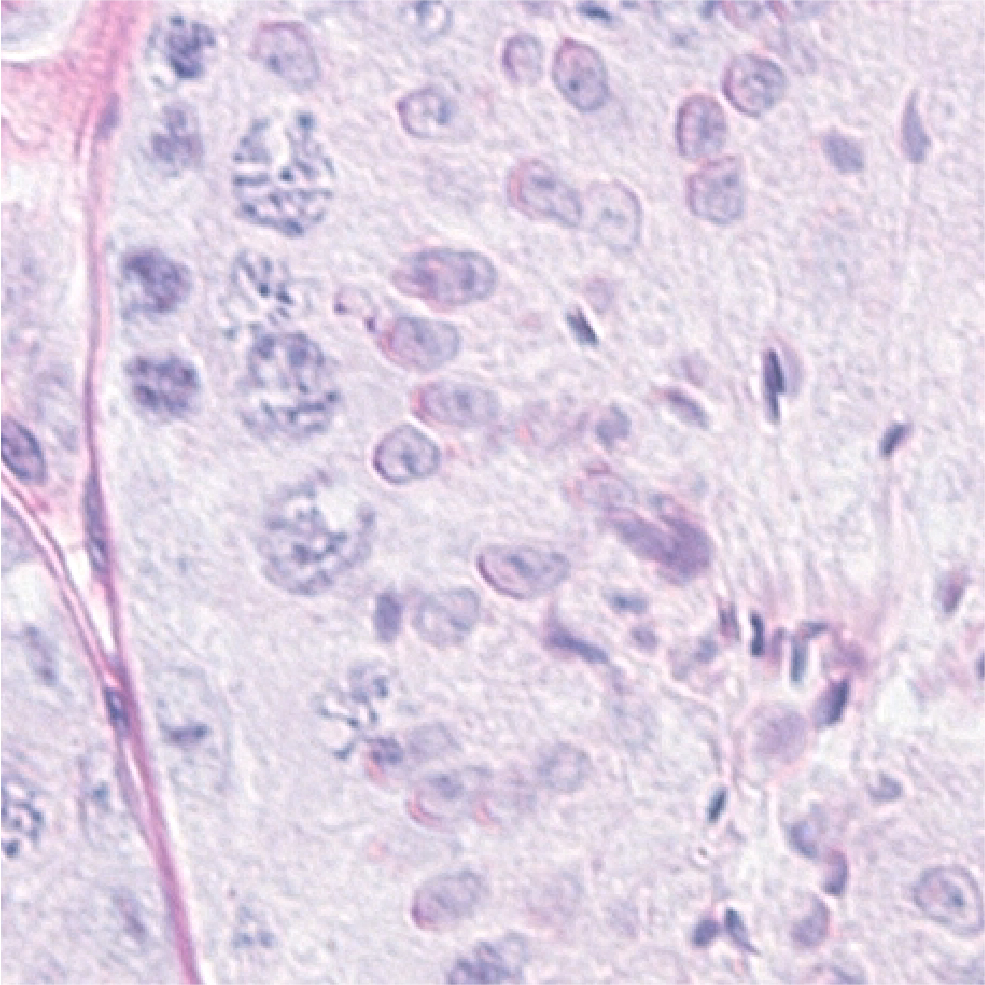

Supplement: Supplementary file 14 — Source data Fig. [file 44318_2025_659_MOESM14_ESM.zip › EMBOJ-2025-121587_Source Data/Source Data Figure 2/SD Figure 2H/Igf2bp3-/- Stage VIII-IX.jpg]

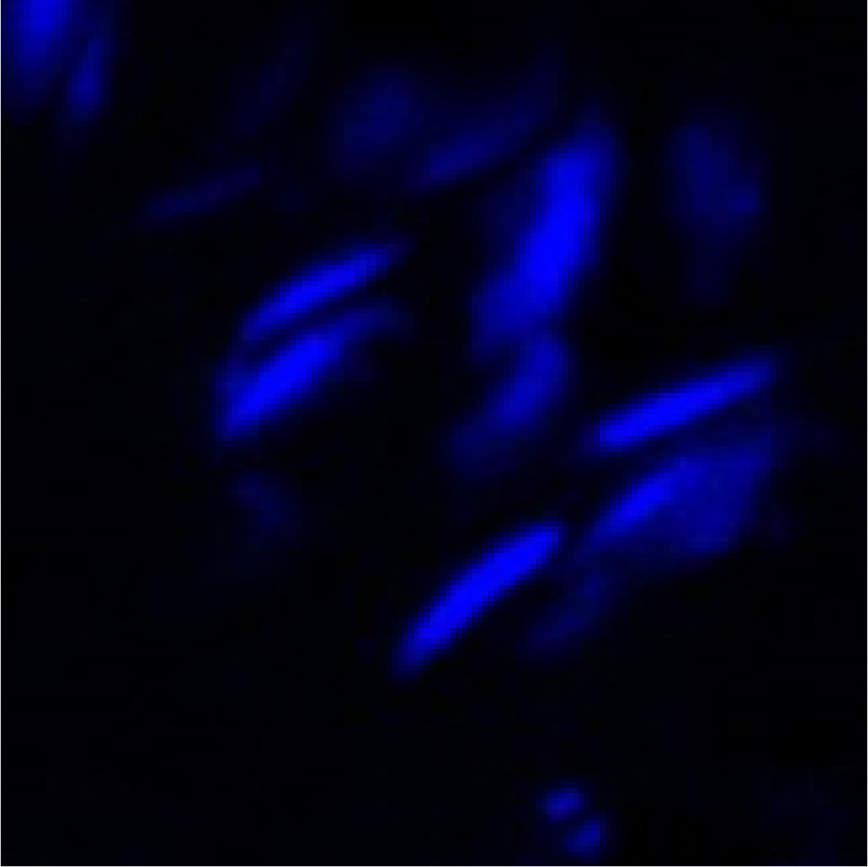

Supplement: Supplementary file 14 — Source data Fig. [file 44318_2025_659_MOESM14_ESM.zip › EMBOJ-2025-121587_Source Data/Source Data Figure 2/SD Figure 2J/Igf2bp3+/- Hoechst.jpg]

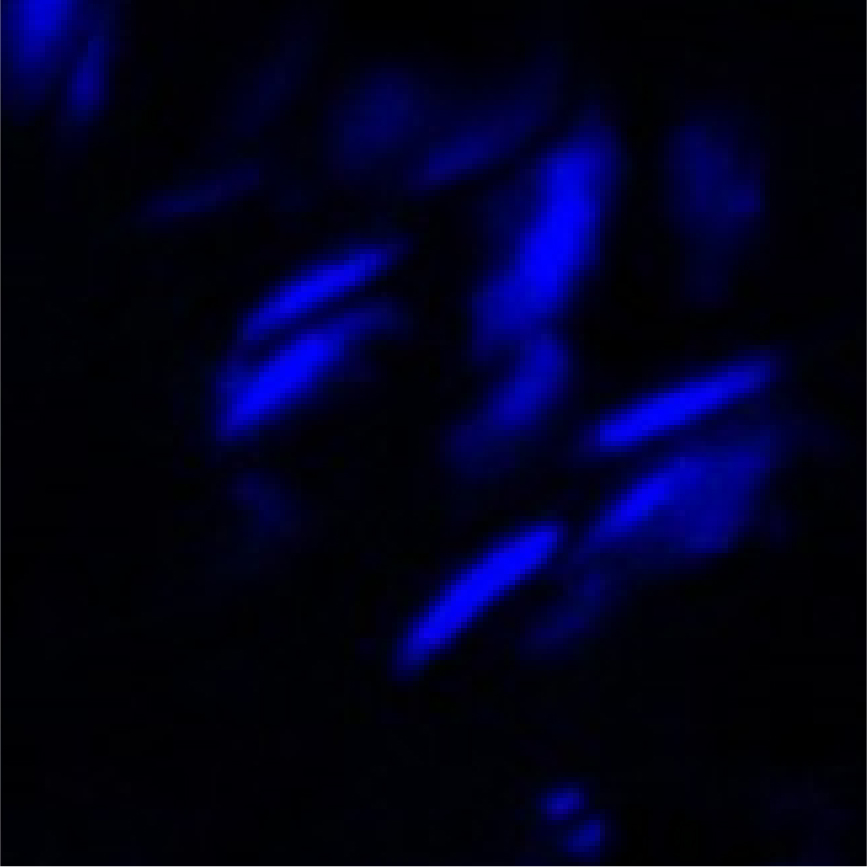

Supplement: Supplementary file 14 — Source data Fig. [file 44318_2025_659_MOESM14_ESM.zip › EMBOJ-2025-121587_Source Data/Source Data Figure 2/SD Figure 2J/Igf2bp3+/- Merge.jpg]

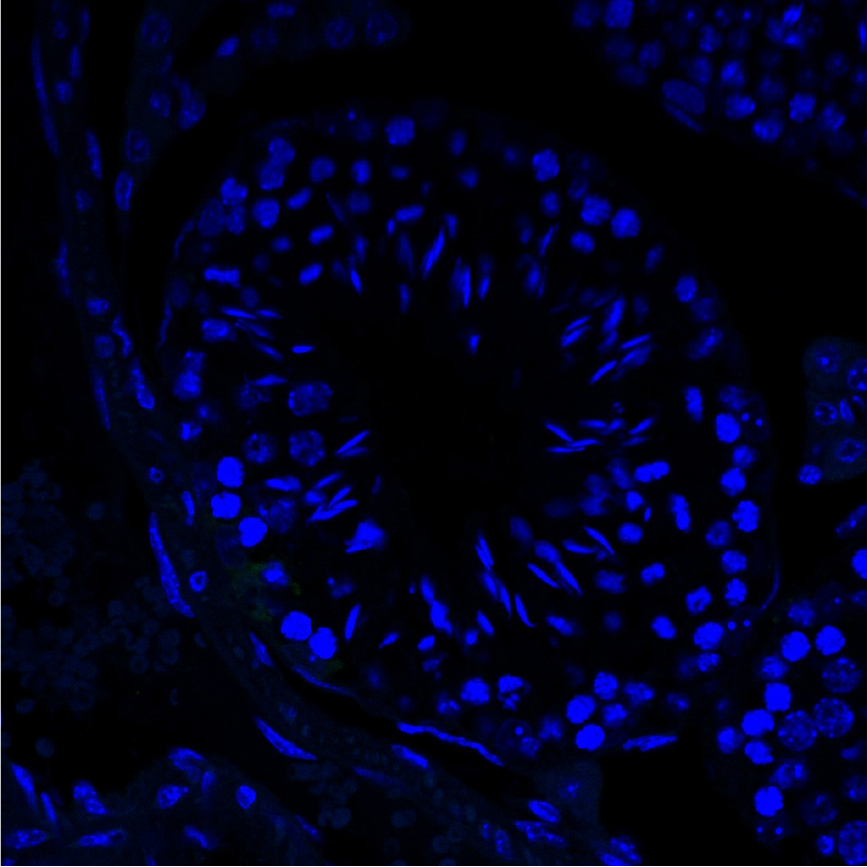

Supplement: Supplementary file 14 — Source data Fig. [file 44318_2025_659_MOESM14_ESM.zip › EMBOJ-2025-121587_Source Data/Source Data Figure 2/SD Figure 2J/Igf2bp3+/- Stage XI-XII.jpg]

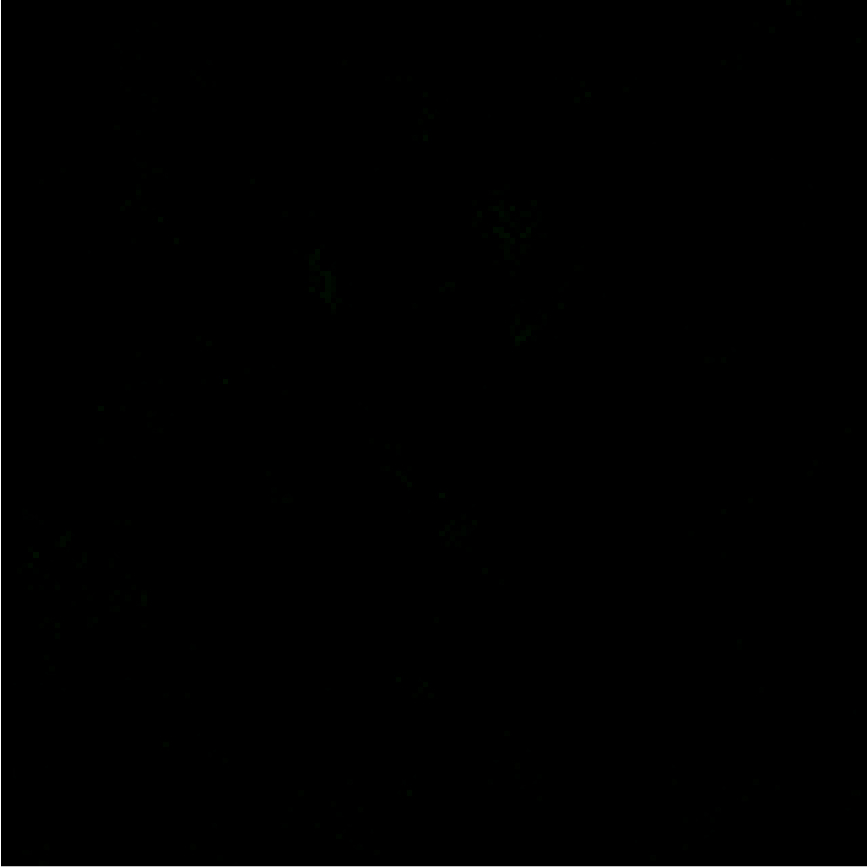

Supplement: Supplementary file 14 — Source data Fig. [file 44318_2025_659_MOESM14_ESM.zip › EMBOJ-2025-121587_Source Data/Source Data Figure 2/SD Figure 2J/Igf2bp3+/- TUNEL.jpg]

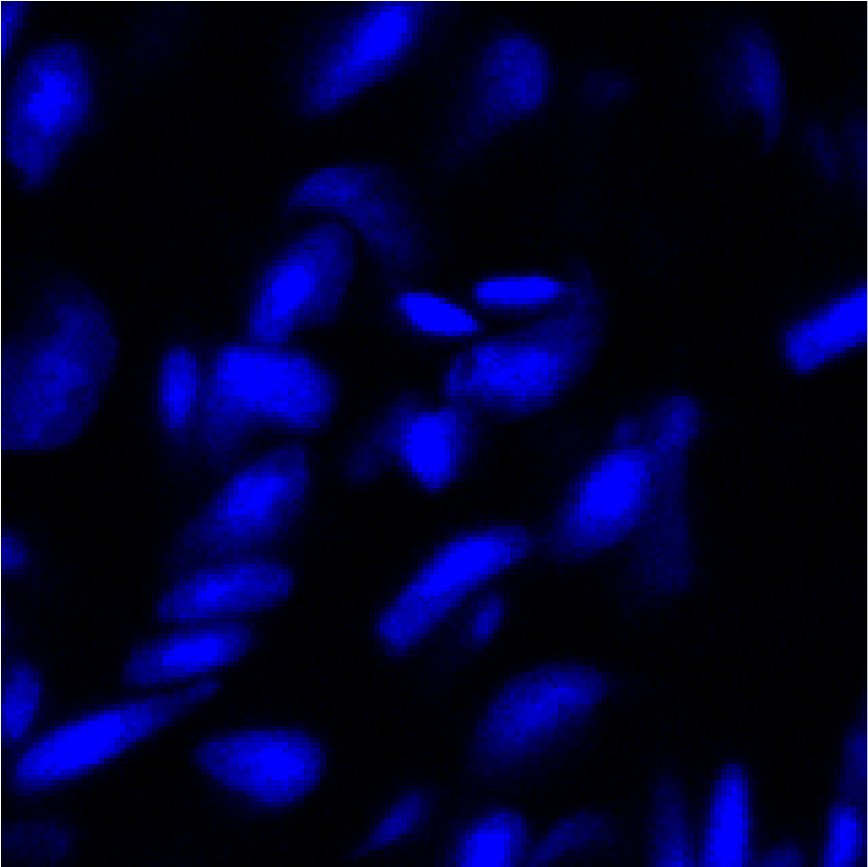

Supplement: Supplementary file 14 — Source data Fig. [file 44318_2025_659_MOESM14_ESM.zip › EMBOJ-2025-121587_Source Data/Source Data Figure 2/SD Figure 2J/Igf2bp3-/- Hoechst .jpg]

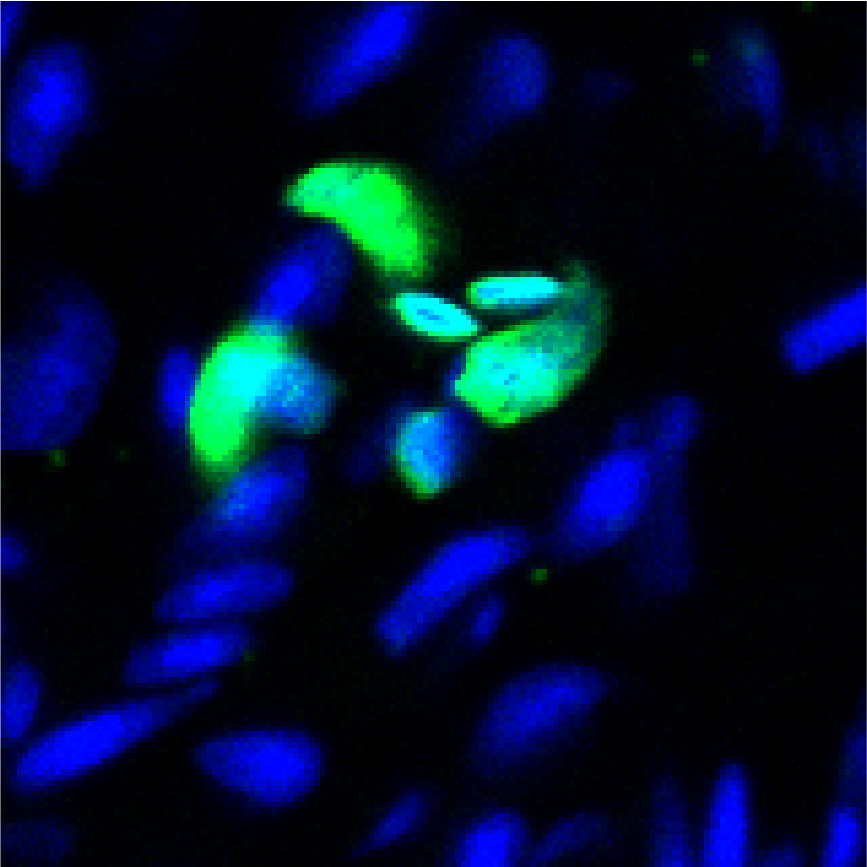

Supplement: Supplementary file 14 — Source data Fig. [file 44318_2025_659_MOESM14_ESM.zip › EMBOJ-2025-121587_Source Data/Source Data Figure 2/SD Figure 2J/Igf2bp3-/- Merge .jpg]

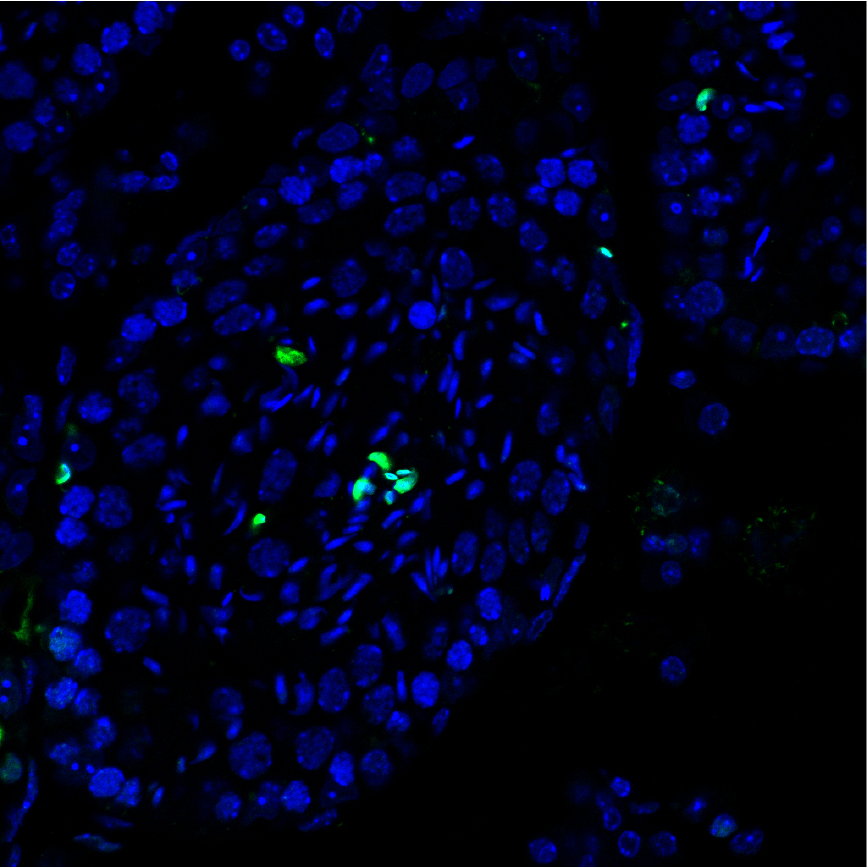

Supplement: Supplementary file 14 — Source data Fig. [file 44318_2025_659_MOESM14_ESM.zip › EMBOJ-2025-121587_Source Data/Source Data Figure 2/SD Figure 2J/Igf2bp3-/- Stage XI-XII.jpg]

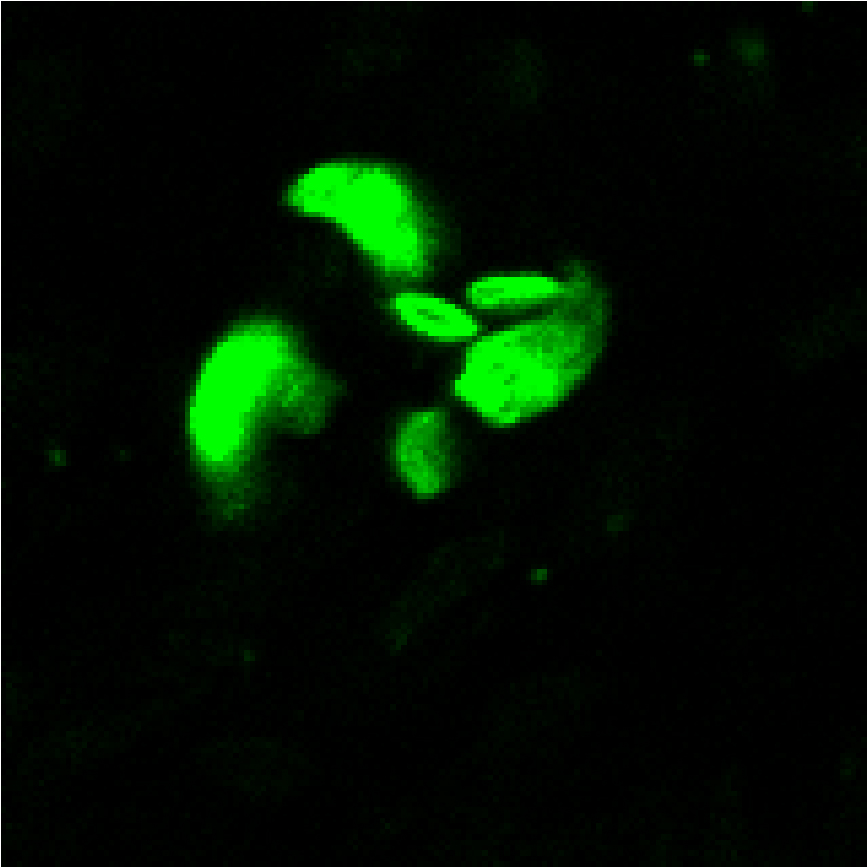

Supplement: Supplementary file 14 — Source data Fig. [file 44318_2025_659_MOESM14_ESM.zip › EMBOJ-2025-121587_Source Data/Source Data Figure 2/SD Figure 2J/Igf2bp3-/- TUNEL .jpg]

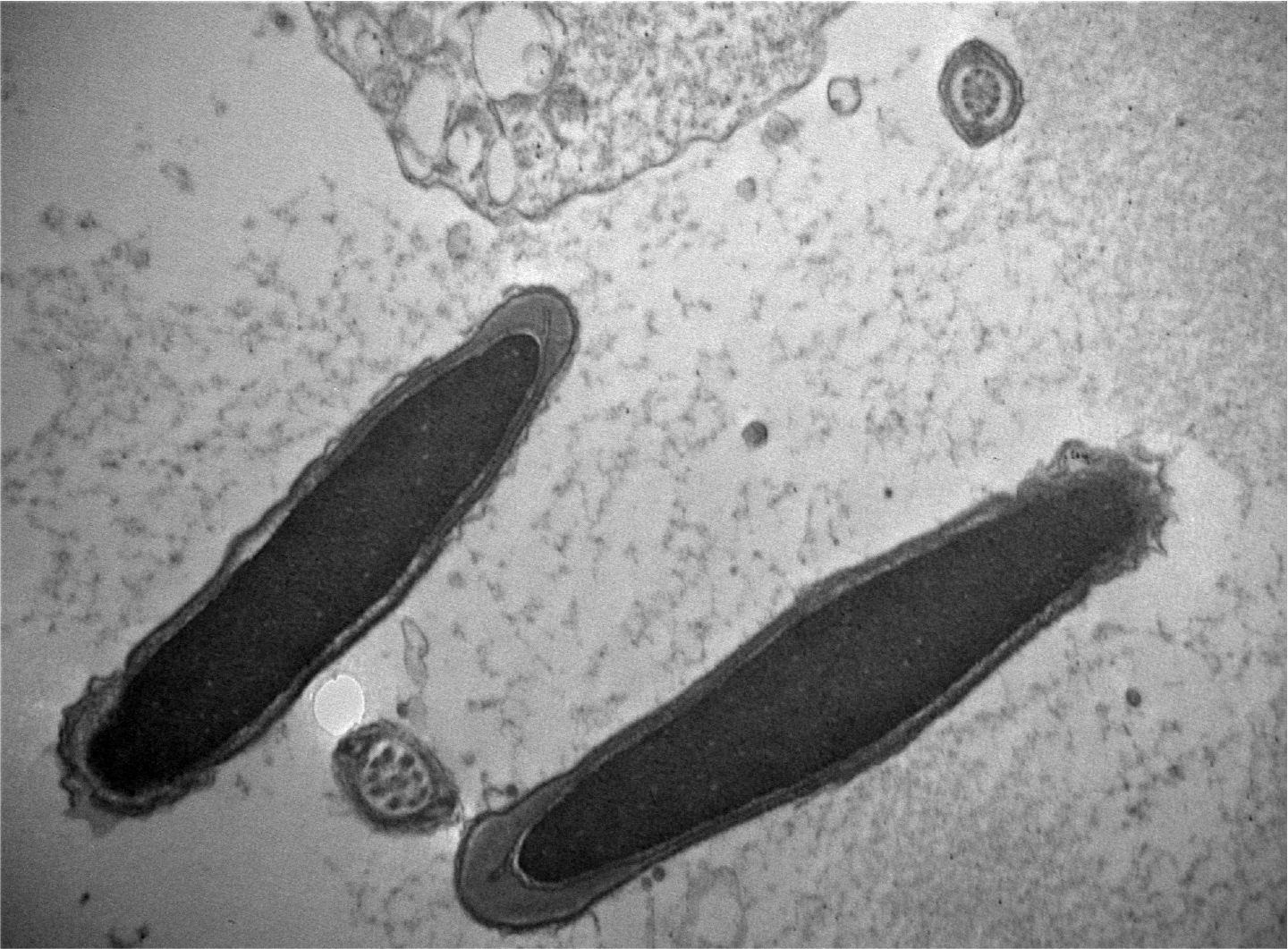

Supplement: Supplementary file 14 — Source data Fig. [file 44318_2025_659_MOESM14_ESM.zip › EMBOJ-2025-121587_Source Data/Source Data Figure 3/SD Figure 3A/Igf2bp3+/- 1.jpg]

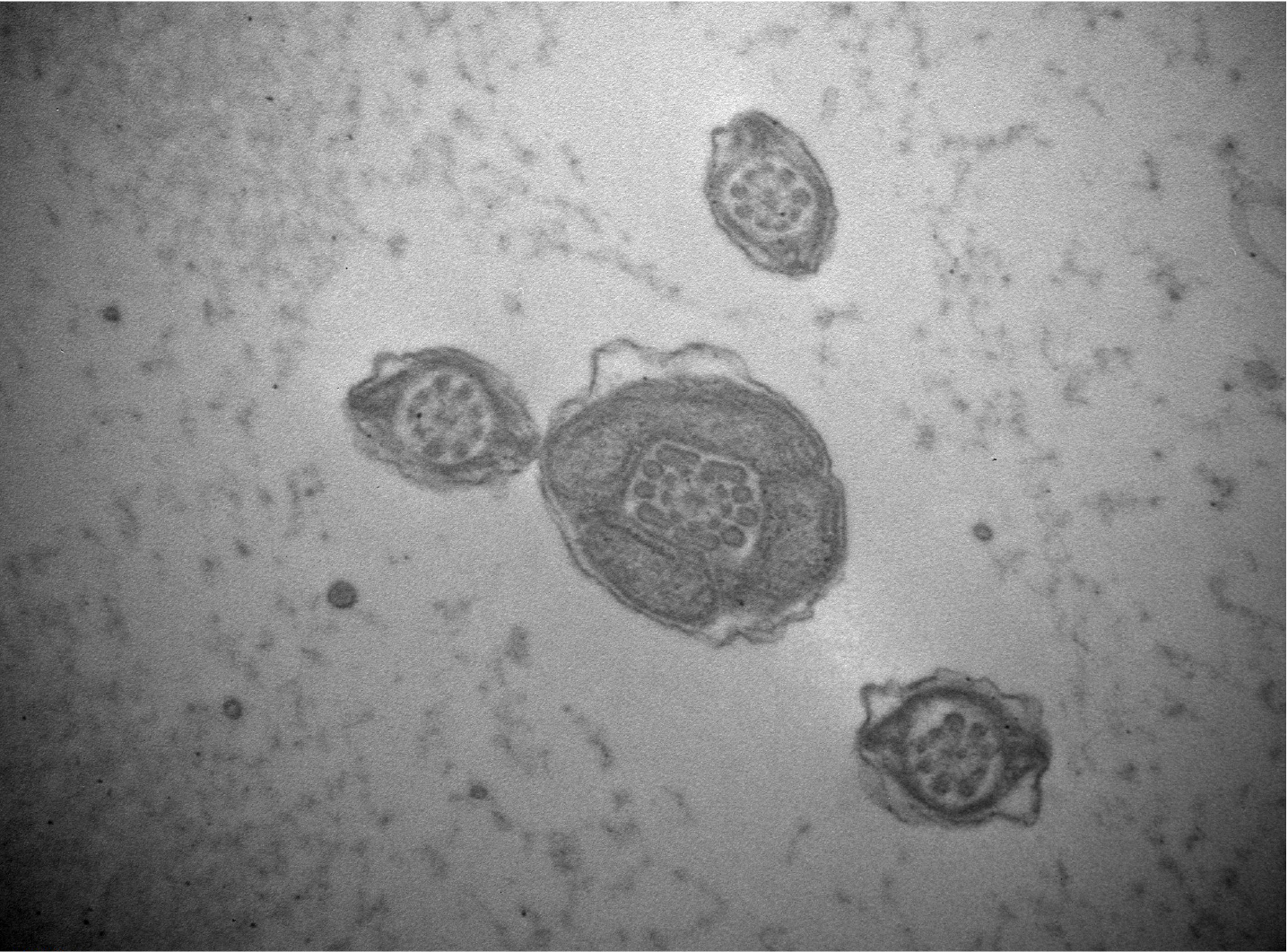

Supplement: Supplementary file 14 — Source data Fig. [file 44318_2025_659_MOESM14_ESM.zip › EMBOJ-2025-121587_Source Data/Source Data Figure 3/SD Figure 3A/Igf2bp3+/- 2.jpg]

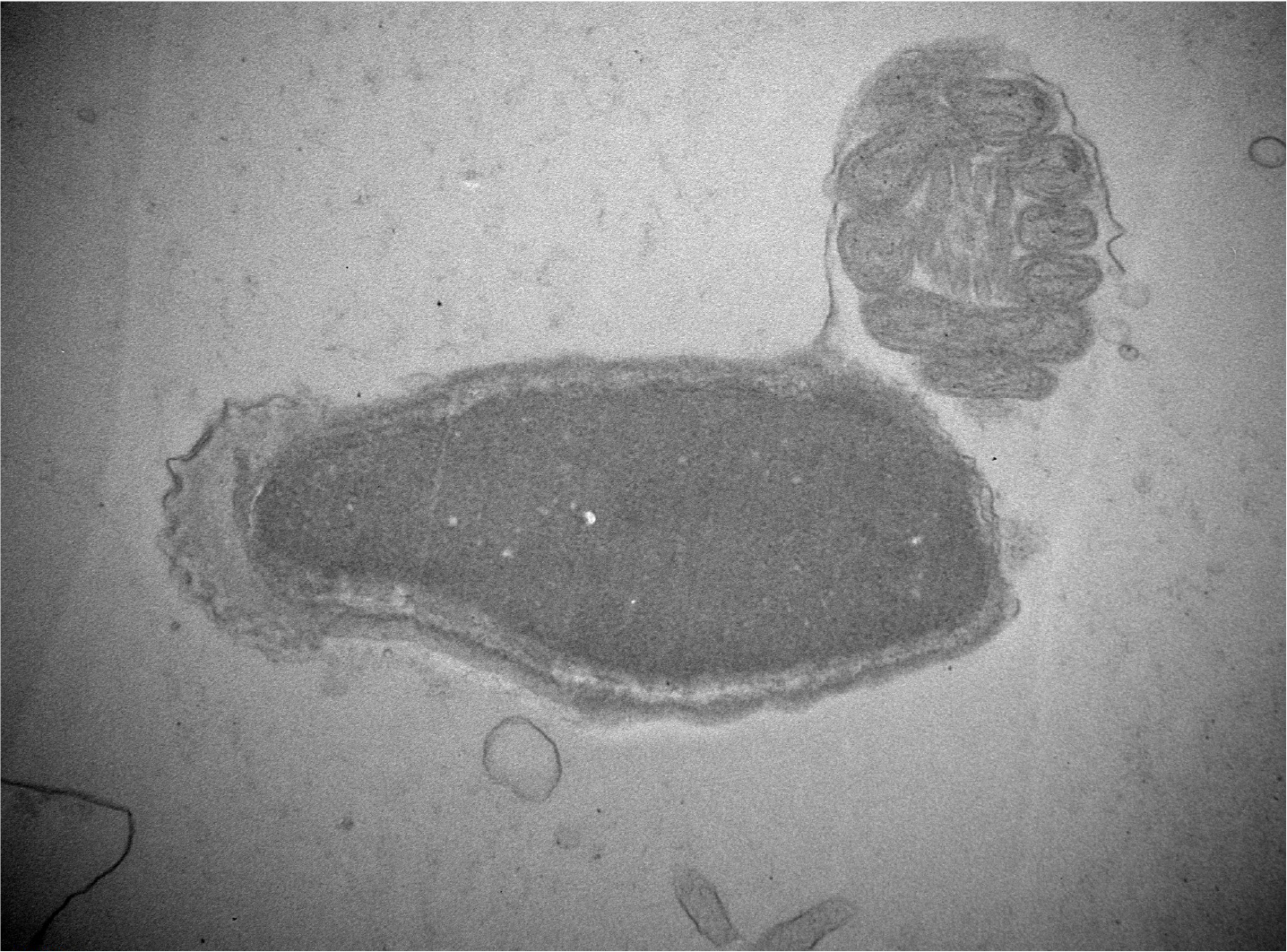

Supplement: Supplementary file 14 — Source data Fig. [file 44318_2025_659_MOESM14_ESM.zip › EMBOJ-2025-121587_Source Data/Source Data Figure 3/SD Figure 3A/Igf2bp3-/- 1.jpg]

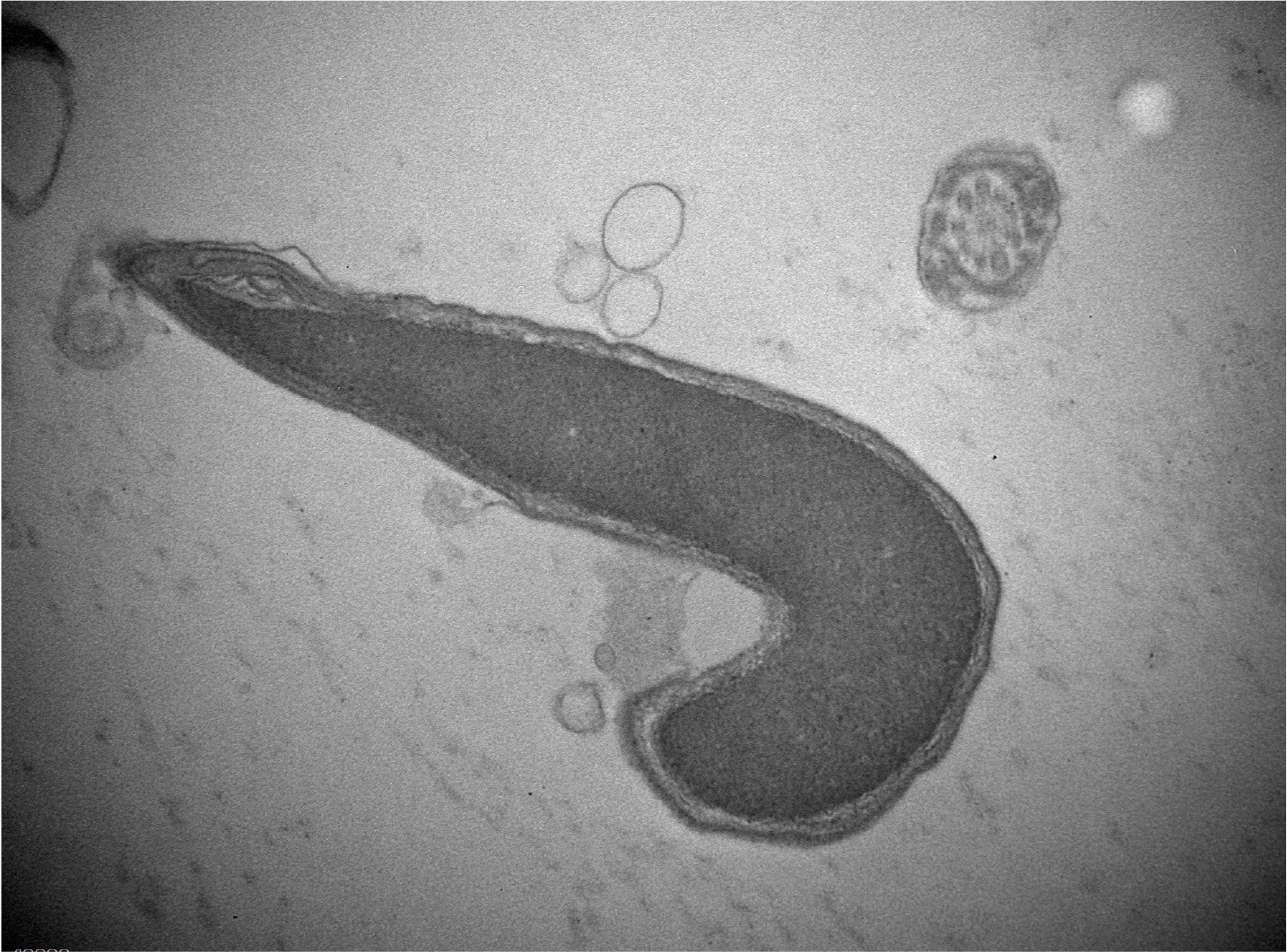

Supplement: Supplementary file 14 — Source data Fig. [file 44318_2025_659_MOESM14_ESM.zip › EMBOJ-2025-121587_Source Data/Source Data Figure 3/SD Figure 3A/Igf2bp3-/- 2.jpg]

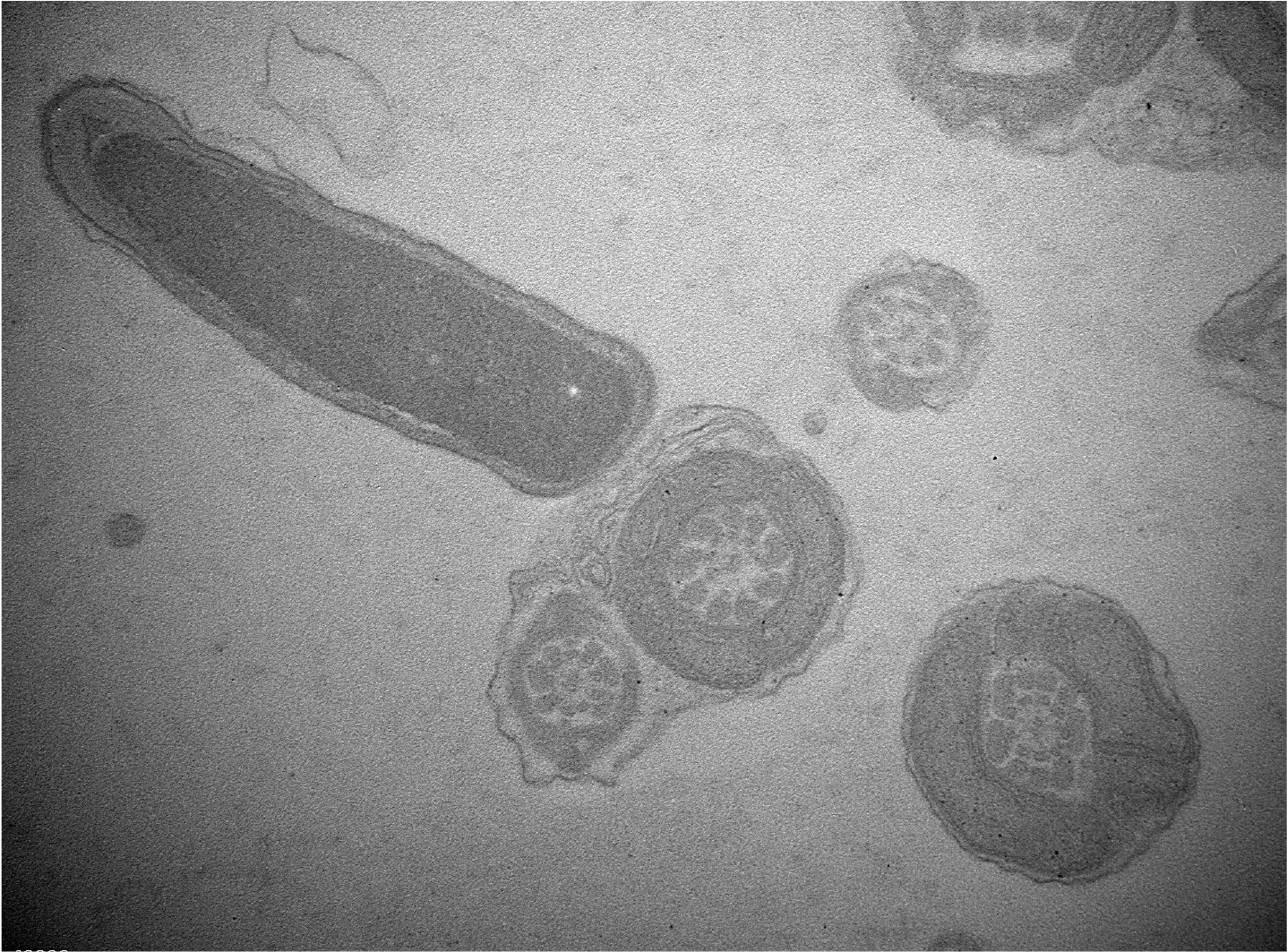

Supplement: Supplementary file 14 — Source data Fig. [file 44318_2025_659_MOESM14_ESM.zip › EMBOJ-2025-121587_Source Data/Source Data Figure 3/SD Figure 3A/Igf2bp3-/- 3.jpg]

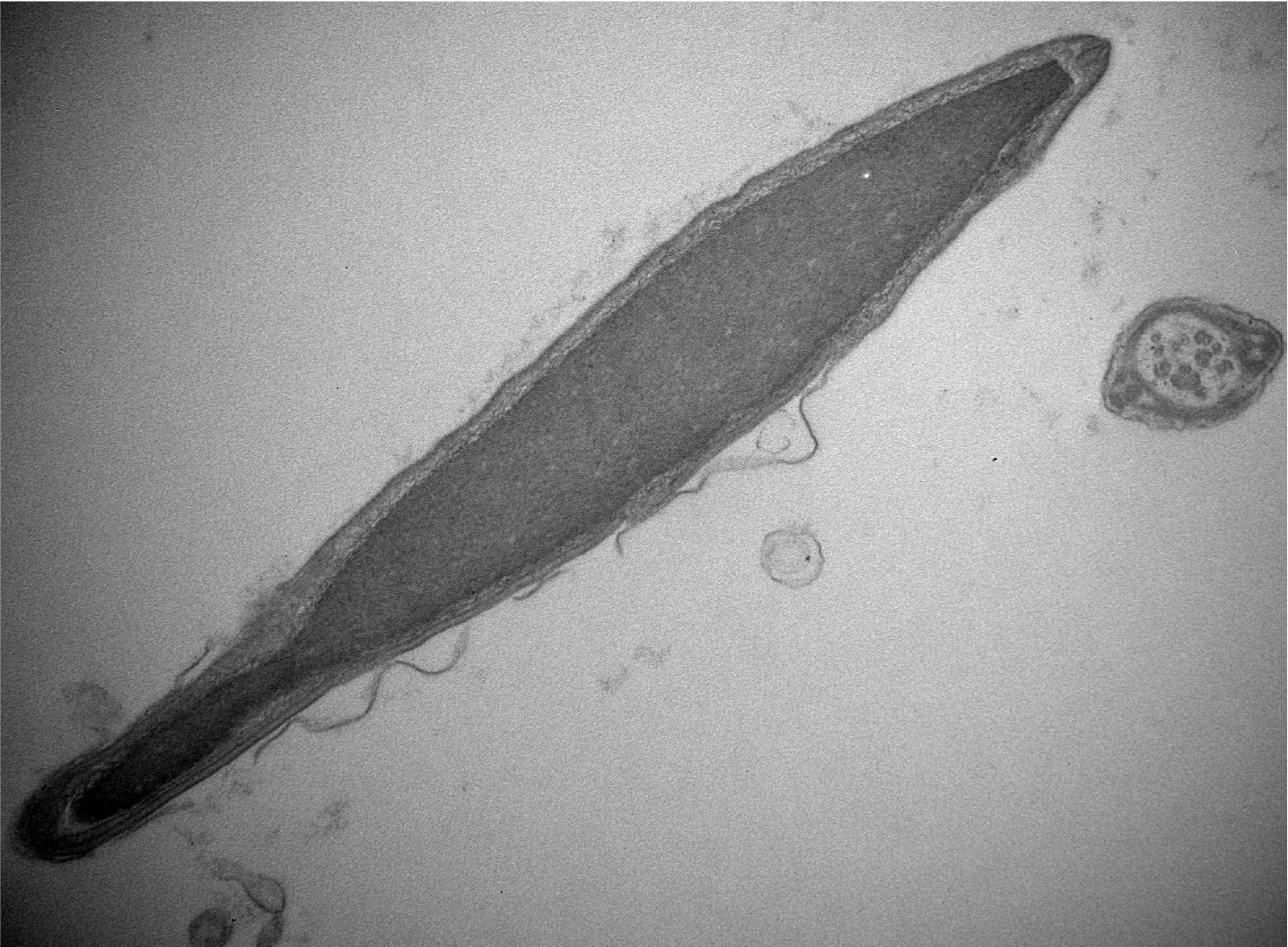

Supplement: Supplementary file 14 — Source data Fig. [file 44318_2025_659_MOESM14_ESM.zip › EMBOJ-2025-121587_Source Data/Source Data Figure 3/SD Figure 3A/Igf2bp3-/- 4.jpg]

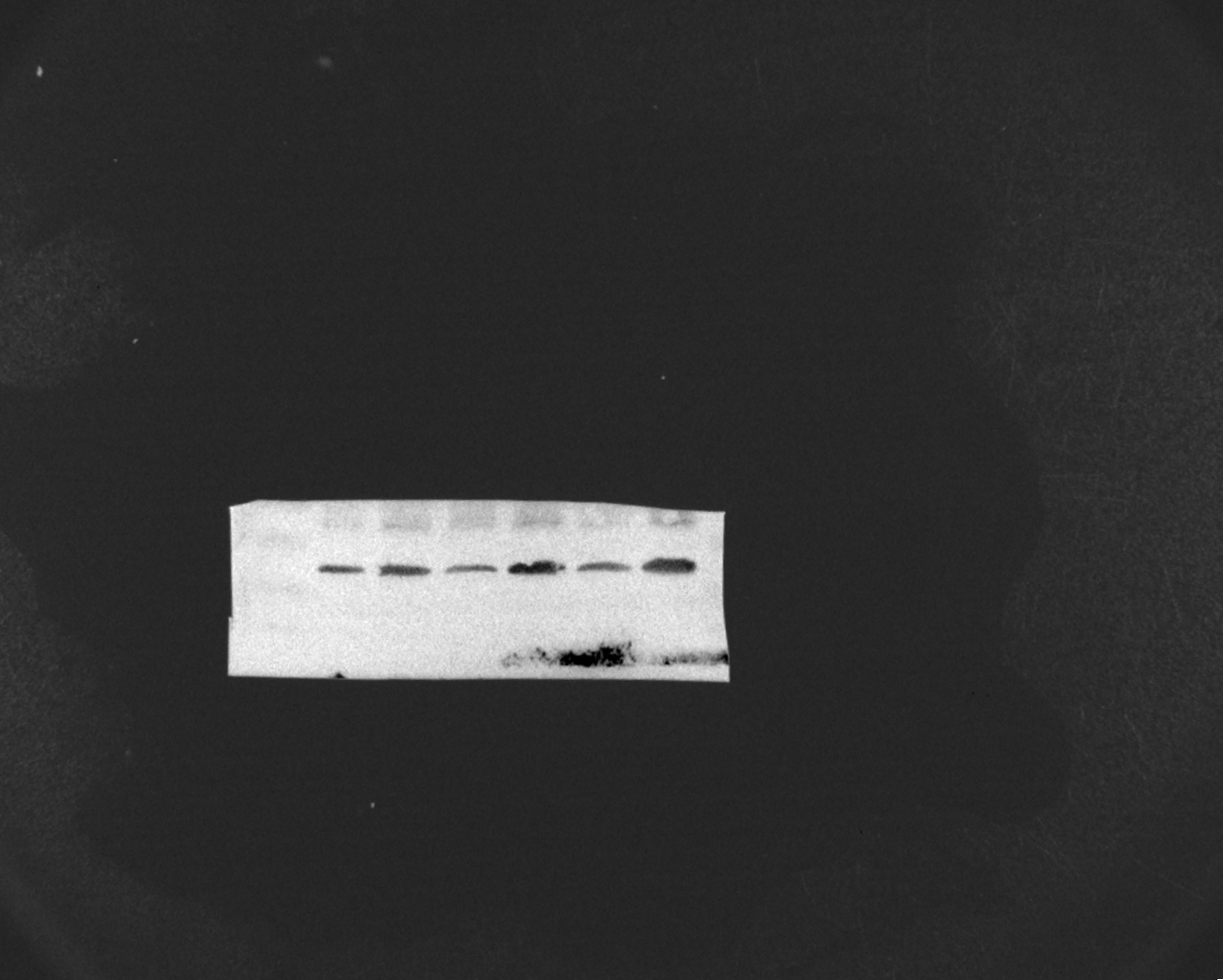

Supplement: Supplementary file 14 — Source data Fig. [file 44318_2025_659_MOESM14_ESM.zip › EMBOJ-2025-121587_Source Data/Source Data Figure 3/SD Figure 3B/SD Figure 3B-H2A.tif]

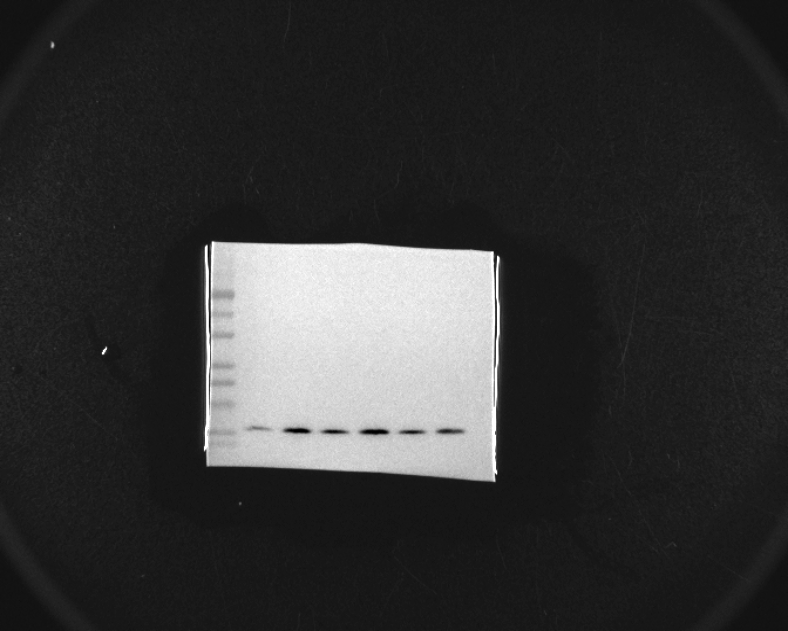

Supplement: Supplementary file 14 — Source data Fig. [file 44318_2025_659_MOESM14_ESM.zip › EMBOJ-2025-121587_Source Data/Source Data Figure 3/SD Figure 3B/SD Figure 3B-H2B.tif]

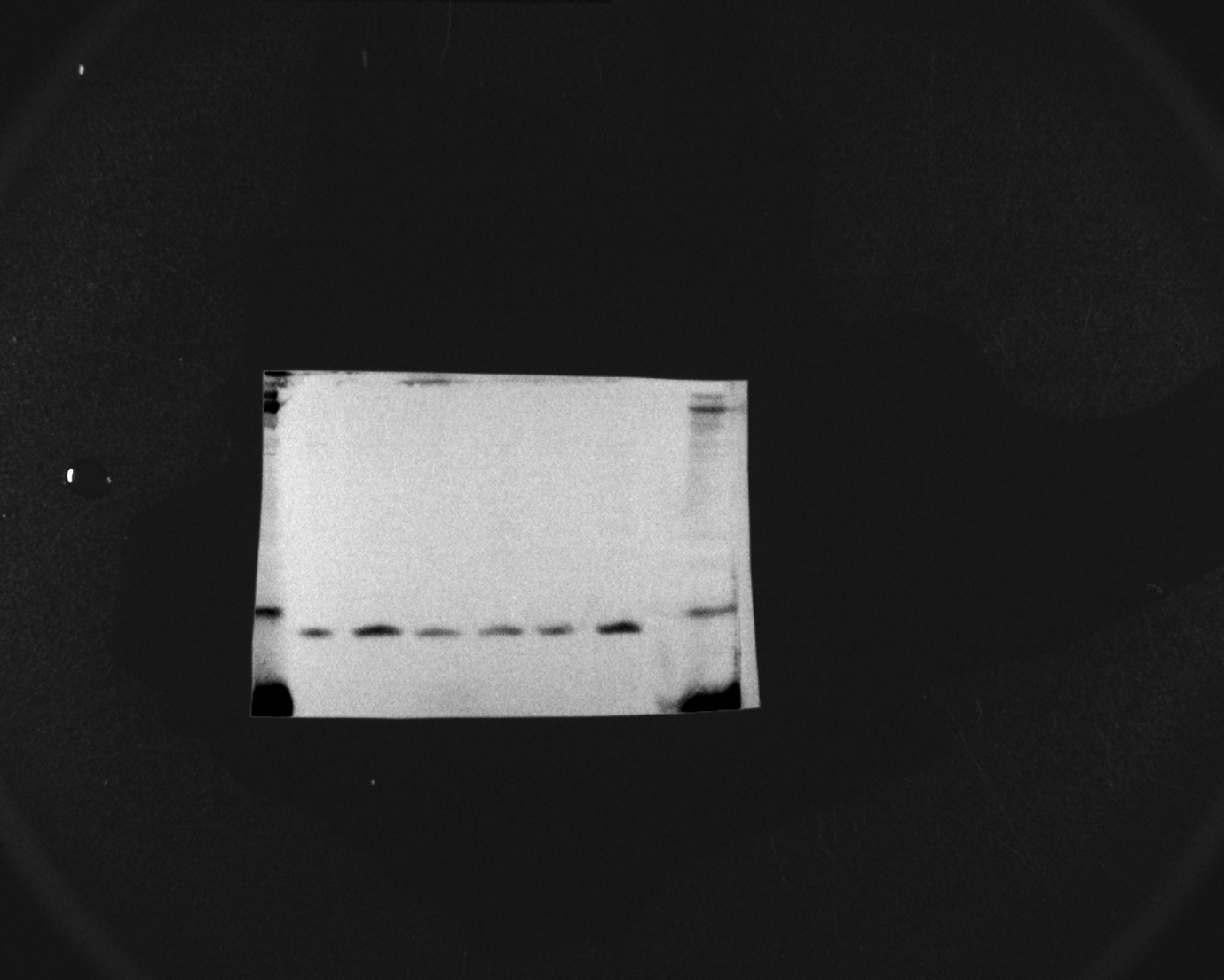

Supplement: Supplementary file 14 — Source data Fig. [file 44318_2025_659_MOESM14_ESM.zip › EMBOJ-2025-121587_Source Data/Source Data Figure 3/SD Figure 3B/SD Figure 3B-H3.tif]

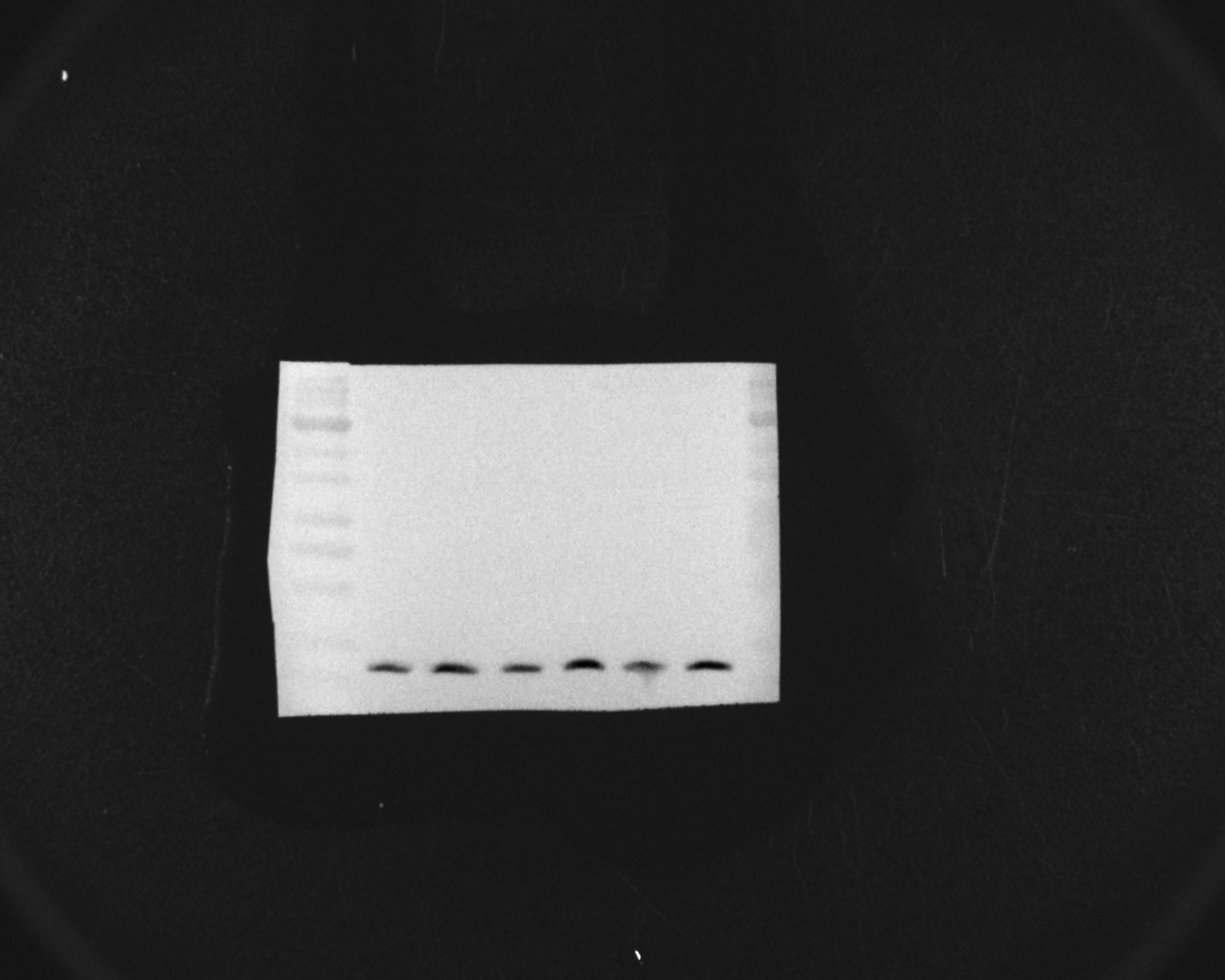

Supplement: Supplementary file 14 — Source data Fig. [file 44318_2025_659_MOESM14_ESM.zip › EMBOJ-2025-121587_Source Data/Source Data Figure 3/SD Figure 3B/SD Figure 3B-H4.tif]

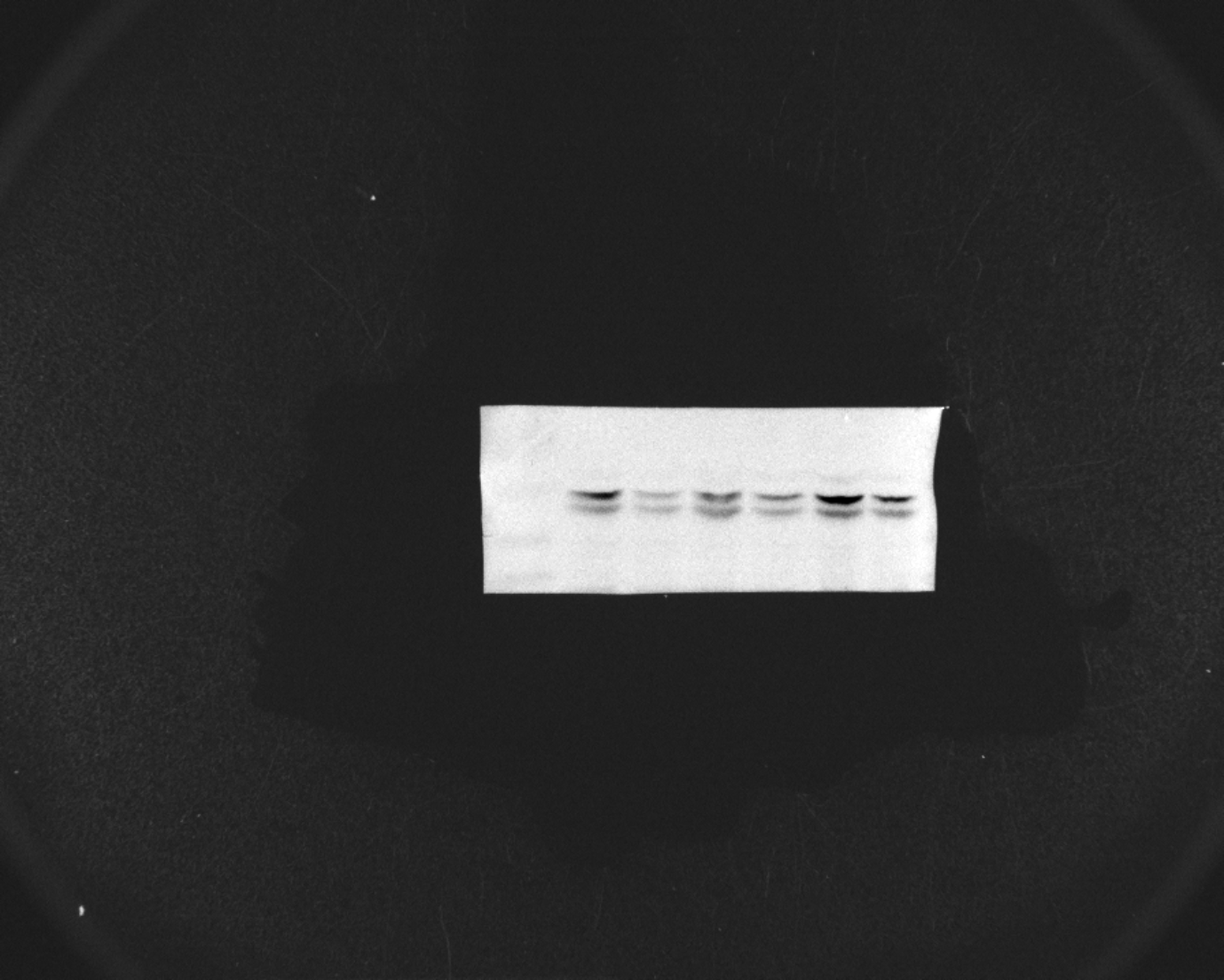

Supplement: Supplementary file 14 — Source data Fig. [file 44318_2025_659_MOESM14_ESM.zip › EMBOJ-2025-121587_Source Data/Source Data Figure 3/SD Figure 3B/SD Figure 3B-PRM1.tif]

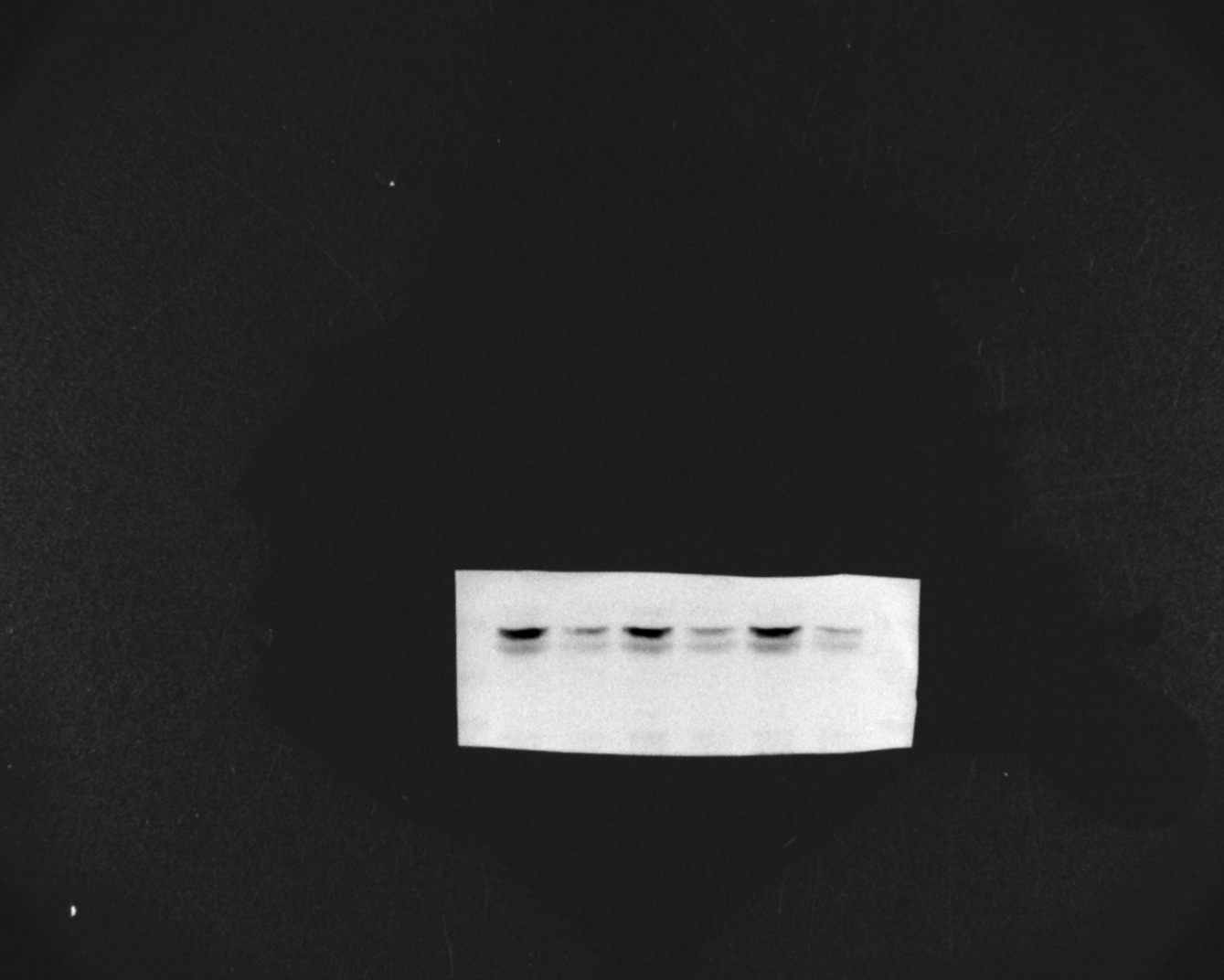

Supplement: Supplementary file 14 — Source data Fig. [file 44318_2025_659_MOESM14_ESM.zip › EMBOJ-2025-121587_Source Data/Source Data Figure 3/SD Figure 3B/SD Figure 3B-PRM2.tif]

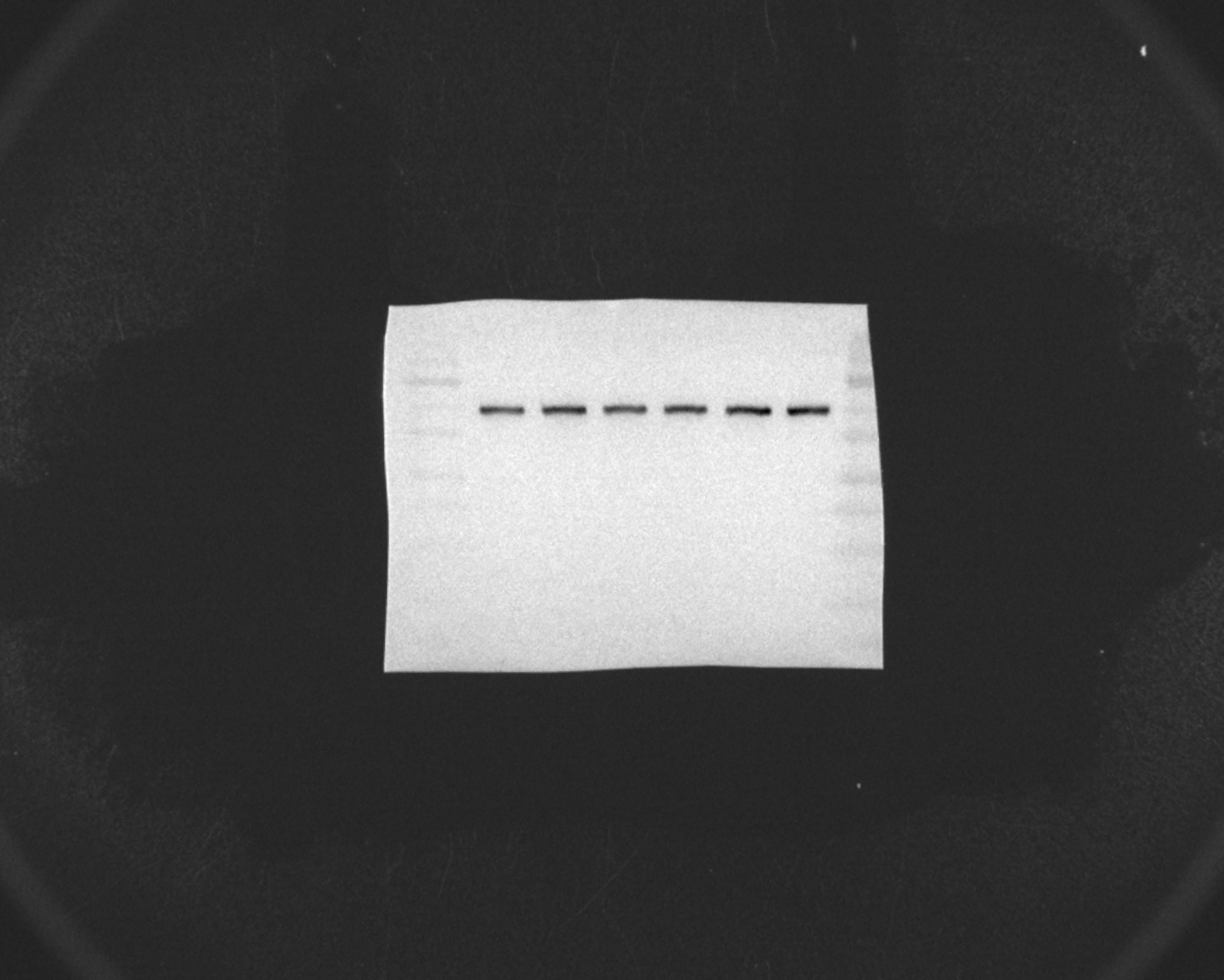

Supplement: Supplementary file 14 — Source data Fig. [file 44318_2025_659_MOESM14_ESM.zip › EMBOJ-2025-121587_Source Data/Source Data Figure 3/SD Figure 3B/SD Figure 3B-TUBLIN.tif]

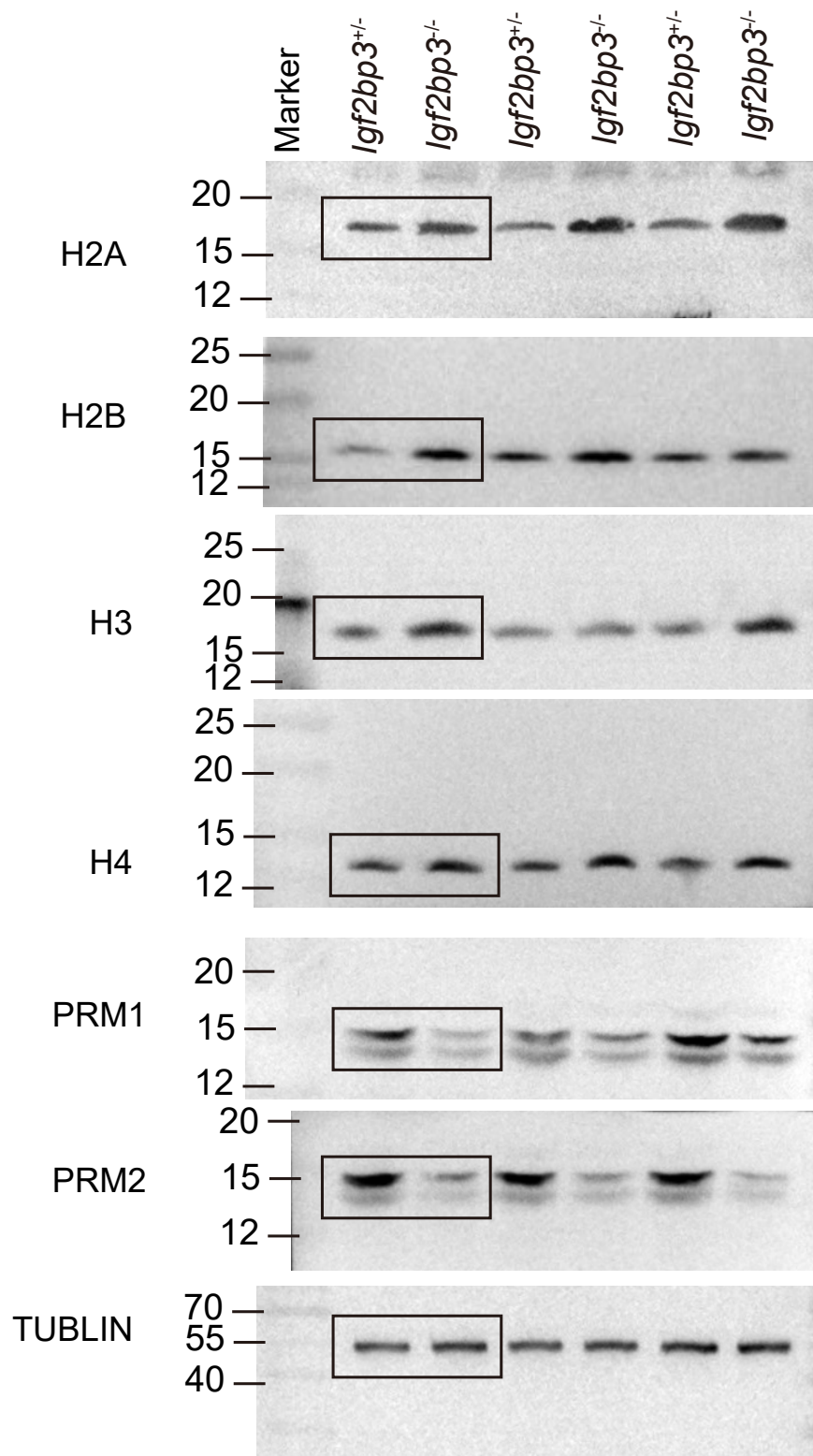

Supplement: Supplementary file 14 — Source data Fig. [file 44318_2025_659_MOESM14_ESM.zip › EMBOJ-2025-121587_Source Data/Source Data Figure 3/SD Figure 3B/SD Figure 3B.pdf]

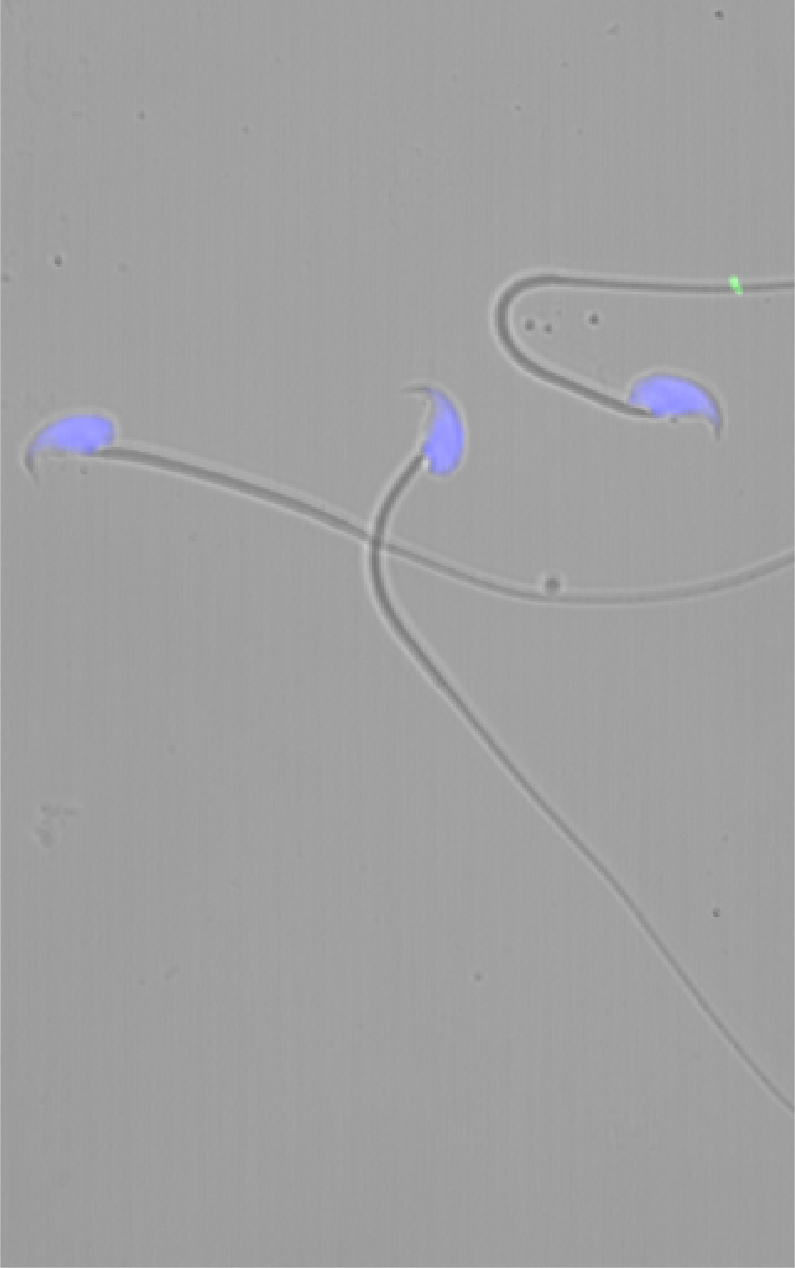

Supplement: Supplementary file 14 — Source data Fig. [file 44318_2025_659_MOESM14_ESM.zip › EMBOJ-2025-121587_Source Data/Source Data Figure 3/SD Figure 3C/Igf2bp3+/- H2A.jpg]

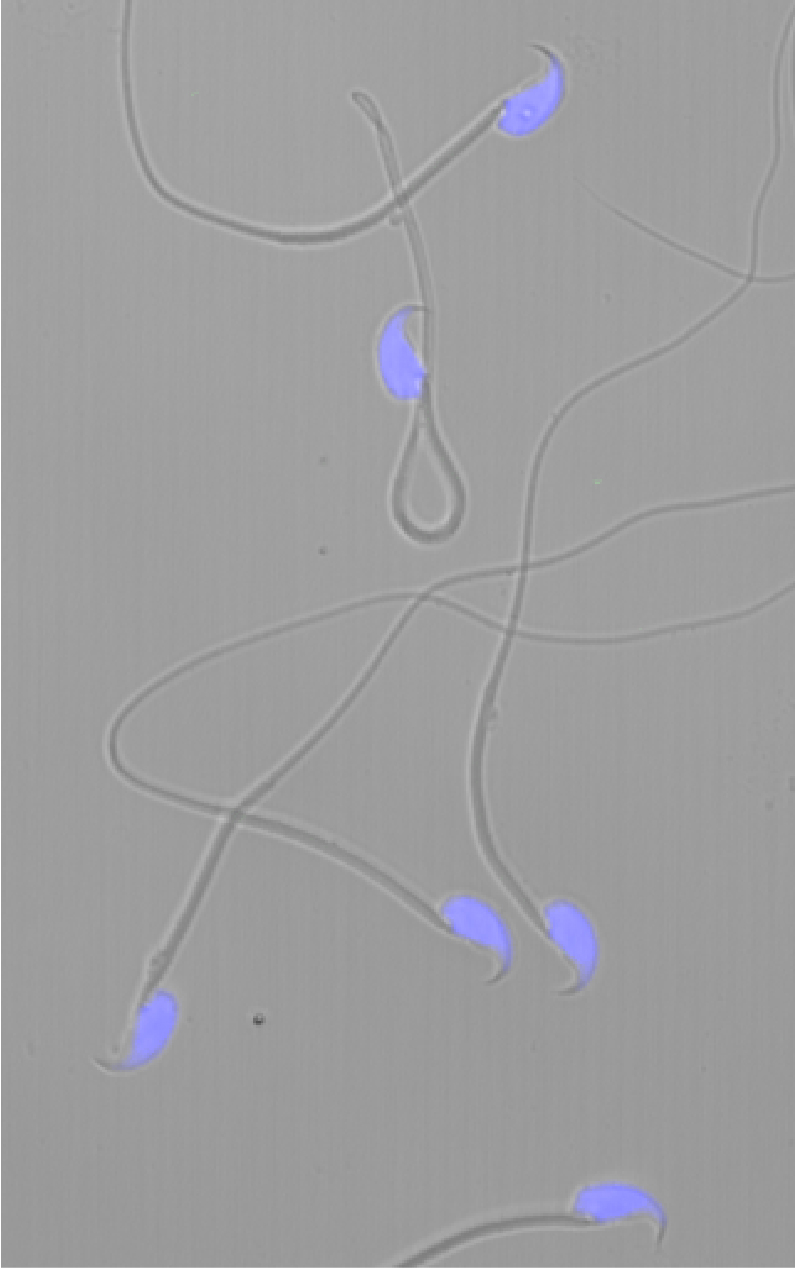

Supplement: Supplementary file 14 — Source data Fig. [file 44318_2025_659_MOESM14_ESM.zip › EMBOJ-2025-121587_Source Data/Source Data Figure 3/SD Figure 3C/Igf2bp3+/- H2B.jpg]

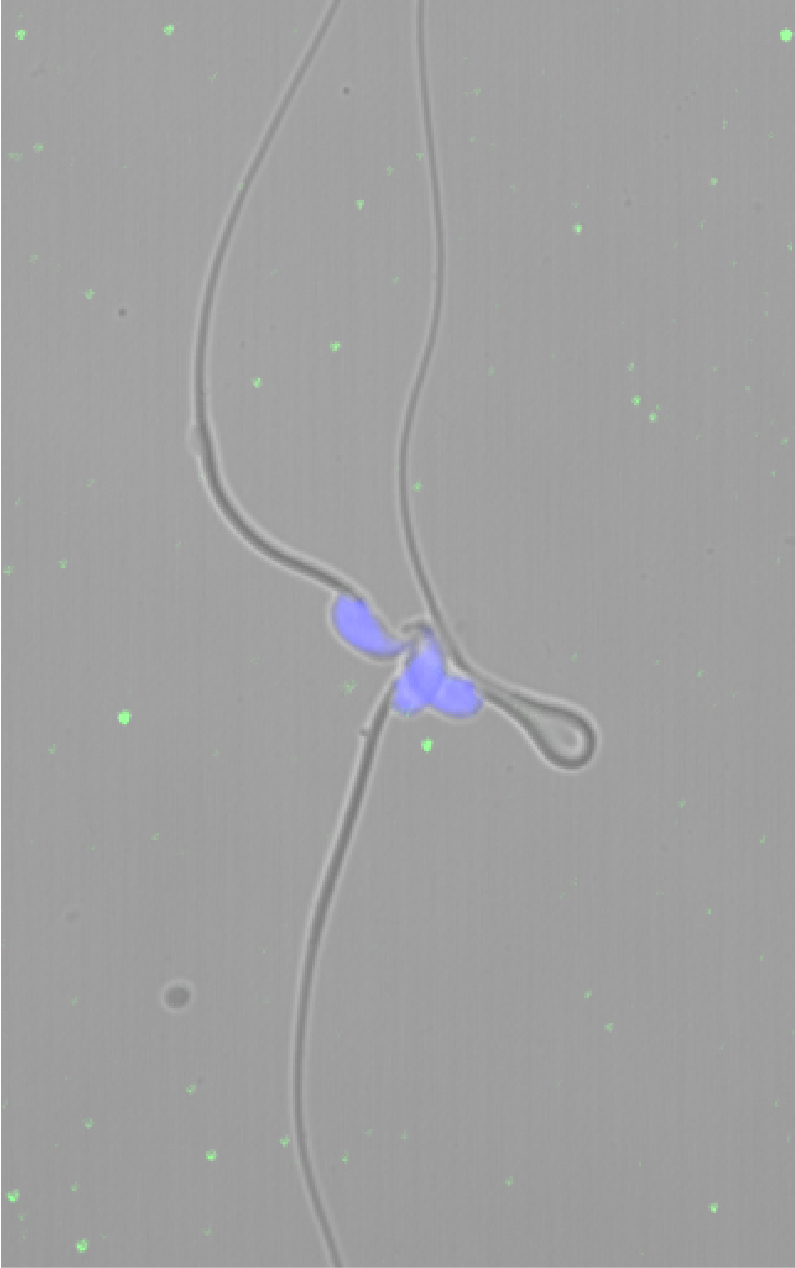

Supplement: Supplementary file 14 — Source data Fig. [file 44318_2025_659_MOESM14_ESM.zip › EMBOJ-2025-121587_Source Data/Source Data Figure 3/SD Figure 3C/Igf2bp3+/- H3.jpg]

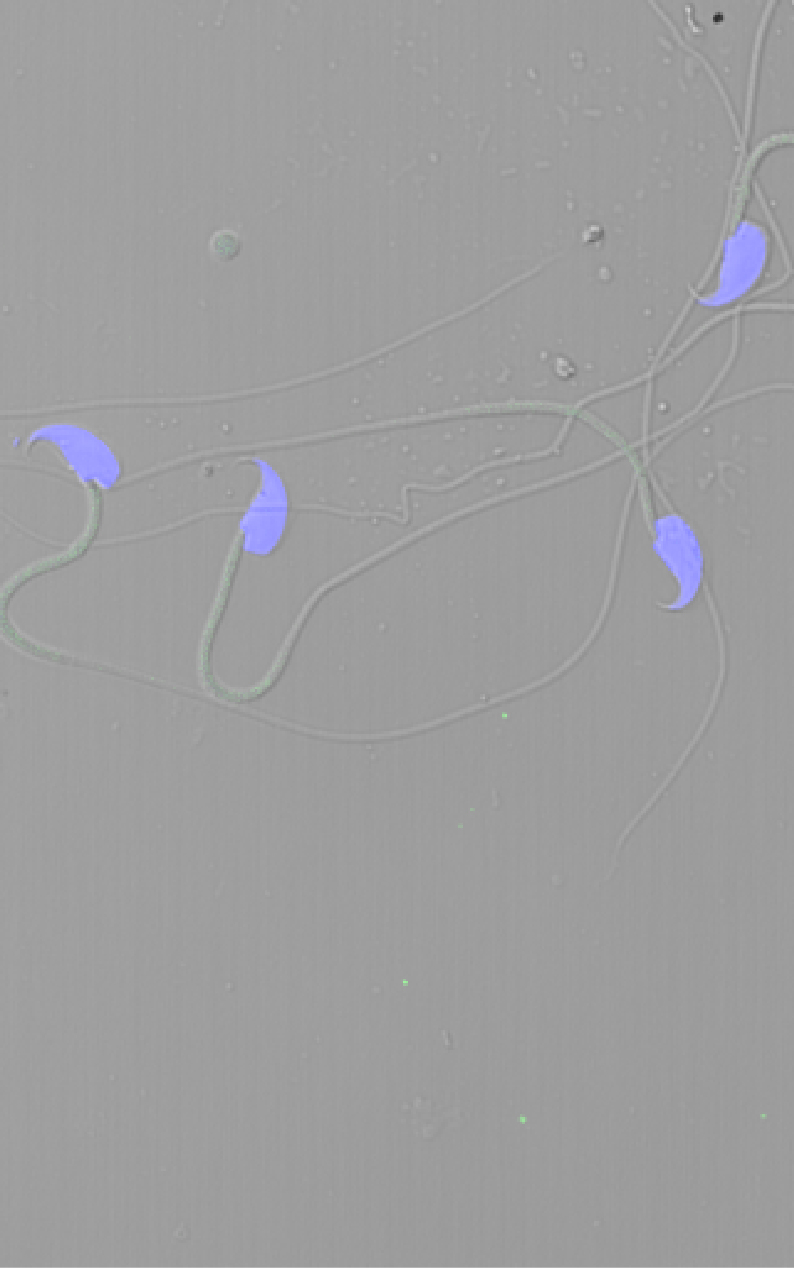

Supplement: Supplementary file 14 — Source data Fig. [file 44318_2025_659_MOESM14_ESM.zip › EMBOJ-2025-121587_Source Data/Source Data Figure 3/SD Figure 3C/Igf2bp3+/- H4.jpg]

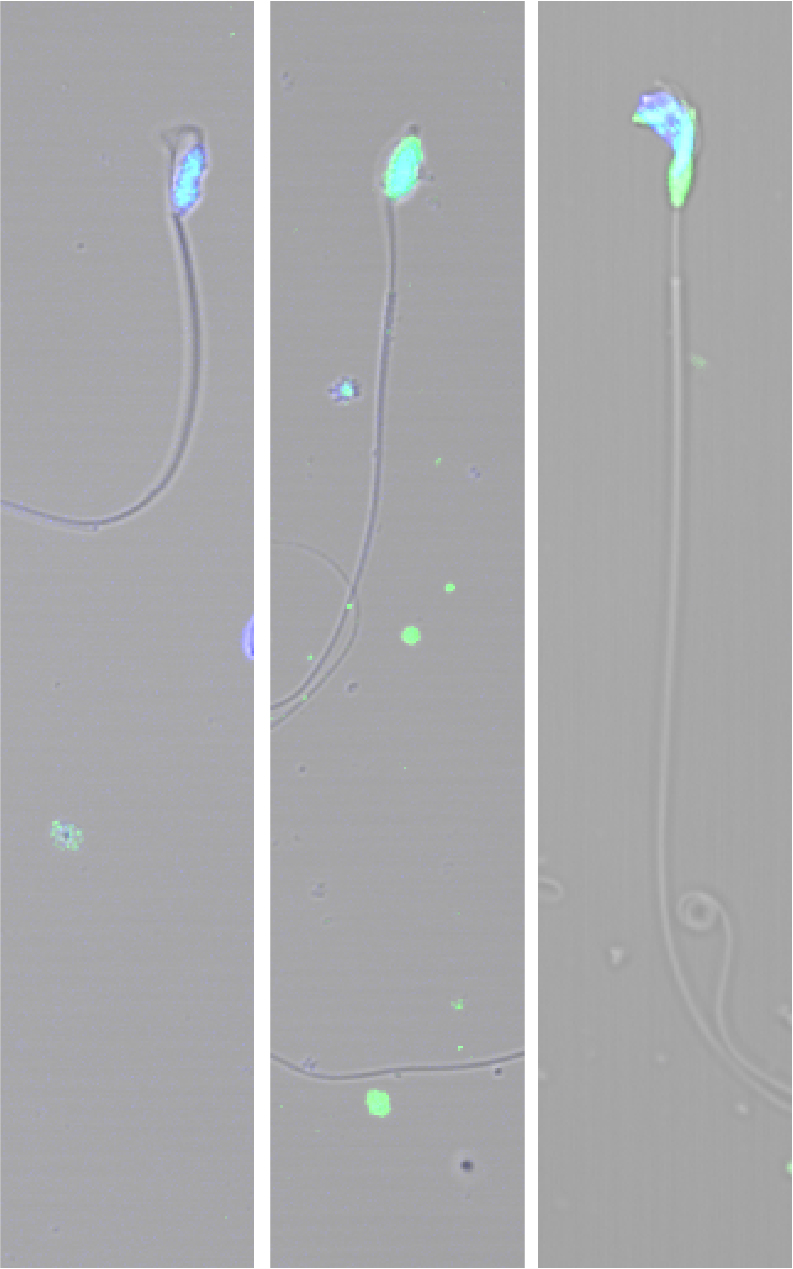

Supplement: Supplementary file 14 — Source data Fig. [file 44318_2025_659_MOESM14_ESM.zip › EMBOJ-2025-121587_Source Data/Source Data Figure 3/SD Figure 3C/Igf2bp3-/- H2A.jpg]

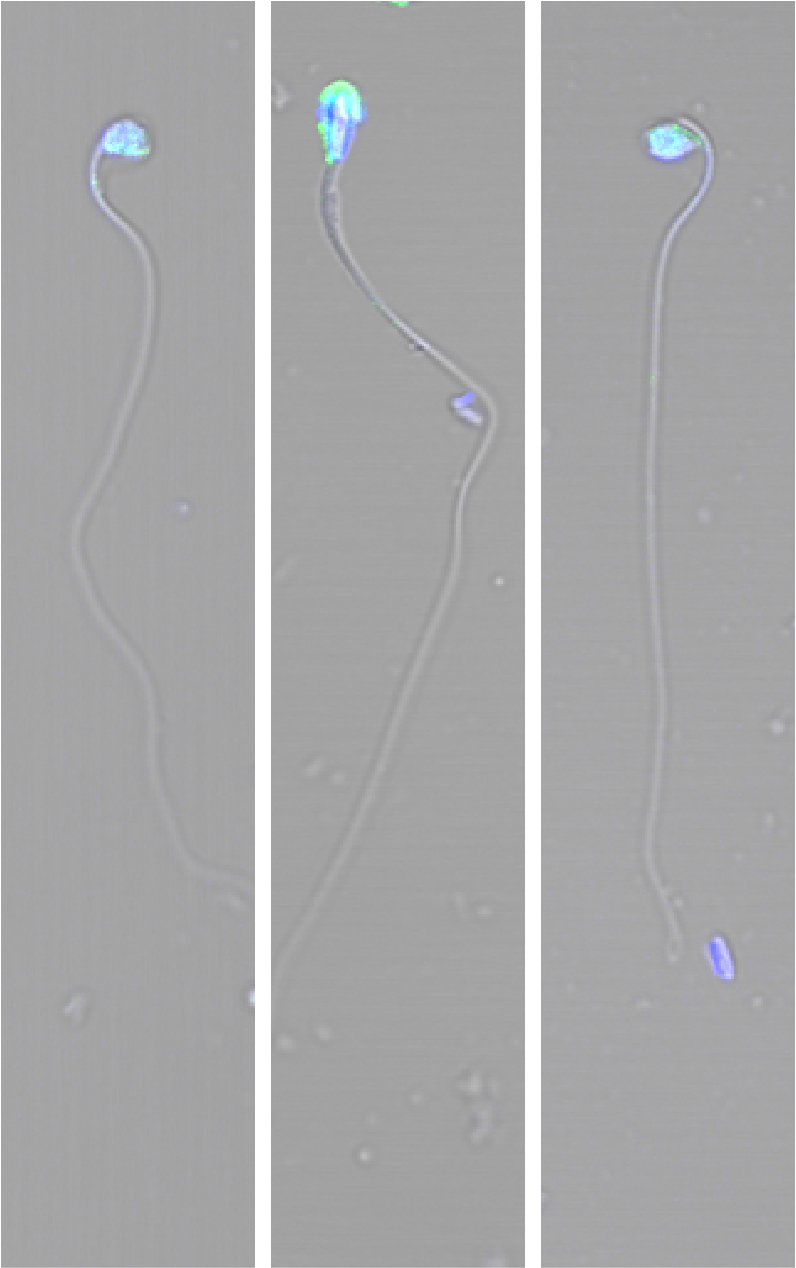

Supplement: Supplementary file 14 — Source data Fig. [file 44318_2025_659_MOESM14_ESM.zip › EMBOJ-2025-121587_Source Data/Source Data Figure 3/SD Figure 3C/Igf2bp3-/- H2B.jpg]

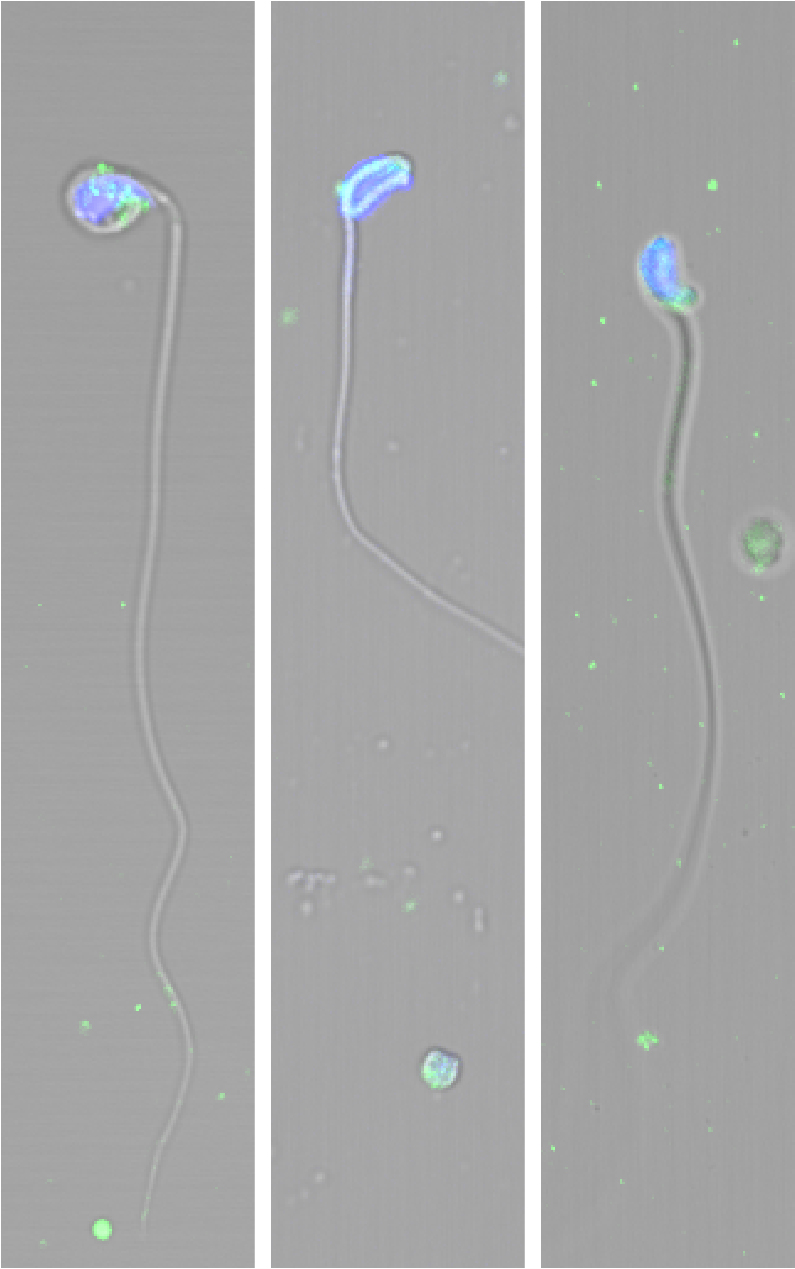

Supplement: Supplementary file 14 — Source data Fig. [file 44318_2025_659_MOESM14_ESM.zip › EMBOJ-2025-121587_Source Data/Source Data Figure 3/SD Figure 3C/Igf2bp3-/- H3.jpg]

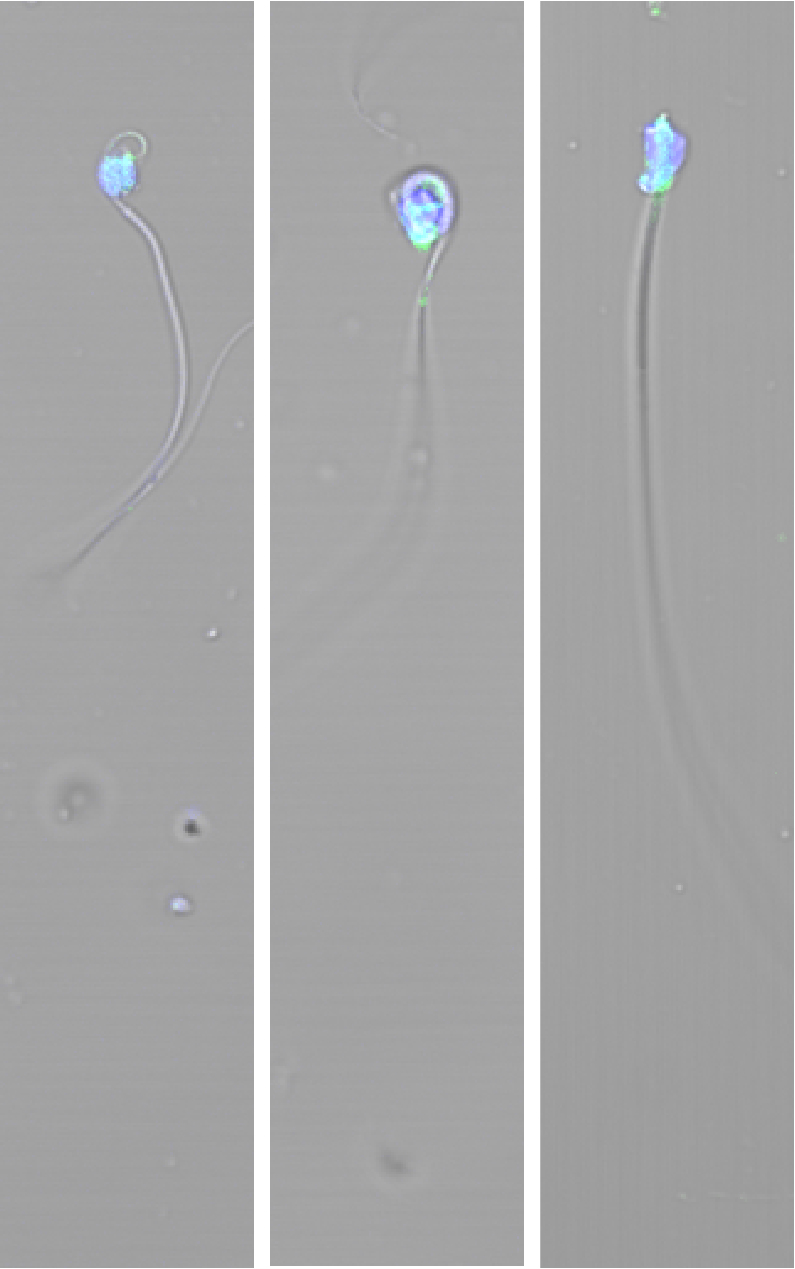

Supplement: Supplementary file 14 — Source data Fig. [file 44318_2025_659_MOESM14_ESM.zip › EMBOJ-2025-121587_Source Data/Source Data Figure 3/SD Figure 3C/Igf2bp3-/- H4.jpg]

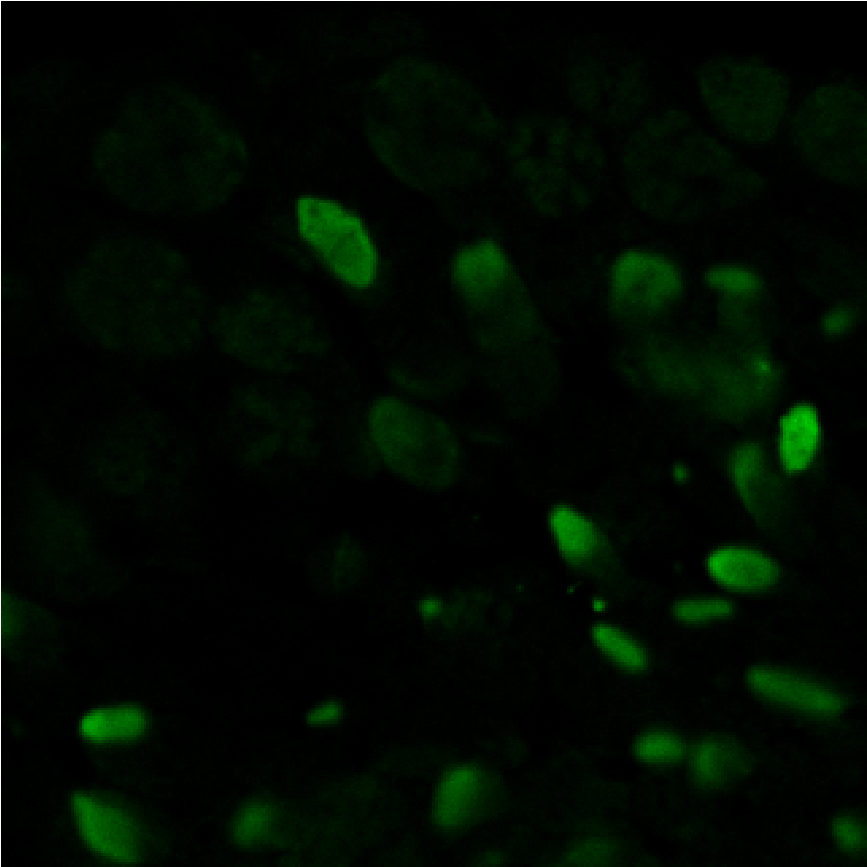

Supplement: Supplementary file 14 — Source data Fig. [file 44318_2025_659_MOESM14_ESM.zip › EMBOJ-2025-121587_Source Data/Source Data Figure 3/SD Figure 3E/Igf2bp3+/- H4Ac.jpg]

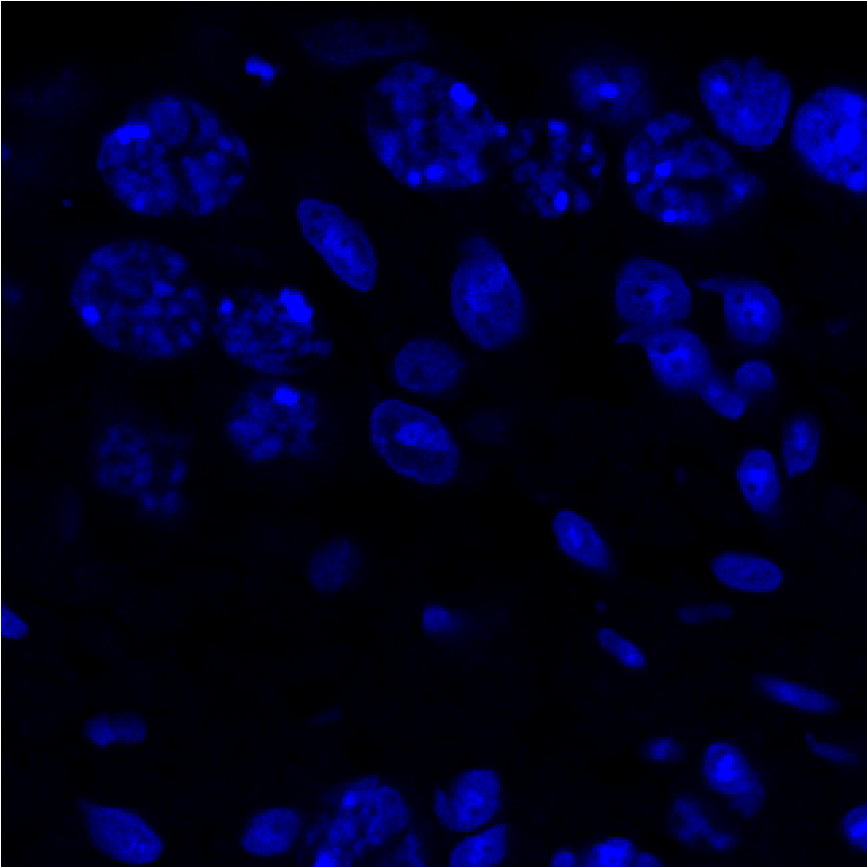

Supplement: Supplementary file 14 — Source data Fig. [file 44318_2025_659_MOESM14_ESM.zip › EMBOJ-2025-121587_Source Data/Source Data Figure 3/SD Figure 3E/Igf2bp3+/- Hoechst.jpg]

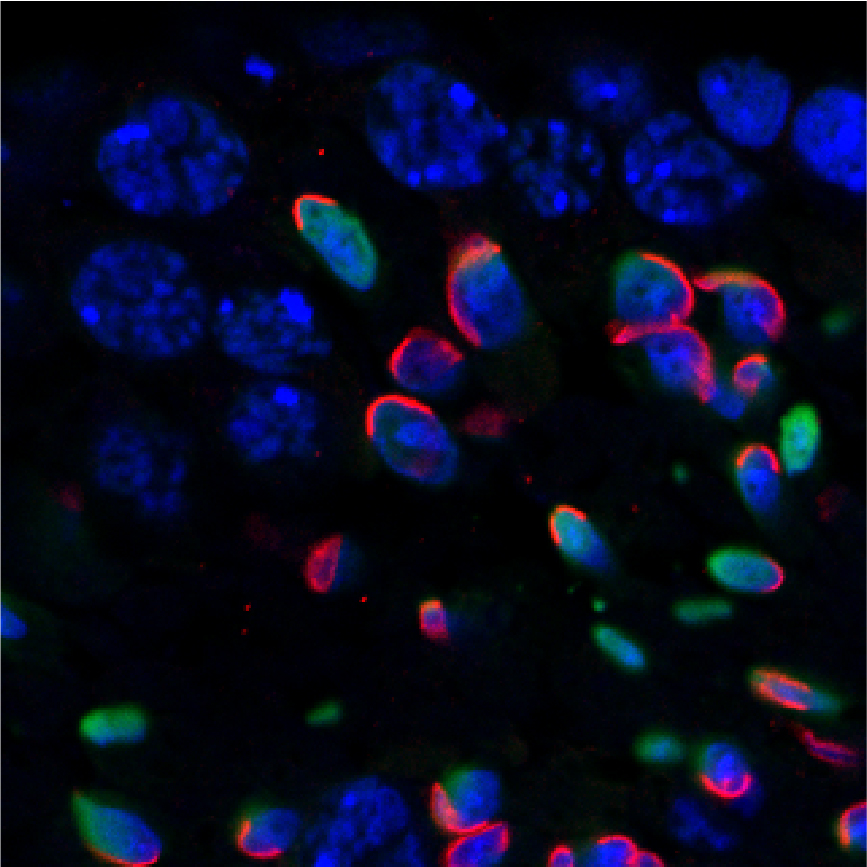

Supplement: Supplementary file 14 — Source data Fig. [file 44318_2025_659_MOESM14_ESM.zip › EMBOJ-2025-121587_Source Data/Source Data Figure 3/SD Figure 3E/Igf2bp3+/- Merge.jpg]

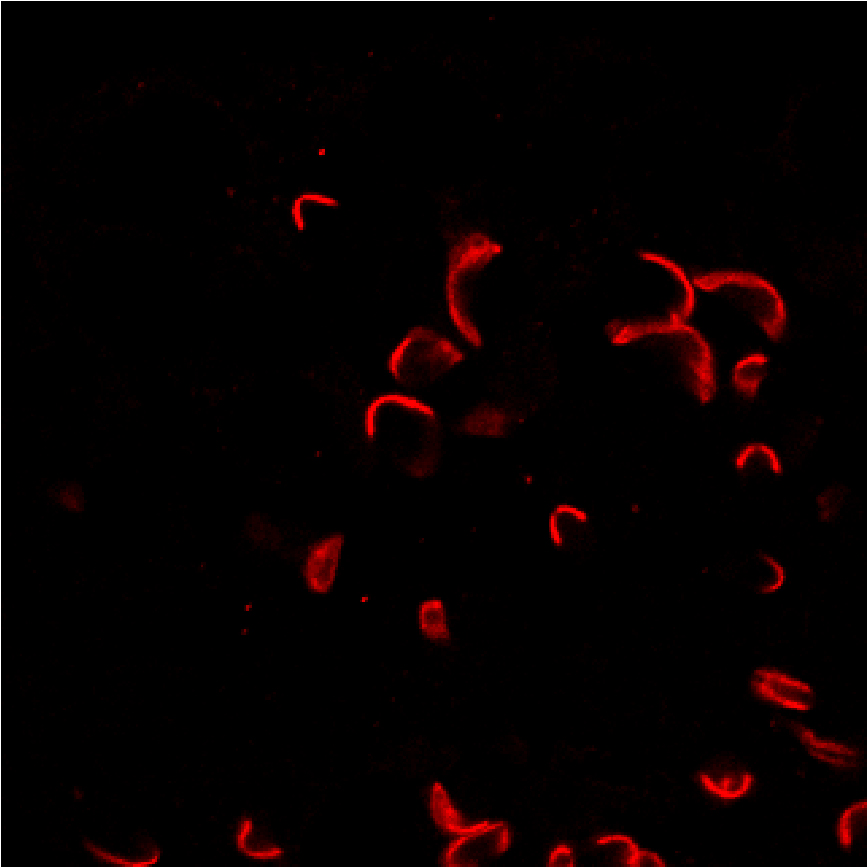

Supplement: Supplementary file 14 — Source data Fig. [file 44318_2025_659_MOESM14_ESM.zip › EMBOJ-2025-121587_Source Data/Source Data Figure 3/SD Figure 3E/Igf2bp3+/- PNA.jpg]

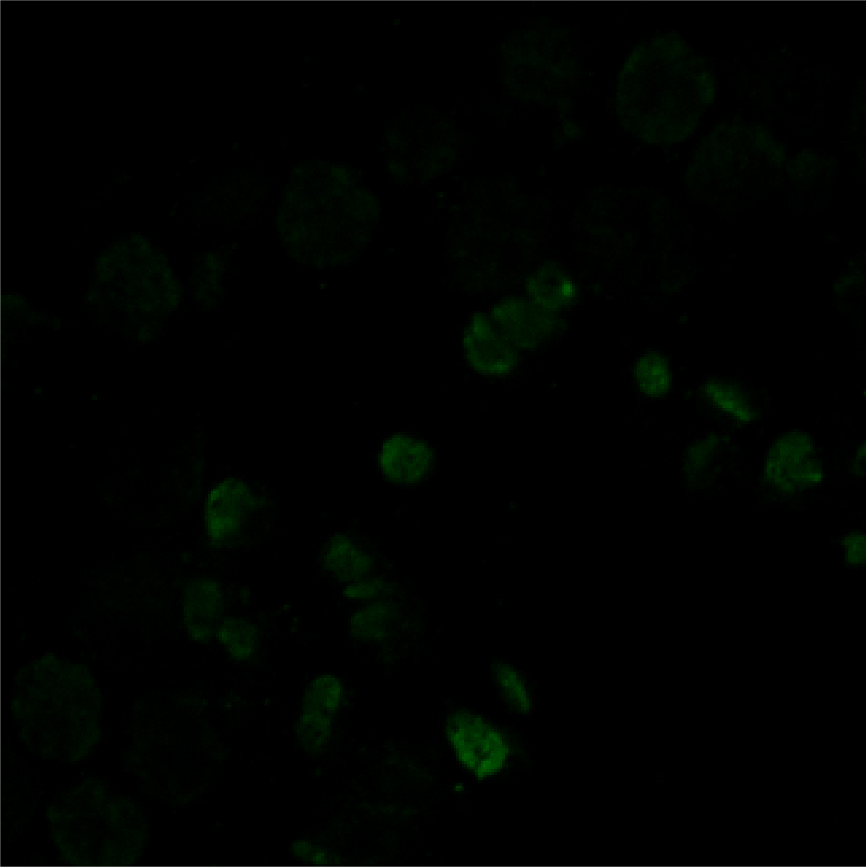

Supplement: Supplementary file 14 — Source data Fig. [file 44318_2025_659_MOESM14_ESM.zip › EMBOJ-2025-121587_Source Data/Source Data Figure 3/SD Figure 3E/Igf2bp3-/- H4Ac.jpg]

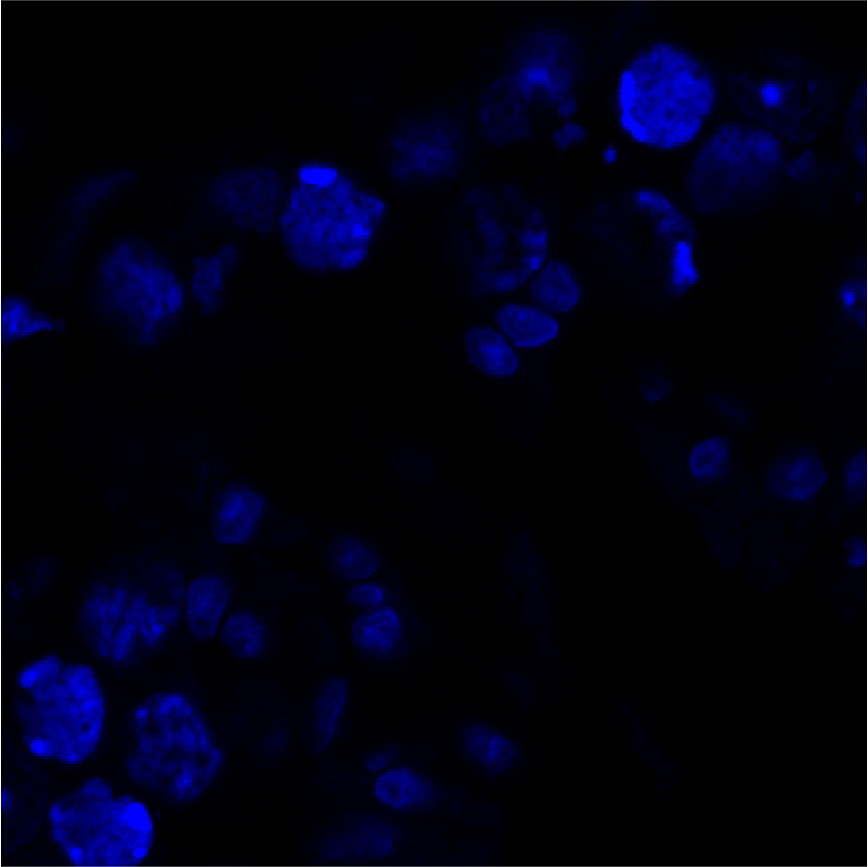

Supplement: Supplementary file 14 — Source data Fig. [file 44318_2025_659_MOESM14_ESM.zip › EMBOJ-2025-121587_Source Data/Source Data Figure 3/SD Figure 3E/Igf2bp3-/- Hoechst.jpg]

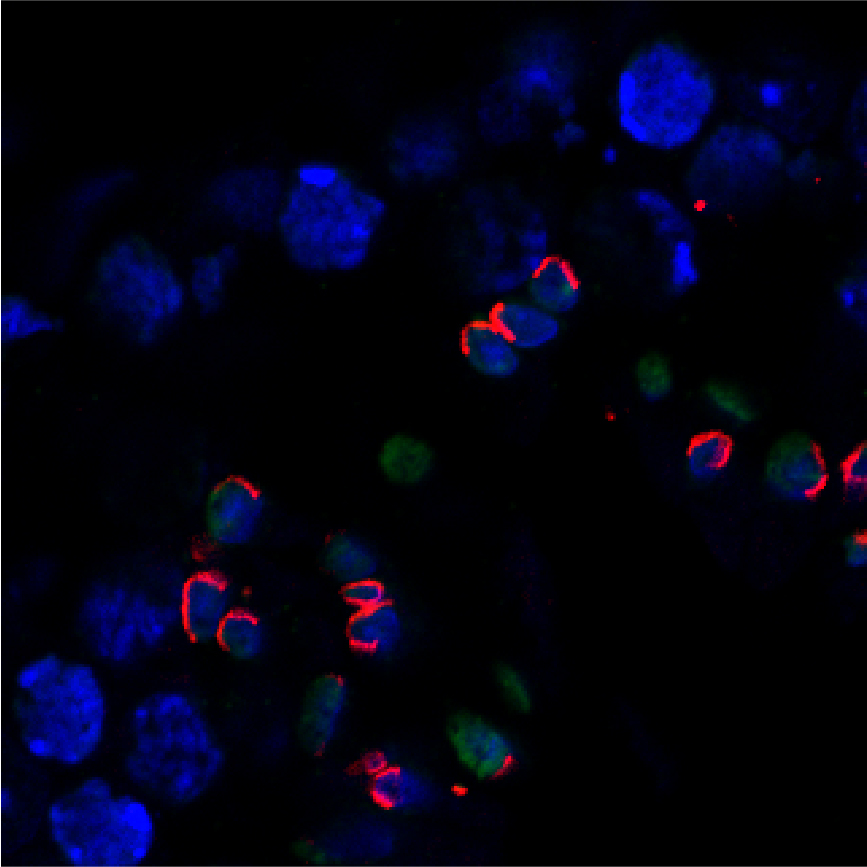

Supplement: Supplementary file 14 — Source data Fig. [file 44318_2025_659_MOESM14_ESM.zip › EMBOJ-2025-121587_Source Data/Source Data Figure 3/SD Figure 3E/Igf2bp3-/- Merge.jpg]

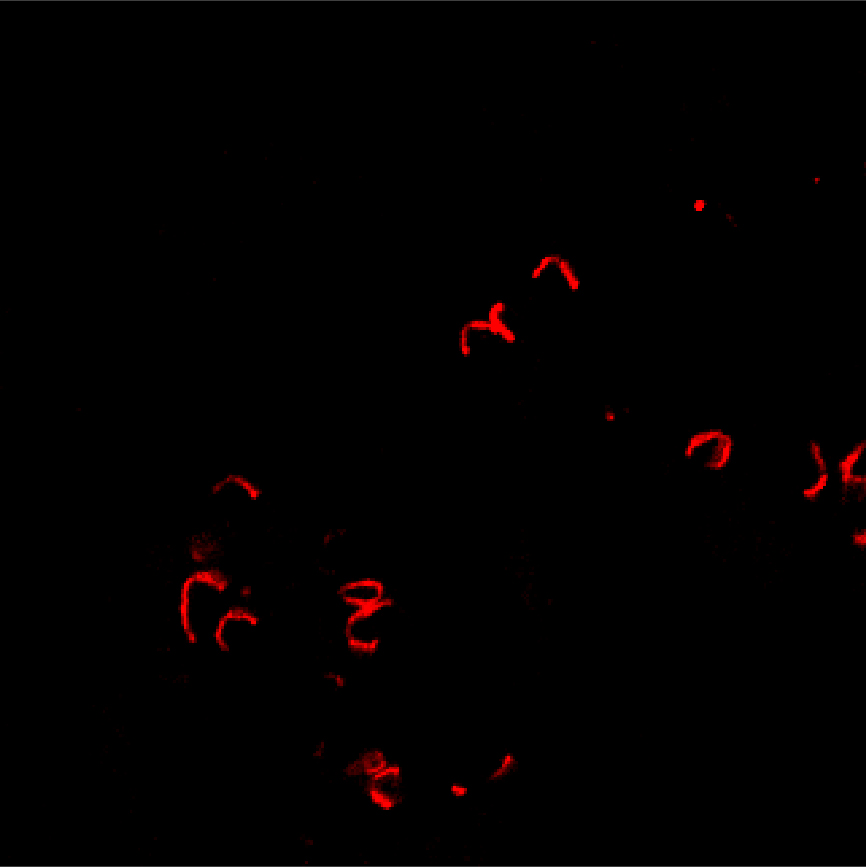

Supplement: Supplementary file 14 — Source data Fig. [file 44318_2025_659_MOESM14_ESM.zip › EMBOJ-2025-121587_Source Data/Source Data Figure 3/SD Figure 3E/Igf2bp3-/- PNA.jpg]

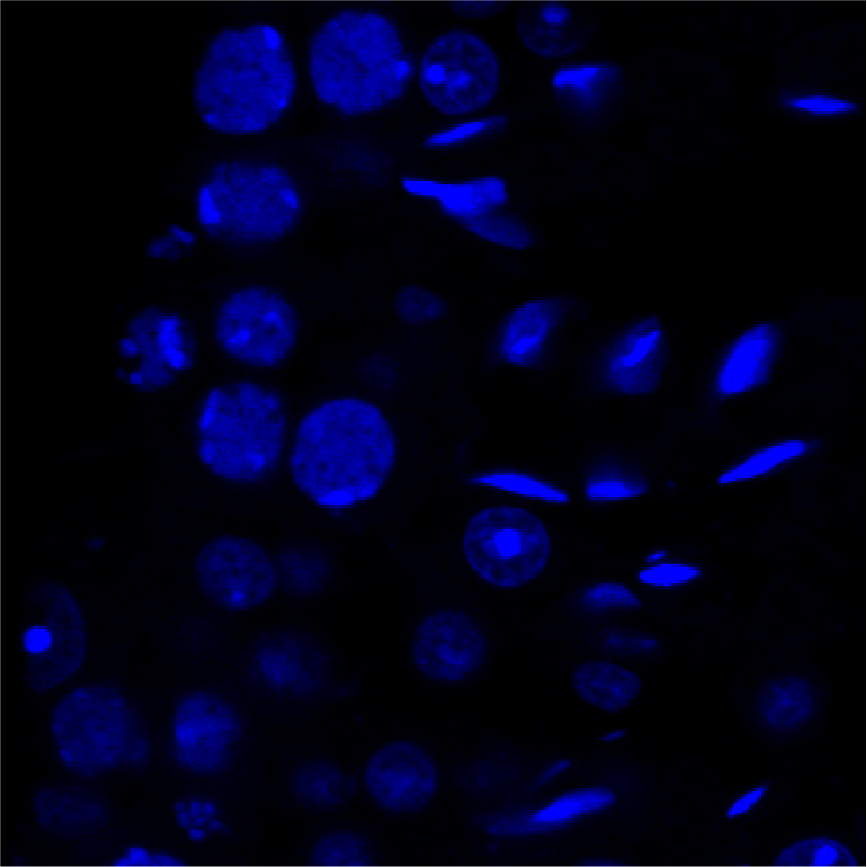

Supplement: Supplementary file 14 — Source data Fig. [file 44318_2025_659_MOESM14_ESM.zip › EMBOJ-2025-121587_Source Data/Source Data Figure 3/SD Figure 3F/Igf2bp3+/- Hoechst.jpg]
