# Supplementary figures and images for: IGF2BP3 recognizes m6A to regulate histone-to-protamine replacement during mouse sperm development (part 3 of 3)
Source: EMBO J. 2025 Dec 5;45(2):504–36. doi: 10.1038/s44318-025-00659-y (PMC12811620; doi:10.1038/s44318-025-00659-y)

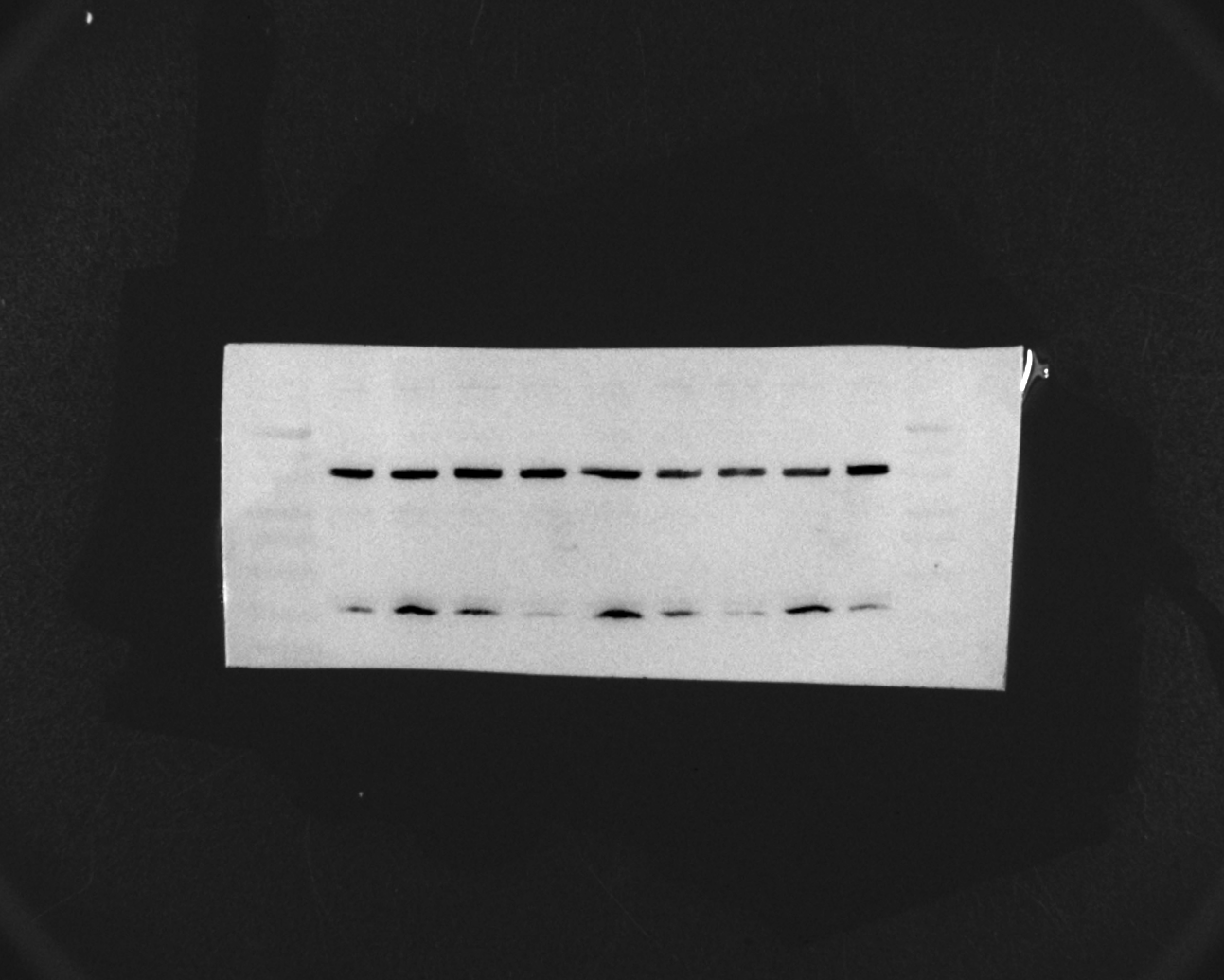

Supplement: Supplementary file 21 — Source data Fig. 7 [file 44318_2025_659_MOESM21_ESM.zip › Source Data Figure 7/SD Figure 7G/SD Figure 7G-H2A.tif]

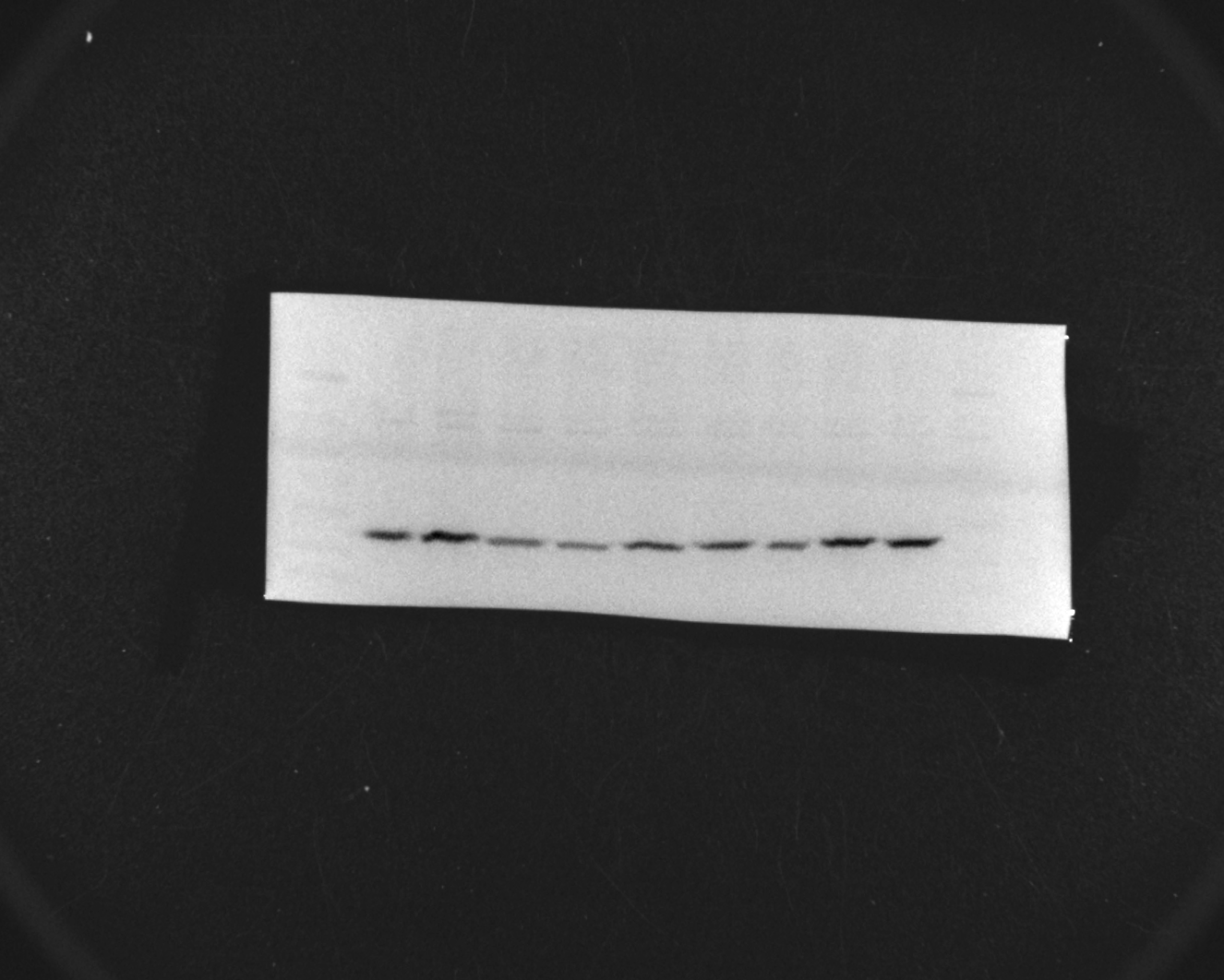

Supplement: Supplementary file 21 — Source data Fig. 7 [file 44318_2025_659_MOESM21_ESM.zip › Source Data Figure 7/SD Figure 7G/SD Figure 7G-H2B.tif]

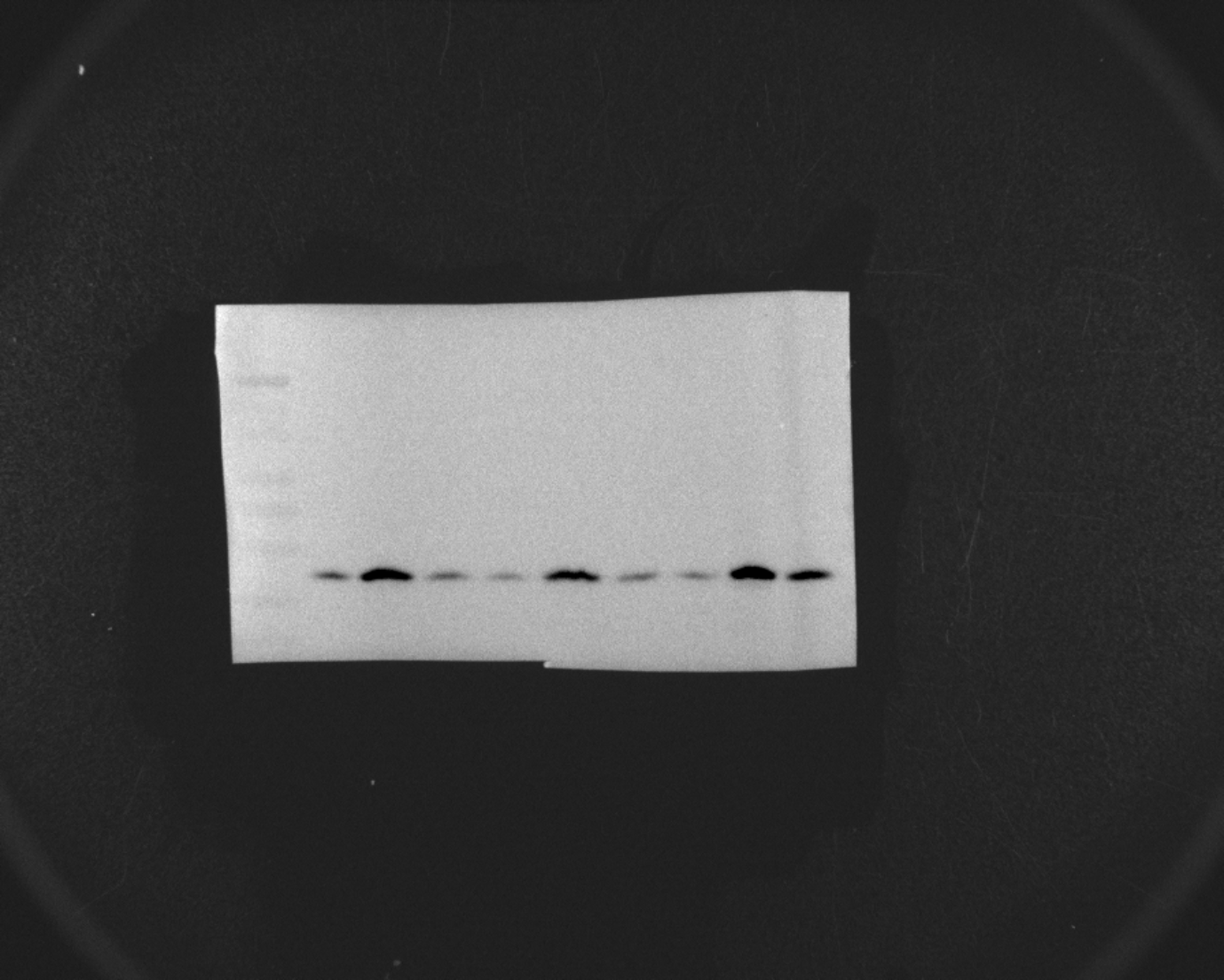

Supplement: Supplementary file 21 — Source data Fig. 7 [file 44318_2025_659_MOESM21_ESM.zip › Source Data Figure 7/SD Figure 7G/SD Figure 7G-H3.tif]

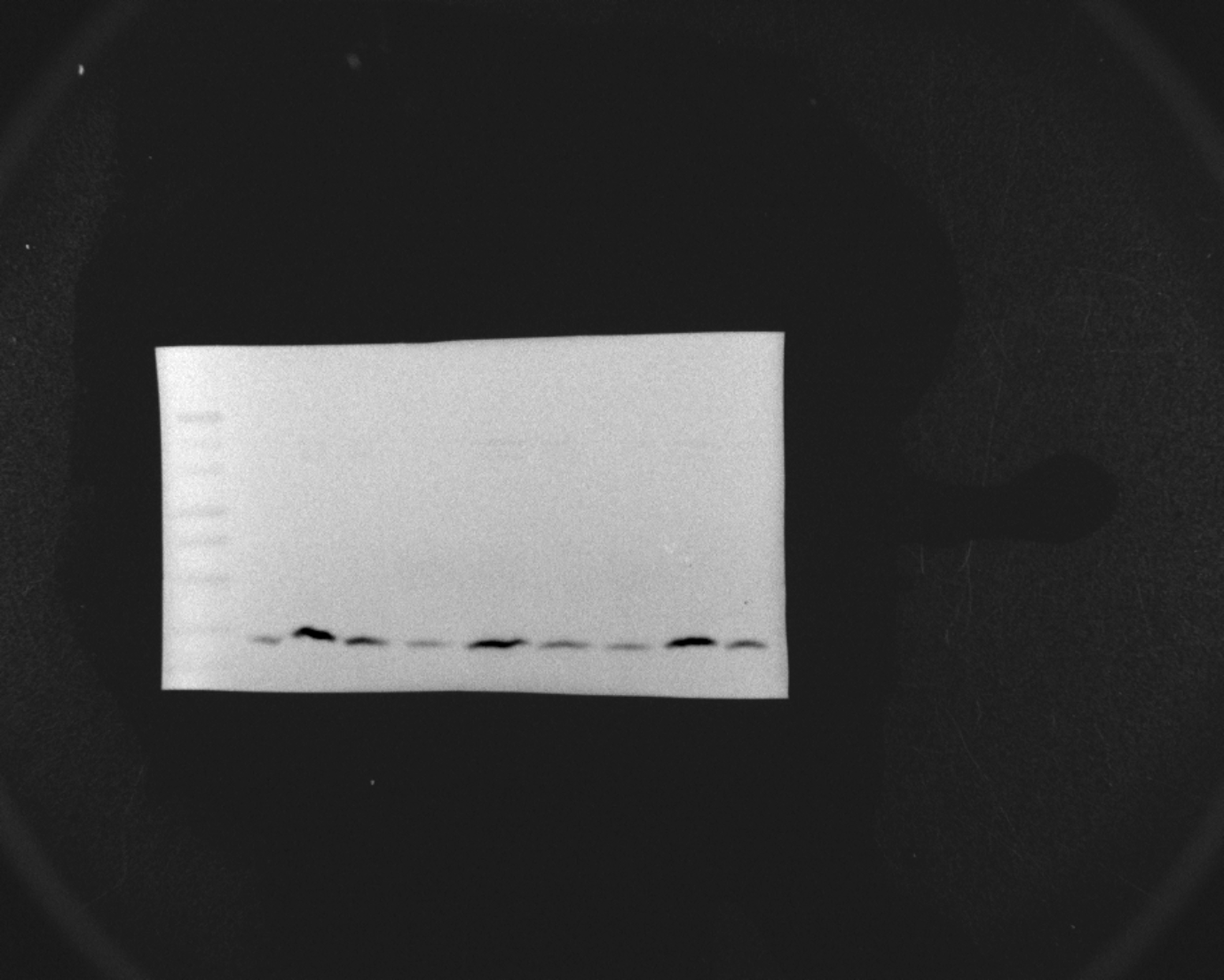

Supplement: Supplementary file 21 — Source data Fig. 7 [file 44318_2025_659_MOESM21_ESM.zip › Source Data Figure 7/SD Figure 7G/SD Figure 7G-H4.tif]

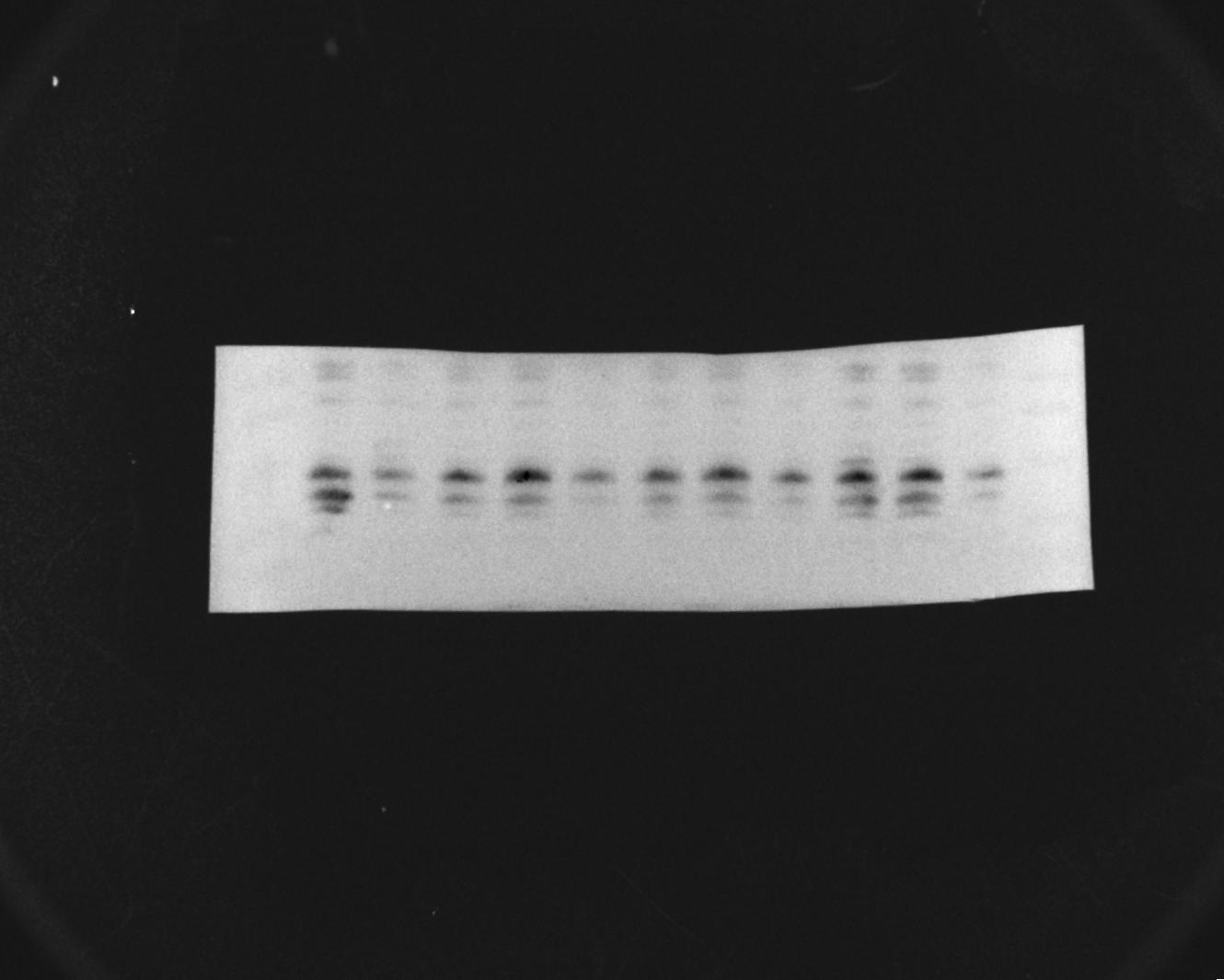

Supplement: Supplementary file 21 — Source data Fig. 7 [file 44318_2025_659_MOESM21_ESM.zip › Source Data Figure 7/SD Figure 7G/SD Figure 7G-PRM1.tif]

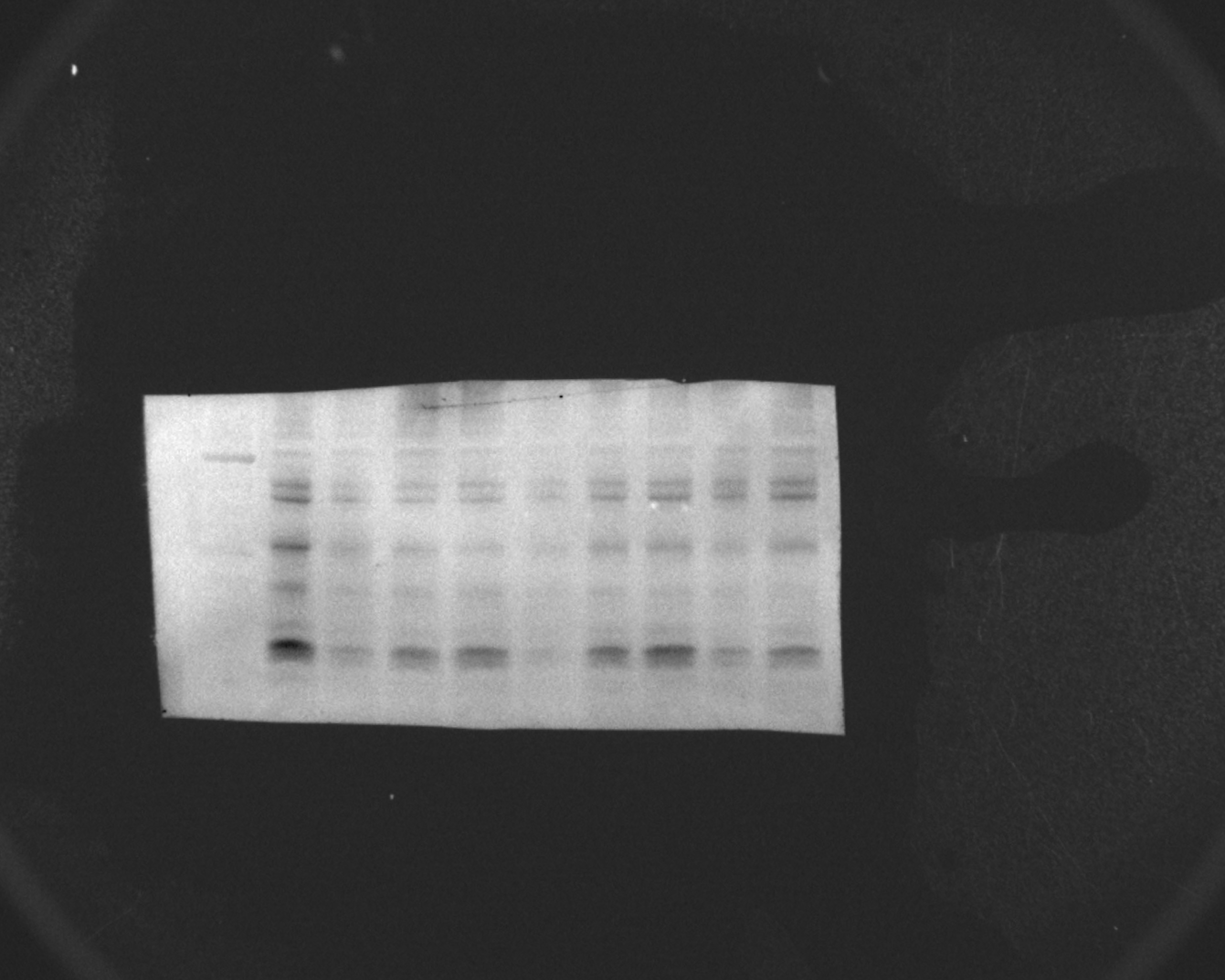

Supplement: Supplementary file 21 — Source data Fig. 7 [file 44318_2025_659_MOESM21_ESM.zip › Source Data Figure 7/SD Figure 7G/SD Figure 7G-PRM2.tif]

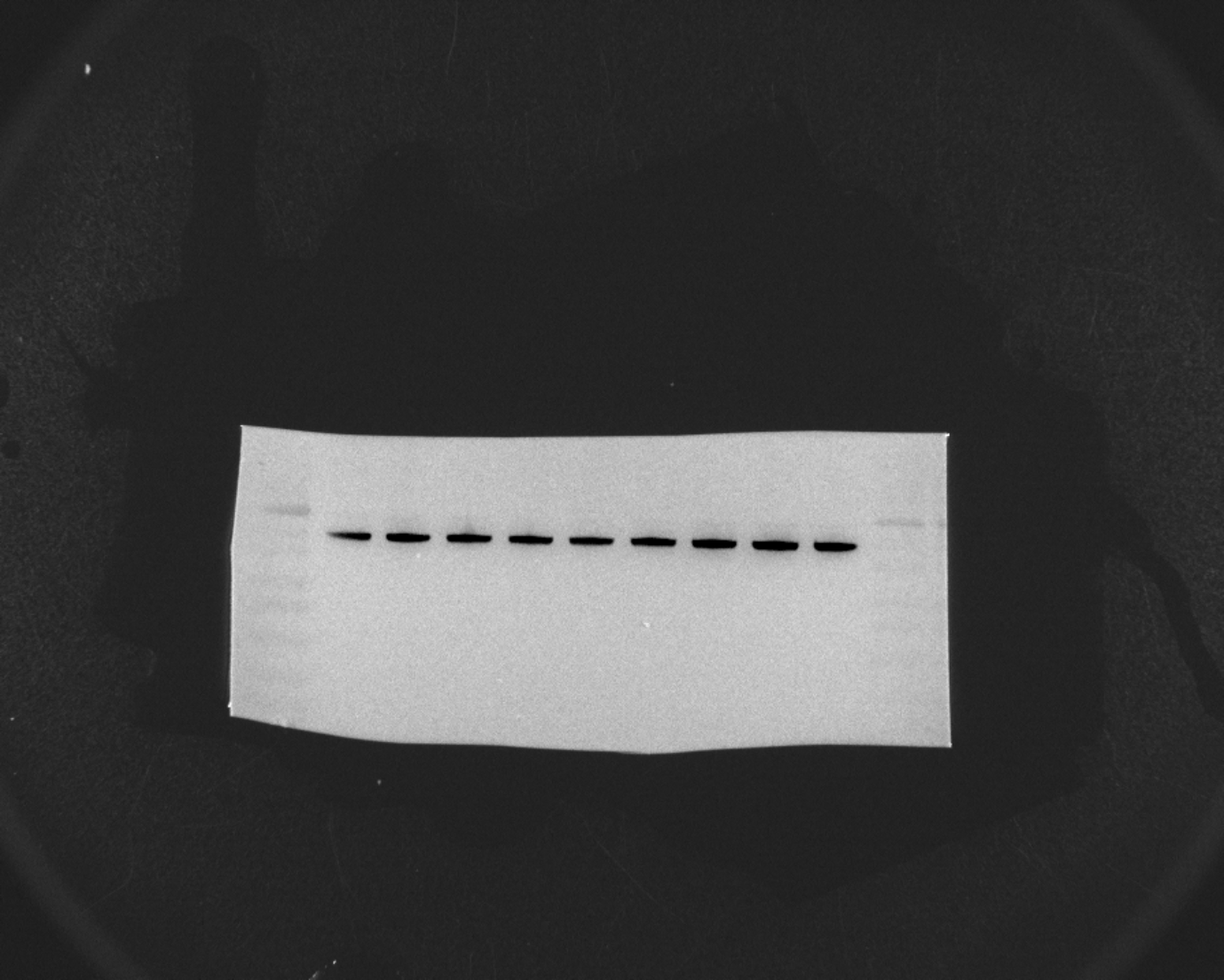

Supplement: Supplementary file 21 — Source data Fig. 7 [file 44318_2025_659_MOESM21_ESM.zip › Source Data Figure 7/SD Figure 7G/SD Figure 7G-TUBLIN.tif]

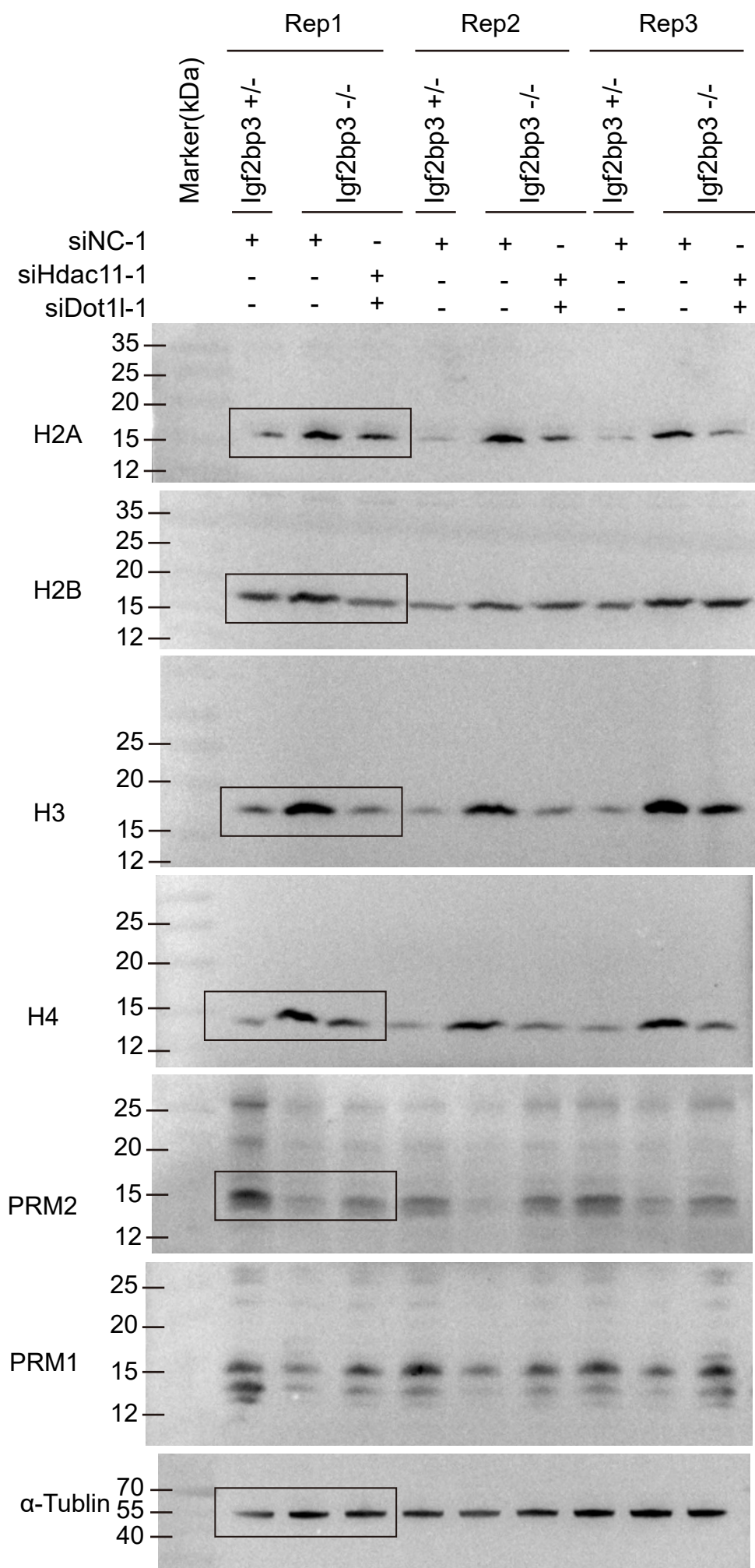

Supplement: Supplementary file 21 — Source data Fig. 7 [file 44318_2025_659_MOESM21_ESM.zip › Source Data Figure 7/SD Figure 7G/SD Figure 7G.pdf]
