# Supplementary material for: IGF2BP3 recognizes m6A to regulate histone-to-protamine replacement during mouse sperm development
Source: EMBO J. 2025 Dec 5;45(2):504–36. doi: 10.1038/s44318-025-00659-y (PMC12811620; doi:10.1038/s44318-025-00659-y)
Supplement: Supplementary file 22 — Expanded View Figures [file 44318_2025_659_MOESM22_ESM.pdf]

Expanded View Figures

Figure EV1. The strategy of *Igf2bp3*-KO mice and its spermatids loss in testis, related to Fig. 2.

(**A**) Schematic structures showing RNA-binding domains within IGF2BP3 proteins (top). Schematic diagram of CRISPR/Cas9 for generating the *Igf2bp3* knockout mice (middle). Sanger sequencing of the targeting locus of *Igf2bp3* in wild-type and *Igf2bp3*$^{-/-}$ mice (bottom). (**B**) Immunofluorescence of IGF2BP3 (top) and secondary antibody (bottom) in adult testicular sections from 8-week-old *Igf2bp3*$^{+/-}$ and *Igf2bp3*$^{-/-}$ mice. Scale bar, 20 µm. (**C**) Western blotting analysis of the protein levels of IGF2BP1, IGF2BP2, and IGF2BP3 in *Igf2bp3*$^{+/+}$, *Igf2bp3*$^{+/-}$, and *Igf2bp3*$^{-/-}$ testes. Both the N-terminal (ab177477) and C-terminal (A303-426A) recognizing antibodies of IGF2BP3 were utilized in this assay. ACTB serves as a loading control. (**D**, **E**) Computer-aided sperm analysis (CASA) of the sperm motility (**D**) and sperm number (**E**) in *Igf2bp3*$^{+/+}$ ($n = 16$), *Igf2bp3*$^{+/-}$ ($n = 18$), and *Igf2bp3*$^{-/-}$ ($n = 39$) mice. P values are calculated by one-way ANOVA. Each bar represents the mean ± SEM from biological replicates. (**F**) Quantitative comparison of abnormal nuclei per tubule between adult *Igf2bp3*$^{+/-}$ and *Igf2bp3*$^{-/-}$ testes. At least 20 tubules of each mouse were calculated. Unpaired two-tailed t test. Error bars, $n = 4$ biological replicates, mean ± SEM. (**G**) PAS staining of the adult testicular sections from 8-week-old *Igf2bp3*$^{+/+}$, *Igf2bp3*$^{+/-}$ and *Igf2bp3*$^{-/-}$ mice. Arrowheads indicate cells with abnormal agglutinated nuclei (left). Scale bar, 40 µm. Lumen area statistics on PAS staining of paraffin sections of *Igf2bp3*$^{+/+}$, *Igf2bp3*$^{+/-}$ and *Igf2bp3*$^{-/-}$ adult testes (right). P values are calculated by one-way ANOVA. Error bars, $n = 3$ biological replicates, mean ± SEM. (**H**) Flow cytometry analysis (left) and bar plot (right) showing the distribution of each ploidy population within adult *Igf2bp3*$^{+/-}$ and *Igf2bp3*$^{-/-}$ testes. Unpaired two-tailed Student's t test. Each error bar represents the mean ± SEM from 5 biological replicates.

© The Author(s)

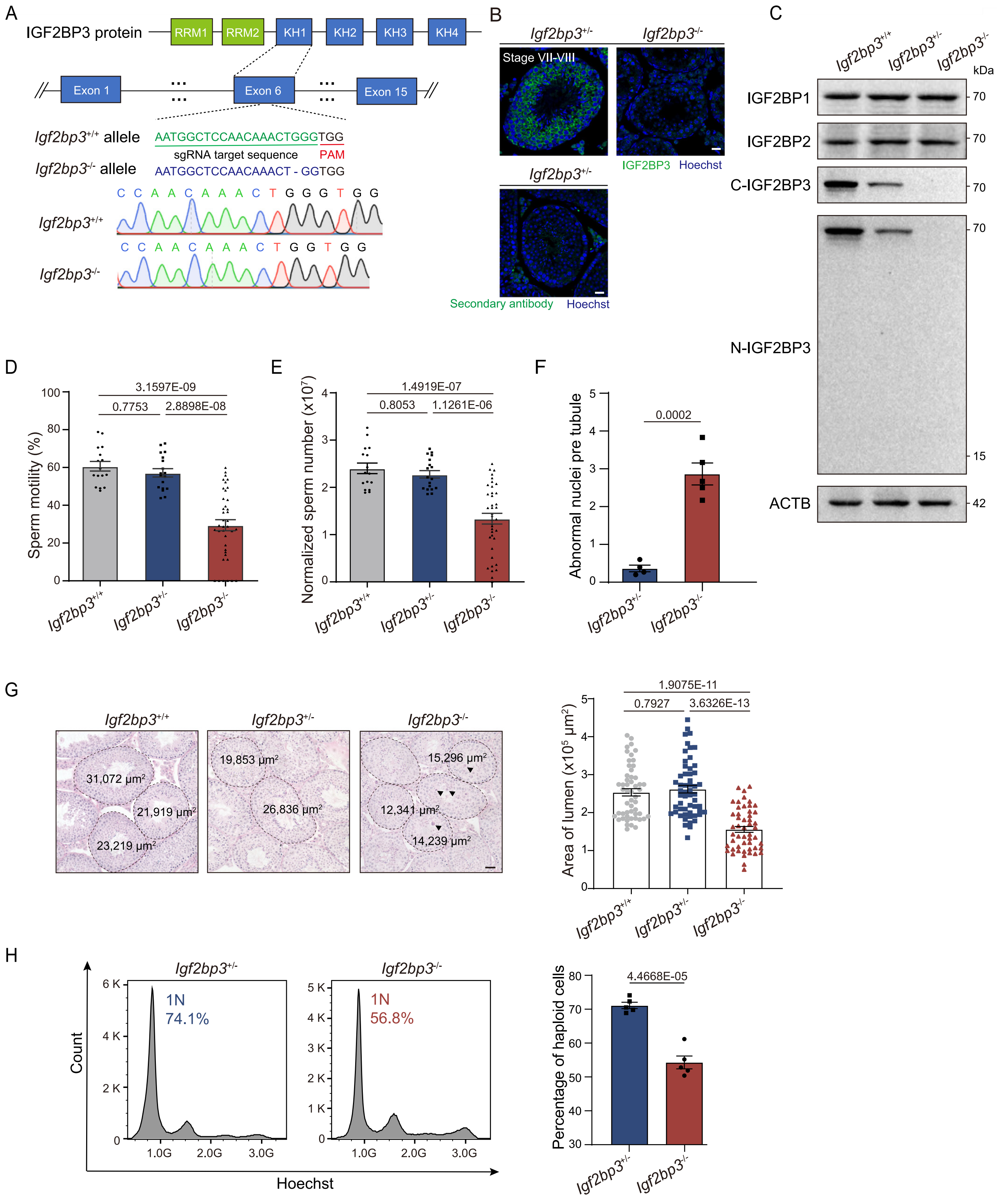
A
IGF2BP3 protein
RRM1
RRM2
KH1
KH2
KH3
KH4
Exon 1
Exon 6
Exon 15
Igf2bp3+/+ allele
AATGGCTCCAACAAACTGGGTGG
sgRNA target sequence
PAM
Igf2bp3-/- allele
AATGGCTCCAACAAACT - GGTGG
B
Igf2bp3+/-
Igf2bp3-/-
Stage VII-VIII
IGF2BP3 Hoechst
Secondary antibody Hoechst
C
IGF2BP1
IGF2BP2
C-IGF2BP3
N-IGF2BP3
ACTB
kDa
70
42
15
D
Sperm motility (%)
3.1597E-09
0.7753
2.8898E-08
E
Normalized sperm number (x10^7)
1.4919E-07
0.8053
1.1261E-06
F
Abnormal nuclei pre tubule
0.0002
G
31,072 μm²
21,919 μm²
23,219 μm²
19,853 μm²
26,836 μm²
15,296 μm²
12,341 μm²
14,239 μm²
Area of lumen (x10^5 μm²)
1.9075E-11
0.7927
3.6326E-13
H
Count
Hoechst
1N
74.1%
56.8%
1.0G
2.0G
3.0G
Percentage of haploid cells
4.4668E-05

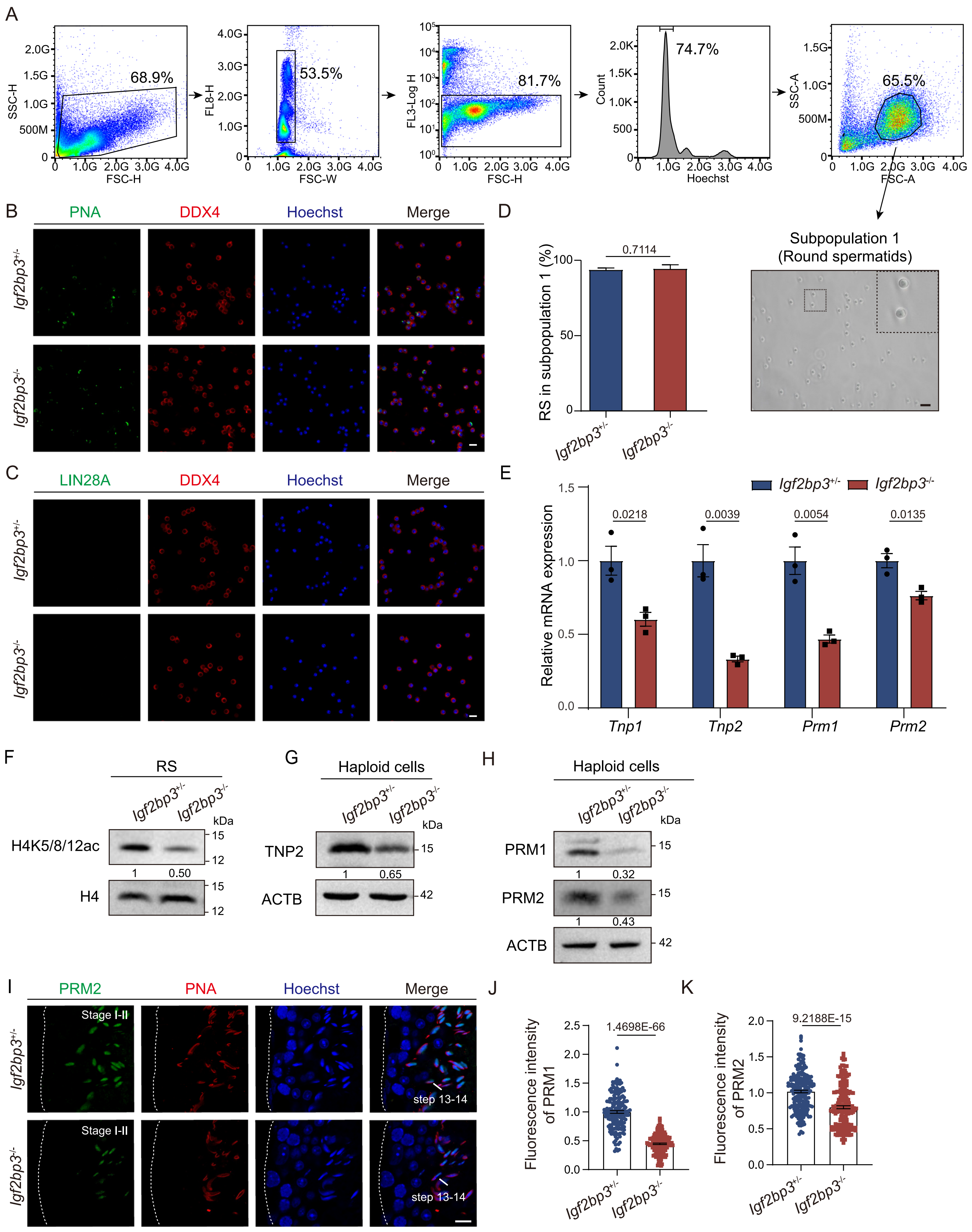
A
SSC-H
FSC-H
68.9%
FL8-H
FSC-W
53.5%
FL3-Log H
81.7%
Count
Hoechst
74.7%
SSC-A
FSC-A
65.5%
B
PNA
DDX4
Hoechst
Merge
Igf2bp3+/-
Igf2bp3-/-
C
LIN28A
D
RS in subpopulation 1 (%)
0.7114
Subpopulation 1
(Round spermatids)
E
Relative mRNA expression
0.0218
0.0039
0.0054
0.0135
Tnp1
Tnp2
Prm1
Prm2
F
RS
H4K5/8/12ac
H4
kDa
1
0.50
G
Haploid cells
TNP2
ACTB
1
0.65
H
PRM1
PRM2
1
0.32
1
0.43
I
PRM2
Stage I-II
step 13-14
J
Fluorescence intensity of PRM1
1.4698E-66
K
Fluorescence intensity of PRM2
9.2188E-15

 © The Author(s)

Figure EV2. The down-regulation of transition proteins and protamines in *Igf2bp3*-KO spermatids, related to Fig. 3.

(**A**) Representative FACS strategy for isolating round spermatids from *Igf2bp3*$^{+/-}$ and *Igf2bp3*$^{-/-}$ testes. Isolated round spermatids showing in the bright field image. Scale bar, 80 µm. (**B**, **C**) Immunostaining of PNA or LIN28A (green), and DDX4 (red) in round spermatids sorted from *Igf2bp3*$^{+/-}$ and *Igf2bp3*$^{-/-}$ testes, respectively. Scale bar, 20 µm. (**D**) Percentage of round spermatids in Subpopulation 1 sorted from adult *Igf2bp3*$^{+/-}$ and *Igf2bp3*$^{-/-}$ testes. Unpaired two-tailed *t* test. Error bars, $n = 3$ biological replicates, mean ± SEM. (**E**) qPCR analysis of the relative expression levels of *Tnp1*, *Tnp2*, *Prm1*, and *Prm2* normalized to *β-Actin* in *Igf2bp3*$^{+/-}$ and *Igf2bp3*$^{-/-}$ RS. Unpaired two-tailed *t* test. Error bars, $n = 3$ biological replicates, mean ± SEM. (**F**) Western blotting analysis of the protein levels of H4K5/8/12ac in *Igf2bp3*$^{+/-}$ and *Igf2bp3*$^{-/-}$ RS. The values below each band represent the relative expression levels of each protein, normalized using H4 as a loading control. (**G**, **H**) Western blotting analysis of the protein levels of TNP2 (**G**), PRM1 and PRM2 (**H**) in haploid cells from adult *Igf2bp3*$^{+/-}$ and *Igf2bp3*$^{-/-}$ testes. The values below each band represent the relative expression levels of each protein, normalized using ACTB as a loading control. (**I**) Immunofluorescence of PRM2 (green) and PNA (red) in adult testicular paraffin sections from 8-week-old *Igf2bp3*$^{+/-}$ and *Igf2bp3*$^{-/-}$ mice. Scale bar, 10 µm. Dotted borders demarcates the basement membrane of the seminiferous tubule. (**J**, **K**) Quantification of fluorescence intensity of PRM1 (Fig. 3G) and PRM2 (Fig. EV2I) in paraffin sections of adult *Igf2bp3*$^{+/-}$ and *Igf2bp3*$^{-/-}$ mouse testes. Unpaired two-tailed *t* test. Error bars, $n =$ over 150 cells from 3 biological replicates, mean ± SEM.

© The Author(s)

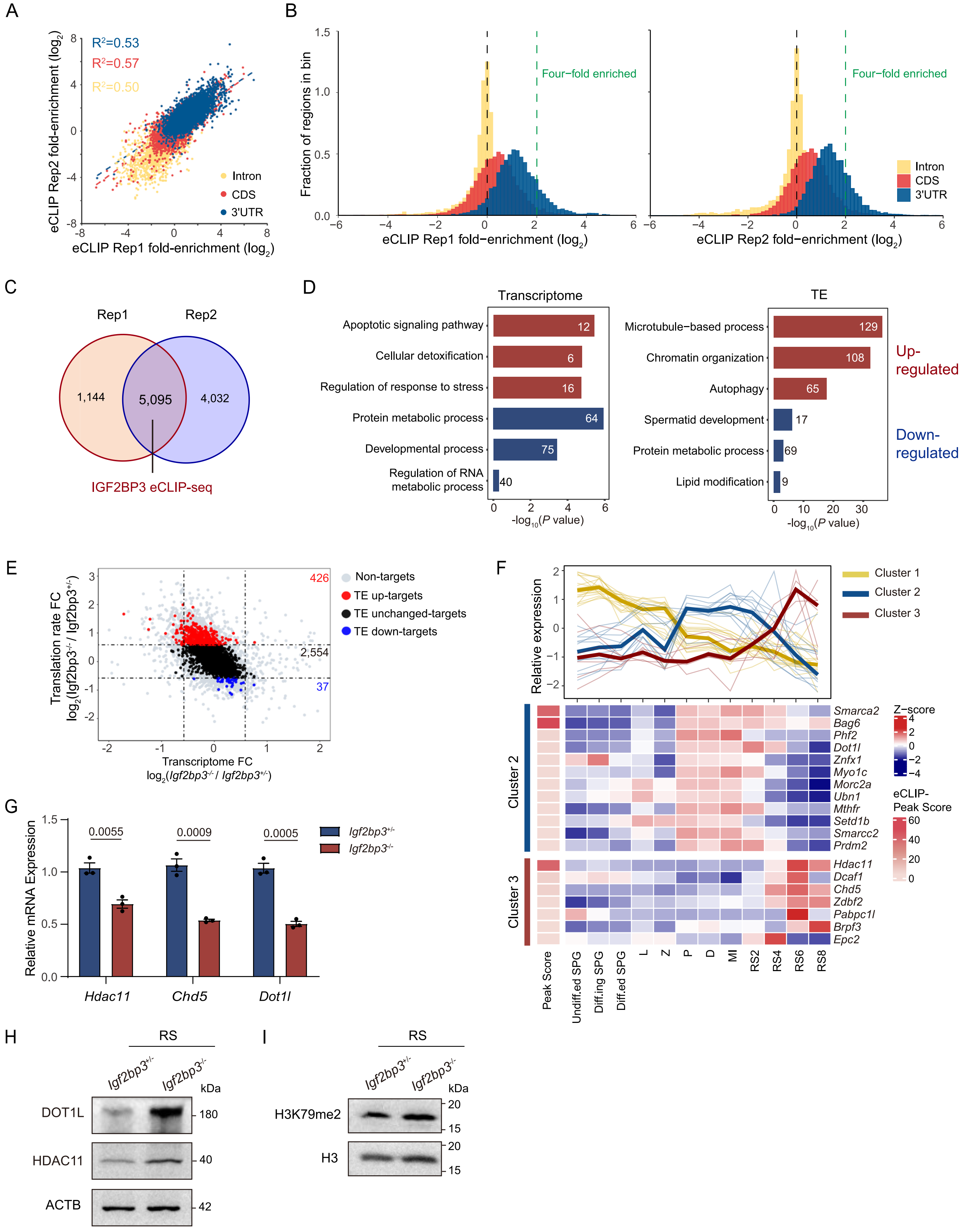
A
R²=0.53
R²=0.57
R²=0.50
eCLIP Rep2 fold-enrichment (log2)
eCLIP Rep1 fold-enrichment (log2)
Intron
CDS
3'UTR
B
Fraction of regions in bin
Four-fold enriched
eCLIP Rep1 fold-enrichment (log2)
eCLIP Rep2 fold-enrichment (log2)
C
Rep1
Rep2
1,144
5,095
4,032
IGF2BP3 eCLIP-seq
D
Transcriptome
Apoptotic signaling pathway
Cellular detoxification
Regulation of response to stress
Protein metabolic process
Developmental process
Regulation of RNA metabolic process
-log10(P value)
TE
Microtubule-based process
Chromatin organization
Autophagy
Spermatid development
Protein metabolic process
Lipid modification
Up-regulated
Down-regulated
E
Non-targets
TE up-targets
TE unchanged-targets
TE down-targets
426
2,554
37
Translation rate FC log2(Igf2bp3-/- / Igf2bp3+/-)
Transcriptome FC log2(Igf2bp3-/- / Igf2bp3+/-)
F
Relative expression
Cluster 1
Cluster 2
Cluster 3
Z-score
eCLIP-Peak Score
Smarca2
Bag6
Phf2
Dot1l
Znfx1
Myo1c
Morc2a
Ubn1
Mthfr
Setd1b
Smarcc2
Prdm2
Hdac11
Dcaf1
Chd5
Zdbf2
Pabpc1l
Brpf3
Epc2
Peak Score
Undiff.ed SPG
Diff.ing SPG
Diff.ed SPG
L
Z
P
D
MI
RS2
RS4
RS6
RS8
G
Relative mRNA Expression
0.0055
0.0009
0.0005
Igf2bp3+/-
Igf2bp3-/-
Hdac11
Chd5
Dot1l
H
RS
Igf2bp3+/-
Igf2bp3-/-
kDa
DOT1L
HDAC11
ACTB
180
40
42
I
RS
H3K79me2
H3
20
15

 © The Author(s)

Figure EV3. Testicular eCLIP-seq of IGF2BP3 and translation efficiency changes between *Igf2bp3*$^{+/-}$ and *Igf2bp3*$^{-/-}$ round spermatids, related to Fig. 4.

(**A**) eCLIP fold enrichment comparison across replicates and samples. Fold enrichment of IGF2BP3 IP1 over paired SMInput1 (*x* axis) versus IGF2BP3 IP2 over paired SMInput2 (*y* axis). Dotted lines and R^2 values indicate least-squares regression performed separately for each region type. CDS, coding sequences; UTR, untranslated regions. Rep, replicates. (**B**) Histogram of region-based fold enrichment for IGF2BP3 (each compared to its paired SMInput). Left, Rep1; right, Rep2. (**C**) Venn diagram showing the 5,095 overlapping target genes from two IGF2BP3 eCLIP-seq biological replicates. (**D**) Top GO terms in biological process categories of down- and upregulated genes at transcriptional-level (left) and TE-level (right). Fisher's exact test with g:Profiler was used in GO enrichment analysis. (**E**) Scatter plot showing mRNA-level changes (*x* axis) against translational efficiencies (TE) changes (*y* axis) between adult *Igf2bp3*$^{-/-}$ and *Igf2bp3*$^{+/-}$ round spermatids. Red dots indicate TE up-targets. Blue dots indicate TE down-targets. Black dots indicate TE unchanged-targets. Gray dots indicate non-targets of IGF2BP3. (**F**) Clustering analysis of 37 TE upregulated targets associated with 'chromatin organization' related to Fig. 4G (top). Heatmap showing the genes in cluster 2 and cluster 3 (bottom). (**G**) qPCR analysis of the relative expression levels of indicated transcripts in round spermatids from adult *Igf2bp3*$^{+/-}$ and *Igf2bp3*$^{-/-}$ testes. Unpaired two-tailed *t* test. Error bars, $n = 3$ biological replicates, mean ± SEM. (**H**) Western blotting analysis of the protein levels of DOT1L and HDAC11 in *Igf2bp3*$^{+/-}$ and *Igf2bp3*$^{-/-}$ RS. ACTB serves as a loading control. (**I**) Western blotting analysis of the protein levels of H3K79me2 in *Igf2bp3*$^{+/-}$ and *Igf2bp3*$^{-/-}$ RS. H3 serves as a loading control.

© The Author(s)

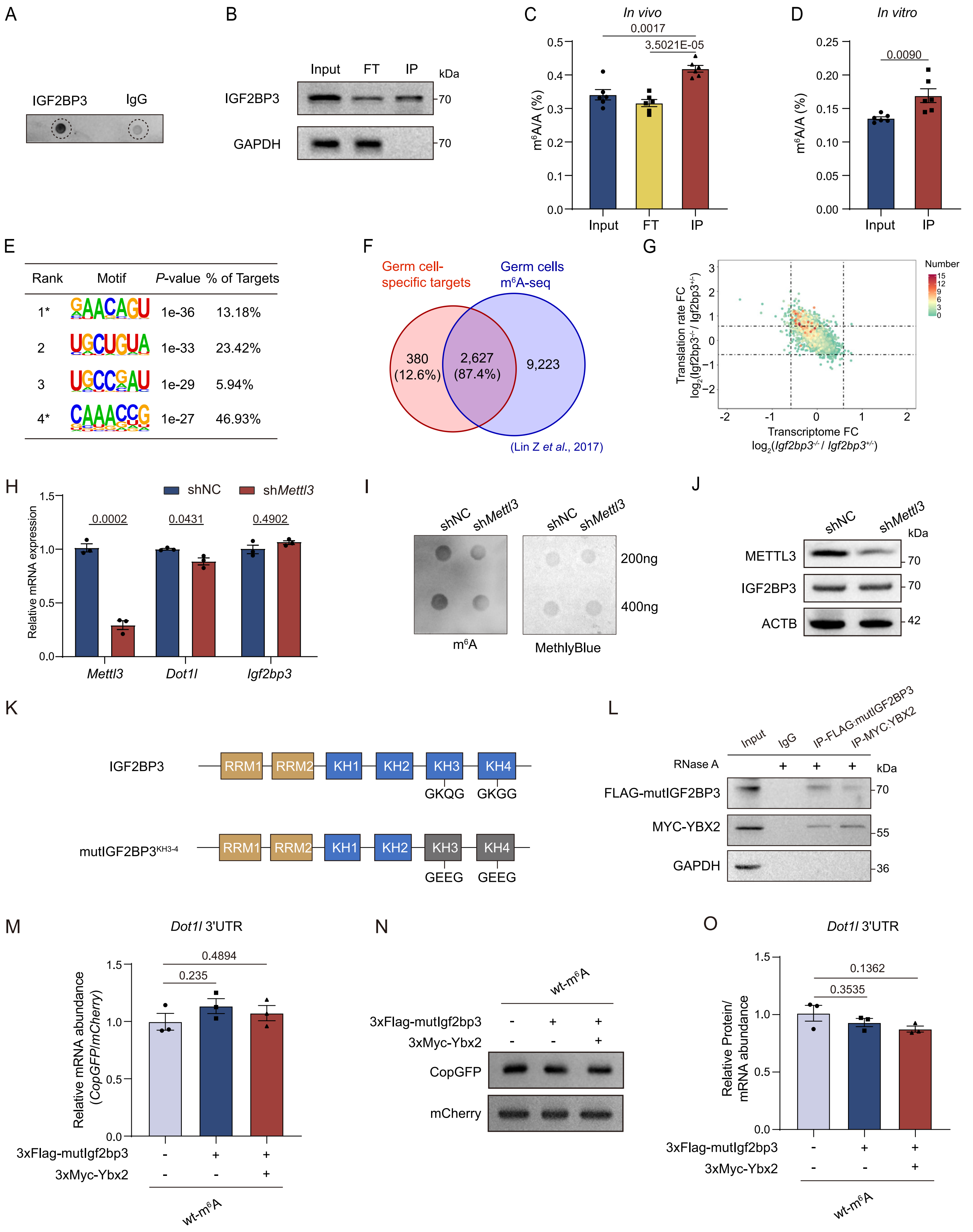
A
IGF2BP3
IgG
B
Input
FT
IP
kDa
IGF2BP3
GAPDH
70
70
C
In vivo
0.0017
3.5021E-05
m6A/A (%)
Input
FT
IP
D
In vitro
0.0090
m6A/A (%)
Input
IP
E
Rank
Motif
P-value
% of Targets
1* GAACAGU 1e-36 13.18%
2 UGCUGUA 1e-33 23.42%
3 UGCCGAU 1e-29 5.94%
4* CAAACCG 1e-27 46.93%
F
Germ cell-specific targets
Germ cells m6A-seq
380 (12.6%)
2,627 (87.4%)
9,223
(Lin Z et al., 2017)
G
Number
Translation rate FC log2(Igf2bp3-/- / Igf2bp3+/-)
Transcriptome FC log2(Igf2bp3-/- / Igf2bp3+/-)
H
shNC
shMettl3
0.0002
0.0431
0.4902
Relative mRNA expression
Mettl3
Dot1l
Igf2bp3
I
shNC
shMettl3
200ng
400ng
m6A
MethlyBlue
J
shNC
shMettl3
kDa
METTL3
IGF2BP3
ACTB
70
70
42
K
IGF2BP3
RRM1
RRM2
KH1
KH2
KH3
KH4
GKQG
GKGG
mutIGF2BP3KH3-4
GEEG
GEEG
L
Input
IgG
IP-FLAG:mutIGF2BP3
IP-MYC:YBX2
RNase A
kDa
FLAG-mutIGF2BP3
MYC-YBX2
GAPDH
70
55
36
M
Dot1l 3'UTR
0.4894
0.235
Relative mRNA abundance (CopGFP/mCherry)
3xFlag-mutIgf2bp3
3xMyc-Ybx2
wt-m6A
N
wt-m6A
3xFlag-mutIgf2bp3
3xMyc-Ybx2
CopGFP
mCherry
O
Dot1l 3'UTR
0.1362
0.3535
Relative Protein/ mRNA abundance
3xFlag-mutIgf2bp3
3xMyc-Ybx2
wt-m6A

 © The Author(s)

Figure EV4. IGF2BP3 recognizes and regulates m^{6}A-containing RNAs in mouse testes, related to Fig. 6.

(**A**) Dot blotting analysis of the m^{6}A level in IGF2BP3-bound RNAs isolated from adult testes with IgG as the control. (**B**) Western blotting analysis using adult mouse testicular lysates prior to RIP. (**C**) RIP LC-MS/MS showing m^{6}A enrichment in IGF2BP3-bound RNAs while depleted in the flow-through (FT) portion (mouse testes) ($n = 6$, two technical replicates over three biologically replicates). Unpaired two-tailed t test. Error bars, mean ± SEM. (**D**) LC-MS/MS showing m^{6}A enrichment in His6-IGF2BP3 bound mRNA portion. ($n = 6$, two technical replicates over three biologically replicates). Unpaired two-tailed t test. Error bars, mean ± SEM. (**E**) Top consensus sequences on overlapping IGF2BP3-bound peaks using HOMER. The motifs marked with * represent m^{6}A motifs. (**F**) Venn diagram showing the overlap between 3,007 germ cell-specific targets of IGF2BP3 identified in Fig. 4C and m^{6}A-modified mRNA in testicular germ cells (Lin et al, 2017). (**G**) Scatter plot showing the number of m^{6}A-modified sites of IGF2BP3 targets with the mRNA-level changes (x axis) against TE changes (y axis) between adult *Igf2bp3*$^{-/-}$ and *Igf2bp3*$^{+/-}$ round spermatids. (**H**) qPCR analyses of the relative expression levels of *Mettl3*, *Dot1l* and *Igf2bp3* mRNA normalized to *β-Actin* in GC-2 cells treated with sh*Mettl3* or control shRNA. Unpaired two-tailed t test. Error bars, $n = 3$ biological replicates, mean ± SEM. (**I**) Dot blotting analysis of the global m^{6}A level of RNA extracted from *Mettl3*-knockdown or control GC-2 cells. (**J**) Western blotting analysis of the protein levels of METTL3 and IGF2BP3 in GC-2 cells treated with sh*Mettl3* or control shRNA. ACTB serves as a loading control. (**K**) Schematic structures showing RNA-binding domains within IGF2BP proteins and a summary of IGF2BP variants used in this study. Yellow boxes are RRM domains, blue boxes are wild-type KH domains with GxxG motifs, and grey boxes are inactive KH domains with GxxG converted to GEEG. (**L**) Western blotting analysis of FLAG-mutIGF2BP3 and MYC-YBX2 in the Flag-mutant *Igf2bp3* and Myc-*Ybx2* co-transfected HEK293T cell lysates (input), and the lysate immunoprecipitation with anti-IgG, anti-FLAG or anti-MYC antibodies treated with RNase A (+), respectively. (**M**) qPCR analyses of the relative levels of *CopGFP* mRNAs normalized to *mCherry* mRNAs. Cell lines were treated with 2 μg/ml actinomycin D for 2 h. Unpaired two-tailed t test. Error bars, $n = 3$ biological replicates, mean ± SEM. (**N**) Western blotting analysis of the protein levels of CopGFP under the regulation of *Dot1l* 3′UTR with the overexpression of FLAG-tagged mutant IGF2BP3 or MYC-tagged YBX2. The level of mCherry is set as the internal control. (**O**) Histogram showing the ratios of CopGFP proteins (normalized to mCherry proteins) to the *CopGFP* mRNAs (normalized to *mCherry* mRNAs), corresponding to Fig. EV4M,N. Unpaired two-tailed t test. Error bars, $n = 3$ biological replicates, mean ± SEM.

© The Author(s)

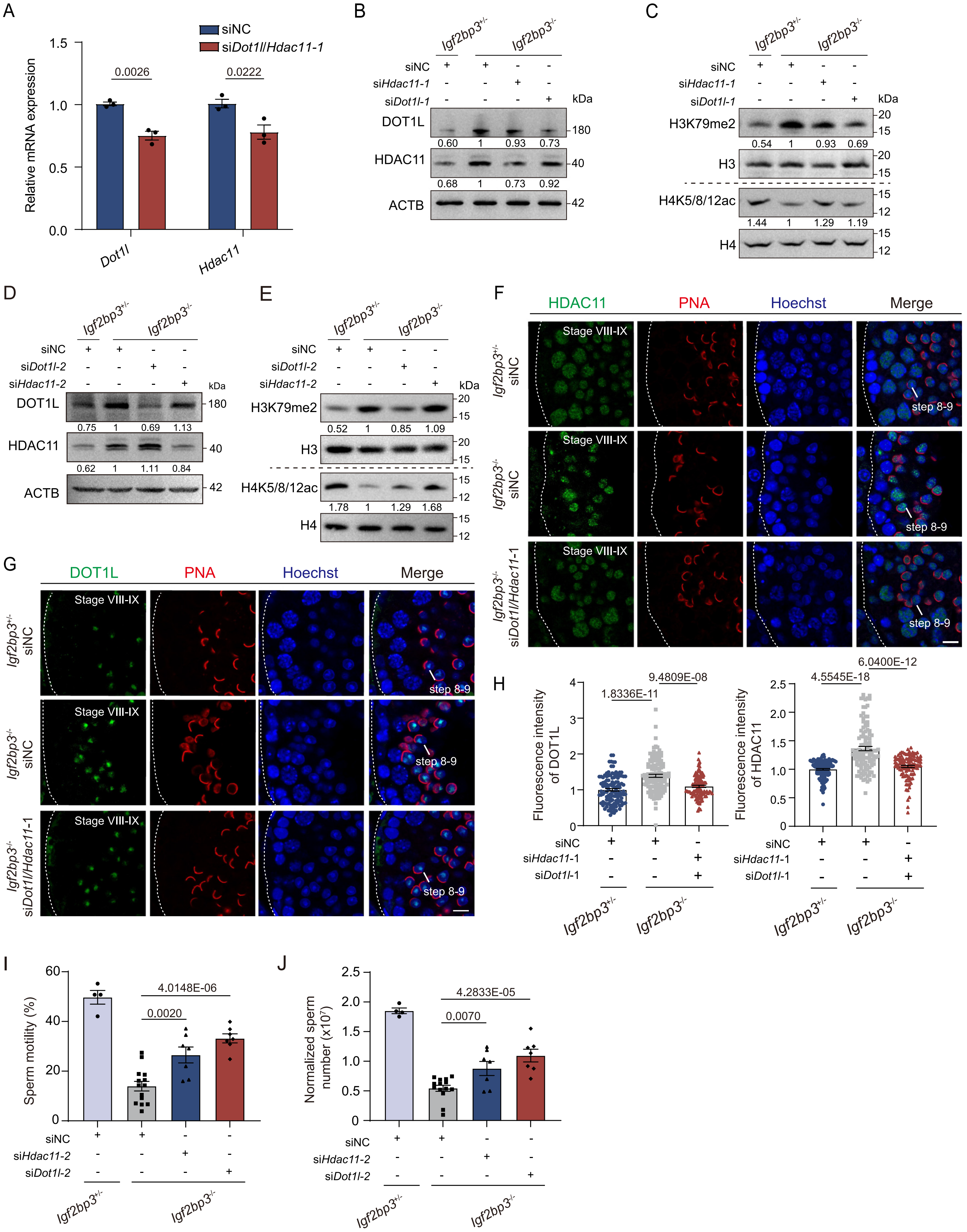
A
siNC
siDot1l/Hdac11-1
Relative mRNA expression
0.0026
0.0222
Dot1l
Hdac11
B
Igf2bp3+/-
Igf2bp3-/-
siNC
siHdac11-1
siDot1l-1
kDa
DOT1L
HDAC11
ACTB
0.60 1 0.93 0.73
0.68 1 0.73 0.92
C
H3K79me2
H3
H4K5/8/12ac
H4
0.54 1 0.93 0.69
1.44 1 1.29 1.19
D
siDot1l-2
siHdac11-2
0.75 1 0.69 1.13
0.62 1 1.11 0.84
E
0.52 1 0.85 1.09
1.78 1 1.29 1.68
F
HDAC11
PNA
Hoechst
Merge
Stage VIII-IX
step 8-9
Igf2bp3-/- siDot1l/Hdac11-1
G
DOT1L
H
Fluorescence intensity of DOT1L
Fluorescence intensity of HDAC11
1.8336E-11
9.4809E-08
4.5545E-18
6.0400E-12
I
Sperm motility (%)
0.0020
4.0148E-06
J
Normalized sperm number (x10^7)
0.0070
4.2833E-05

 © The Author(s)

Figure EV5. Testicular DOT1L and HDAC11 down-regulation rescue the sperm developmental disorders in IGF2BP3-KO testes, related to Fig. 7.

(**A**) qPCR analyses of the relative expression levels of *Dot1l* and *Hdac11* mRNAs normalized to *β-Actin* in RS from *Igf2bp3*$^{-/-}$ mouse testes injected with siNC or si*Dot1l/Hdac11*-1. Unpaired two-tailed *t* test. Error bars, $n = 3$ biological replicates, mean ± SEM. (**B–E**) Western blotting analysis of the protein levels of DOT1L, HDAC11, H3K79me2 and H4K5/8/12ac from adult *Igf2bp3*$^{+/-}$ and *Igf2bp3*$^{-/-}$ mouse testes injected with negative control siRNA (siNC), siRNA targeting *Dot1l* (si*Dot1l*-1/2) or siRNA targeting *Dot1l* (si*Hdac11*-1/2). The values below each band represent the relative expression levels of each protein. ACTB serves as the internal control of DOT1L and HDAC11, H3 serves as the internal control of H3K79me2, H4 serves as the internal control of H4Ac. (**F**, **G**) Immunofluorescence of HDAC11 or DOT1L (green) and PNA (red) in paraffin sections of adult *Igf2bp3*$^{+/-}$ and *Igf2bp3*$^{-/-}$ mouse testes injected with siNC or si*Dot1l/Hdac11*-1. Scale bar, 10 µm. Dotted borders demarcates the basement membrane of the seminiferous tubule. (**H**) Quantification of fluorescence intensity of DOT1L (left) and HDAC11 (right) in paraffin sections of adult *Igf2bp3*$^{+/-}$ and *Igf2bp3*$^{-/-}$ mouse testes injected with siNC or si*Dot1l/Hdac11*-1, corresponding to Fig. EV5F,G. Unpaired two-tailed *t* test. Error bars, $n =$ over 88 cells from 3 biological replicates, mean ± SEM. (**I**, **J**) CASA of the percentage of motile sperm (**I**) and the corresponding concentration of epididymal spermatozoa (**J**) from adult *Igf2bp3*$^{+/-}$ and *Igf2bp3*$^{-/-}$ mouse injected with siNC, si*Dot1l*-2 or si*Hdac11*-2. Unpaired two-tailed *t* test. Each bar represents the mean ± SEM from 7 biological replicates.

© The Author(s)
