# Supplementary material for: IGF2BP3 recognizes m6A to regulate histone-to-protamine replacement during mouse sperm development
Source: EMBO J. 2025 Dec 5;45(2):504–36. doi: 10.1038/s44318-025-00659-y (PMC12811620; doi:10.1038/s44318-025-00659-y)
Supplement: Supplementary file 14 — Source data Fig. [file SD Figure 1A.pdf › EMBOJ-2025-121587_Source Data/Source Data Figure 1/SD Figure 1A/SD Figure 1A.pdf]

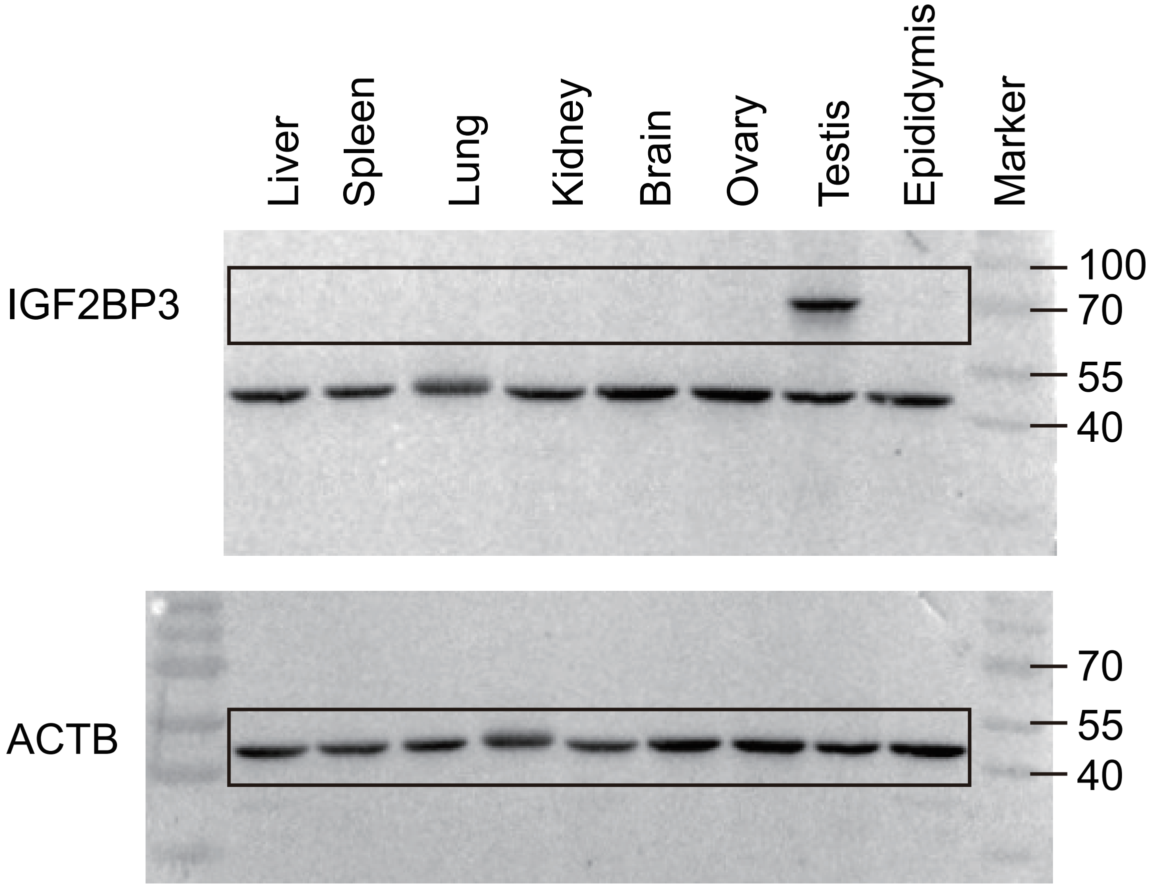
Liver
Spleen
Lung
Kidney
Brain
Ovary
Testis
Epididymis
Marker
IGF2BP3
ACTB
100
70
55
40
70
55
40
